# Identification of novel candidate drivers connecting different dysfunctional levels for lung adenocarcinoma using protein-protein interactions and a shortest path approach

**DOI:** 10.1038/srep29849

**Published:** 2016-07-14

**Authors:** Lei Chen, Tao Huang, Yu-Hang Zhang, Yang Jiang, Mingyue Zheng, Yu-Dong Cai

**Affiliations:** 1 School of Life Sciences, Shanghai University, Shanghai 200444, People’s Republic of China; 2 College of Information Engineering, Shanghai Maritime University, Shanghai 201306, People’s Republic of China; 3 Institute of Health Sciences, Shanghai Institutes for Biological Sciences, Chinese Academy of Sciences, Shanghai 200031, People’s Republic of China; 4 Department of Surgery, China-Japan Union Hospital of Jilin University, Changchun 130033, People’s Republic of China; 5 Drug Discovery and Design Center, State Key Laboratory of Drug Research, Shanghai Institute of Materia Medica, Chinese Academy of Sciences, Shanghai 201203, People’s Republic of China

## Abstract

Tumors are formed by the abnormal proliferation of somatic cells with disordered growth regulation under the influence of tumorigenic factors. Recently, the theory of “cancer drivers” connects tumor initiation with several specific mutations in the so-called cancer driver genes. According to the differentiation of four basic levels between tumor and adjacent normal tissues, the cancer drivers can be divided into the following: (1) Methylation level, (2) microRNA level, (3) mutation level, and (4) mRNA level. In this study, a computational method is proposed to identify novel lung adenocarcinoma drivers based on dysfunctional genes on the methylation, microRNA, mutation and mRNA levels. First, a large network was constructed using protein-protein interactions. Next, we searched all of the shortest paths connecting dysfunctional genes on different levels and extracted new candidate genes lying on these paths. Finally, the obtained candidate genes were filtered by a permutation test and an additional strict selection procedure involving a betweenness ratio and an interaction score. Several candidate genes remained, which are deemed to be related to two different levels of cancer. The analyses confirmed our assertions that some have the potential to contribute to the tumorigenesis process on multiple levels.

Tumors are defined as new creatures formed by the abnormal proliferation of somatic cells with disordered growth regulation under the influence of tumorigenic factors^1^. Around the world, tumors have been reported to be the second killer of human health, ranked only behind cardiovascular disease. However, it is still not clear how tumor tissues initiate and invade during the precancerous lesion stage^2^. Specific genetic alterations have been detected in tumor cells of different types. Some well-known genes, such as p53, K-Ras, *etc.*, have been reported in various tumor types, which have been regarded as genomic markers for the given tumors and may be the original mutation related to tumor initiation and progression^3^,^4^.

In 2012, the theory of “cancer drivers” was first presented at the RAOF (Round Asia Oncology Forum), which connects tumor initiation with several specific mutations in the so-called cancer driver genes^5^. Such theory attributes tumor initiation to several original specific genomic alterations, which sequentially induce metabolic and functional disorders in somatic cells. As we know, based on the differentiation of four basic levels between the tumor and adjacent normal tissues, we can divide cancer driver genes into four clusters: (1) Methylated CpG site genes, (2) microRNA target genes, (3) somatic mutation genes, and (4) mRNA genes.

First, the level of methylation of driver genes may change during tumor initiation. Generally, the methylation and demethylation of peculiar regions in chromosomes reflects the regulation of gene expression on the transcriptional level^6^. The demethylation of oncogenes and/or the methylation of tumor suppressors may induce the proliferation and genomic instability of tumor cells^7^. Such processes may be the driving procedures of tumor initiation and progression. In addition to methylation and demethylation, another level cancer driver genes contribute to is associated with microRNA expression^8^. microRNA is a small non-coding RNA molecule that contributes to post-transcriptional regulation by RNA silencing^9^. During tumor initiation and progression, the specific microRNA expression level changes and may further regulate its functional target genes^10^. Such microRNA target genes are regulated differently in the tumor versus normal tissues. Thus, the level of functional microRNAs may also reveal several cancer driver genes. Genomic instability is another basic characteristic of tumor cells. As a result of that, mutations exist extensively in malignant cells. Therefore, the third level for cancer driver genes to initiate tumors is mutations. Some cancer driver genes, such as p53 and K-Ras, include specific mutations which contribute to the structural and functional changes of their protein products and may further induce tumor initiation and progression^11^. Mutations not only occur in the exons of driver genes but in their regulatory sequences as well, such as promoters, enhancers, *etc.*^12^,^13^. These mutations may not alter the structures or functions of target proteins which are encoded by driver genes but may strongly improve or reduce the quantity of mRNAs and proteins^14^. Apart from that, some unique abnormal regulatory factors with mutations may also contribute to the regulation of cancer genes at the transcription and/or translation levels^15^.

Lung adenocarcinoma is a typical subtype of lung cancer that is highly related to specific genetic background^16^. As a crucial threat to human health, however, most studies on lung adenocarcinoma drivers are restricted to a single level (one of the four clusters as we have mentioned above) and the core driver factors that contribute to the initiation and progression of lung adenocarcinoma have not been fully revealed. In these studies, most progressions of lung adenocarcinoma associated drivers mainly concentrate to the mutations and copy number variations (CNVs) of particular oncogenes^17^,^18^. For example, it has been widely confirmed by *in vitro* and *in vivo* experiments that specific mutations of EGFR and KRAS may exactly drive the initiation and progression of lung adenocarcinoma, implying the key role of certain drivers for the tumorigenesis of lung adenocarcinoma^19^,^20^. Apart from that, on another level, a specific microRNA associated mutation (target site on KRT81) has also been reported to be associated with lung adenocarcinoma^21^. As stated above, the original specific genomic alterations may contribute to tumor initiation in multiple levels, implying the analysis of specific variant should be extended to multi-omics. However, few multi-level analysis (such as the combination of mutations and copy number variants) of lung adenocarcinoma drivers have been presented and reported. For the first time, based on TCGA database, our study concentrate on all the four levels of drivers as we have mentioned above and try to fill the gap of this research field.

In this study, we investigated the specific driver factors of lung adenocarcinoma on four functional levels based on the gene expression, microRNA expression, DNA methylation and somatic mutation data of lung adenocarcinoma tissues and normal control samples from TCGA (The Cancer Genome Atlas)^22^. We first sought to search all the shortest paths (SP) connecting dysfunctional genes on different levels in a large network constructed by protein-protein interactions (PPI) and to identify new candidate cancer driver genes on these paths. Then, these genes were filtered by a permutation test and a strict selection procedure. The final obtained candidate genes were deemed to be related to two different levels, *i.e.*, they can drive tumorigenesis on two levels. Furthermore, some candidate genes may occur more than once, meaning that they can drive tumorigenesis on multiple levels. The more levels a gene can drive tumor initiation, the more significant the gene may be.

## Materials and Methods

### Dataset

We downloaded the gene expression, microRNA expression, DNA methylation and somatic mutation data of lung adenocarcinoma tissues and normal control samples from TCGA (https://tcga-data.nci.nih.gov/docs/publications/luad_2014/)^22^.

The expression level of 20,531 genes in 230 tumor samples and 43 normal samples was measured with RNA-Seq and transformed into the log2 scale. The expression level of 1,046 microRNAs in 181 tumor samples and 32 normal samples was measured with microRNA-Seq and also transformed onto the log2 scale. The DNA methylation level of 485,577 CpG sites in 181 tumor samples and 21 normal samples was measured using the Infinium HumanMethylation450 BeadChip array. The somatic mutation data of 14,989 genes in 230 tumor samples were called with MuTect^23^.

### Identification of the differentially expressed mRNA genes, microRNAs, methylated CpG sites and somatic mutation genes

We used the SAM (Significance Analysis of Microarrays) method^24^ to detect differentially expressed genes, microRNAs, and the methylated CpG sites. There were 1,373 differentially expressed mRNA genes, 42 microRNAs and 295 methylated CpG sites with a SAM FDR (False Discovery Rate) less than 0.01 and a fold change greater than 5. The 42 microRNAs regulated 825 target genes based on at least three out of six microRNA-target databases, including miRBase^25^ (http://microrna.sanger.ac.uk/targets/v5/), TargetScan^26^ (http://www.targetscan.org/), miRanda^27^ (http://www.microrna.org/microrna/), TarBase^28^ (http://diana.cslab.ece.ntua.gr/tarbase/), mirTarget2^29^ (http://mirdb.org/miRDB/download.html), and PicTar^30^ (http://pictar.mdc-berlin.de/). The 295 differentially expressed methylated CpG sites were annotated to 153 genes based on their chromosome locations from TCGA. The 197 somatic mutation genes with mutation frequencies greater than 10% were selected.

As a result, 153 methylated CpG site genes, 825 microRNA target genes, 197 somatic mutation genes, and 1,373 differentially expressed mRNA genes were considered seed genes and comprised the gene sets *G*_1,_ *G*_2,_ *G*_3_ and *G*_4_, respectively. The symbols of these genes are provided in the Supplementary Material I.

### Network construction

The constructed network was built according to the PPI information retrieved from STRING (Search Tool for the Retrieval of Interacting Genes/Proteins, http://www.string-db.org/, Version 9.1)^31^,^32^. The obtained file, ‘protein.links.v9.1.txt.gz’, contained large numbers of PPIs involving 1,133 organisms. In total, 2,425,314 human PPIs were extracted from this file by selecting lines starting with ‘9606.’. PPIs reported in STRING are obtained by the following sources: (1) genomic context, (2) high-throughput experiments, (3) (conserved) co-expression, and (4) previous knowledge. Thus, they can widely measure the associations between proteins and have been used to deal with several protein related problems^33^,^34^,^35^,^36^,^37^,^38^,^39^,^40^. For each of the 2,425,314 human PPIs, there are two proteins represented by Ensembl IDs and a score, indicating the strength of the interaction ranging between 150 and 999. Proteins in an interaction with a high score have strong associations. The constructed network took all the proteins occurring in 2,425,314 human PPIs, totaling 20,770 proteins, as nodes, and two nodes were connected by an edge if and only if the corresponding proteins could comprise an interaction. Clearly, each edge represented a human PPI. To indicate the score of each interaction, each edge should be assigned a weight. Because the range of the interaction score is between 150 and 999, *i.e.*, the maximum value of interaction score is 999, and a model using the shortest path algorithm as a basic algorithm requires edges assigned low weights indicate strong associations between corresponding nodes, the weight of each edge in the constructed network was defined as 1,000 minus the interaction score of the corresponding interaction.

### SP method for searching new candidate cancer drivers

It has been reported in some studies that two proteins in a PPI may share similar functions^33^,^35^,^38^,^41^,^42^,^43^. By further considering the interaction score, two proteins in a PPI with a high score are more likely to share similar functions. This can be further induced by the fact that if *p*_1_, *p*_2_,…,*p*_s_ is a series of proteins such that *p*_*i*_ and *p*_i+1_ can comprise a PPI with a high score (the corresponding edge in the network was assigned a low weight), and *p*_1_ and *p*_*s*_ are two proteins encoded by dysfunctional genes, then *p*_2_,…,*p*_s−1_ may also be encoded by dysfunctional genes. By mapping *p*_1_, *p*_2_,…,*p*_s_ to the network constructed in Section “Network construction”, the corresponding nodes can comprise the shortest path by the construction of the network. Because dysfunctional genes can be divided into four levels, comprising of four sets of seed genes denoted by *G*_1,_ *G*_2,_ *G*_3_ and *G*_4_, we searched candidate genes by pairing *G*_i_ and *G*_*j*_ (*i*  ≠ *j*) to identify new genes that can drive tumorigenesis on two levels. Thus, for any *G*_i_ and *G*_*j*_ (*i * ≠ *j*), we searched all of the shortest paths connecting genes in *G*_i_ and *G*_*j*_ by Dijkstra’s algorithm^44^. Accordingly, we obtained six sets of the shortest paths. For each set, we extracted genes that occurred in at least one path as the candidate genes. Furthermore to distinguish them, a measurement, namely betweenness^45^, was conducted for each candidate gene, which is defined as the number of paths containing the gene. Betweenness is a measure of centrality of a vertex within a graph which counts the number of times a node acts as a bridge in the shortest path between two other nodes, which in this study can be used to judge whether the candidate genes can drive tumor initiation on two levels^46^. For convenience, the set consisting of candidate genes for *G*_*i*_ and *G*_*j*_ is denoted by 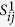.

### Permutation test

For *G*_*i*_ and *G*_*j*_ (*i *≠ *j*), we can obtain a set of candidate genes, making up the gene set 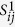, by the method described in Section “SP method for searching new candidate cancer drivers”. However, not all of them have the potential to become novel driver genes. False positives are inevitable. Among them, some are produced by the construction of the network. Taking this into consideration, we randomly produced two groups of gene sets; each group contained 1,000 gene sets, denoted by 
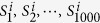
 and 
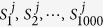
. 
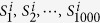
 is the same size as *G*_*i*_ , while 
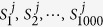
 is the same size as *G*_*j*_ . For 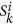 and 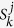 (*k* = 1,2, …, 1000), all the shortest paths connecting one gene in 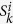 and one gene in 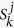 were searched in the constructed network. We counted the betweenness of the candidate genes in 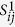 based on these paths. Thus, each candidate gene had one betweenness on *G*_*i*_ and *G*_*j*_ and 1,000 betweenness on 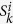 and 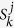 (*k* = 1,2, …,1000). Another measurement, namely permutation FDR, was computed for each candidate gene, which is defined as the ratio of the 1,000 betweenness on 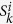
and 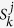 (*k* = 1,2, …,1000) that are larger than the betweenness on *G*_*i*_ and *G*_*j*_. It can be seen that a candidate gene with a high permutation FDR is more likely to be a false positive produced by the construction of the network and should be excluded. Thus, candidate genes with a permutation FDR larger than or equal to 0.05 were discarded. The remaining candidate genes, making up the gene set 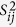, were further evaluated by the method in the following section.

### Further selection using betweenness and PPI

For *G*_*i*_ and *G*_*j*_ (*i *≠ *j*), some candidate genes remained after executing the permutation test. However, some of them may have strong associations with cancer, while others have weak associations. To reflect this fact, we proposed some rules in this section and selected the important candidate genes based on these rules.

It has been elaborated that the betweenness of a candidate gene is the number of paths connecting genes in *G*_*i*_ and in *G*_*j*_ including the candidate gene. Clearly, a candidate gene with high betweenness suggests it has strong associations with genes in *G*_*i*_ and *G*_*j*_, thereby having a high likelihood of being a novel cancer driver gene. To build a uniform rule, we must also consider the sizes of *G*_*i*_ and *G*_*j*_ because a candidate gene with a small betweenness for small *G*_*i*_ and *G*_*j*_ is not always less important than another candidate gene with a large betweenness for large *G*_*i*_ and *G*_*j*_. Thus, we set a betweenness ratio *R*(*g*) to measure the importance of a candidate gene *g* based on its betweenness and sizes of *G*_*i*_ and *G*_*j*_, which is defined as


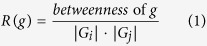


It can be seen that a high betweenness ratio of a candidate gene means that the majority of the shortest paths connecting genes in *G*_*i*_ and in *G*_*j*_ contains the candidate gene. We set a threshold of 0.01 for the betweenness ratio to select important candidate genes.

Another rule was built based on the PPIs and their interaction scores. It has been reported that two proteins in a PPI with a high score are more likely to share similar functions^33^,^47^,^48^. Thus, for a candidate gene *g*, if *g* and genes in *G*_*i*_ and *G*_*j*_ can comprise PPIs with high interaction scores, *g* has strong associations with genes in *G*_*i*_ and *G*_*j*_. Thus, we computed the following value, namely the min-max interaction score:


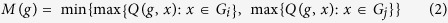


Similarly, a candidate gene with a high min-max interaction score implies that it has strong associations with at least one gene in *G*_*i*_ and at least one gene in *G*_*j*_, indicating that it has a high linkage with cancer. Similar to the proportion mentioned in the paragraph above, we can also set a threshold of 400 for the min-max interaction score to select important candidate genes.

For the gene sets *G*_*i*_ and *G*_*j*_, genes in 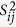 were filtered by the above two rules. The remaining genes constituted the set 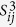, which were deemed to be significant for two levels.

## Results

**Figure 1:**
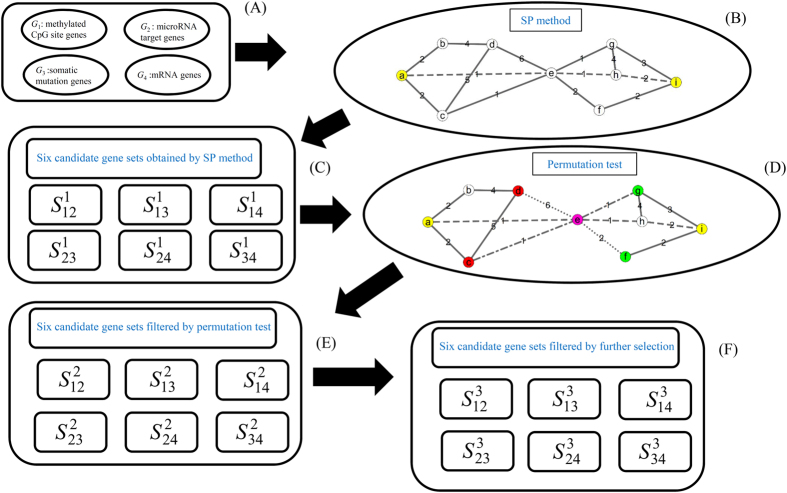
Flowchart of our method. (**A**) Four gene sets consisting of dysfunctional genes on four levels; (**B**) SP method to search candidates in a network. Yellow nodes represent dysfunctional genes on different levels, and the dashed lines represent the shortest path connecting a and i, e and h are selected; (**C**) Six candidate gene sets obtained by the SP method; (**D**) Permutation test to filter some false positives. Two randomly produced sets {d, f} and {c, g} were shown in the network (highlighted in red and green), in detail, red nodes d and c replace yellow node a, while green nodes g and f replace yellow node i, dotted lines represent the shortest path connecting d and f, dashed-dotted lines represent the shortest path connecting c and g, and e (highlighted in pink) is removed by the permutation test; (**E**) Six candidate gene sets filtered by the permutation test; (**F**) Six candidate gene sets filtered by further selection using betweenness and PPI.

In this study, we proposed a computational method to identify candidate cancer driver genes that can drive tumor initiation on multiple levels. The flowchart of our method is shown in Fig. 1. The results of our method are described in the following sections.

### Results of the SP method

As mentioned in Section “Identification of the differentially expressed mRNA genes, microRNAs, methylated CpG sites and somatic mutation genes”, we employed four gene sets *G*_1,_ *G*_2_, *G*_3_ and *G*_4_ with different expression levels for cancer. For each pair, *e.g.*, *G*_*i*_ and *G*_*j*_ (*i *≠ *j*), we searched all the shortest paths connecting any gene in *G*_*i*_ with any gene in *G*_*j*_ in a network constructed in Section “Network construction” and extracted genes on these paths. The obtained six sets of candidate genes, 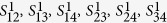, are provided in the Supplementary Material II. The numbers of genes in these sets are listed in column 2 of Table 1. It can be seen that many candidate genes were included in each set, meaning that further filtering was necessary. Furthermore, the betweenness of each candidate gene in 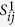 was calculated and also provided in the Supplementary Material II.

### Results of the permutation test

To control for the false positives produced by the construction of the network in each candidate set 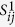, a permutation test was adopted. A permutation FDR was calculated for each candidate gene in 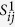, which is also listed in the Supplementary Material II. By setting a threshold of 0.05 for the permutation FDR, we extracted a candidate gene subset 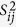 from 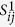. The detailed genes in the six gene sets 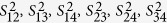 are provided in the Supplementary Material III, and the numbers of genes in these sets are listed in column 3 of Table 1, from which we can see that the number of candidate genes decreased significantly and became close to the reality.

### Results of further selection

To select the core genes in 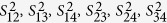, we calculated the betweenness ratio (cf. Equation 1) and the min-max interaction score (cf. Equation 2) for each candidate gene using a threshold of 0.01 for the betweenness ratio and a threshold of 400 for the min-max interaction score. These two measurements of each candidate gene are provided in the Supplementary Material III, and the remaining genes are listed in the Supplementary Material IV. The number of genes in the gene sets 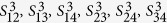 is listed in column 4 of Table 1. Compared to the number of candidate genes listed in column 3 of Table 1, the candidate genes were again decreased significantly. It is believed that these candidate genes have few false positives and have strong associations with cancer. Some of them are discussed in the following section.

## Discussion

### Analysis of candidate genes of two levels

Cancer driver genes as we have mentioned above have been widely reported to be the driving force for the tumorigenesis. According to our method, we obtained various genes that contribute to the initiation and progression of lung adenocarcinoma on at least two levels of the four basic driver levels (methylation, mutations, microRNA expression and expression/mRNA). The important candidates involving any two driver levels are listed in Tables 2, 3, 4, 5, 6, 7. Here, we only provide the brief analyses, the detailed analyses are provided in Supplementary Material V.

For the levels of methylation diversity and microRNA expression abundance, 27 genes were predicted to driver the lung adenocarcinoma in such two levels. Among them, six of them have the evidences to support the claim and are listed in Table 2. Interacting with specific microRNAs such as microRNA-142-3p, functional genes like TCF3, MEN1, MLL, EFNA4, PBX1, and SHH have all been confirmed to contribute to tumorigenesis via methylation alteration and microRNA regulation (The detailed analysis of the important candidates can be seen in Supplementary Material V). Take TCF3 as an example. The methylation alteration of TCF3 has been confirmed to contribute to the proliferation of A549 cells, a typical lung adenocarcinoma cell line *in vitro* experiments, implying that such gene may also contribute to the initiation and progression of lung adenocarcinoma on such level^49^. As for the microRNA level, the interactions between TCF3 and a group of microRNAs (miR-590, miR-17 and miR-18) has been confirmed, validating that TCF3 may also contribute to the initiation and progression of lung adenocarcinoma on such level^50^. For the methylation diversity and mutation differentiation, there are still several genes (such as TCF3, MLL, MEN1, SHH, CTNNB1 and FZD1, listed in Table 3) that have been reported to participate in the tumor associated pathways (The detailed analysis of the important candidates can be seen in Supplementary Material V). The methylation and mutation status of such genes have been confirmed to be abnormal during the progression of lung adenocarcinoma and similar solid tumors. Take FZD1 as an example, FZD1 is a functional gene on our list, which has been widely reported to be related to various tumor subtypes^51^,^52^. The methylation status of this gene has been associated with prostate cancer and age-associated diseases^53^,^54^. There are only a few reports of FZD1-associated mutations. However, it has been proven that the mutations of FZD-1 may be crucial for specific diseases including tumors, validating our prediction^55^,^56^. Considering that the gene expression is regulated by specific methylation process in the genome, this level, corresponding to the mRNA level diversity between the tumor tissue and the adjacent normal tissue, is associated with the first level (methylation diversity). Genes like MEN1, TCF3 and SHH, listed in Table 4, are all crucial genes that contribute to the initiation and progression of tumors on multiple levels (The detailed analysis of the important candidates can be seen in Supplementary Material V). TCF3 as we have mentioned above turns out to be confirmed to contribute to lung adenocarcinoma on methylation alteration^49^. What’s more, considering the expression regulation function of microRNAs, such identified microRNA-associated cancer driver may also contribute to tumor genesis on mRNA level. Some of the candidate genes have also been predicted to be related to both microRNA expression differentiation and mutation diversity of malignant and somatic cells. Interacting with microRNA-365 and microRNA-27b, the most important candidates for these two levels are listed in Table 5. COL1A2, SHC1, FKBP1A, TTN and NGF are all crucial cancer driver genes that contribute to lung adenocarcinoma in their respective ways (The detailed analysis of the important candidates can be seen in Supplementary Material V). The fifth group of genes (including PTCH1, SHH, ITGA2, ITGA5, GRB2, EP300 and SMC3, listed in Table 6) contribute to the progression of lung adenocarcinoma on at least the microRNA regulation and mRNA expression level (The detailed analysis of the important candidates can be seen in Supplementary Material V). As for the last set of genes, such genes ITGA2, ITGA5, NOTCH1, PXN and DYNLL1, listed in Table 7, contributes to tumors on both the mutation and mRNA levels (The detailed analysis of the important candidates can be seen in Supplementary Material V). All of our predicted genes that contribute to at least two levels have been confirmed to be real driver genes by recent publications

### Analysis of candidate genes of high frequencies

In Section “Results”, six sets of candidate genes were obtained that were deemed to induce tumor initiation and progression on two levels. We took the union operation of these six sets and obtained 110 candidate genes. Among them, some genes occurred many times, meaning that they may drive tumor initiation and progression on multiple levels. Thus, the frequencies of 110 candidate genes were counted and listed in the Supplementary Material VI. Because there were totally six sets of candidate genes, six is the maximum value of frequencies for each candidate gene. This section gives a detailed discussion of the genes with frequencies greater than three (half of the maximum frequency), which are listed in Table 8. These candidate genes occurred in more than half of the candidate gene sets and have been reported and confirmed to participate in and contribute to the process of tumorigenesis. Here, their brief discussion is provided. Readers can found the detailed analyses in Supplementary Material VII.

Genes occurred in more than three gene sets have been analyzed. Three genes, PTCH1, CTNNB1, and FYN, have been predicted to contribute to the initiation and progression of lung adenocarcinoma in all the six gene sets. Such three genes have all been regarded as core functional cancer driver genes. Associated with proliferation and adhesion associated pathways such a PI-3K cascade, such three genes not only participate in the initiation and proliferation of the tumor , but regulate the metastasis processes as well^57^ (The detailed analysis of such genes can be seen in the Supplementary Material VII). As for the five genes (BCAR1, SHH, NGF, VEGFA, and GCG) that can be identified to be shared in five gene sets, they are also confirmed to be significant driver genes. Take BCAR1 as an example, such gene involves in crucial regulatory pathways like tyrosine kinase signaling pathways and further contribute to the survival, proliferation and invasion processes during tumorigenesis^58^ (The detailed analysis of such genes can be seen in the Supplementary Material VII). Quite more genes have been clustered in the group with the regulatory level frequency of four. Such genes regulate the abnormal pathways during the tumorigenesis processes such as the cell-cell adhesion regulation (CDH1), proliferation (STAT3, SRC), and chronic inflammatory reaction (LEP). All of such genes can be confirmed to be cancer driver genes by recent publications (The detailed analysis of such genes can be seen in the Supplementary Material VII).

As we have mentioned above, we identified a group of candidate cancer drivers that contribute to lung adenocarcinoma in multiple levels, which are all proved by recent literatures. Here, we may propose a new hypothesis for the initiation and progression of lung adenocarcinoma: the real core driver of lung adenocarcinoma (and maybe other cancers) may contribute to tumor genesis simultaneously on multiple levels. Considering the complicated regulatory system of human bodies, a single abnormal variation that contribute to the genomic alterations of a single level (*e.g.* mutations) may not be functional and significant enough to initiate the tumor genesis. The real core driver of cancer (lung adenocarcinoma) may contribute to tumor genesis on at least two levels to insure the initiation of malignant changes of normal cells. For example, the well-known typical core drivers for lung adenocarcinoma like EGFR all contribute to lung adenocarcinoma on multiple levels, though haven’t been identified and analyzed in the same publications^19^,^59^. All in all, based on our newly presented computational methods, we not only identified a group of novel cancer drivers for lung adenocarcinoma, but presented a new perspective for the underlying mechanisms of tumor genesis, providing a new sight into the initiation and progression of lung adenocarcinoma.

## Conclusions

This contribution investigated the so-called cancer driver genes. A computational method was built to identify new potential candidate cancer driver genes. The analyses indicate that some of the obtained genes have the potential to drive tumorigenesis on multiple differentiation levels. It is hopeful that the findings presented in this study will promote the study of cancer driver genes and provide new insights into the investigation of tumor initiation. In this study, we used the protein information (protein-protein interaction) to investigate cancer driver genes. In future, we will consider adding some other information, such as microRNA related to cancer^60^, into our method, which may yield more useful information for the study of cancer driver gene.

## Additional Information

**How to cite this article**: Chen, L. *et al.* Identification of novel candidate drivers connecting different dysfunctional levels for lung adenocarcinoma using protein-protein interactions and a shortest path approach. *Sci. Rep.* **6**, 29849; doi: 10.1038/srep29849 (2016).

## Supplementary Material

Supplementary Information

## Figures and Tables

**Table 1:** Number of candidate genes obtained by the SP method and filtered by the permutation test and further selection.

Pair of gene sets	Number of candidate genes obtained by SP method ( 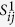 )	Number of candidate genes filtered by permutation test ( 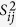 )	Number of candidate genes filtered by further selection using betweenness and PPI ( 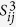 )
*G*_1_ and *G*_2_	1355	310	27
*G*_1_ and *G*_3_	723	242	42
*G*_1_ and *G*_4_	1606	455	39
*G*_2_ and *G*_3_	1402	357	45
*G*_2_ and *G*_4_	2515	485	33
*G*_3_ and *G*_4_	1705	431	56

*G*_1_: A set containing 153 methylated CpG site genes; *G*_2_: A set containing 825 microRNA target genes; *G*_3_: A set containing 197 somatic mutation genes; *G*_4_: A set containing 1,373 mRNA genes.

**Table 2:** Important candidate genes in 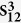 (based on methylated CpG site genes and microRNA target genes) identified by our method.

Ensembl ID	Gene symbol	Description	Betweenness	Permutation FDR	Betweenness ratio	Min-Max interaction score
ENSP00000403005	EFNA4	Ephrin-A4	1597	<0.001	0.014897	679
ENSP00000337088	MEN1	Multiple Endocrine Neoplasia I	7269	<0.001	0.067808	719
ENSP00000352262	MLL	Myeloid/lymphoid or mixed-lineage leukemia (trithorax homolog, Drosophila)	7342	<0.001	0.068489	988
ENSP00000405890	PBX1	Pre-B-Cell Leukemia Homeobox 1	2529	<0.001	0.023591	822
ENSP00000297261	SHH	Sonic Hedgehog	2800	0.001	0.026119	986
ENSP00000262965	TCF3	Transcription Factor 3	2651	<0.001	0.024729	985

**Table 3:** Important candidate genes in 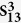 (based on methylated CpG site genes and somatic mutation genes) identified by our method.

Ensembl ID	Gene symbol	Description	Betweenness	Permutation FDR	Betweenness ratio	Min-Max interaction score
ENSP00000344456	CTNNB1	Catenin (Cadherin-Associated Protein), Beta 1, 88kDa	3240	<0.001	0.126592	996
ENSP00000287934	FZD1	Frizzled Class Receptor 1	382	0.002	0.014925	813
ENSP00000337088	MEN1	Multiple Endocrine Neoplasia I	1567	<0.001	0.061225	719
ENSP00000352262	MLL	Myeloid/lymphoid or mixed-lineage leukemia (trithorax homolog, Drosophila)	1581	<0.001	0.061772	571
ENSP00000297261	SHH	Sonic Hedgehog	706	<0.001	0.027585	985
ENSP00000262965	TCF3	Transcription Factor 3	571	0.002	0.02231	420

**Table 4:** Important candidate genes in 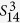 (based on methylated CpG site genes and mRNA genes) identified by our method.

Ensembl ID	Gene symbol	Description	Betweenness	Permutation FDR	Betweenness ratio	Min-Max interaction score
ENSP00000337088	MEN1	Multiple Endocrine Neoplasia I	9748	<0.001	0.059972	719
ENSP00000297261	SHH	Sonic Hedgehog	4782	<0.001	0.02942	995
ENSP00000262965	TCF3	Transcription Factor 3	4274	<0.001	0.026295	987

**Table 5:** Important candidate genes in 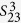 (based on microRNA target genes and somatic mutation genes) identified by our method.

Ensembl ID	Gene symbol	Description	Betweenness	Permutation FDR	Betweenness ratio	Min-Max interaction score
ENSP00000297268	COL1A2	Collagen, Type I, Alpha 2	2577	<0.001	0.016865	985
ENSP00000371138	FKBP1A	FK506 Binding Protein 1A, 12kDa	3140	<0.001	0.02055	998
ENSP00000358525	NGF	Nerve Growth Factor (Beta Polypeptide)	4923	<0.001	0.032219	943
ENSP00000401303	SHC1	SHC (Src Homology 2 Domain Containing) Transforming Protein 1	6291	<0.001	0.041171	999
ENSP00000348444	TTN	Titin	3248	<0.001	0.021257	504

**Table 6:** Important candidate genes in 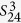 (based on microRNA target genes and mRNA genes) identified by our method.

Ensembl ID	Gene symbol	Description	Betweenness	Permutation FDR	Betweenness ratio	Min-Max interaction score
ENSP00000263253	EP300	E1A Binding Protein P300	60273	<0.001	0.062112	995
ENSP00000339007	GRB2	Growth Factor Receptor-Bound Protein 2	48282	<0.001	0.049755	939
ENSP00000296585	ITGA2	Integrin, Alpha 2 (CD49B, Alpha 2 Subunit Of VLA-2 Receptor)	12251	0.002	0.012625	987
ENSP00000293379	ITGA5	Integrin, Alpha 5 (Fibronectin Receptor, Alpha Polypeptide)	25513	0.004	0.026291	964
ENSP00000332353	PTCH1	Patched 1	17300	<0.001	0.017828	939
ENSP00000297261	SHH	Sonic Hedgehog	10778	<0.001	0.011107	986
ENSP00000354720	SMC3	Structural Maintenance Of Chromosomes 3	10413	0.001	0.010731	986

**Table 7:** Important candidate genes in 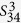 (based on somatic mutation genes and mRNA genes) identified by our method.

Ensembl ID	Gene symbol	Description	Betweenness	Permutation FDR	Betweenness ratio	Min-Max interaction score
ENSP00000242577	DYNLL1	Dynein, Light Chain, LC8-Type 1	6746	<0.001	0.029117	803
ENSP00000296585	ITGA2	Integrin, Alpha 2 (CD49B, Alpha 2 Subunit Of VLA-2 Receptor)	9109	<0.001	0.039317	959
ENSP00000293379	ITGA5	Integrin, Alpha 5 (Fibronectin Receptor, Alpha Polypeptide)	10741	<0.001	0.046361	835
ENSP00000277541	NOTCH1	Notch 1	11069	<0.001	0.047776	948
ENSP00000228307	PXN	Paxillin	3913	<0.001	0.016889	702

**Table 8:** Frequencies of some core candidate genes.

Ensembl ID	Gene symbol	Description	Frequency	Pair of gene sets producing the candidate gene
ENSP00000332353	PTCH1	Patched 1	6	*G*_1_ and *G*_2_, *G*_1_ and *G*_3_, *G*_1_ and *G*_4_, *G*_2_ and *G*_3_, *G*_2_ and *G*_4_, *G*_3_ and *G*_4_
ENSP00000344456	CTNNB1	Catenin (Cadherin-Associated Protein), Beta 1, 88 kDa	6	*G*_1_ and *G*_2_, *G*_1_ and *G*_3_, *G*_1_ and *G*_4_, *G*_2_ and *G*_3_, *G*_2_ and *G*_4_, *G*_3_ and *G*_4_
ENSP00000357656	FYN	FYN Proto-Oncogene, Src Family Tyrosine Kinase	6	*G*_1_ and *G*_2_, *G*_1_ and *G*_3_, *G*_1_ and *G*_4_, *G*_2_ and *G*_3_, *G*_2_ and *G*_4_, *G*_3_ and *G*_4_
ENSP00000162330	BCAR1	Breast Cancer Anti-Estrogen Resistance 1	5	*G*_1_ and *G*_3_, *G*_1_ and *G*_4_, *G*_2_ and *G*_3_, *G*_2_ and *G*_4_, *G*_3_ and *G*_4_
ENSP00000297261	SHH	Sonic Hedgehog	5	*G*_1_ and *G*_2_, *G*_1_ and *G*_3_, *G*_1_ and *G*_4_, *G*_2_ and *G*_4_, *G*_3_ and *G*_4_
ENSP00000358525	NGF	Nerve Growth Factor (Beta Polypeptide)	5	*G*_1_ and *G*_2_, *G*_1_ and *G*_3_, *G*_2_ and *G*_3_, *G*_2_ and *G*_4_, *G*_3_ and *G*_4_
ENSP00000361125	VEGFA	Vascular Endothelial Growth Factor A	5	*G*_1_ and *G*_3_, *G*_1_ and *G*_4_, *G*_2_ and *G*_3_, *G*_2_ and *G*_4_, *G*_3_ and *G*_4_
ENSP00000387662	GCG	Glucagon	5	*G*_1_ and *G*_2_, *G*_1_ and *G*_3_, *G*_1_ and *G*_4_, *G*_2_ and *G*_4_, *G*_3_ and *G*_4_
ENSP00000261769	CDH1	Cadherin 1, Type 1, E-Cadherin (Epithelial)	4	*G*_1_ and *G*_3_, *G*_1_ and *G*_4_, *G*_2_ and *G*_3_, *G*_3_ and *G*_4_
ENSP00000264657	STAT3	Signal Transducer And Activator Of Transcription 3 (Acute-Phase Response Factor)	4	*G*_1_ and *G*_2_, *G*_1_ and *G*_3_, *G*_1_ and *G*_4_, *G*_2_ and *G*_4_
ENSP00000277541	NOTCH1	Notch 1	4	*G*_1_ and *G*_3_, *G*_2_ and *G*_3_, *G*_2_ and *G*_4_, *G*_3_ and *G*_4_
ENSP00000296585	ITGA2	Integrin, Alpha 2 (CD49B, Alpha 2 Subunit Of VLA-2 Receptor)	4	*G*_1_ and *G*_3_, *G*_2_ and *G*_3_, *G*_2_ and *G*_4_, *G*_3_ and *G*_4_
ENSP00000312652	LEP	Leptin	4	*G*_1_ and *G*_2_, *G*_1_ and *G*_3_, *G*_1_ and *G*_4_, *G*_2_ and *G*_4_
ENSP00000350941	SRC	SRC Proto-Oncogene, Non-Receptor Tyrosine Kinase	4	*G*_1_ and *G*_3_, *G*_2_ and *G*_3_, *G*_2_ and *G*_4_, *G*_3_ and *G*_4_

*G*_1_: A set containing 153 methylated CpG site genes; *G*_2_: A set containing 825 microRNA target genes; *G*_3_: A set containing 197 somatic mutation genes; *G*_4_: A set containing 1,373 mRNA genes.
