# Supplementary material for: Identification of novel candidate drivers connecting different dysfunctional levels for lung adenocarcinoma using protein-protein interactions and a shortest path approach
Source: Sci Rep. 2016 Jul 14;6:29849. doi: 10.1038/srep29849 (PMC4944139; doi:10.1038/srep29849)
Supplement: Supplementary Information [file srep29849-s1.pdf]

Identification of novel candidate drivers connecting different dysfunctional levels for lung adenocarcinoma using protein-protein interactions and a shortest path approach

Lei Chen, Tao Huang, Yu-Hang Zhang, Yang Jiang, Mingyue Zheng, Yu-Dong Cai

Supplementary Material I. Dysfunctional genes on four different levels

1. **153 methylation CpG site genes**

SCT	SST	T	TRH	ACTL6B
AJAP1	ALX3	ALX4	APCDD1L	ASCL4
BARHL2	C1orf94	CCDC105	CDO1	CLEC14A
CMTM2	CNGA3	COL23A1	CORO6	CRMP1
CRYGD	CSDAP1	CXCL16	CYP26A1	DARS
DBX1	DDX25	DLX4	DMRTA2	DRD2
DRD5	EMID2	EMX2	EMX2OS	EPHA10
FAIM2	FAM19A2	FAM38B	FERD3L	FGF8
FSCN1	GABRA4	GALR1	GAS1	GATA2
GATA3	GDF6	GRM1	GRM6	HAND2
HLA-G	HMX3	HOXA10	HOXA11	HOXA9
HOXB4	HOXD10	HOXD12	HOXD4	HOXD8
HOXD9	HPSE2	KCNA6	KCNC2	KIF15
LBXCOR1	LHX1	LHX4	LRRN2	MACROD1
11-Mar	MEIS1	MIR10B	MRC2	MYOD1
NDRG4	NEFH	NEFM	NEUROD1	NID2
NKX2-6	NOL4	NPAS4	NR2E1	NRN1
NRXN1	OLIG2	ONECUT2	OTX1	OTX2OS1
PAX5	PAX6	PCDH8	PDX1	PHOX2B
PITX1	PITX2	PLEC1	POU4F2	PRAC
PRDM14	PRKAB1	PUS3	QRFPR	RAB6C
ROBO1	SFRP2	SH3BP1	SIM1	SIM2
SIX3	SIX6	SLC24A4	SLC2A14	SLC32A1
SLC7A14	SOX1	SRCIN1	ST8SIA3	TAC1
TBX20	TCF15	TFAP2B	TRABD	TRIM58
TSPAN9	TULP1	UNCX	VAX1	WDR8
WNT3	ZIC4	ZMYND15	ZNF177	ZNF382
ZNF385A	ZNF529	ZNF536	ZNF761	ZNF781
ADAMTS20	C12orf42	C14orf23	C17orf93	C20orf160
FLJ32063	HOXA11AS	KIAA1143	LOC147804	LOC150786
LOC645323	NBLA00301	TCTEX1D1		

2. **825 microRNA target genes**

CKB	CSK	CTH	DST	FAS
FGG	FN1	FRK	FUS	GEM
GHR	GK	ID2	IL7	JUN

LUM	MAK	MET	MRO	NCL
NF1	NIN	NTM	OAT	PAM
PC	PSD	PTH	SF1	SI
SON	SP3	TEK	TSN	TTK
TTN	ZFX	ABCA12	ABHD3	ABI1
ABI2	ABLIM1	ACAD11	ACBD3	ACBD5
ACER3	ACOT4	ACSL4	ACTR3	ADAMTS6
ADCYAP1	ADHFE1	AFF4	AIFM3	ALDH1A1
ALS2	ALX1	ANKRD29	ANO1	ANXA4
ANXA7	AP3M1	APBB2	APLF	AQP9
ARF4	ARHGAP6	ARID1A	ARL4D	ARL6IP1
ARMCX2	ARPC3	ARPP21	ASH2L	ASPA
ASZ1	ATE1	ATF2	ATP1B3	ATP2C1
B4GALT4	BAG1	BAI3	BAMBI	BANF1
BBS12	BCL2L12	BDNF	BEX5	BHLHE22
BRPF1	BTBD10	BTBD3	BTG1	BZW2
C2orf15	C5orf15	C5orf22	C8orf4	C9orf72
CAB39	CAMTA1	CAP2	CAPN15	CAPN6
CAPRIN2	CASD1	CASKIN1	CBX8	CCDC132
CCDC181	CCDC82	CCDC91	CCL1	CCL20
CCNC	CCNE2	CCNJL	CCNT2	CD34
CD68	CDC73	CDH2	CDH20	CDK13
CDKL2	CDKN2B	CDKN2C	CEBPZ	CELF1
CEP112	CEP55	CEP63	CEP83	CETN1
CFAP20	CFL2	CHAMP1	CHCHD1	CHD5
CHFR	CHGA	CHIC2	CHMP4A	CHMP4B
CHSY1	CHTOP	CLCN3	CLEC4A	CLTA
CLTC	CLUL1	CNN3	CNOT11	CNOT8
COL6A3	COLEC12	COMMD2	COPS2	COX8C
CPEB1	CPSF6	CREB3L1	CREM	CRISP3
CRYZL1	CSDE1	CSN2	CSPG5	CTBP1
CTSC	CTTNBP2	CXCL10	CYP4B1	DARS
DCUN1D3	DDHD1	DDX17	DDX5	DENND5B
DGKG	DHX15	DIAPH2	DLG2	DMXL1
DNA2	DNAJB11	DNAJC12	DOCK7	DOK4
DPY19L4	DTNBP1	DUSP10	DVL2	DYNLL2
DZIP1	E2F5	E2F8	EEF2	EFCAB5
EFEMP1	EFNA3	EFTUD2	EGLN1	EGR1
EIF2A	EIF2AK4	EIF3A	EIF4E	EIF4E3
EIF4G2	ELF2	ELK3	EML1	EMP2
ENAH	ENPP2	ENPP3	ENSA	EOGT
EPHA7	EPHB1	EPM2A	EPS8	EPYC
ERBB4	ESCO1	ETS1	ETV2	EVA1C
EWSR1	EXO1	EXOC4	FABP4	FAIM

FAM135A	FAM150A	FAM174A	FAM188A	FAM43A
FAM49B	FAM71F1	FANCA	FBN1	FBN2
FBXL3	FBXO32	FBXO4	FBXO9	FBXW2
FCHSD2	FCRLA	FGD6	FGF13	FGF9
FGL2	FLI1	FNIP1	FOXF2	FOXN3
FOXO3	FOXR2	FREM2	FRMD3	FRMD6
FSIP1	FSTL5	FUBP1	FXR1	GABPB1
GABPB2	GAD2	GADD45A	GALK2	GALNT12
GAP43	GAPT	GAR1	GAREM	GATA3
GCNT1	GCSAM	GDF10	GEMIN8	GFPT2
GGNBP2	GID4	GJA1	GLCCI1	GLDC
GLRA2	GLRA3	GLRB	GLUL	GON4L
GPC3	GPR124	GPR135	GPR34	GPX7
GRAMD3	GRHL2	GRIA1	GTF2B	GTF2H1
GTF3C6	H2AFV	HAS2	HAT1	HAX1
HBEGF	HBP1	HDHD2	HECTD2	HERC1
HIVEP2	HMGN5	HNRNPC	HNRNPF	HNRNPM
HNRNPU	HORMAD1	HOXA10	HOXA4	HOXA5
HOXB3	HOXB4	HPRT1	HPS3	HSDL1
HSF2	HSP90B1	HSPD1	ICA1	IDH2
IGF1R	IGIP	IL10	IL12A	IL15
IL15RA	IL16	IMPA1	ING3	IP6K2
IRS1	ISL1	ITGB1	ITIH6	ITPR2
JAG1	JAK2	JAKMIP1	JAKMIP2	JARID2
JMJD1C	KAT6B	KCNAB3	KCNJ6	KCNK10
KCNMA1	KCNN3	KCTD4	KCTD8	KDM1B
KDM2B	KDM4C	KDM7A	KHDRBS3	KIF2A
KIF7	KLHDC9	KLHL11	KLHL7	KMT2A
KRIT1	KRT12	KRT27	KRT76	KTN1
LACTB2	LHFP	LHX2	LIMCH1	LOXL4
LPHN2	LRIG3	LRP2	LRP6	LRRC16A
LRRC66	LRRC7	LRRC8A	LRRK1	LSM14A
LUC7L3	LYSMD2	LYVE1	MAB21L1	MANF
MAP2K1	MAP3K4	MAP3K8	MAP7D1	MAP9
MAPK10	2-Mar	MARK1	MASP1	MASTL
MAT2B	MDM1	MED1	MED12L	MED21
MED30	MED4	MELK	MEMO1	MFSD1
MGAT1	MGAT4A	MIER1	MON2	MORF4L1
MORF4L2	MPP6	MRAS	MRC2	MRPL9
MSANTD2	MSH4	MSX1	MTMR8	MTOR
MTRR	MUM1L1	MYH1	MYT1L	NBPF10
NBPF14	NCKIPSD	NCOR1	NDNF	NDUFA4
NEFL	NELL1	NEO1	NETO2	NFIB
NFKBIZ	NINL	NKX3-1	NLGN2	NME7

NMRK1	NPAT	NPM1	NPTN	NR2F2
NR3C2	NR5A2	NRIP3	NRN1	NRP2
NSL1	NTF3	NTN4	NTNG1	NUAK1
NULL	NXF2	NXT2	ODC1	OLFML1
ONECUT1	OR13A1	OSBPL10	OSR2	OTOGL
OTUD4	OXR1	P4HA1	PAIP2	PAN3
PAWR	PCBP1	PCDH11X	PCDH15	PCDH18
PCDH20	PCDH8	PCTP	PDCD10	PDCD4
PDE7A	PDHX	PDZD11	PDZD4	PELI1
PEX7	PFKM	PGGT1B	PHACTR2	PHF12
PHF20L1	PHKB	PHLDB2	PITX2	PKD2
PKHD1	PLAA	PLAGL1	PLCB4	PLCL2
PLEKHA3	PLXDC2	PNRC1	POLI	POLR3F
POMP	POSTN	PPIL6	PPP2CB	PREP
PRKCB	PRKRA	PRKRIR	PROK2	PRPF18
PRR15	PRRT3	PRSS54	PSAP	PSTPIP2
PTF1A	PTHLH	PTK2	PTPN2	PTPN4
PTPN9	PTPRD	RAB21	RAB34	RAB3IP
RAD9B	RALGPS1	RANBP9	RAPGEF4	RAPGEF6
RAPH1	RARB	RARG	RASA1	RBFOX1
RBFOX2	RCC1	RCN2	RECK	REV1
REXO2	RGS14	RGS17	RGS2	RGS7
RHBDL2	RHPN2	RIF1	RIT2	RNF13
RNF139	RNF145	RNF149	RNF6	RNMT
RPS6KA5	RRBP1	RSBN1	RSL24D1	RTCA
RTN4	S100A14	SACM1L	SACS	SAMD9L
SAP30L	SAT1	SCEL	SCML2	SCN11A
SDC2	SEC24D	SEC61B	SERBP1	SERTAD3
SESN1	SETX	SF3B1	SGK3	SGMS2
SGPP1	SH3BGRL	SH3KBP1	SHMT1	SHMT2
SIAH1	SIN3A	SIT1	SKP1	SLAIN1
SLC10A4	SLC12A6	SLC20A1	SLC25A4	SLC25A5
SLC30A7	SLC30A9	SLC31A2	SLC35A1	SLC35G1
SLC38A9	SLC45A3	SLC4A11	SLC50A1	SLC5A7
SLIT2	SLITRK2	SLITRK3	SLITRK4	SLMAP
SMAD1	SMAP1	SMAP2	SMARCA1	SMARCD1
SMIM14	SMIM8	SNAP25	SNIP1	SNX1
SNX2	SNX7	SOWAHB	SOX2	SOX30
SOX5	SPAG17	SPATS1	SPRY2	SPSB1
SQLE	SRA1	SRGN	SRSF10	SRSF11
SRSF9	SSR2	ST8SIA3	STAG1	STAG2
STIM2	STK17B	STK19	STK33	STXBP3
STYK1	SUCLA2	SUCLG2	SULF1	SUMO1
SUSD1	SUV39H2	SYBU	SYDE1	SYT6

TAB3	TACC1	TACC2	TAF15	TAF1B
TAGLN2	TBK1	TC2N	TCERG1	TCF7L2
TCFL5	TESK2	TET3	TEX2	TFAP2A
TFAP2D	TFAP2E	TFAP4	TGFBI	TJP1
TM9SF1	TMED10	TMEFF2	TMEM145	TMEM2
TMEM243	TMEM245	TMEM27	TMEM50B	TMEM52B
TMEM65	TMF1	TMSB4X	TNFSF11	TOMM70A
TRAPPC3	TRAPPC8	TRIM10	TRIM2	TRPC1
TRPM7	TSC22D3	TSEN54	TSHZ3	TSPAN13
TTC13	TTC26	TTC7B	TTLL9	TUBG1
U2SURP	UBA2	UBA3	UBE2K	UBTD2
UGP2	UNC13B	UQCRFS1	URI1	USP1
USP28	USP47	USP8	UTRN	VAPB
VBP1	VCAM1	VLDLR	VMA21	VPS37A
VPS54	VRK1	VTI1A	VWDE	WAPAL
WDFY2	WDR20	WDR44	WDR47	WFDC8
WIF1	WNT1	WWP1	XAGE3	XPOT
YIPF6	ZBTB18	ZC3H11A	ZC3H15	ZCCHC11
ZCCHC3	ZDHHC6	ZFC3H1	ZFP36L1	ZFYVE26
ZNF107	ZNF131	ZNF207	ZNF25	ZNF277
ZNF3	ZNF385B	ZNF468	ZNF644	ZNF655
ZNF777	AASDHPPT	ANKRD13C	APOBEC3F	ARHGAP17
ARHGAP18	ARHGAP21	ARHGAP32	ARHGAP40	C11orf30
C11orf73	C11orf87	C15orf27	C16orf87	C18orf21
C18orf54	C1orf101	C6orf211	CDKN2AIP	CTNNBIP1
DYNC1LI2	DYNC2LI1	FAM114A1	IRAK1BP1	KIAA1217
KIAA1429	KIAA2018	MIR99AHG	MPHOSPH6	MPHOSPH9
PAFAH1B2	PHOSPHO1	PPARGC1B	PPP1R12C	PPP1R14C
RAB3GAP2	SECISBP2L	SLC25A36	SLC4A1AP	TMEM167B
TMEM170A	TMEM178A	TMEM184C	TNFRSF11B	XRCC6BP1

3. 197 somatic mutation genes

DMD	DST	F8	FLG	NEB
NF1	SI	TNN	TNR	TTN
ZP4	ABCA13	ADCY8	AFF2	AHNAK
AHNAK2	AKAP6	ALMS1	ALPK2	ANK2
APOB	ASPM	ASTN1	ASTN2	ASXL3
ATRNL1	BAI3	BCLAF1	C15orf2	CACNA1E
CDH10	CDH12	CDH18	CDH9	CNTNAP2
CNTNAP5	COL11A1	COL22A1	COL3A1	COL5A2
COL6A3	CPS1	CRB1	CSMD1	CSMD2
CSMD3	CTNNA2	CTNND2	CUBN	DCAF4L2
DCHS2	DNAH11	DNAH3	DNAH5	DNAH7
DNAH8	DNAH9	DOCK2	DYSF	EGFR
EPHA3	EPHA5	EPHB6	FAM135B	FAM47B

FAM47C	FAM5C	FAM75A6	FAT1	FAT2
FAT3	FAT4	FBN2	FCGBP	FER1L6
FLG2	FMN2	FRG1B	GPR112	GPR158
GRIN2A	GRIN2B	GRM1	GRM8	HMCN1
HRNR	HYDIN	KEAP1	KIF2B	KLHL1
KRAS	LAMA1	LAMA2	LPHN3	LPPR4
LRFN5	LRP1B	LRP2	LRRC7	LTBP1
MACF1	MAGEC1	MKI67	MLL2	MLL3
MUC16	MUC17	MUC5B	MXRA5	MYH1
MYH13	MYH2	MYH7	MYH8	MYO18B
NALCN	NAV3	NCKAP5	NELL1	NLRP12
NLRP3	NOTCH4	NRXN1	OBSCN	ODZ1
ODZ2	PAK7	PAPPA2	PCDH10	PCDH11X
PCDH15	PCDH17	PCDHB4	PCDHB7	PCLO
PEG3	PKD1L1	PKHD1	PKHD1L1	PLCB1
PLXNA4	PRDM9	PRUNE2	PTPRD	PTPRT
PTPRZ1	PXDNL	RELN	RIMS2	RP1L1
RYR1	RYR2	RYR3	SALL1	SCN10A
SETBP1	SLC8A1	SLIT2	SLITRK1	SLITRK2
SNTG1	SORCS1	SORCS3	SPEF2	SPTA1
SSPO	STK11	SYNE1	SYNE2	TAF1L
TEX15	TLR4	TP53	TPTE	TRPA1
TRPS1	TSHZ2	TSHZ3	USH2A	VCAN
VPS13B	WDFY3	XIRP2	ZFHX4	ZNF208
ZNF423	ZNF521	ZNF536	ZNF804A	ZNF831
ADAMTS12	ADAMTS20	ANKRD30A	C1orf173	FLJ46321
KIAA1409	SLC39A12			

4. 1373 mRNA genes

ARC	BMX	C6	C7	C8B
CA1	CA3	CA4	CA7	CA9
CFD	CFP	CKM	CP	CR2
DAO	DCC	DES	F11	F12
FAP	FGR	GAL	GHR	GP9
HBB	HK3	ID4	IHH	IL6
KL	KLB	LHB	LPL	MAG
MB	MDK	MME	NMU	NPW
NRK	OGN	OTC	PAH	PBK
PF4	PRX	PZP	RP1	RS1
SP8	SPN	T	TEK	TK1
TNR	TTK	VGF	VIP	VIT
VWF	XDH	AADAC	AATK	ABCA12
ABCA3	ABCA8	ABCC11	ABCC13	ABI3BP
ABP1	ACADL	ACMSD	ACOXL	ACRV1
ACTN2	ACVRL1	ACY3	ADAM12	ADAMTS1

ADAMTS8	ADCY8	ADH1A	ADH1B	ADM2
ADRA1A	ADRA1D	ADRB1	ADRB2	AFF3
AGAP11	AGBL1	AGER	AGMAT	AGRP
AGTR1	AGTR2	AJAP1	AK3L1	AKAP14
AKAP2	AKR1B10	AKR1B15	AKR7A3	ALAS2
ALG1L	ALG1L2	ALOX15	ALOXE3	ALPK2
ALPP	ANGPT1	ANGPT4	ANGPTL1	ANGPTL5
ANGPTL7	ANKRD1	ANKRD22	ANKRD29	ANKS4B
ANLN	ANO2	ANXA8	ANXA8L2	AOC3
APCDD1L	APOA1	APOA5	APOBEC4	AQP1
AQP10	AQP4	ARHGAP6	ARL9	ARMC4
ARNTL2	ARSH	ART1	ART4	ASAH2B
ASF1B	ASPA	ASPG	ASPM	ATOH8
ATP10B	ATP13A4	ATP1A2	AURKA	AURKB
AVPR2	AWAT2	B3GNT3	B3GNT4	B3GNT6
BAI3	BARX1	BARX2	BCHE	BCL2L10
BCL2L15	BDNF	BEAN	BET3L	BEX1
BHLHA15	BIRC5	BMP8A	BMPER	BRIP1
BTNL8	BTNL9	BUB1	BUB1B	C11orf9
C1QTNF7	C1orf61	C1orf65	C1orf87	C1orf92
C2CD4A	C2orf39	C2orf40	C2orf48	C2orf62
C2orf71	C2orf73	C3orf16	C3orf50	C4orf31
C4orf7	C5orf34	C5orf46	C6orf25	C7orf10
C7orf16	C7orf52	C8orf77	C8orf84	C8orf85
C9orf24	C9orf84	CABYR	CACNA1E	CACNA1S
CACNG1	CACNG4	CACNG6	CALCRL	CALN1
CAMK2N2	CAMP	CAPSL	CARD14	CASC5
CASKIN1	CASP12	CASQ2	CAV1	CAV2
CAV3	CBLC	CCBE1	CCDC108	CCDC141
CCDC147	CCDC150	CCDC17	CCDC33	CCDC37
CCDC42B	CCDC48	CCDC54	CCDC60	CCDC65
CCDC81	CCDC85A	CCL14	CCL23	CCL25
CCL7	CCNA2	CCNB1	CCNB2	CCNE1
CCNO	CCRL1	CD101	CD19	CD300LG
CD36	CD52	CD5L	CD93	CDC20
CDC25A	CDC25C	CDC45	CDC6	CDCA2
CDCA3	CDCA5	CDCA7	CDCA8	CDH17
CDH19	CDH3	CDH5	CDHR3	CDHR4
CDK5R2	CDKN3	CDO1	CDT1	CDX1
CEACAM5	CEACAM7	CEACAM8	CELF5	CELSR3
CENPA	CENPE	CENPF	CENPI	CEP55
CES1	CES4	CFTR	CGNL1	CGREF1
CHD5	CHRDL1	CHRM1	CHRM2	CHRNA2
CHRNA5	CHRNA6	CHRNB4	CHST9	CILP2

CKAP2L	CLDN14	CLDN18	CLDN5	CLEC12A
CLEC14A	CLEC1A	CLEC3B	CLEC4M	CLIC3
CLIC5	CLSPN	CMTM2	CMTM5	CNFN
CNGA3	CNGA4	CNKSR2	CNTD2	CNTFR
CNTN6	COCH	COL10A1	COL11A1	COL17A1
COL1A1	COL22A1	COL29A1	COL3A1	COL4A3
COL4A6	COL6A6	COL7A1	COLEC10	COMP
CORO2B	COX4I2	COX6B2	CPAMD8	CPB2
CPNE4	CPNE7	CPXM1	CRABP1	CRABP2
CRTAC1	CRYAB	CRYGN	CSF3	CST1
CST2	CST4	CST5	CTHRC1	CTNND2
CWH43	CXCL13	CXCL14	CXCL2	CXCR1
CXCR2	CXorf22	CXorf41	CXorf61	CYP17A1
CYP1A1	CYP1A2	CYP24A1	CYP27B1	CYP27C1
CYP2F1	CYP3A7	CYP4B1	CYP4Z1	CYP7A1
CYS1	DACH1	DAPK2	DARC	DCDC2B
DCST1	DDIT4L	DEFA1B	DEPDC1	DEPDC1B
DGCR5	DGCR9	DGKI	DIO2	DLC1
DLGAP5	DLL3	DMBT1	DMBX1	DMRTA2
DNAH10	DNAH12	DNAH9	DNAI1	DNAI2
DNAJC12	DNAJC22	DNASE2B	DOK5	DPEP1
DPEP2	DPP6	DRP2	DTHD1	DUOX1
DUOXA1	DUSP13	DUSP27	DUSP9	DVWA
DYNLRB2	E2F7	E2F8	ECEL1	ECSCR
EDN3	EDNRB	EEF1A2	EFCAB1	EFHB
EFNA3	EFNA4	ELMOD1	EMCN	EME1
EMP2	EMR1	EMR3	ENDOU	ENTPD8
EPAS1	EPHA10	EPHX4	EPN3	EPR1
EPYC	ERBB4	ERCC6L	ESCO2	ESPL1
ETV4	EXO1	EYA4	EZH2	FABP4
FADS6	FAM107A	FAM111B	FAM131C	FAM150B
FAM154B	FAM155B	FAM163A	FAM166B	FAM167A
FAM177B	FAM178B	FAM179A	FAM180B	FAM181A
FAM183A	FAM46B	FAM64A	FAM83A	FAM92B
FAT3	FBN3	FCGR3B	FCN3	FCRL4
FCRL5	FER1L4	FERMT1	FEZF1	FGD5
FGF10	FGF11	FGF19	FGF2	FGFBP2
FGFR4	FGL1	FHL1	FHL5	FIBIN
FIGF	FMO2	FOLR3	FOSB	FOXA3
FOXD3	FOXE1	FOXF1	FOXH1	FOXI3
FOXM1	FOXP3	FPR2	FRMD3	FRMD5
FRMPD1	FRMPD4	FSD1	FUT2	FUT6
FUT9	FXYD1	GAD1	GALNT13	GALNT14
GAS2L2	GATA1	GBA3	GBP7	GCM1

GCNT3	GCOM1	GDF10	GFAP	GFI1B
GFRA1	GGTLC1	GIMAP8	GINS1	GINS2
GIPC2	GJB2	GJB6	GKN2	GLDN
GLIPR2	GLRA3	GLT25D2	GNG4	GNGT1
GOLGA7B	GOLM1	GPA33	GPC2	GPC3
GPC5	GPD1	GPER	GPIHBP1	GPM6A
GPM6B	GPR115	GPR120	GPR123	GPR146
GPR17	GPR19	GPR87	GPRIN1	GPRIN2
GPT2	GPX3	GRASP	GREB1L	GREM1
GRHL3	GRIA1	GRIK4	GRIN1	GRK5
GSG1L	GSTA3	GSTM5	GTSE1	GTSF1L
GUCA1A	GUCA2A	GUCY1B2	GYG2	GYPE
HAVCR1	HBA1	HBA2	HBEGF	HBG1
HBG2	HECW1	HEG1	HELLS	HEMGN
HHIP	HHIPL2	HIGD1B	HJURP	HMGA2
HMGB3	HMMR	HNF4G	HOTAIR	HOXA10
HOXB9	HOXC10	HOXC11	HOXC13	HPCA
HPDL	HPSE2	HRASLS	HS6ST2	HSD17B6
HSF2BP	HSPA12B	HSPB3	HSPB6	HTR3A
HTR3C	HYAL1	IBSP	IFLTD1	IGF2BP1
IGF2BP3	IGFL2	IGSF10	IGSF9	IL11
IL17REL	IL1A	IL1F5	IL1F7	IL1F9
IL1RL1	IL1RL2	IL22RA2	IL23A	IL31RA
IL33	IL5RA	IL7R	INMT	INSC
INSL3	IQGAP3	IQSEC3	IRX1	ITGA11
ITGA8	ITIH5	ITLN1	ITLN2	ITPKA
IZUMO1	JAM2	JPH4	JSRP1	KAL1
KANK3	KANK4	KCNA4	KCNE1	KCNG2
KCNH6	KCNIP1	KCNJ16	KCNK12	KCNK3
KCNMB2	KCNN4	KCNQ3	KCNT2	KHDRBS2
KIF11	KIF14	KIF15	KIF18B	KIF20A
KIF23	KIF26B	KIF2C	KIF4A	KIFC1
KIR2DL1	KIR3DL1	KIRREL2	KISS1R	KLF4
KLHL33	KLK10	KLK6	KPNA7	KREMEN2
KRT16	KRT27	KRT4	KRT6A	KRT79
KRT81	KRT83	KRT86	LAMP3	LCTL
LDB2	LDLRAD1	LEFTY2	LEMD1	LEPREL1
LGI2	LGI3	LGR4	LGSN	LHFPL3
LHFPL5	LHX2	LIMS2	LIN7A	LIPN
LOXHD1	LPPR3	LRRC18	LRRC2	LRRC36
LRRC50	LRRC67	LRRK2	LRRN3	LRRTM4
LTBP4	LY6D	LYPD1	LYPD2	LYPD3
LYVE1	MAD2L1	MAMDC2	MAP3K15	MAP7D2
MAPK4	MARCO	MASP1	MAST1	MC4R

MCHR1	MCM10	MELK	MESP1	MESP2
METTL7B	MEX3A	MFAP3L	MFAP4	MFI2
MGAT3	MGAT4C	MIOX	MKI67	MMP1
MMP10	MMP11	MMP12	MMP13	MMP28
MMP3	MND1	MNX1	MORN5	MRC1
MS4A15	MS4A2	MS4A7	MS4A8B	MSL3L2
MSR1	MT1M	MTNR1A	MUSK	MYADML2
MYBL2	MYBPH	MYCN	MYEOV	MYH11
MYH2	MYO3B	MYO7B	MYOC	MYOCD
MYOZ1	NCAPG	NCAPH	NCKAP5	NDC80
NDRG4	NECAB1	NEIL3	NEK2	NETO1
NGEF	NKAIN1	NKAIN4	NKPD1	NKX3-2
NLRC4	NMUR1	NOS1	NOX1	NPNT
NPR1	NPSR1	NPY5R	NQO1	NR2E1
NR4A3	NRG3	NRXN1	NTNG1	NTRK2
NTRK3	NUF2	NUSAP1	NXF3	NXPH1
NXPH3	NXPH4	ODAM	ODF3L1	OIP5
OLR1	ONECUT1	ONECUT2	OR1L8	OR2W3
OR51E1	OR7E37P	ORC1L	ORC6L	OSCAR
OSTBETA	OTX1	OVCH1	OVCH2	OVOL1
OXGR1	P2RX2	P2RX6	P2RY6	P4HA3
PACRG	PACSIN1	PADI1	PADI4	PAEP
PAK7	PCDH10	PCDH11X	PCDH15	PCDH9
PCOLCE2	PCP4	PCSK9	PCYT1B	PDE1C
PDK4	PDZD2	PDZRN4	PEAR1	PEBP4
PGLYRP1	PGLYRP3	PGLYRP4	PHACTR1	PHLDA2
PI16	PIP5K1B	PITX1	PITX2	PKHD1L1
PKMYT1	PKNOX2	PLA2G1B	PLA2G2F	PLA2G3
PLA2G4F	PLAC1	PLAC2	PLAC9	PLEK2
PLEKHN1	PLK1	PLP1	PLSCR2	PLXNB3
PMCH	PODNL1	PODXL2	POLE2	POLQ
POU3F2	POU4F1	PPAP2C	PPBP	PPP2R2C
PPP4R4	PRAM1	PRAME	PRB3	PRC1
PRDM12	PREX2	PRG4	PRIMA1	PRKG2
PROC	PROM2	PRPH	PRR11	PRSS1
PRSS3	PRSS35	PRSS50	PSAPL1	PSAT1
PTCRA	PTGES	PTGFR	PTH1R	PTPN21
PTPN5	PTPRB	PTPRH	PTPRQ	PTPRT
PTRF	PTTG3P	PVT1	PYCR1	RAB26
RAB3B	RAD51	RAD54L	RADIL	RAET1K
RAMP2	RAMP3	RANBP3L	RASAL1	RASGRF1
RASIP1	RBP2	RBP4	RCOR2	RDM1
RECQL4	REEP1	RETN	RFX8	RGS17
RGS20	RGS22	RGS6	RGS9	RHBDL1

RHBDL2	RHBG	RHOV	RIC3	RIMS4
RMST	RNF182	RNF183	RNF186	ROBO4
ROPN1L	RPH3A	RPSAP52	RRM2	RSPH4A
RSPO1	RSPO2	RSPO4	RTBDN	RTKN2
RTN4RL2	RXFP1	RXRG	S100A12	S100A2
S100A3	S100P	S1PR1	SALL1	SALL4
SCARA5	SCG5	SCGB1A1	SCN1A	SCN4B
SCN7A	SCN8A	SCUBE1	SDPR	SEC14L3
SEC14L4	SELP	SEMA3B	SEMA3G	SEMA5A
SEMA6A	SEMA6D	SEZ6	SFRP5	SFTA1P
SFTPA1	SFTPA2	SFTPC	SFTPD	SGCA
SGCG	SGEF	SGOL1	SH2D3C	SH2D4B
SH3GL2	SH3GL3	SHCBP1	SHOX2	SIRPB1
SIRPD	SIX1	SKA1	SKA3	SLC14A1
SLC19A3	SLC1A1	SLC24A2	SLC27A6	SLC29A4
SLC2A1	SLC2A5	SLC39A8	SLC46A2	SLC4A1
SLC5A4	SLC5A7	SLC5A9	SLC6A13	SLC6A4
SLC7A10	SLCO1A2	SLCO1B3	SLCO5A1	SLFNL1
SLIT2	SLIT3	SLITRK2	SLITRK3	SMAD6
SMC1B	SNTN	SOHLH2	SOSTDC1	SOX11
SOX17	SOX7	SPAG4	SPAG5	SPATA4
SPATS1	SPC24	SPC25	SPDEF	SPERT
SPHKAP	SPINK1	SPINK13	SPINK2	SPINLW1
SPOCK2	SPP1	SPP2	SPRED3	SPRR1B
SPTBN2	SRPK3	SRRM4	SSTR1	ST8SIA2
ST8SIA6	STAC	STEAP1	STIL	STOML3
STRA6	STX11	STX1A	STXBP6	STYK1
SULT1C4	SUSD2	SVEP1	SYN2	SYNGR3
SYNGR4	SYNPO2L	SYT12	SYT14	SYT15
SYT16	SYT4	SYT7	TAF7L	TAL1
TBX15	TBX3	TBX4	TCEAL2	TCF21
TCN1	TCTE1	TEKT1	TEKT2	TEKT3
TEKT5	TERT	TEX11	TEX19	TFAP2A
TFF1	TFR2	TGFBR3	TGM1	THBD
THBS2	TKTL1	TLL2	TMC2	TMEM100
TMEM139	TMEM145	TMEM146	TMEM156	TMEM171
TMEM190	TMEM212	TMEM232	TMEM59L	TMEM63C
TMEM82	TMEM88	TMEM90A	TMPRSS4	TNFSF11
TNNC1	TNNI2	TNNT2	TNNT3	TNS1
TNS4	TNXB	TOP2A	TPPP3	TPX2
TRHDE	TRIM15	TRIM31	TRIM54	TRIM58
TRIM67	TRIM71	TRIP13	TROAP	TRPC2
TRPM8	TSPAN7	TTC29	TTLL10	TTLL7
TTPA	TTYH1	TUBA4B	TUBB1	TUBB3

UBE2C	UBE2T	UCA1	UCN2	UHRF1
UMODL1	UNC45B	UNC5CL	UPK3A	UPK3B
USHBP1	VEPH1	VIL1	VIPR1	VPREB3
VSIG2	VSIG4	VSX1	VWA3A	VWA3B
VWC2	WDR16	WDR38	WDR63	WDR65
WFDC12	WFDC3	WFDC6	WFIKKN2	WIF1
WISP2	WNT3A	WNT7A	WWC2	XAGE1D
XAGE2	XKRX	XRCC2	YBX2	YSK4
ZBED2	ZBTB16	ZDHHC19	ZIC2	ZMYND10
ZNF385B	ZNF536	ZNF695	ZPLD1	ZWINT
ZYG11A	ADAMDEC1	ADAMTS14	ADAMTS16	ADAMTS18
ADAMTSL3	ADAMTSL4	ADCYAP1R1	ALS2CR12	ANKRD34B
ARHGAP31	ARHGEF15	B4GALNT4	BAIAP2L2	C10orf107
C10orf116	C10orf67	C10orf79	C11orf66	C11orf86
C11orf88	C12orf36	C12orf42	C13orf15	C13orf30
C13orf36	C13orf38	C14orf132	C14orf180	C14orf86
C15orf26	C15orf42	C15orf48	C16orf59	C17orf53
C18orf16	C18orf56	C19orf26	C19orf45	C19orf59
C19orf69	C19orf77	C1orf129	C1orf141	C1orf158
C1orf168	C1orf170	C1orf173	C1orf182	C1orf189
C1orf220	C20orf151	C20orf160	C20orf202	C20orf26
C20orf70	C20orf85	C21orf125	C22orf15	C22orf41
C6orf103	C6orf105	C6orf118	C6orf126	C6orf155
C6orf174	C6orf222	C8ORFK29	C9orf140	C9orf171
CACNA2D2	CATSPERB	COL6A4P2	DNASE1L3	ERVFRDE1
FAM189A1	FAM189A2	FLJ26850	FLJ34503	FLJ37543
HIST1H1B	HIST1H1D	HIST1H1E	HIST1H2AG	HIST1H2AJ
HIST1H2AL	HIST1H2AM	HIST1H2BO	HIST1H3J	HSD17B13
IL1RAPL2	KIAA0101	KIAA0408	KIAA1324L	KIAA1683
KRTAP4-1	LOC121838	LOC127841	LOC148709	LOC149620
LOC150197	LOC150622	LOC158376	LOC283392	LOC284100
LOC285629	LOC339674	LOC389791	LOC399815	LOC400804
LOC400891	LOC440356	LOC572558	LOC645323	LOC647946
LOC723809	LOC84740	MGC14436	MGC27382	MGC42105
NCRNA00160	NCRNA00162	PALM2-AKAP2	PPAPDC1A	PPP1R14D
RPL13AP17	RSPH10B2	SERPINA4	SERPINA9	SIGLEC11
SLC16A11	SLC22A18AS	SLC6A10P	ST6GALNAC5	TMEM132C
TMEM150B	TMEM184A	TNFRSF13C	LOC100131551	LOC100131726
LOC100144604	LOC100190938	LOC100287718		

Supplementary Material II. Detailed information of candidate genes obtained by SP method.

1. Candidate genes for methylation CpG site genes and microRNA target genes

Ensembl ID	Gene symbol	Betweenness	Permutation FDR
ENSP00000075120	SLC2A3	799	<0.001
ENSP00000204615	THPO	932	<0.001
ENSP00000248272	GAN	10	<0.001
ENSP00000252818	JUND	7269	<0.001
ENSP00000259216	CFC1	1	<0.001
ENSP00000262965	TCF3	2651	<0.001
ENSP00000265713	KAT6A	1	<0.001
ENSP00000290295	HOXB13	800	<0.001
ENSP00000293362	PSME3	800	<0.001
ENSP00000294309	TPCN2	4	<0.001
ENSP00000307479	ARNT2	799	<0.001
ENSP00000320081	C3orf58	1	<0.001
ENSP00000333188	FOXL2	801	<0.001
ENSP00000337088	MEN1	7269	<0.001
ENSP00000348815	HYLS1	800	<0.001
ENSP00000352262	MLL	7342	<0.001
ENSP00000361548	MPL	932	<0.001
ENSP00000376268	SEC14L1	1	<0.001

ENSP00000376350	TPCN1	1	<0.001
ENSP00000376849	CASP5	800	<0.001
ENSP00000384179	ZFPM2	2	<0.001
ENSP00000401435	VPS53	799	<0.001
ENSP00000403005	EFNA4	1597	<0.001
ENSP00000405890	PBX1	2529	<0.001
ENSP00000220478	SCG3	4	0.001
ENSP00000222725	LFNG	1727	0.001
ENSP00000241125	GJA3	788	0.001
ENSP00000253571	RLIM	800	0.001
ENSP00000256151	CCDC59	800	0.001
ENSP00000257829	NAT10	572	0.001
ENSP00000297261	SHH	2800	0.001
ENSP00000306245	FOS	7888	0.001
ENSP00000332353	PTCH1	3044	0.001
ENSP00000351141	WTAP	134	0.001
ENSP00000355140	HOXB1	800	0.001
ENSP00000363827	HSPG2	803	0.001
ENSP00000365682	TLE1	1598	0.001
ENSP00000221855	TBCB	2	0.002
ENSP00000291842	SHKBP1	800	0.002
ENSP00000319118	GSX2	800	0.002

ENSP00000321826	STXBP5	134	0.002
ENSP00000331057	TCF12	800	0.002
ENSP00000339328	PLAUR	1730	0.002
ENSP00000341550	SLC24A5	1	0.002
ENSP00000351209	EPHA2	1983	0.002
ENSP00000358497	RNGTT	932	0.002
ENSP00000360891	IFIT2	134	0.002
ENSP00000370256	FOXC1	799	0.002
ENSP00000222792	CHN2	134	0.003
ENSP00000240652	IAPP	134	0.003
ENSP00000263253	EP300	8814	0.003
ENSP00000295600	MITF	800	0.003
ENSP00000304915	IL13	930	0.003
ENSP00000328364	MAFA	799	0.003
ENSP00000344460	CBS	912	0.003
ENSP00000345487	QRFP	387	0.003
ENSP00000345752	MTMR2	2	0.003
ENSP00000355927	RPS6KC1	1	0.003
ENSP00000360076	SGIP1	795	0.003
ENSP00000364092	ASIP	785	0.003
ENSP00000373700	ALK	800	0.003
ENSP00000375629	LILRB2	802	0.003

ENSP00000388996	AP1M1	800	0.003
ENSP00000287934	FZD1	1595	0.004
ENSP00000298552	TSC1	791	0.004
ENSP00000304414	CXCR6	796	0.004
ENSP00000315997	LILRB1	800	0.004
ENSP00000331791	TBX1	799	0.004
ENSP00000337675	EBAG9	1	0.004
ENSP00000339916	LIMK2	134	0.004
ENSP00000340328	NYX	1	0.004
ENSP00000347710	OPHN1	134	0.004
ENSP00000349955	RPRD1A	134	0.004
ENSP00000359910	PSMA7	134	0.004
ENSP00000366603	TGOLN2	134	0.004
ENSP00000417132	BAP1	1063	0.004
ENSP00000230895	DAP	134	0.005
ENSP00000247026	NSRP1	134	0.005
ENSP00000269280	NLRP1	800	0.005
ENSP00000284154	GRAP	133	0.005
ENSP00000287322	BAG4	127	0.005
ENSP00000318057	EGR3	798	0.005
ENSP00000318128	BLOC1S4	4	0.005
ENSP00000339845	DROSHA	134	0.005

ENSP00000381634	SLC38A1	2	0.005
ENSP00000231228	IL12B	134	0.006
ENSP00000265529	KIF9	134	0.006
ENSP00000274711	LRRTM2	134	0.006
ENSP00000314897	ANGPT2	814	0.006
ENSP00000340688	LPHN1	134	0.006
ENSP00000344456	CTNNB1	9830	0.006
ENSP00000349275	NRG1	818	0.006
ENSP00000352673	ELF3	134	0.006
ENSP00000354541	NLGN1	943	0.006
ENSP00000358799	RBM15	799	0.006
ENSP00000359215	TLX1	798	0.006
ENSP00000284690	DHX32	134	0.007
ENSP00000317872	RBBP6	134	0.007
ENSP00000329715	DRG1	134	0.007
ENSP00000346886	GABPA	134	0.007
ENSP00000366347	NKX2-2	800	0.007
ENSP00000370521	AIPL1	796	0.007
ENSP00000387662	GCG	3370	0.007
ENSP00000254691	CARD6	134	0.008
ENSP00000265164	CASP6	134	0.008
ENSP00000310440	CHMP2A	798	0.008

ENSP00000320838	GSG1	134	0.008
ENSP00000331358	GAST	1284	0.008
ENSP00000334940	GGN	134	0.008
ENSP00000350990	TNKS1BP1	134	0.008
ENSP00000357674	SNAPIN	139	0.008
ENSP00000361423	ABL1	2071	0.008
ENSP00000226091	EFNB3	134	0.009
ENSP00000236192	VAMP4	930	0.009
ENSP00000247843	YEATS4	800	0.009
ENSP00000266085	TIMP3	134	0.009
ENSP00000309622	TFDP2	134	0.009
ENSP00000326432	CCR8	134	0.009
ENSP00000362744	RPS4X	134	0.009
ENSP00000382895	RPGRIP1	1	0.009
ENSP00000406359	HSPA1A	134	0.009
ENSP00000250111	ATP1B2	134	0.01
ENSP00000254661	RAMP1	134	0.01
ENSP00000257818	LMO2	936	0.01
ENSP00000258301	STX6	1329	0.01
ENSP00000283977	PGM3	134	0.01
ENSP00000302397	ATP1A3	2	0.01
ENSP00000315173	ZNF41	134	0.01

ENSP00000337194	PRPF4B	134	0.01
ENSP00000344871	MYO1F	134	0.01
ENSP00000354111	DNAJC5	764	0.01
ENSP00000392466	LDB1	800	0.01
ENSP00000401018	GINS3	798	0.01
ENSP00000308549	ADORA1	134	0.011
ENSP00000336666	AP1S1	153	0.011
ENSP00000384169	FBLN2	3	0.011
ENSP00000394033	KCNK2	134	0.011
ENSP00000263209	DGCR8	134	0.012
ENSP00000275603	CCT6A	134	0.012
ENSP00000307549	NPTX1	134	0.012
ENSP00000340507	TRIM24	134	0.012
ENSP00000356162	KISS1	387	0.012
ENSP00000365766	TIMM17B	134	0.012
ENSP00000328181	NOG	800	0.013
ENSP00000351363	MSMB	134	0.013
ENSP00000357692	S100A16	134	0.013
ENSP00000398644	NUB1	796	0.013
ENSP00000316042	HNRNPA0	500	0.014
ENSP00000354033	PCGF2	134	0.014
ENSP00000355566	TOMM20	267	0.014

ENSP00000355961	INTS7	796	0.014
ENSP00000357453	MAN1A1	7	0.014
ENSP00000367959	HTR2A	5	0.014
ENSP00000385021	FANCL	134	0.014
ENSP00000225893	HNF1B	134	0.015
ENSP00000264657	STAT3	8193	0.015
ENSP00000297562	AP5Z1	134	0.015
ENSP00000351407	ARNT	2622	0.015
ENSP00000357283	LMNA	932	0.015
ENSP00000407431	HLA-C	3827	0.015
ENSP00000170630	IL4R	1104	0.016
ENSP00000219473	USP10	227	0.016
ENSP00000312652	LEP	4184	0.016
ENSP00000340858	B2M	3999	0.016
ENSP00000362555	RNF19B	3	0.016
ENSP00000364398	HABP4	134	0.016
ENSP00000403557	PPP1R11	134	0.016
ENSP00000216410	GNPNAT1	134	0.017
ENSP00000273398	ATP6V1A	131	0.017
ENSP00000285398	ERCC3	131	0.017
ENSP00000344822	S100A13	12	0.017
ENSP00000404232	EFHC2	134	0.017

ENSP00000219244	CCL17	134	0.018
ENSP00000229307	NANOG	799	0.018
ENSP00000250894	MAPK8IP3	134	0.018
ENSP00000309968	ADAM17	134	0.018
ENSP00000312697	DMAP1	1063	0.018
ENSP00000313581	KLK2	134	0.018
ENSP00000334594	SLC10A7	134	0.018
ENSP00000262053	ATF1	134	0.019
ENSP00000276420	DOK2	304	0.019
ENSP00000318472	NCAM1	1330	0.019
ENSP00000343943	PPOX	1	0.019
ENSP00000229030	FZD10	795	0.02
ENSP00000294053	CLPB	134	0.02
ENSP00000299163	HIF1AN	134	0.02
ENSP00000354720	SMC3	1474	0.02
ENSP00000359174	SLC35A3	134	0.02
ENSP00000386171	ESRRG	134	0.02
ENSP00000305595	B3GNT2	133	0.021
ENSP00000405041	POU5F1	798	0.021
ENSP00000006053	CX3CL1	788	0.022
ENSP00000264193	CPOX	1	0.022
ENSP00000303423	FNTA	134	0.022

ENSP00000341848	GOLGB1	133	0.022
ENSP00000354003	GYPA	258	0.022
ENSP00000357311	CENPW	134	0.022
ENSP00000388241	KIF26A	134	0.022
ENSP00000264499	BBS7	267	0.023
ENSP00000272233	RHOB	134	0.023
ENSP00000342215	KIR2DL3	3827	0.023
ENSP00000354901	CXCL9	913	0.023
ENSP00000357656	FYN	5938	0.023
ENSP00000263431	PRKCG	788	0.024
ENSP00000290597	ALDH4A1	1	0.024
ENSP00000293831	EIF4A1	268	0.024
ENSP00000200181	ITGB4	921	0.025
ENSP00000251337	GNAT2	800	0.025
ENSP00000260950	MSTN	134	0.025
ENSP00000282903	PLOD2	1	0.025
ENSP00000346148	PRKAA1	134	0.025
ENSP00000371308	CENPJ	799	0.025
ENSP00000266744	ASCL1	134	0.026
ENSP00000297338	RAD21	197	0.026
ENSP00000311579	TNKS	134	0.026
ENSP00000351926	AP2A1	1057	0.026

ENSP00000244741	CDKN1A	1254	0.027
ENSP00000293288	BAX	962	0.027
ENSP00000346440	TCF4	134	0.027
ENSP00000354376	RAB25	134	0.027
ENSP00000256689	SLC38A2	132	0.028
ENSP00000278198	LRRC4C	134	0.028
ENSP00000293441	SHANK1	133	0.028
ENSP00000310723	DDX23	121	0.028
ENSP00000315167	ALOX12B	134	0.028
ENSP00000356529	RGS16	134	0.028
ENSP00000357244	CCT3	134	0.028
ENSP00000361473	KDM4A	134	0.028
ENSP00000380066	MAP4K1	788	0.028
ENSP00000335632	CHP1	134	0.029
ENSP00000382166	CX3CR1	788	0.029
ENSP00000269485	TNFRSF11A	268	0.03
ENSP00000281928	MED13L	134	0.03
ENSP00000305913	COL8A2	134	0.03
ENSP00000328216	ORAI1	134	0.03
ENSP00000240874	KALRN	134	0.031
ENSP00000255688	RARRES3	116	0.031
ENSP00000260867	TIMM23	393	0.031

ENSP00000371475	TP53BP1	134	0.031
ENSP00000385057	APOBEC3G	134	0.031
ENSP00000274353	BHMT	1	0.033
ENSP00000299339	CLDN10	133	0.033
ENSP00000304102	COPS6	134	0.033
ENSP00000314491	SRRT	1164	0.033
ENSP00000356694	FASLG	798	0.033
ENSP00000360217	RHAG	258	0.033
ENSP00000419923	KLF6	134	0.033
ENSP00000291582	AIRE	134	0.034
ENSP00000365811	SPAG6	134	0.034
ENSP00000383263	CCHCR1	2	0.034
ENSP00000168216	HSD17B10	134	0.035
ENSP00000249647	SNAP23	1304	0.035
ENSP00000300574	CRK	1621	0.035
ENSP00000326830	CLK1	134	0.035
ENSP00000367747	PLCH2	132	0.035
ENSP00000219172	CENPT	134	0.036
ENSP00000307939	GCC2	134	0.036
ENSP00000322229	FADS1	134	0.036
ENSP00000222005	CDC37	1357	0.037
ENSP00000386896	ITGA6	932	0.037

ENSP00000346879	NKX2-1	800	0.038
ENSP00000356623	CITED2	268	0.038
ENSP00000357748	BCCIP	134	0.038
ENSP00000366819	UCHL3	134	0.038
ENSP00000382791	GRIK1	134	0.038
ENSP00000254351	SDC1	249	0.039
ENSP00000264637	THRA	160	0.039
ENSP00000337722	ARL6	134	0.039
ENSP00000396439	RING1	134	0.04
ENSP00000218652	NDFIP2	132	0.041
ENSP00000252997	GATA5	134	0.041
ENSP00000258774	HUS1	134	0.041
ENSP00000338207	LMO1	134	0.041
ENSP00000410294	FGFR2	801	0.041
ENSP00000414237	INTS2	134	0.041
ENSP00000262105	MCM4	916	0.042
ENSP00000293897	SSTR5	6	0.042
ENSP00000320567	MRPS33	134	0.042
ENSP00000329684	GALR2	1	0.042
ENSP00000331514	ACTG1	135	0.042
ENSP00000352522	ATP6V1H	134	0.042
ENSP00000354777	TBKBP1	133	0.042

ENSP00000360183	STX16	267	0.042
ENSP00000281821	EPHA4	138	0.043
ENSP00000326261	SRRM1	134	0.043
ENSP00000184266	NDUFB4	1	0.044
ENSP00000228641	MYF6	133	0.044
ENSP00000296490	WDR82	134	0.044
ENSP00000347839	RAB11FIP2	268	0.044
ENSP00000352708	TRAPPC2	134	0.044
ENSP00000250448	FOXA1	817	0.045
ENSP00000263205	MED15	134	0.045
ENSP00000345728	ATP7A	134	0.045
ENSP00000358525	NGF	1572	0.045
ENSP00000264033	CBL	19835	0.046
ENSP00000281950	GEMIN6	134	0.046
ENSP00000355180	COL6A1	1	0.046
ENSP00000261636	ARL1	134	0.047
ENSP00000265351	XPO5	134	0.047
ENSP00000297373	PHKG1	134	0.047
ENSP00000233057	EIF2AK2	268	0.048
ENSP00000263025	MAPK3	169	0.048
ENSP00000317379	GLS	1	0.048
ENSP00000335657	CCK	398	0.048

ENSP00000246194	RALY	130	0.049
ENSP00000333769	BSG	800	0.049
ENSP00000339151	IKBKB	286	0.049
ENSP00000341032	WNT7B	795	0.049
ENSP00000349708	ZMYM6	1	0.049
ENSP00000378288	MYLK3	134	0.049
ENSP00000225831	CCL2	788	0.05
ENSP00000227507	CCND1	7091	0.05
ENSP00000253024	TRIM28	933	0.05
ENSP00000257963	ACVR1B	134	0.05
ENSP00000257789	ORC3	134	0.051
ENSP00000318374	LENG8	134	0.051
ENSP00000326819	FANCB	134	0.051
ENSP00000341170	PTN	10	0.051
ENSP00000400806	APTX	268	0.051
ENSP00000225603	CBX1	134	0.052
ENSP00000343782	ADRB3	2	0.052
ENSP00000355361	CD47	258	0.052
ENSP00000368678	AGRN	2	0.053
ENSP00000377148	AP1G1	647	0.053
ENSP00000390427	PPIL2	62	0.053
ENSP00000216540	SLC10A1	134	0.054

ENSP00000257497	ANXA1	134	0.054
ENSP00000322142	ING5	266	0.054
ENSP00000324274	ZCCHC10	134	0.054
ENSP00000335544	CCKBR	361	0.054
ENSP00000248933	SEZ6L	1	0.055
ENSP00000265773	SMARCA2	801	0.055
ENSP00000356541	SF3B5	134	0.055
ENSP00000360992	STAMBPL1	1	0.055
ENSP00000370408	CDX2	134	0.055
ENSP00000375863	HNRNPUL1	134	0.055
ENSP00000246337	UROD	1	0.056
ENSP00000298852	PSMC3	134	0.056
ENSP00000320604	FAXDC2	133	0.056
ENSP00000380227	ITGA4	1167	0.056
ENSP00000238112	CPSF3	134	0.057
ENSP00000295066	DPY30	134	0.057
ENSP00000337103	CHAT	268	0.057
ENSP00000363019	UBE2D1	134	0.057
ENSP00000249042	TST	3	0.058
ENSP00000280193	VEGFC	134	0.058
ENSP00000321345	IL23R	19	0.058
ENSP00000267085	CSAD	1	0.059

ENSP00000246032	STK35	134	0.06
ENSP00000264079	MCOLN1	133	0.06
ENSP00000353483	MAPK8	1974	0.06
ENSP00000361824	SPTAN1	1	0.06
ENSP00000227618	ANAPC15	133	0.061
ENSP00000265729	SRI	134	0.061
ENSP00000278616	ATM	2072	0.061
ENSP00000303686	NTSR2	2	0.061
ENSP00000355599	TSNAX	134	0.061
ENSP00000222139	EPOR	134	0.062
ENSP00000300134	STAT6	1335	0.062
ENSP00000349960	ACTB	401	0.062
ENSP00000354532	PNP	7	0.062
ENSP00000245323	EFNB2	140	0.063
ENSP00000249373	SMO	134	0.063
ENSP00000250617	ARHGEF6	134	0.063
ENSP00000255465	CCNA1	134	0.063
ENSP00000265012	GCNT2	2	0.063
ENSP00000327145	FLNC	134	0.063
ENSP00000349577	PRODH	2	0.063
ENSP00000380378	PAFAH1B1	136	0.063
ENSP00000407964	NEDD1	132	0.063

ENSP00000261366	LMNB1	929	0.064
ENSP00000223208	CEP41	133	0.065
ENSP00000300737	STIM1	134	0.065
ENSP00000370503	CCM2	134	0.065
ENSP00000226574	NFKB1	268	0.066
ENSP00000228958	EIF2B1	134	0.067
ENSP00000234590	ENO1	134	0.067
ENSP00000255380	CHRM3	1	0.067
ENSP00000264708	POMC	1725	0.067
ENSP00000360798	EPS15	1583	0.067
ENSP00000362166	MEAF6	266	0.067
ENSP00000296084	RYK	133	0.068
ENSP00000308236	COMMD1	134	0.068
ENSP00000354623	DFNB31	134	0.068
ENSP00000005279	SYNRG	640	0.069
ENSP00000263980	SLC9A1	134	0.069
ENSP00000309555	HCFC1	1041	0.069
ENSP00000358994	MYO6	135	0.069
ENSP00000315955	FOXA2	286	0.07
ENSP00000257254	APLNR	8	0.071
ENSP00000360672	PARD6B	134	0.071
ENSP00000236671	CTSD	134	0.072

ENSP00000256379	MED6	136	0.072
ENSP00000295685	ARPC2	134	0.072
ENSP00000346155	UCKL1	2	0.072
ENSP00000261267	LYZ	134	0.073
ENSP00000345656	VAPA	134	0.073
ENSP00000348107	C1D	134	0.073
ENSP00000228841	MYL2	134	0.074
ENSP00000297518	CDK5	1668	0.074
ENSP00000358867	GNAI3	4	0.075
ENSP00000361125	VEGFA	2011	0.075
ENSP00000366843	ATXN2	134	0.075
ENSP00000254480	SMARCC1	861	0.076
ENSP00000257899	BLOC1S1	400	0.076
ENSP00000298281	PCF11	133	0.076
ENSP00000307078	KIF5B	134	0.076
ENSP00000330032	UPP1	1	0.076
ENSP00000338785	STARD13	122	0.076
ENSP00000358857	EMD	135	0.076
ENSP00000254322	DNAJB1	134	0.077
ENSP00000261890	RAB11A	268	0.077
ENSP00000346566	CKAP5	134	0.077
ENSP00000348986	INS-IGF2	2080	0.077

ENSP00000353701	DPP3	134	0.077
ENSP00000367802	TAF1C	134	0.077
ENSP00000384515	PARVB	134	0.077
ENSP00000413234	AP2A2	531	0.077
ENSP00000250495	NEDD8	1064	0.078
ENSP00000282344	USP12	134	0.078
ENSP00000306881	SEC23A	268	0.078
ENSP00000322016	PUF60	134	0.078
ENSP00000324740	YES1	133	0.078
ENSP00000377446	SUCLG1	267	0.078
ENSP00000168712	FGF4	2	0.079
ENSP00000334458	GATA4	802	0.079
ENSP00000344741	INSIG1	134	0.079
ENSP00000254950	VPS4A	798	0.08
ENSP00000263026	EEF2K	12	0.08
ENSP00000305892	TMEM208	133	0.08
ENSP00000340820	MAPT	1657	0.08
ENSP00000350198	SSTR2	133	0.08
ENSP00000366976	KRTAP1-4	134	0.081
ENSP00000389184	MARK2	134	0.081
ENSP00000257895	RDH5	1	0.082
ENSP00000332592	SPAG16	134	0.083

ENSP00000355050	CTNNBL1	134	0.083
ENSP00000361310	POLH	134	0.083
ENSP00000162330	BCAR1	905	0.084
ENSP00000247866	NDUFB2	134	0.084
ENSP00000322570	POLE	369	0.084
ENSP00000342952	ADCY2	138	0.084
ENSP00000242067	BBS9	401	0.086
ENSP00000309629	CFL1	139	0.086
ENSP00000348965	DYNC1H1	134	0.086
ENSP00000354960	COLGALT2	1	0.086
ENSP00000254998	NXT1	268	0.087
ENSP00000260570	IFT172	12	0.087
ENSP00000267845	HDC	131	0.087
ENSP00000354119	LAT	133	0.087
ENSP00000360372	CYP2C19	2	0.087
ENSP00000316854	ATOX1	134	0.088
ENSP00000333633	MTA1	392	0.088
ENSP00000255266	PDE6A	1	0.089
ENSP00000371682	DCAF16	120	0.089
ENSP00000264839	RIMS1	268	0.09
ENSP00000357392	EFNA1	264	0.091
ENSP00000357775	UROS	1	0.091

ENSP00000265641	CPT1A	134	0.092
ENSP00000301838	FADD	915	0.092
ENSP00000278385	CD44	143	0.093
ENSP00000302665	IGF1	3012	0.093
ENSP00000368683	EDN1	793	0.093
ENSP00000416753	MUC15	22	0.094
ENSP00000260130	SDCBP	248	0.095
ENSP00000265459	NRXN2	111	0.095
ENSP00000305529	SIRPG	230	0.095
ENSP00000363021	RPA2	134	0.095
ENSP00000363822	AR	1644	0.095
ENSP00000206249	ESR1	11996	0.096
ENSP00000262134	LPCAT2	134	0.096
ENSP00000271526	PRCC	62	0.096
ENSP00000326630	ZFPM1	8	0.096
ENSP00000350199	AP1B1	4	0.096
ENSP00000401303	SHC1	2309	0.096
ENSP00000164133	PPP2R5B	133	0.097
ENSP00000225655	PFN1	272	0.097
ENSP00000242152	NPY	807	0.097
ENSP00000262958	GNA15	127	0.097
ENSP00000301843	CTTN	134	0.097

ENSP00000303830	INSR	1922	0.097
ENSP00000263697	DNAJC8	133	0.099
ENSP00000356256	TIMM17A	393	0.099
ENSP00000358242	PNISR	134	0.099
ENSP00000359151	DBT	94	0.099
ENSP00000276414	GNRH1	522	0.1
ENSP00000254584	ARFIP2	134	0.101
ENSP00000351486	NTRK1	2023	0.101
ENSP00000384573	DAZ1	2	0.101
ENSP00000299626	ALG8	1	0.102
ENSP00000336740	LIMK1	272	0.103
ENSP00000396620	NFYC	133	0.103
ENSP00000272937	HES6	2	0.104
ENSP00000318297	RUVBL1	1055	0.104
ENSP00000323858	DDX54	133	0.104
ENSP00000216832	PNN	268	0.105
ENSP00000254227	NR0B2	267	0.105
ENSP00000256759	FST	134	0.105
ENSP00000349052	OSCP1	4	0.105
ENSP00000367766	RPGR	1	0.105
ENSP00000035307	CHPF2	134	0.106
ENSP00000303325	TACR3	2	0.107

ENSP00000043402	RTN4R	134	0.108
ENSP00000175506	ASNS	264	0.108
ENSP00000284384	PRKCA	1055	0.109
ENSP00000318775	ANAPC4	133	0.109
ENSP00000320866	CALR	1073	0.109
ENSP00000343313	ATG5	134	0.109
ENSP00000359518	MRGBP	134	0.109
ENSP00000307567	QARS	1	0.11
ENSP00000360882	COL5A1	2	0.11
ENSP00000331327	WT1	134	0.111
ENSP00000376822	STEAP3	132	0.112
ENSP00000276689	NDUFB9	134	0.113
ENSP00000220751	RIPK2	134	0.114
ENSP00000236147	SELL	134	0.114
ENSP00000266427	ETV6	133	0.114
ENSP00000296785	ANKRA2	7	0.114
ENSP00000363435	ITPR3	36	0.114
ENSP00000411286	GABBR1	2	0.114
ENSP00000305464	APLN	1	0.115
ENSP00000316578	SUZ12	134	0.115
ENSP00000361202	IRS4	133	0.115
ENSP00000370719	ITSN1	134	0.115

ENSP00000410076	CASP1	800	0.115
ENSP00000261813	PFDN1	134	0.116
ENSP00000290974	ZFYVE28	134	0.116
ENSP00000351446	WDR5	1175	0.116
ENSP00000249344	STRIP2	5	0.117
ENSP00000262519	SETD1A	134	0.117
ENSP00000328777	EFNA5	138	0.117
ENSP00000300289	PDIA3	1022	0.118
ENSP00000307491	WDR48	134	0.118
ENSP00000219548	STUB1	1486	0.12
ENSP00000360645	MAN1B1	127	0.12
ENSP00000228837	FGF6	799	0.121
ENSP00000243077	LRP1	146	0.122
ENSP00000265354	SRF	1062	0.122
ENSP00000251102	CNGB1	134	0.123
ENSP00000352425	WASF1	9	0.124
ENSP00000353731	DPP4	138	0.124
ENSP00000355536	MTR	135	0.124
ENSP00000366563	PIK3CD	132	0.124
ENSP00000262305	RAB11FIP3	268	0.126
ENSP00000290039	CACHD1	3	0.126
ENSP00000304283	RAC3	133	0.126

ENSP00000217901	IDH3G	1	0.127
ENSP00000246041	AP5S1	133	0.127
ENSP00000262304	PKD1	128	0.127
ENSP00000245157	BBS2	401	0.129
ENSP00000313199	HNRNPD	389	0.129
ENSP00000327541	BLZF1	109	0.13
ENSP00000362578	RNF8	130	0.13
ENSP00000364839	ASXL1	26	0.13
ENSP00000368464	KIF24	1	0.13
ENSP00000301457	NDUFA7	1	0.131
ENSP00000377941	ACTN1	134	0.131
ENSP00000204604	CHRD	134	0.132
ENSP00000262320	AXIN1	3159	0.133
ENSP00000260810	TOPBP1	249	0.134
ENSP00000354497	FAN1	132	0.134
ENSP00000354927	MAP3K3	134	0.134
ENSP00000362014	DNM1	402	0.134
ENSP00000358866	FLNA	706	0.136
ENSP00000262186	KCNH2	799	0.138
ENSP00000278715	HMBS	1	0.138
ENSP00000308938	PLG	2387	0.138
ENSP00000249075	LIF	794	0.139

ENSP00000355747	PSEN2	134	0.139
ENSP00000362795	CXCR3	127	0.139
ENSP00000394794	PTPN13	2	0.139
ENSP00000226299	LAP3	1	0.14
ENSP00000271411	POU2F1	132	0.14
ENSP00000383623	MLLT4	797	0.14
ENSP00000248996	GNAZ	130	0.141
ENSP00000252034	ELN	134	0.141
ENSP00000284995	TSEN2	133	0.141
ENSP00000352257	XRCC6	268	0.141
ENSP00000375809	ERCC2	134	0.141
ENSP00000263464	BIRC3	134	0.142
ENSP00000264554	SHC2	282	0.142
ENSP00000296412	ADH5	1	0.142
ENSP00000344468	SDC3	10	0.142
ENSP00000348573	AKAP9	2	0.142
ENSP00000265734	CDK6	133	0.144
ENSP00000340330	KAT5	2529	0.144
ENSP00000302234	CCL11	138	0.145
ENSP00000359506	FMR1	230	0.145
ENSP00000372975	HLA-C	801	0.145
ENSP00000229135	IFNG	268	0.146

ENSP00000260356	THBS1	42	0.146
ENSP00000338862	BRIX1	134	0.146
ENSP00000247161	ELK1	37	0.147
ENSP00000364519	TFDP1	134	0.147
ENSP00000370938	CDK8	327	0.147
ENSP00000227378	HSPA8	2456	0.148
ENSP00000019103	SCTR	2	0.149
ENSP00000223127	PLOD3	1	0.149
ENSP00000263817	ABCB11	134	0.149
ENSP00000267082	ITGB7	691	0.151
ENSP00000327801	P4HB	135	0.151
ENSP00000342011	XRCC4	134	0.151
ENSP00000369855	ASB9	134	0.151
ENSP00000264126	GPSM2	1	0.152
ENSP00000270538	TIMM44	393	0.152
ENSP00000310771	GRB7	84	0.152
ENSP00000288602	BRAF	134	0.153
ENSP00000314813	OAZ1	400	0.153
ENSP00000335333	PIP5K1C	132	0.154
ENSP00000240055	NFYB	133	0.156
ENSP00000263036	OPTN	260	0.156
ENSP00000261558	AP5M1	133	0.158

ENSP00000311344	PPP2R1B	66	0.158
ENSP00000362082	CCND3	134	0.158
ENSP00000256497	EDEM1	127	0.159
ENSP00000268171	FURIN	134	0.159
ENSP00000156109	GPKOW	134	0.16
ENSP00000333194	RGS19	46	0.16
ENSP00000321326	F2R	123	0.161
ENSP00000347733	TRRAP	502	0.161
ENSP00000359206	BTRC	156	0.162
ENSP00000368350	TPT1	393	0.162
ENSP00000338799	IL6ST	794	0.164
ENSP00000267101	ERBB3	939	0.165
ENSP00000319664	NUDC	2	0.165
ENSP00000303019	GPHN	402	0.166
ENSP00000319060	CAMK2G	134	0.166
ENSP00000157812	PSMC4	763	0.167
ENSP00000164247	KCNAB2	1	0.167
ENSP00000305422	CEBPB	40	0.167
ENSP00000264039	GPC1	1	0.168
ENSP00000298130	SPTSSA	132	0.168
ENSP00000316029	TLN1	132	0.168
ENSP00000173229	NTN1	417	0.169

ENSP00000340766	SMG7	133	0.17
ENSP00000204961	EFNB1	52	0.171
ENSP00000296140	CCR1	134	0.171
ENSP00000351049	PAK4	266	0.171
ENSP00000368914	PSTPIP1	134	0.171
ENSP00000262188	SMARCD3	122	0.173
ENSP00000304704	CLP1	133	0.173
ENSP00000338345	SNCA	785	0.173
ENSP00000225402	AATF	566	0.174
ENSP00000231790	MLH1	260	0.174
ENSP00000259089	BLK	129	0.174
ENSP00000261037	COL8A1	132	0.175
ENSP00000295598	ATP1A1	266	0.175
ENSP00000359297	NSDHL	133	0.175
ENSP00000361405	MMP9	286	0.176
ENSP00000359539	GOT1	1	0.177
ENSP00000361151	CEL	134	0.177
ENSP00000370473	IGFBP3	2341	0.177
ENSP00000407401	PEX5	134	0.177
ENSP00000313922	OAZ3	132	0.178
ENSP00000342755	RNF41	134	0.178
ENSP00000302707	FPR1	130	0.179

ENSP00000300935	RAB8A	266	0.18
ENSP00000361892	STK4	134	0.181
ENSP00000375777	STRN4	533	0.181
ENSP00000339992	MYB	932	0.182
ENSP00000341344	GGA1	926	0.182
ENSP00000317992	NOC2L	133	0.183
ENSP00000324549	CYFIP1	230	0.183
ENSP00000360916	VAV2	279	0.184
ENSP00000220584	FDFT1	267	0.185
ENSP00000257430	APC	134	0.185
ENSP00000358022	MCL1	1443	0.188
ENSP00000360683	PTPN1	413	0.188
ENSP00000256383	EIF2S1	530	0.189
ENSP00000311360	RAD9A	139	0.189
ENSP00000316460	FYB	155	0.189
ENSP00000359939	EXOSC1	3	0.189
ENSP00000261837	GNB5	118	0.19
ENSP00000346659	OCA2	10	0.19
ENSP00000298532	SNAPC4	124	0.191
ENSP00000254066	RARA	222	0.193
ENSP00000330393	LEPR	62	0.193
ENSP00000331817	ALYREF	134	0.193

ENSP00000338868	PHF8	139	0.193
ENSP00000357206	NES	3	0.193
ENSP00000293970	TBC1D24	134	0.194
ENSP00000340698	GIPC1	159	0.194
ENSP00000248923	GGT1	1	0.195
ENSP00000227752	IL10RA	134	0.196
ENSP00000261783	ARG2	262	0.197
ENSP00000331746	CALCA	221	0.197
ENSP00000405965	SUMO2	134	0.197
ENSP00000228307	PXN	657	0.198
ENSP00000323967	SMARCE1	1	0.198
ENSP00000329357	SP1	1702	0.199
ENSP00000232461	GNAT1	266	0.2
ENSP00000290541	PSMB4	533	0.2
ENSP00000388107	UBA52	134	0.2
ENSP00000206542	OSGEP	133	0.201
ENSP00000229769	FANCE	272	0.201
ENSP00000265969	KCNC1	1	0.201
ENSP00000297564	COX6C	133	0.201
ENSP00000316605	CNGB3	1	0.201
ENSP00000323050	RBBP8	260	0.201
ENSP00000333982	NDEL1	271	0.201

ENSP00000339428	SOCS2	2	0.201
ENSP00000381293	NSF	76	0.201
ENSP00000313681	SPHK1	268	0.202
ENSP00000265333	VDAC1	268	0.203
ENSP00000256958	SLCO1B1	134	0.204
ENSP00000382723	AGPAT1	3	0.205
ENSP00000305480	FEN1	134	0.206
ENSP00000348786	RAP1A	266	0.206
ENSP00000262887	XRCC1	268	0.207
ENSP00000293330	HCRT	134	0.207
ENSP00000311449	RAB6A	134	0.208
ENSP00000326550	TACC3	138	0.208
ENSP00000084795	RPL18	132	0.209
ENSP00000216341	GZMB	134	0.209
ENSP00000221265	PAF1	134	0.209
ENSP00000324173	HSPA5	134	0.209
ENSP00000313391	DAB2	129	0.21
ENSP00000332973	SMAD3	982	0.21
ENSP00000338018	HIF1A	8824	0.21
ENSP00000346389	MEF2A	106	0.21
ENSP00000363092	PRKG1	268	0.21
ENSP00000369889	COL2A1	134	0.21

ENSP00000234160	GORASP2	109	0.211
ENSP00000272521	TMEM177	1	0.211
ENSP00000333275	NR2C1	111	0.211
ENSP00000351273	CASP8	146	0.212
ENSP00000361162	TOE1	4	0.212
ENSP00000196061	PLOD1	132	0.213
ENSP00000414982	KLC1	134	0.213
ENSP00000216223	IL2RB	493	0.214
ENSP00000277541	NOTCH1	2896	0.214
ENSP00000411672	ATP6V0E2	13	0.215
ENSP00000334188	PFDN5	398	0.216
ENSP00000242208	INHBA	1	0.218
ENSP00000269571	ERBB2	1470	0.218
ENSP00000361219	GTF3C4	134	0.218
ENSP00000259808	RIPK1	1036	0.219
ENSP00000338369	CELA3B	1	0.219
ENSP00000307387	PDCD6IP	1195	0.22
ENSP00000311677	PPP1R8	1062	0.22
ENSP00000351997	MAP2K6	460	0.221
ENSP00000303522	TACR1	3	0.222
ENSP00000360268	ALDH18A1	131	0.222
ENSP00000287647	FANCD2	443	0.223

ENSP00000258962	SRSF1	283	0.224
ENSP00000347198	SRGAP1	133	0.224
ENSP00000372326	FECH	7	0.224
ENSP00000261769	CDH1	939	0.225
ENSP00000262854	HUWE1	129	0.225
ENSP00000276062	NDUFB11	5	0.225
ENSP00000286317	MED7	2	0.225
ENSP00000364649	SDHB	269	0.225
ENSP00000367830	PRKCZ	134	0.225
ENSP00000001008	FKBP4	134	0.226
ENSP00000247461	CANX	1022	0.226
ENSP00000358105	APH1A	134	0.226
ENSP00000340409	SMPD1	134	0.227
ENSP00000396308	DHFR	134	0.227
ENSP00000006275	TRAPPC6A	134	0.228
ENSP00000368119	GALT	134	0.228
ENSP00000406037	KAT8	134	0.228
ENSP00000221573	SNAPC2	124	0.23
ENSP00000318585	BACE1	134	0.23
ENSP00000257904	CDK4	830	0.231
ENSP00000326031	PPP1CA	932	0.231
ENSP00000394624	OPRM1	134	0.232

ENSP00000232458	ECT2	2	0.233
ENSP00000306497	KCNJ4	4	0.233
ENSP00000345731	DLG1	268	0.233
ENSP00000313007	PABPC1	268	0.234
ENSP00000366565	VPS28	267	0.236
ENSP00000317337	CD300LB	126	0.237
ENSP00000350720	SMARCA4	1168	0.237
ENSP00000263168	CAPZA1	134	0.238
ENSP00000312122	SEC13	268	0.238
ENSP00000340466	GANAB	6	0.238
ENSP00000352516	DNMT1	307	0.238
ENSP00000296755	MAP1B	25	0.239
ENSP00000401445	ERN1	134	0.239
ENSP00000209875	CBX5	932	0.24
ENSP00000233146	MSH2	394	0.241
ENSP00000349465	PICK1	134	0.241
ENSP00000245206	GOT2	11	0.242
ENSP00000307235	EIF2AK3	508	0.243
ENSP00000312999	GNAI2	272	0.244
ENSP00000356713	IFNGR1	266	0.245
ENSP00000261531	SNW1	134	0.247
ENSP00000354251	NCKAP1	239	0.247

ENSP00000276603	TERF1	134	0.248
ENSP00000261507	MSMO1	133	0.249
ENSP00000367615	APRT	2	0.25
ENSP00000297268	COL1A2	135	0.251
ENSP00000361965	ADA	4	0.251
ENSP00000245451	BMP4	406	0.252
ENSP00000316032	NUP98	268	0.254
ENSP00000340944	PTPN11	1082	0.254
ENSP00000367408	CASK	656	0.254
ENSP00000336701	RAD51C	133	0.256
ENSP00000365775	MTHFR	132	0.257
ENSP00000306330	YWHAG	5	0.259
ENSP00000344352	ATF3	1038	0.259
ENSP00000360149	ALG6	1	0.26
ENSP00000262367	CREBBP	4389	0.262
ENSP00000349594	ELAVL4	1	0.262
ENSP00000363079	MBL2	133	0.262
ENSP00000216797	NFKBIA	228	0.263
ENSP00000267430	FANCM	140	0.264
ENSP00000354782	CD247	133	0.264
ENSP00000302150	PRL	843	0.265
ENSP00000401508	NAPRT1	127	0.265

ENSP00000261799	PDGFRB	409	0.266
ENSP00000280357	IL18	2	0.266
ENSP00000284957	RABGEF1	1031	0.266
ENSP00000347719	TBCD	3	0.266
ENSP00000262629	TYROBP	3838	0.267
ENSP00000349547	RASSF1	114	0.267
ENSP00000256857	GRP	553	0.27
ENSP00000221233	EXOSC5	134	0.271
ENSP00000246891	CSN1S1	134	0.271
ENSP00000324806	GSK3B	1887	0.272
ENSP00000347942	RET	268	0.272
ENSP00000320180	GHRHR	4	0.273
ENSP00000373614	SELPLG	134	0.273
ENSP00000226218	SEBOX	77	0.274
ENSP00000273588	AMT	10	0.274
ENSP00000386284	ALAD	1	0.274
ENSP00000231509	NR3C1	53	0.275
ENSP00000315859	RNPS1	410	0.275
ENSP00000356520	DHX9	8	0.275
ENSP00000262013	SPAG9	134	0.276
ENSP00000264380	PIKFYVE	2	0.276
ENSP00000354394	STAT1	1683	0.277

ENSP00000358335	MAP3K7	460	0.277
ENSP00000365891	WAS	686	0.277
ENSP00000299518	IDH3A	1	0.278
ENSP00000358323	TXNIP	133	0.278
ENSP00000231449	IL4	124	0.28
ENSP00000339109	ANAPC1	1327	0.28
ENSP00000230340	BYSL	1	0.281
ENSP00000363763	EPHB2	22	0.281
ENSP00000265094	FBXW11	12	0.284
ENSP00000336790	ATF4	904	0.285
ENSP00000292303	CCR5	756	0.286
ENSP00000365851	BMI1	268	0.286
ENSP00000264972	ZAP70	126	0.287
ENSP00000280704	LDHC	1	0.287
ENSP00000302961	HSPA4	341	0.287
ENSP00000234296	ORC2	5	0.288
ENSP00000339007	GRB2	4313	0.288
ENSP00000256443	CDK7	402	0.289
ENSP00000353224	TFRC	106	0.29
ENSP00000361186	TP53RK	133	0.292
ENSP00000400591	SNRPE	53	0.292
ENSP00000263923	KDR	134	0.293

ENSP00000320940	NCOA1	1825	0.293
ENSP00000337053	SEL1L	127	0.294
ENSP00000239223	DUSP1	53	0.295
ENSP00000155840	KCNQ1	2	0.296
ENSP00000179259	C12orf5	134	0.298
ENSP00000315791	CSTF3	401	0.298
ENSP00000216442	ATP6V1D	131	0.299
ENSP00000263354	NAPA	76	0.299
ENSP00000301071	TUBA1A	133	0.299
ENSP00000251968	TSG101	1466	0.301
ENSP00000269397	CBX4	401	0.301
ENSP00000210313	PSMD5	1	0.303
ENSP00000308208	MMP14	1	0.304
ENSP00000237527	GHRH	4	0.305
ENSP00000293379	ITGA5	2127	0.307
ENSP00000360532	CDC5L	2	0.307
ENSP00000262477	RABEP1	927	0.308
ENSP00000262584	RPL8	122	0.308
ENSP00000299543	CTDP1	481	0.308
ENSP00000334100	EXOC7	134	0.308
ENSP00000345530	NEDD4	132	0.308
ENSP00000360515	SUPT3H	133	0.309

ENSP00000231454	IL5	2	0.311
ENSP00000260947	BARD1	535	0.311
ENSP00000315644	TYMS	2564	0.313
ENSP00000363868	ABCA1	266	0.314
ENSP00000264335	YWHAE	6	0.315
ENSP00000357753	IVL	131	0.315
ENSP00000387699	CREB1	134	0.315
ENSP00000220764	DECR1	132	0.319
ENSP00000217109	CSTF1	401	0.32
ENSP00000262554	SPTLC1	132	0.32
ENSP00000317955	EEA1	21	0.32
ENSP00000320949	CNOT1	35	0.321
ENSP00000358554	BCAS2	55	0.323
ENSP00000223029	AIMP2	931	0.324
ENSP00000261819	ANAPC5	5	0.326
ENSP00000263967	PIK3CA	356	0.326
ENSP00000324248	PENK	42	0.328
ENSP00000334061	HDAC6	133	0.328
ENSP00000364898	SYK	3968	0.328
ENSP00000371432	PRLR	793	0.328
ENSP00000363880	CUL2	2	0.33
ENSP00000368104	BMP2	970	0.33

ENSP00000271620	PRUNE	1	0.332
ENSP00000343274	INTS8	265	0.332
ENSP00000229794	MAPK14	1034	0.337
ENSP00000245932	VASP	134	0.34
ENSP00000326366	PSEN1	958	0.34
ENSP00000241416	ACVR2A	1	0.341
ENSP00000413625	FNBP1	115	0.341
ENSP00000338934	EZR	2117	0.342
ENSP00000263331	POLR1B	133	0.343
ENSP00000315702	MOB4	426	0.343
ENSP00000332643	NDN	45	0.343
ENSP00000281453	MLF1IP	125	0.344
ENSP00000341538	SEC61G	268	0.344
ENSP00000343204	JAK1	794	0.344
ENSP00000223023	WASL	1237	0.345
ENSP00000302564	BCL2L1	295	0.345
ENSP00000303191	PLRG1	2	0.345
ENSP00000296871	CSF2	214	0.346
ENSP00000297151	SLU7	428	0.346
ENSP00000374372	SPTB	1	0.346
ENSP00000303507	BCR	28	0.347
ENSP00000337014	HFE2	4	0.348

ENSP00000383059	ARGLU1	38	0.349
ENSP00000264515	RBBP5	14	0.35
ENSP00000304350	PRPF8	55	0.351
ENSP00000339353	CPSF1	134	0.352
ENSP00000296223	POLR2H	4	0.353
ENSP00000359663	CD40LG	133	0.353
ENSP00000391901	PHF1	139	0.353
ENSP00000262613	SLC9A3R1	2228	0.354
ENSP00000246071	SNRPB2	9	0.355
ENSP00000242057	AHR	1058	0.356
ENSP00000382373	DAZL	2	0.358
ENSP00000339399	CRYZ	2	0.361
ENSP00000370557	MIS12	3	0.361
ENSP00000257181	PRPF38A	199	0.362
ENSP00000285379	CA2	6	0.362
ENSP00000301280	CHAF1A	938	0.362
ENSP00000302620	AGXT	134	0.363
ENSP00000264998	TF	106	0.364
ENSP00000265517	MTTP	1	0.365
ENSP00000334448	GNG2	253	0.365
ENSP00000320171	PKM	133	0.367
ENSP00000351896	TRAPPC4	134	0.369

ENSP00000287727	ZFYVE9	642	0.37
ENSP00000338964	GGT7	1	0.371
ENSP00000255764	MED10	133	0.372
ENSP00000405934	ITPR1	268	0.375
ENSP00000316377	DLGAP1	260	0.376
ENSP00000362649	HDAC1	2284	0.376
ENSP00000196551	RPS5	3	0.377
ENSP00000360154	OCRL	132	0.377
ENSP00000291554	CRYAA	9	0.378
ENSP00000360286	RAE1	268	0.379
ENSP00000377262	SRPK2	38	0.379
ENSP00000274335	PIK3R1	1659	0.386
ENSP00000353154	NFASC	5	0.386
ENSP00000007708	PDK2	129	0.387
ENSP00000273047	RAB5A	154	0.387
ENSP00000284981	APP	1457	0.389
ENSP00000262193	PSMB1	3	0.395
ENSP00000269300	PIK3R5	240	0.396
ENSP00000263918	STRN	5	0.4
ENSP00000293272	CCL5	169	0.4
ENSP00000310040	EIF3F	31	0.4
ENSP00000352980	HIST1H4A	533	0.4

ENSP00000361850	PLAU	131	0.4
ENSP00000346236	DDX46	38	0.402
ENSP00000290663	MED8	133	0.403
ENSP00000307188	ASL	1	0.406
ENSP00000265023	KNG1	827	0.409
ENSP00000309845	HRAS	1327	0.412
ENSP00000345206	RBPJ	268	0.415
ENSP00000288135	KIT	133	0.416
ENSP00000297185	HSPA9	393	0.416
ENSP00000350275	HIST1H3A	333	0.421
ENSP00000317714	STX4	477	0.423
ENSP00000356918	STX7	209	0.423
ENSP00000368438	PCNA	5124	0.423
ENSP00000270142	SOD1	134	0.429
ENSP00000299421	ILK	287	0.429
ENSP00000362441	ATRX	134	0.43
ENSP00000324729	SAV1	20	0.431
ENSP00000283635	CD8A	25	0.433
ENSP00000238081	YWHAQ	1	0.435
ENSP00000301788	POLR2G	6	0.436
ENSP00000334564	POLR3C	134	0.436
ENSP00000263864	VAMP8	209	0.44

ENSP00000257770	NT5E	2	0.441
ENSP00000355759	PARP1	268	0.441
ENSP00000226279	CD38	2	0.443
ENSP00000366395	SYVN1	127	0.444
ENSP00000225983	HDAC5	3	0.445
ENSP00000354791	DCTN1	134	0.445
ENSP00000381098	GRIP1	133	0.445
ENSP00000341551	SMAD4	1002	0.446
ENSP00000317159	CYC1	399	0.447
ENSP00000218388	TIMP1	9	0.448
ENSP00000262768	TIMP2	1	0.448
ENSP00000300161	YWHAB	171	0.449
ENSP00000352121	PIK3CG	240	0.45
ENSP00000254719	RPA1	130	0.451
ENSP00000319501	UGDH	1	0.451
ENSP00000359727	BAG2	427	0.451
ENSP00000231572	RARS	1	0.452
ENSP00000264414	CUL3	268	0.452
ENSP00000276682	EIF3H	103	0.452
ENSP00000351894	NCOA6	61	0.452
ENSP00000219476	TSC2	1015	0.454
ENSP00000419692	RXRA	441	0.456

ENSP00000316328	CIITA	6	0.459
ENSP00000266970	CDK2	2884	0.46
ENSP00000219240	DHODH	1	0.461
ENSP00000262735	PPARA	298	0.461
ENSP00000366410	NMNAT1	127	0.461
ENSP00000267163	RB1	2556	0.466
ENSP00000384053	CSF2RB	214	0.467
ENSP00000337759	DOM3Z	7	0.468
ENSP00000361021	PTEN	134	0.469
ENSP00000259512	DERL1	133	0.47
ENSP00000220849	EIF3E	101	0.471
ENSP00000299293	FRS2	1	0.472
ENSP00000370151	RAD17	15	0.473
ENSP00000221972	CD79A	131	0.474
ENSP00000263694	SNRNP40	1	0.474
ENSP00000335074	GHRL	129	0.475
ENSP00000345571	E2F1	526	0.475
ENSP00000212015	SIRT1	261	0.476
ENSP00000314520	KCNA2	1	0.476
ENSP00000228682	GLI1	14	0.477
ENSP00000299402	APBB1	853	0.479
ENSP00000417764	ALG2	134	0.479

ENSP00000367276	CKAP2	134	0.48
ENSP00000354673	CNOT4	35	0.482
ENSP00000246747	ARL2	1	0.484
ENSP00000005257	RALA	152	0.485
ENSP00000303634	LRP8	7	0.485
ENSP00000361359	CD40	133	0.485
ENSP00000317904	GYS1	395	0.486
ENSP00000350003	CCR3	22	0.486
ENSP00000264220	PPAT	2	0.489
ENSP00000361066	NCOA3	72	0.492
ENSP00000216254	ACO2	1	0.493
ENSP00000226730	IL2	135	0.494
ENSP00000314214	VAMP2	475	0.494
ENSP00000384675	SOS1	228	0.495
ENSP00000296585	ITGA2	395	0.496
ENSP00000244051	MOCS3	3	0.498
ENSP00000337825	LCK	2603	0.498
ENSP00000291700	S100B	134	0.5
ENSP00000310170	FOSL1	131	0.5
ENSP00000290649	AMFR	133	0.502
ENSP00000262340	RPE65	1	0.503
ENSP00000309166	RBM4	8	0.504

ENSP00000232607	UMPS	1	0.505
ENSP00000349437	IGF2R	145	0.505
ENSP00000260970	PPIG	199	0.507
ENSP00000338297	IGF2	145	0.508
ENSP00000355518	FH	4	0.509
ENSP00000011653	CD4	1797	0.51
ENSP00000396219	MEF2C	39	0.51
ENSP00000274255	SKP2	1292	0.513
ENSP00000379110	CXCL1	132	0.513
ENSP00000300738	RRM1	9	0.516
ENSP00000259469	RPL35	268	0.517
ENSP00000172229	NGFR	272	0.518
ENSP00000222673	OGDH	2	0.519
ENSP00000256010	NTS	1	0.519
ENSP00000322542	GTF2I	6	0.519
ENSP00000334122	FGF3	132	0.519
ENSP00000344936	PTTG1	132	0.52
ENSP00000383199	NEDD4L	133	0.521
ENSP00000252487	TOMM40	8	0.524
ENSP00000337040	UNC119	1	0.525
ENSP00000417404	HFE	106	0.525
ENSP00000304669	CTNNA1	134	0.526

ENSP00000354586	GLI2	11	0.527
ENSP00000410732	GABRG2	1	0.528
ENSP00000356425	UCHL5	134	0.534
ENSP00000085219	CD22	10	0.539
ENSP00000224337	BLNK	131	0.539
ENSP00000276571	CRH	9	0.54
ENSP00000343745	DICER1	37	0.54
ENSP00000350283	BRCA1	2524	0.544
ENSP00000356248	PTPN7	131	0.544
ENSP00000342793	PLD1	152	0.545
ENSP00000352798	COL18A1	1	0.545
ENSP00000265056	MCM2	4	0.546
ENSP00000215832	MAPK1	1133	0.548
ENSP00000244007	PLCG1	1556	0.548
ENSP00000381717	UBE2D2	35	0.548
ENSP00000264832	ICAM1	3	0.549
ENSP00000262633	RBM42	134	0.55
ENSP00000264705	CAD	2	0.55
ENSP00000228916	SCNN1A	133	0.554
ENSP00000261023	ITGAV	83	0.554
ENSP00000229022	VDR	569	0.555
ENSP00000369055	B4GALT1	1	0.556

ENSP00000352514	RUNX2	134	0.557
ENSP00000303088	POLR3D	268	0.558
ENSP00000291552	U2AF1	197	0.559
ENSP00000242577	DYNLL1	752	0.562
ENSP00000372793	LTA	75	0.562
ENSP00000355493	ADSS	3	0.564
ENSP00000265171	EGF	570	0.566
ENSP00000351665	CLIP1	134	0.566
ENSP00000357858	BUB3	1807	0.566
ENSP00000380280	FGFR1	931	0.566
ENSP00000052754	DCN	399	0.567
ENSP00000348442	PSMD12	101	0.568
ENSP00000252945	CYP2E1	133	0.57
ENSP00000379204	BMP7	1	0.572
ENSP00000281708	FBXW7	181	0.576
ENSP00000287497	ITGAM	134	0.576
ENSP00000305790	SF3B3	1	0.579
ENSP00000220592	AGO2	37	0.585
ENSP00000390500	STK3	20	0.585
ENSP00000244289	LIPE	7	0.586
ENSP00000350941	SRC	5013	0.59
ENSP00000314004	ANAPC2	31	0.592

ENSP00000358918	SUFU	2	0.592
ENSP00000397552	ACTL6A	54	0.593
ENSP00000267415	TINF2	99	0.595
ENSP00000378529	FZR1	80	0.595
ENSP00000365439	HNRNPK	3431	0.596
ENSP00000253004	ASS1	3	0.598
ENSP00000367910	FANCG	130	0.598
ENSP00000382004	CTNND1	196	0.598
ENSP00000046794	LCP2	230	0.603
ENSP00000235090	WDR77	1624	0.603
ENSP00000369497	BRCA2	242	0.604
ENSP00000222256	RAB3A	268	0.606
ENSP00000228872	CDKN1B	748	0.606
ENSP00000350512	COPS5	543	0.607
ENSP00000313829	KHDRBS1	3099	0.61
ENSP00000216194	ADSL	5	0.611
ENSP00000264606	HDAC4	10	0.613
ENSP00000264818	TYK2	101	0.613
ENSP00000363641	TXN	133	0.614
ENSP00000302269	VAV1	562	0.615
ENSP00000223129	RPA3	4	0.616
ENSP00000298139	WRN	18	0.616

ENSP00000306512	IL8	583	0.616
ENSP00000372860	VARS2	2	0.618
ENSP00000294172	NXF1	580	0.619
ENSP00000215587	POLR2E	406	0.621
ENSP00000342056	CS	3	0.627
ENSP00000355153	CDKN2A	138	0.635
ENSP00000217244	CSNK2A1	134	0.636
ENSP00000262160	SMAD2	903	0.638
ENSP00000354476	SREBF2	268	0.638
ENSP00000382595	PAICS	5	0.639
ENSP00000391349	DOM3Z	58	0.644
ENSP00000219252	POLR2C	133	0.646
ENSP00000316176	UBE2N	130	0.65
ENSP00000010338	TRAF3IP3	3	0.651
ENSP00000260762	EXOC6	6	0.651
ENSP00000335304	DLST	3	0.653
ENSP00000352400	NUP214	568	0.656
ENSP00000263754	KAT2B	131	0.659
ENSP00000366013	GNB2L1	153	0.66
ENSP00000262056	EIF4B	1	0.661
ENSP00000367797	SKI	4	0.662
ENSP00000270474	PDE4A	2	0.663

ENSP00000271628	SF3B4	18	0.664
ENSP00000268058	PML	247	0.668
ENSP00000355325	PSMB5	131	0.669
ENSP00000217958	PSMD10	132	0.67
ENSP00000358716	DDX20	37	0.671
ENSP00000360141	GNAS	5	0.671
ENSP00000352929	CSNK1E	3	0.678
ENSP00000248566	SHFM1	2641	0.679
ENSP00000307288	MCM7	3	0.68
ENSP00000361418	IPO13	197	0.68
ENSP00000261205	SYT1	307	0.682
ENSP00000419851	GMPS	134	0.685
ENSP00000352138	KIRREL	163	0.688
ENSP00000356087	IKBKE	78	0.688
ENSP00000389934	EXOC5	7	0.688
ENSP00000251810	RRM2B	9	0.689
ENSP00000308533	GEMIN2	37	0.689
ENSP00000313420	PRKDC	475	0.689
ENSP00000367872	GNB1	2	0.694
ENSP00000306866	GABARAP	1	0.695
ENSP00000354554	MT-CYB	531	0.698
ENSP00000251849	RAF1	1630	0.7

ENSP00000358812	PDCD11	2	0.7
ENSP00000237014	TTR	7	0.701
ENSP00000308176	BTK	4	0.701
ENSP00000381064	INTS10	22	0.708
ENSP00000262238	YY1	234	0.711
ENSP00000354961	MT-ND4	530	0.711
ENSP00000311032	CASP3	139	0.712
ENSP00000368699	ISG15	134	0.712
ENSP00000416097	GOLGA2	242	0.712
ENSP00000413493	CPSF3L	2	0.713
ENSP00000355261	SMG5	3	0.714
ENSP00000298316	ARF6	545	0.718
ENSP00000310127	IRF3	98	0.718
ENSP00000162749	TNFRSF1A	344	0.725
ENSP00000211998	VCL	9	0.725
ENSP00000261461	PPP2R5A	152	0.729
ENSP00000384708	FSHR	2	0.729
ENSP00000348554	CDC16	49	0.731
ENSP00000324804	PPP2R1A	77	0.732
ENSP00000307863	U2AF2	2171	0.736
ENSP00000265709	ANK1	4	0.738
ENSP00000330237	CASP9	3	0.738

ENSP00000254122	FSHB	2	0.74
ENSP00000314458	CDC42	1011	0.74
ENSP00000327070	MDH2	9	0.74
ENSP00000354876	MT-CO2	133	0.742
ENSP00000378338	GIT1	1	0.742
ENSP00000411698	USO1	133	0.743
ENSP00000391592	PTPN6	110	0.744
ENSP00000311113	JUP	4	0.75
ENSP00000252486	APOE	16	0.751
ENSP00000362592	RBBP4	282	0.754
ENSP00000227524	PRPF19	77	0.758
ENSP00000225577	RPS6KB1	14	0.761
ENSP00000381331	HDAC2	222	0.762
ENSP00000312262	ADRBK1	12	0.763
ENSP00000205402	DLD	103	0.764
ENSP00000266000	DAXX	134	0.768
ENSP00000318861	SF3B2	34	0.768
ENSP00000356587	NPHS2	163	0.768
ENSP00000248553	HSPB1	6	0.773
ENSP00000358541	SIKE1	66	0.773
ENSP00000396127	RAN	1084	0.775
ENSP00000304845	UGT1A1	1	0.776

ENSP00000285814	MKI67IP	1	0.777
ENSP00000288986	NCK1	138	0.777
ENSP00000362361	CDK9	268	0.779
ENSP00000262803	UPF1	137	0.781
ENSP00000348577	RANGAP1	1084	0.793
ENSP00000230449	EXOC2	7	0.794
ENSP00000249299	NAA38	57	0.798
ENSP00000316879	EIF4G1	513	0.798
ENSP00000297494	NOS3	1634	0.801
ENSP00000265564	EXOSC7	1	0.804
ENSP00000215071	PSMD8	1	0.807
ENSP00000256474	VHL	3738	0.812
ENSP00000269321	ARHGDIA	177	0.813
ENSP00000292644	PSMC2	3862	0.814
ENSP00000349467	CALM1	1495	0.815
ENSP00000351777	VCP	260	0.815
ENSP00000300413	SNRPD1	37	0.818
ENSP00000338983	MUC1	423	0.819
ENSP00000366396	XRN2	45	0.819
ENSP00000223095	SERPINE1	147	0.822
ENSP00000254940	NIP7	133	0.822
ENSP00000369213	DDX58	134	0.823

ENSP00000359074	L1CAM	1	0.825
ENSP00000289779	F11R	14	0.829
ENSP00000347858	XIAP	3	0.829
ENSP00000379933	TPI1	129	0.829
ENSP00000348877	GPI	129	0.831
ENSP00000284811	TCEB1	153	0.833
ENSP00000335153	HSP90AA1	3615	0.834
ENSP00000245414	IRF1	10	0.835
ENSP00000326804	CUL1	64	0.836
ENSP00000346437	ATG7	1	0.837
ENSP00000274459	ATG12	1	0.839
ENSP00000219070	MMP2	1	0.842
ENSP00000312435	DAG1	134	0.846
ENSP00000384273	RELA	699	0.846
ENSP00000253856	ATP6V0A4	278	0.847
ENSP00000343054	RBM5	113	0.848
ENSP00000400175	RHOA	134	0.851
ENSP00000358997	IRAK1	268	0.853
ENSP00000367207	MYC	1178	0.855
ENSP00000221930	TGFB1	399	0.857
ENSP00000295767	CHCHD4	8	0.859
ENSP00000221494	SF3A2	1867	0.86

ENSP00000352264	CD2AP	163	0.86
ENSP00000227758	BIRC2	133	0.861
ENSP00000252444	LDLR	11	0.862
ENSP00000353452	MYLK	134	0.863
ENSP00000374354	EXOSC8	157	0.865
ENSP00000302967	HDAC3	129	0.866
ENSP00000355890	EPRS	1	0.866
ENSP00000264279	NOP58	133	0.87
ENSP00000225916	KAT2A	2	0.872
ENSP00000324897	UBE2I	1358	0.872
ENSP00000262435	SMURF2	3	0.873
ENSP00000348551	NCOR2	20	0.873
ENSP00000309103	BAD	31	0.874
ENSP00000401980	MAVS	133	0.874
ENSP00000262158	SMAD7	334	0.875
ENSP00000346022	RPL9	134	0.875
ENSP00000343535	USP7	134	0.878
ENSP00000371236	GART	7	0.879
ENSP00000356438	PTGS2	266	0.88
ENSP00000358595	CGA	2	0.884
ENSP00000404121	ILF3	96	0.887
ENSP00000351908	MAP3K5	134	0.89

ENSP00000247668	TRAF2	983	0.893
ENSP00000304592	FASN	134	0.893
ENSP00000368880	FOXO1	2	0.893
ENSP00000254942	TERF2	99	0.901
ENSP00000332468	TRAF3	132	0.901
ENSP00000229854	MCM3	3	0.902
ENSP00000248114	GFER	8	0.902
ENSP00000216225	RBX1	576	0.903
ENSP00000303242	ITGB2	131	0.908
ENSP00000278916	CHEK1	26	0.909
ENSP00000312735	POLR2B	672	0.916
ENSP00000230354	TBP	524	0.918
ENSP00000301764	DDB1	120	0.921
ENSP00000356070	MAPKAPK2	10	0.923
ENSP00000216605	MTHFD1	7	0.925
ENSP00000314949	POLR2A	449	0.928
ENSP00000229239	GAPDH	129	0.929
ENSP00000308541	F2	256	0.929
ENSP00000310596	LSM1	54	0.939
ENSP00000265335	RAD50	93	0.944
ENSP00000348307	SIRPA	14	0.947
ENSP00000325448	KARS	1	0.95

ENSP00000276201	UPF3B	152	0.954
ENSP00000374455	SQSTM1	125	0.954
ENSP00000268182	IQGAP1	72	0.958
ENSP00000364133	TGFBR1	385	0.958
ENSP00000418447	PPP2CA	570	0.96
ENSP00000363921	PARD3	12	0.966
ENSP00000252102	NDUFA2	804	0.967
ENSP00000269349	EIF4A3	117	0.967
ENSP00000359345	RPL5	268	0.968
ENSP00000264951	XRN1	735	0.969
ENSP00000329623	BCL2	270	0.971
ENSP00000358563	DKC1	267	0.971
ENSP00000348461	RAC1	1213	0.973
ENSP00000342374	SNRPD2	7	0.974
ENSP00000362820	SRSF3	4	0.976
ENSP00000329380	GP1BA	255	0.977
ENSP00000344818	UBC	55873	0.98
ENSP00000215829	SNRPD3	617	0.981
ENSP00000369757	RPS6	1	0.981
ENSP00000219255	PARD6A	21	0.984
ENSP00000295897	ALB	682	0.984
ENSP00000309503	YWHAZ	284	0.985

ENSP00000245960	CDC25B	51	0.986
ENSP00000319169	PRMT5	529	0.986
ENSP00000348708	UPF2	142	0.988
ENSP00000350877	SRSF2	4	0.989
ENSP00000366135	EXOSC10	1775	0.989
ENSP00000240185	TARDBP	1837	0.99
ENSP00000363676	RPL11	524	0.991
ENSP00000333001	RBM8A	2	0.992
ENSP00000361626	YBX1	8	0.993
ENSP00000414634	LSM2	103	0.993
ENSP00000252622	LSM7	299	0.994
ENSP00000398597	EXOSC6	72	0.994
ENSP00000270202	AKT1	5225	0.996
ENSP00000296271	RHO	1059	0.997
ENSP00000377141	ARRB1	1044	0.997
ENSP00000263309	CLNS1A	394	1
ENSP00000417281	MDM2	2155	1

2. Candidate genes for methylation CpG site genes and somatic mutation genes

Ensembl ID	Gene symbol	Betweenness	Permutation FDR
ENSP00000075120	SLC2A3	191	<0.001
ENSP00000204615	THPO	191	<0.001
ENSP00000221855	TBCB	2	<0.001

ENSP00000222725	LFNG	382	<0.001
ENSP00000248272	GAN	2	<0.001
ENSP00000252818	JUND	1567	<0.001
ENSP00000253571	RLIM	191	<0.001
ENSP00000256151	CCDC59	191	<0.001
ENSP00000257829	NAT10	143	<0.001
ENSP00000290295	HOXB13	191	<0.001
ENSP00000293362	PSME3	191	<0.001
ENSP00000294309	TPCN2	2	<0.001
ENSP00000295600	MITF	191	<0.001
ENSP00000297261	SHH	706	<0.001
ENSP00000315997	LILRB1	191	<0.001
ENSP00000325123	ZSCAN2	2	<0.001
ENSP00000328364	MAFA	191	<0.001
ENSP00000330633	CNTN2	256	<0.001
ENSP00000332353	PTCH1	931	<0.001
ENSP00000333188	FOXL2	191	<0.001
ENSP00000334044	UBL4B	1	<0.001
ENSP00000335038	VSTM2B	1	<0.001
ENSP00000337088	MEN1	1567	<0.001
ENSP00000344456	CTNNB1	3240	<0.001
ENSP00000351209	EPHA2	640	<0.001

ENSP00000352262	MLL	1581	<0.001
ENSP00000355140	HOXB1	191	<0.001
ENSP00000358497	RNGTT	191	<0.001
ENSP00000364946	MKX	2	<0.001
ENSP00000373700	ALK	191	<0.001
ENSP00000376849	CASP5	191	<0.001
ENSP00000401435	VPS53	191	<0.001
ENSP00000403005	EFNA4	382	<0.001
ENSP00000405890	PBX1	573	<0.001
ENSP00000020926	SYT13	1	0.001
ENSP00000247843	YEATS4	191	0.001
ENSP00000257068	MTNR1B	8	0.001
ENSP00000291842	SHKBP1	191	0.001
ENSP00000296585	ITGA2	792	0.001
ENSP00000319118	GSX2	191	0.001
ENSP00000359215	TLX1	191	0.001
ENSP00000360076	SGIP1	190	0.001
ENSP00000361548	MPL	191	0.001
ENSP00000375629	LILRB2	191	0.001
ENSP00000400223	FJX1	1	0.001
ENSP00000254351	SDC1	199	0.002
ENSP00000262965	TCF3	571	0.002

ENSP00000287934	FZD1	382	0.002
ENSP00000304669	CTNNA1	536	0.002
ENSP00000306245	FOS	1790	0.002
ENSP00000307479	ARNT2	191	0.002
ENSP00000331791	TBX1	191	0.002
ENSP00000341550	SLC24A5	1	0.002
ENSP00000348815	HYLS1	191	0.002
ENSP00000357656	FYN	1799	0.002
ENSP00000358525	NGF	814	0.002
ENSP00000365682	TLE1	382	0.002
ENSP00000371138	FKBP1A	530	0.002
ENSP00000388996	AP1M1	191	0.002
ENSP00000162330	BCAR1	485	0.003
ENSP00000297268	COL1A2	400	0.003
ENSP00000322898	EBF1	268	0.003
ENSP00000331057	TCF12	191	0.003
ENSP00000363827	HSPG2	191	0.003
ENSP00000401303	SHC1	976	0.003
ENSP00000229030	FZD10	191	0.004
ENSP00000269280	NLRP1	191	0.004
ENSP00000286621	ADK	134	0.004
ENSP00000312435	DAG1	666	0.004

ENSP00000314897	ANGPT2	191	0.004
ENSP00000354541	NLGN1	315	0.004
ENSP00000359074	L1CAM	527	0.004
ENSP00000359245	ABCA4	1	0.004
ENSP00000366347	NKX2-2	191	0.004
ENSP00000370521	AIPL1	190	0.004
ENSP00000387662	GCG	918	0.004
ENSP00000241125	GJA3	183	0.005
ENSP00000252456	CNN1	134	0.005
ENSP00000304915	IL13	191	0.005
ENSP00000318057	EGR3	190	0.005
ENSP00000364092	ASIP	186	0.005
ENSP00000370256	FOXC1	188	0.005
ENSP00000372313	MSLN	134	0.005
ENSP00000260130	SDCBP	198	0.006
ENSP00000331358	GAST	419	0.006
ENSP00000339328	PLAUR	382	0.006
ENSP00000341170	PTN	135	0.006
ENSP00000344460	CBS	188	0.006
ENSP00000351486	NTRK1	913	0.006
ENSP00000383210	NEK3	134	0.006
ENSP00000265709	ANK1	398	0.007

ENSP00000293379	ITGA5	1098	0.007
ENSP00000328181	NOG	191	0.007
ENSP00000349275	NRG1	191	0.007
ENSP00000354111	DNAJC5	183	0.007
ENSP00000265335	RAD50	570	0.008
ENSP00000298552	TSC1	187	0.008
ENSP00000303522	TACR1	134	0.008
ENSP00000355961	INTS7	191	0.008
ENSP00000371308	CENPJ	191	0.008
ENSP00000265071	CDH6	134	0.009
ENSP00000281708	FBXW7	222	0.009
ENSP00000304414	CXCR6	190	0.009
ENSP00000330032	UPP1	1	0.009
ENSP00000345487	QRFP	85	0.009
ENSP00000348634	MYH6	134	0.009
ENSP00000352657	ME3	134	0.009
ENSP00000398644	NUB1	190	0.009
ENSP00000200181	ITGB4	313	0.01
ENSP00000303634	LRP8	14	0.01
ENSP00000362555	RNF19B	2	0.01
ENSP00000385450	MAGI1	134	0.01
ENSP00000392466	LDB1	191	0.01

ENSP00000405041	POU5F1	191	0.01
ENSP00000228307	PXN	347	0.011
ENSP00000229307	NANOG	192	0.011
ENSP00000268171	FURIN	268	0.011
ENSP00000358799	RBM15	190	0.011
ENSP00000372160	DOK6	134	0.011
ENSP00000382193	MYBPC3	135	0.012
ENSP00000242839	ATP7B	134	0.013
ENSP00000263856	CHMP3	1	0.013
ENSP00000274711	LRRTM2	1	0.013
ENSP00000341032	WNT7B	191	0.013
ENSP00000361125	VEGFA	728	0.013
ENSP00000363360	INIP	134	0.013
ENSP00000379457	FAF1	134	0.013
ENSP00000397181	RGS4	1	0.013
ENSP00000225893	HNF1B	134	0.014
ENSP00000251337	GNAT2	191	0.014
ENSP00000236192	VAMP4	191	0.015
ENSP00000309595	C10orf2	134	0.015
ENSP00000336666	AP1S1	40	0.015
ENSP00000401018	GINS3	189	0.015
ENSP00000222399	LAMB1	131	0.016

ENSP00000254950	VPS4A	320	0.016
ENSP00000262053	ATF1	134	0.016
ENSP00000276603	TERF1	264	0.016
ENSP00000296785	ANKRA2	14	0.016
ENSP00000310440	CHMP2A	189	0.016
ENSP00000344468	SDC3	134	0.016
ENSP00000367486	MEIG1	134	0.016
ENSP00000400258	DLGAP2	1	0.016
ENSP00000417132	BAP1	191	0.016
ENSP00000233813	IGFBP5	134	0.017
ENSP00000302665	IGF1	1010	0.017
ENSP00000355180	COL6A1	1	0.017
ENSP00000360882	COL5A1	2	0.017
ENSP00000369654	HBD	1	0.017
ENSP00000254942	TERF2	456	0.018
ENSP00000340698	GIPC1	253	0.018
ENSP00000367714	HES5	134	0.018
ENSP00000386896	ITGA6	317	0.018
ENSP00000312185	ELMO1	134	0.019
ENSP00000410294	FGFR2	191	0.019
ENSP00000257818	LMO2	195	0.02
ENSP00000312652	LEP	1064	0.02

ENSP00000293288	BAX	272	0.021
ENSP00000293897	SSTR5	3	0.021
ENSP00000356641	RFWD2	133	0.021
ENSP00000419361	ADCY5	4	0.021
ENSP00000248933	SEZ6L	1	0.022
ENSP00000261769	CDH1	505	0.022
ENSP00000277541	NOTCH1	1128	0.022
ENSP00000360200	INADL	133	0.022
ENSP00000346879	NKX2-1	191	0.023
ENSP00000006053	CX3CL1	186	0.024
ENSP00000357283	LMNA	191	0.024
ENSP00000257254	APLNR	8	0.025
ENSP00000299413	TRIM44	1	0.025
ENSP00000333769	BSG	191	0.025
ENSP00000361850	PLAU	261	0.025
ENSP00000265362	SEMA3A	134	0.026
ENSP00000250448	FOXA1	191	0.027
ENSP00000263431	PRKCG	183	0.027
ENSP00000265773	SMARCA2	192	0.027
ENSP00000380066	MAP4K1	188	0.027
ENSP00000222982	CYP3A5	1	0.028
ENSP00000260570	IFT172	7	0.028

ENSP00000262968	TJP3	2	0.028
ENSP00000263281	GIPR	3	0.028
ENSP00000301838	FADD	321	0.028
ENSP00000340858	B2M	953	0.028
ENSP00000372547	SRY	134	0.028
ENSP00000295709	STK36	133	0.029
ENSP00000346155	UCKL1	2	0.029
ENSP00000291525	TFF3	133	0.03
ENSP00000338785	STARD13	122	0.03
ENSP00000344822	S100A13	3	0.03
ENSP00000382166	CX3CR1	186	0.03
ENSP00000264657	STAT3	1898	0.031
ENSP00000310244	RASGRP1	12	0.031
ENSP00000332973	SMAD3	456	0.031
ENSP00000348168	GTF2E2	131	0.031
ENSP00000361311	TMEM53	1	0.031
ENSP00000370408	CDX2	134	0.031
ENSP00000254480	SMARCC1	292	0.032
ENSP00000344742	STAMBP	134	0.033
ENSP00000267845	HDC	131	0.034
ENSP00000407431	HLA-C	874	0.034
ENSP00000264039	GPC1	1	0.035

ENSP00000258341	LAMC1	134	0.036
ENSP00000350941	SRC	1965	0.036
ENSP00000356694	FASLG	190	0.036
ENSP00000361759	BEX2	5	0.036
ENSP00000338018	HIF1A	2624	0.037
ENSP00000361777	SET	134	0.037
ENSP00000245323	EFNB2	134	0.038
ENSP00000264708	POMC	511	0.038
ENSP00000308176	BTK	171	0.038
ENSP00000356162	KISS1	85	0.038
ENSP00000360689	TNKS2	132	0.038
ENSP00000411286	GABBR1	1	0.038
ENSP00000227507	CCND1	1813	0.039
ENSP00000304188	OR8U1	2	0.039
ENSP00000242577	DYNLL1	544	0.04
ENSP00000264033	CBL	4975	0.04
ENSP00000313921	MSRA	1	0.04
ENSP00000300134	STAT6	431	0.041
ENSP00000300574	CRK	436	0.041
ENSP00000380349	CAPN3	134	0.041
ENSP00000260356	THBS1	22	0.042
ENSP00000264637	THRA	43	0.042

ENSP00000303325	TACR3	3	0.042
ENSP00000337397	DKFZP686J19100	134	0.042
ENSP00000348089	ERCC6	129	0.042
ENSP00000267415	TINF2	195	0.043
ENSP00000305464	APLN	1	0.043
ENSP00000308938	PLG	766	0.044
ENSP00000343479	NBR1	134	0.044
ENSP00000019103	SCTR	1	0.045
ENSP00000261681	MPP5	131	0.045
ENSP00000312697	DMAP1	191	0.045
ENSP00000339861	ENY2	133	0.045
ENSP00000206249	ESR1	3113	0.046
ENSP00000258301	STX6	191	0.046
ENSP00000351407	ARNT	560	0.046
ENSP00000219473	USP10	48	0.047
ENSP00000276420	DOK2	54	0.047
ENSP00000326630	ZFPM1	2	0.048
ENSP00000364839	ASXL1	8	0.048
ENSP00000171214	RDH8	1	0.049
ENSP00000377958	CCT4	134	0.05
ENSP00000334458	GATA4	193	0.051
ENSP00000347197	C5AR1	1	0.051

ENSP00000362403	TACR2	1	0.051
ENSP00000332592	SPAG16	134	0.052
ENSP00000366563	PIK3CD	132	0.052
ENSP00000374372	SPTB	134	0.053
ENSP00000308208	MMP14	2	0.054
ENSP00000333194	RGS19	25	0.054
ENSP00000363435	ITPR3	18	0.054
ENSP00000331327	WT1	134	0.055
ENSP00000369129	DSP	134	0.055
ENSP00000266376	CACNA1C	137	0.056
ENSP00000342215	KIR2DL3	874	0.057
ENSP00000345656	VAPA	134	0.057
ENSP00000358022	MCL1	522	0.057
ENSP00000377148	AP1G1	151	0.057
ENSP00000225831	CCL2	185	0.058
ENSP00000290039	CACHD1	1	0.058
ENSP00000320401	UGT2B17	1	0.058
ENSP00000346659	OCA2	2	0.058
ENSP00000358323	TXNIP	134	0.058
ENSP00000360310	SPO11	133	0.058
ENSP00000301200	CDC42EP5	133	0.059
ENSP00000318641	INTS3	134	0.059

ENSP00000261366	LMNB1	195	0.06
ENSP00000328777	EFNA5	136	0.06
ENSP00000361965	ADA	128	0.06
ENSP00000316854	ATOX1	134	0.061
ENSP00000380227	ITGA4	292	0.061
ENSP00000343313	ATG5	134	0.062
ENSP00000359297	NSDHL	133	0.062
ENSP00000362058	NDUFS5	134	0.062
ENSP00000252034	ELN	134	0.063
ENSP00000366746	STAM	133	0.063
ENSP00000222005	CDC37	323	0.064
ENSP00000262188	SMARCD3	122	0.064
ENSP00000338934	EZR	857	0.064
ENSP00000263253	EP300	1711	0.065
ENSP00000247161	ELK1	13	0.066
ENSP00000170630	IL4R	191	0.067
ENSP00000286548	GNAQ	126	0.067
ENSP00000322542	GTF2I	134	0.067
ENSP00000349960	ACTB	135	0.067
ENSP00000253024	TRIM28	191	0.068
ENSP00000358994	MYO6	133	0.068
ENSP00000359506	FMR1	134	0.068

ENSP00000223642	C5	134	0.069
ENSP00000265354	SRF	325	0.069
ENSP00000354532	PNP	1	0.069
ENSP00000354901	CXCL9	186	0.069
ENSP00000305422	CEBPB	19	0.07
ENSP00000265171	EGF	364	0.071
ENSP00000316460	FYB	134	0.072
ENSP00000417864	ANP32A	134	0.072
ENSP00000410076	CASP1	191	0.073
ENSP00000005279	SYNRG	151	0.074
ENSP00000310448	SART1	134	0.074
ENSP00000349204	CRB3	131	0.074
ENSP00000377941	ACTN1	134	0.074
ENSP00000335657	CCK	103	0.077
ENSP00000333275	NR2C1	24	0.078
ENSP00000225698	C1QBP	134	0.079
ENSP00000281821	EPHA4	2	0.079
ENSP00000370473	IGFBP3	727	0.08
ENSP00000226218	SEBOX	51	0.081
ENSP00000262186	KCNH2	191	0.081
ENSP00000291688	MCM3AP	133	0.081
ENSP00000353224	TFRC	60	0.081

ENSP00000390849	ABHD5	133	0.082
ENSP00000256443	CDK7	263	0.084
ENSP00000264998	TF	60	0.084
ENSP00000371512	SGCZ	133	0.085
ENSP00000228841	MYL2	133	0.086
ENSP00000324549	CYFIP1	134	0.086
ENSP00000351926	AP2A1	189	0.086
ENSP00000261507	MSMO1	133	0.087
ENSP00000347719	TBCD	3	0.087
ENSP00000273588	AMT	2	0.089
ENSP00000357292	UBQLN4	136	0.089
ENSP00000364133	TGFBR1	656	0.089
ENSP00000384015	SUN1	2	0.089
ENSP00000257430	APC	134	0.09
ENSP00000262105	MCM4	187	0.09
ENSP00000262613	SLC9A3R1	851	0.09
ENSP00000335544	CCKBR	101	0.09
ENSP00000346294	S100A4	135	0.09
ENSP00000283875	GTF2E1	131	0.091
ENSP00000313391	DAB2	128	0.091
ENSP00000352514	RUNX2	267	0.091
ENSP00000242152	NPY	190	0.092

ENSP00000319060	CAMK2G	134	0.093
ENSP00000263967	PIK3CA	132	0.094
ENSP00000345206	RBPJ	264	0.094
ENSP00000364336	TBXA2R	2	0.094
ENSP00000351894	NCOA6	135	0.098
ENSP00000265023	KNG1	420	0.099
ENSP00000354586	GLI2	139	0.099
ENSP00000157812	PSMC4	188	0.1
ENSP00000356602	VTA1	133	0.1
ENSP00000372975	HLA-C	194	0.101
ENSP00000347198	SRGAP1	133	0.103
ENSP00000382723	AGPAT1	1	0.103
ENSP00000318472	NCAM1	191	0.105
ENSP00000357392	EFNA1	132	0.105
ENSP00000368683	EDN1	188	0.108
ENSP00000282588	ITGA1	3	0.11
ENSP00000300055	PLIN1	133	0.112
ENSP00000271620	PRUNE	1	0.114
ENSP00000223023	WASL	460	0.115
ENSP00000228837	FGF6	190	0.115
ENSP00000249075	LIF	190	0.116
ENSP00000308021	CEP290	134	0.118

ENSP00000380378	PAFAH1B1	2	0.119
ENSP00000259808	RIPK1	320	0.12
ENSP00000361423	ABL1	343	0.12
ENSP00000263025	MAPK3	13	0.121
ENSP00000309629	CFL1	3	0.121
ENSP00000363021	RPA2	132	0.123
ENSP00000354251	NCKAP1	134	0.124
ENSP00000001008	FKBP4	134	0.125
ENSP00000219476	TSC2	424	0.126
ENSP00000264515	RBBP5	9	0.127
ENSP00000351446	WDR5	316	0.128
ENSP00000262367	CREBBP	1199	0.129
ENSP00000353731	DPP4	128	0.131
ENSP00000262768	TIMP2	2	0.132
ENSP00000297518	CDK5	331	0.132
ENSP00000375809	ERCC2	132	0.132
ENSP00000244741	CDKN1A	202	0.133
ENSP00000383623	MLLT4	190	0.134
ENSP00000225655	PFN1	48	0.135
ENSP00000249647	SNAP23	191	0.135
ENSP00000219240	DHODH	1	0.136
ENSP00000263918	STRN	5	0.137

ENSP00000353154	NFASC	6	0.138
ENSP00000261531	SNW1	130	0.139
ENSP00000218388	TIMP1	6	0.14
ENSP00000291554	CRYAA	8	0.14
ENSP00000340820	MAPT	329	0.141
ENSP00000361405	MMP9	149	0.141
ENSP00000252486	APOE	19	0.142
ENSP00000283635	CD8A	10	0.145
ENSP00000312262	ADRBK1	125	0.145
ENSP00000315955	FOXA2	13	0.146
ENSP00000263923	KDR	17	0.147
ENSP00000321326	F2R	3	0.147
ENSP00000302234	CCL11	128	0.148
ENSP00000348786	RAP1A	134	0.149
ENSP00000324248	PENK	6	0.151
ENSP00000225983	HDAC5	6	0.153
ENSP00000264705	CAD	3	0.153
ENSP00000358866	FLNA	163	0.155
ENSP00000337224	LRAT	1	0.157
ENSP00000229854	MCM3	136	0.158
ENSP00000338799	IL6ST	190	0.16
ENSP00000360683	PTPN1	150	0.16

ENSP00000265371	NRP1	134	0.161
ENSP00000360916	VAV2	134	0.161
ENSP00000366006	UBIAD1	1	0.162
ENSP00000353099	HLA-DRB1	134	0.163
ENSP00000358918	SUFU	6	0.163
ENSP00000244289	LIPE	133	0.164
ENSP00000298139	WRN	19	0.164
ENSP00000355536	MTR	1	0.164
ENSP00000381064	INTS10	134	0.165
ENSP00000365775	MTHFR	1	0.166
ENSP00000394624	OPRM1	3	0.167
ENSP00000267082	ITGB7	151	0.168
ENSP00000297494	NOS3	872	0.168
ENSP00000350283	BRCA1	916	0.168
ENSP00000223095	SERPINE1	258	0.169
ENSP00000232607	UMPS	1	0.169
ENSP00000331201	HGS	133	0.171
ENSP00000413234	AP2A2	40	0.171
ENSP00000363763	EPHB2	9	0.173
ENSP00000378338	GIT1	120	0.173
ENSP00000318297	RUVBL1	190	0.18
ENSP00000243077	LRP1	13	0.181

ENSP00000363641	TXN	134	0.181
ENSP00000220584	FDFT1	133	0.184
ENSP00000250495	NEDD8	190	0.184
ENSP00000044462	PSMA4	130	0.185
ENSP00000228682	GLI1	8	0.185
ENSP00000261783	ARG2	130	0.186
ENSP00000264220	PPAT	2	0.19
ENSP00000245479	SOX9	133	0.191
ENSP00000225402	AATF	142	0.193
ENSP00000311113	JUP	136	0.193
ENSP00000309555	HCFC1	183	0.195
ENSP00000256474	VHL	1507	0.196
ENSP00000332258	DGAT1	1	0.197
ENSP00000312999	GNAI2	140	0.199
ENSP00000348986	INS-IGF2	382	0.199
ENSP00000336740	LIMK1	3	0.201
ENSP00000287766	SLC6A1	133	0.202
ENSP00000265038	ERCC8	3	0.203
ENSP00000417404	HFE	60	0.205
ENSP00000354791	DCTN1	134	0.206
ENSP00000351997	MAP2K6	113	0.207
ENSP00000355325	PSMB5	130	0.207

ENSP00000254719	RPA1	128	0.208
ENSP00000327070	MDH2	134	0.208
ENSP00000338345	SNCA	183	0.208
ENSP00000354720	SMC3	181	0.208
ENSP00000246747	ARL2	1	0.209
ENSP00000284384	PRKCA	193	0.21
ENSP00000365851	BMI1	134	0.21
ENSP00000320866	CALR	191	0.211
ENSP00000400717	GNA13	2	0.211
ENSP00000227524	PRPF19	137	0.213
ENSP00000352798	COL18A1	2	0.213
ENSP00000266970	CDK2	886	0.214
ENSP00000360798	EPS15	229	0.214
ENSP00000297338	RAD21	10	0.215
ENSP00000349467	CALM1	807	0.215
ENSP00000261023	ITGAV	47	0.219
ENSP00000302564	BCL2L1	148	0.219
ENSP00000046794	LCP2	88	0.221
ENSP00000320940	NCOA1	499	0.221
ENSP00000340330	KAT5	533	0.223
ENSP00000330237	CASP9	122	0.224
ENSP00000337040	UNC119	1	0.228

ENSP00000350003	CCR3	19	0.234
ENSP00000390427	PPIL2	4	0.236
ENSP00000276414	GNRH1	87	0.237
ENSP00000309845	HRAS	413	0.238
ENSP00000267101	ERBB3	189	0.239
ENSP00000209875	CBX5	191	0.243
ENSP00000253792	ACLY	133	0.243
ENSP00000303830	INSR	354	0.243
ENSP00000410732	GABRG2	1	0.243
ENSP00000347379	OCLN	2	0.244
ENSP00000271526	PRCC	4	0.247
ENSP00000261479	PSMA6	3	0.256
ENSP00000356671	SERPINC1	134	0.256
ENSP00000264606	HDAC4	8	0.257
ENSP00000358335	MAP3K7	113	0.257
ENSP00000265056	MCM2	4	0.26
ENSP00000300289	PDIA3	182	0.26
ENSP00000350720	SMARCA4	293	0.262
ENSP00000269397	CBX4	134	0.263
ENSP00000339992	MYB	189	0.266
ENSP00000341344	GGA1	151	0.266
ENSP00000351665	CLIP1	134	0.268

ENSP00000364893	ARHGEF7	78	0.269
ENSP00000307387	PDCD6IP	319	0.271
ENSP00000329380	GP1BA	399	0.272
ENSP00000270474	PDE4A	2	0.273
ENSP00000299402	APBB1	281	0.273
ENSP00000326031	PPP1CA	191	0.273
ENSP00000304592	FASN	267	0.274
ENSP00000332643	NDN	19	0.274
ENSP00000245206	GOT2	1	0.275
ENSP00000396219	MEF2C	8	0.275
ENSP00000314458	CDC42	480	0.276
ENSP00000340944	PTPN11	239	0.277
ENSP00000216194	ADSL	3	0.278
ENSP00000223129	RPA3	4	0.288
ENSP00000172229	NGFR	142	0.29
ENSP00000343054	RBM5	134	0.295
ENSP00000382595	PAICS	3	0.295
ENSP00000231509	NR3C1	7	0.297
ENSP00000251849	RAF1	613	0.297
ENSP00000224337	BLNK	23	0.307
ENSP00000306866	GABARAP	1	0.313
ENSP00000333982	NDEL1	2	0.314

ENSP00000288986	NCK1	134	0.325
ENSP00000262320	AXIN1	585	0.328
ENSP00000223029	AIMP2	191	0.329
ENSP00000291700	S100B	133	0.329
ENSP00000311677	PPP1R8	191	0.329
ENSP00000237014	TTR	5	0.331
ENSP00000309503	YWHAZ	412	0.331
ENSP00000326366	PSEN1	259	0.332
ENSP00000292303	CCR5	167	0.34
ENSP00000404121	ILF3	129	0.342
ENSP00000353483	MAPK8	293	0.347
ENSP00000374455	SQSTM1	220	0.347
ENSP00000329357	SP1	334	0.348
ENSP00000314004	ANAPC2	10	0.349
ENSP00000359300	CETN2	133	0.35
ENSP00000308541	F2	269	0.352
ENSP00000337759	DOM3Z	1	0.352
ENSP00000323050	RBBP8	65	0.353
ENSP00000382004	CTNND1	122	0.355
ENSP00000362900	SRSF4	4	0.359
ENSP00000262629	TYROBP	864	0.364
ENSP00000293272	CCL5	21	0.366

ENSP00000252444	LDLR	14	0.368
ENSP00000244769	ATXN1	2	0.369
ENSP00000219548	STUB1	237	0.375
ENSP00000262435	SMURF2	3	0.376
ENSP00000351273	CASP8	1	0.377
ENSP00000285021	XPC	133	0.378
ENSP00000247461	CANX	182	0.379
ENSP00000349437	IGF2R	23	0.383
ENSP00000248553	HSPB1	1	0.384
ENSP00000219255	PARD6A	131	0.386
ENSP00000229794	MAPK14	191	0.386
ENSP00000347858	XIAP	122	0.387
ENSP00000317714	STX4	134	0.388
ENSP00000380942	ARHGEF12	2	0.39
ENSP00000222256	RAB3A	134	0.396
ENSP00000285814	MKI67IP	1	0.396
ENSP00000385269	ELAVL1	1	0.398
ENSP00000257904	CDK4	153	0.402
ENSP00000342952	ADCY2	2	0.404
ENSP00000301280	CHAF1A	191	0.411
ENSP00000276571	CRH	1	0.423
ENSP00000295797	PRKCI	86	0.425

ENSP00000329623	BCL2	207	0.427
ENSP00000388526	HLA-A	5	0.429
ENSP00000278616	ATM	272	0.436
ENSP00000302967	HDAC3	119	0.436
ENSP00000011653	CD4	483	0.437
ENSP00000302961	HSPA4	25	0.437
ENSP00000346437	ATG7	1	0.437
ENSP00000364898	SYK	864	0.439
ENSP00000261205	SYT1	143	0.441
ENSP00000219070	MMP2	2	0.442
ENSP00000338297	IGF2	23	0.442
ENSP00000350275	HIST1H3A	61	0.443
ENSP00000221930	TGFB1	268	0.444
ENSP00000256996	DDB2	6	0.444
ENSP00000242057	AHR	187	0.446
ENSP00000052754	DCN	134	0.447
ENSP00000338983	MUC1	268	0.447
ENSP00000261799	PDGFRB	52	0.449
ENSP00000368104	BMP2	190	0.449
ENSP00000334448	GNG2	1	0.45
ENSP00000350708	RAD23B	127	0.453
ENSP00000378529	FZR1	22	0.455

ENSP00000351777	VCP	134	0.459
ENSP00000363822	AR	201	0.459
ENSP00000274459	ATG12	1	0.46
ENSP00000351908	MAP3K5	134	0.462
ENSP00000400591	SNRPE	3	0.467
ENSP00000262633	RBM42	1	0.469
ENSP00000251968	TSG101	281	0.482
ENSP00000245451	BMP4	1	0.483
ENSP00000354476	SREBF2	133	0.484
ENSP00000419692	RXRA	93	0.484
ENSP00000371236	GART	5	0.493
ENSP00000264414	CUL3	4	0.501
ENSP00000335153	HSP90AA1	1048	0.502
ENSP00000330393	LEPR	4	0.503
ENSP00000262238	YY1	66	0.51
ENSP00000367408	CASK	85	0.51
ENSP00000354394	STAT1	297	0.515
ENSP00000362649	HDAC1	455	0.515
ENSP00000401980	MAVS	133	0.529
ENSP00000314214	VAMP2	133	0.531
ENSP00000380280	FGFR1	238	0.536
ENSP00000326804	CUL1	22	0.542

ENSP00000332468	TRAF3	132	0.542
ENSP00000384675	SOS1	46	0.544
ENSP00000353452	MYLK	133	0.547
ENSP00000391349	DOM3Z	4	0.548
ENSP00000348551	NCOR2	9	0.55
ENSP00000302150	PRL	84	0.553
ENSP00000341551	SMAD4	190	0.554
ENSP00000365891	WAS	62	0.556
ENSP00000361066	NCOA3	11	0.559
ENSP00000269571	ERBB2	209	0.564
ENSP00000216605	MTHFD1	5	0.566
ENSP00000267163	RB1	559	0.566
ENSP00000005257	RALA	5	0.572
ENSP00000358997	IRAK1	134	0.574
ENSP00000304350	PRPF8	2	0.589
ENSP00000358554	BCAS2	2	0.589
ENSP00000216225	RBX1	233	0.592
ENSP00000300161	YWHAB	14	0.596
ENSP00000217958	PSMD10	3	0.599
ENSP00000371432	PRLR	82	0.599
ENSP00000262477	RABEP1	128	0.6
ENSP00000348554	CDC16	12	0.604

ENSP00000356070	MAPKAPK2	2	0.606
ENSP00000397552	ACTL6A	2	0.611
ENSP00000311032	CASP3	1	0.612
ENSP00000366396	XRN2	2	0.614
ENSP00000256857	GRP	23	0.622
ENSP00000284981	APP	199	0.624
ENSP00000284957	RABGEF1	128	0.626
ENSP00000357879	PSMD4	127	0.626
ENSP00000359206	BTRC	1	0.634
ENSP00000274335	PIK3R1	288	0.639
ENSP00000337825	LCK	518	0.643
ENSP00000268182	IQGAP1	38	0.645
ENSP00000262160	SMAD2	192	0.65
ENSP00000299421	ILK	8	0.652
ENSP00000324806	GSK3B	279	0.66
ENSP00000381331	HDAC2	30	0.661
ENSP00000313420	PRKDC	129	0.662
ENSP00000342793	PLD1	5	0.662
ENSP00000315644	TYMS	376	0.664
ENSP00000343204	JAK1	49	0.668
ENSP00000400175	RHOA	2	0.688
ENSP00000254066	RARA	20	0.689

ENSP00000216797	NFKBIA	1	0.691
ENSP00000358541	SIKE1	11	0.701
ENSP00000339109	ANAPC1	146	0.702
ENSP00000239223	DUSP1	4	0.705
ENSP00000295897	ALB	407	0.705
ENSP00000244007	PLCG1	273	0.715
ENSP00000361418	IPO13	1	0.715
ENSP00000301764	DDB1	9	0.72
ENSP00000306512	IL8	116	0.73
ENSP00000345571	E2F1	23	0.731
ENSP00000309103	BAD	16	0.747
ENSP00000315702	MOB4	28	0.769
ENSP00000245960	CDC25B	27	0.772
ENSP00000249299	NAA38	9	0.794
ENSP00000352980	HIST1H4A	27	0.799
ENSP00000274255	SKP2	172	0.814
ENSP00000230354	TBP	135	0.821
ENSP00000296871	CSF2	2	0.824
ENSP00000314949	POLR2A	129	0.824
ENSP00000287647	FANCD2	2	0.831
ENSP00000352516	DNMT1	2	0.833
ENSP00000356087	IKBKE	4	0.837

ENSP00000162749	TNFRSF1A	1	0.838
ENSP00000318861	SF3B2	2	0.839
ENSP00000369497	BRCA2	19	0.84
ENSP00000384053	CSF2RB	2	0.841
ENSP00000258962	SRSF1	3	0.847
ENSP00000310127	IRF3	5	0.849
ENSP00000339007	GRB2	629	0.852
ENSP00000384273	RELA	129	0.853
ENSP00000299543	CTDP1	5	0.856
ENSP00000269321	ARHGDIA	2	0.857
ENSP00000268058	PML	10	0.861
ENSP00000391592	PTPN6	10	0.876
ENSP00000215832	MAPK1	86	0.877
ENSP00000366013	GNB2L1	4	0.888
ENSP00000227378	HSPA8	183	0.891
ENSP00000396127	RAN	130	0.892
ENSP00000368438	PCNA	727	0.894
ENSP00000284811	TCEB1	4	0.898
ENSP00000235090	WDR77	205	0.899
ENSP00000247668	TRAF2	134	0.902
ENSP00000261461	PPP2R5A	11	0.902
ENSP00000348577	RANGAP1	130	0.906

ENSP00000418447	PPP2CA	138	0.907
ENSP00000229022	VDR	44	0.91
ENSP00000362592	RBBP4	8	0.915
ENSP00000315859	RNPS1	1	0.917
ENSP00000357858	BUB3	201	0.926
ENSP00000248566	SHFM1	318	0.927
ENSP00000278916	CHEK1	1	0.936
ENSP00000292644	PSMC2	610	0.94
ENSP00000264951	XRN1	122	0.952
ENSP00000348461	RAC1	219	0.953
ENSP00000270202	AKT1	1432	0.954
ENSP00000252102	NDUFA2	134	0.956
ENSP00000344818	UBC	13387	0.956
ENSP00000302269	VAV1	25	0.958
ENSP00000398597	EXOSC6	12	0.973
ENSP00000367207	MYC	43	0.975
ENSP00000324897	UBE2I	125	0.977
ENSP00000310596	LSM1	1	0.984
ENSP00000228872	CDKN1B	3	0.985
ENSP00000296271	RHO	190	0.985
ENSP00000377141	ARRB1	186	0.986
ENSP00000269349	EIF4A3	1	0.992

ENSP00000316879	EIF4G1	2	0.994
ENSP00000312735	POLR2B	1	0.997
ENSP00000414634	LSM2	8	0.997
ENSP00000366135	EXOSC10	119	0.998
ENSP00000240185	TARDBP	113	0.999
ENSP00000252622	LSM7	20	0.999
ENSP00000313829	KHDRBS1	146	0.999
ENSP00000215829	SNRPD3	22	1
ENSP00000221494	SF3A2	18	1
ENSP00000263309	CLNS1A	18	1
ENSP00000276201	UPF3B	2	1
ENSP00000307863	U2AF2	14	1
ENSP00000319169	PRMT5	18	1
ENSP00000348708	UPF2	2	1
ENSP00000365439	HNRNPK	14	1
ENSP00000417281	MDM2	226	1

3. Candidate genes for methylation CpG site genes and mRNA genes

Ensemb ID	Gene symbol	Betweenness	Permutation FDR
ENSP00000321821	CYP4F12	1	<0.001
ENSP00000352162	ELAVL3	1	<0.001
ENSP00000351209	EPHA2	3066	<0.001
ENSP00000306245	FOS	13726	<0.001

ENSP00000326371	FOXC2	1	<0.001
ENSP00000333188	FOXL2	1213	<0.001
ENSP00000354607	FZD5	2	<0.001
ENSP00000401018	GINS3	1342	<0.001
ENSP00000355140	HOXB1	1345	<0.001
ENSP00000363827	HSPG2	1222	<0.001
ENSP00000252818	JUND	9748	<0.001
ENSP00000365469	KLHL35	1	<0.001
ENSP00000312652	LEP	7481	<0.001
ENSP00000222725	LFNG	2802	<0.001
ENSP00000328364	MAFA	1344	<0.001
ENSP00000337088	MEN1	9748	<0.001
ENSP00000352262	MLL	9758	<0.001
ENSP00000257829	NAT10	930	<0.001
ENSP00000255262	NMUR2	1	<0.001
ENSP00000405890	PBX1	3895	<0.001
ENSP00000309509	PLAC8	1	<0.001
ENSP00000375557	POU5F1B	2	<0.001
ENSP00000261454	PROX1	1	<0.001
ENSP00000332353	PTCH1	5250	<0.001
ENSP00000253571	RLIM	1346	<0.001
ENSP00000297261	SHH	4782	<0.001

ENSP00000273158	SLC25A38	8	<0.001
ENSP00000075120	SLC2A3	1208	<0.001
ENSP00000262965	TCF3	4274	<0.001
ENSP00000204615	THPO	1213	<0.001
ENSP00000365682	TLE1	2693	<0.001
ENSP00000309818	TRHR	1	<0.001
ENSP00000277225	ZNF462	1	<0.001
ENSP00000325123	ZSCAN2	1	<0.001
ENSP00000373700	ALK	1213	0.001
ENSP00000314897	ANGPT2	1384	0.001
ENSP00000388996	AP1M1	1213	0.001
ENSP00000307479	ARNT2	1213	0.001
ENSP00000340858	B2M	7379	0.001
ENSP00000259216	CFC1	2	0.001
ENSP00000344456	CTNNB1	16234	0.001
ENSP00000006053	CX3CL1	1423	0.001
ENSP00000382166	CX3CR1	1423	0.001
ENSP00000227451	DTX4	1	0.001
ENSP00000263253	EP300	14130	0.001
ENSP00000296839	FOXQ1	1	0.001
ENSP00000248272	GAN	6	0.001
ENSP00000375629	LILRB2	1235	0.001

ENSP00000296589	SLC45A2	1	0.001
ENSP00000309741	SLCO4C1	1	0.001
ENSP00000268704	SPG7	134	0.001
ENSP00000264657	STAT3	14590	0.001
ENSP00000299550	TRIM66	2	0.001
ENSP00000351407	ARNT	4772	0.002
ENSP00000364092	ASIP	1196	0.002
ENSP00000320965	B4GALT3	1	0.002
ENSP00000264033	CBL	33959	0.002
ENSP00000351767	COL20A1	1	0.002
ENSP00000318057	EGR3	1211	0.002
ENSP00000287934	FZD1	2551	0.002
ENSP00000241125	GJA3	1185	0.002
ENSP00000342215	KIR2DL3	7176	0.002
ENSP00000315997	LILRB1	1212	0.002
ENSP00000346689	LMO3	1	0.002
ENSP00000354541	NLGN1	1720	0.002
ENSP00000328181	NOG	1479	0.002
ENSP00000245457	PTGER2	6	0.002
ENSP00000345487	QRFP	588	0.002
ENSP00000358799	RBM15	1212	0.002
ENSP00000384179	ZFPM2	2	0.002

ENSP00000390784	B3GALT4	1	0.003
ENSP00000362372	BRWD3	134	0.003
ENSP00000377840	CACNB1	134	0.003
ENSP00000344460	CBS	1318	0.003
ENSP00000222792	CHN2	134	0.003
ENSP00000260433	CYP19A1	5	0.003
ENSP00000355316	GRM3	2	0.003
ENSP00000407431	HLA-C	6929	0.003
ENSP00000304915	IL13	1344	0.003
ENSP00000355961	INTS7	1340	0.003
ENSP00000315757	LCP1	1	0.003
ENSP00000233616	MOGS	134	0.003
ENSP00000350833	MPPED2	1	0.003
ENSP00000340328	NYX	2	0.003
ENSP00000339328	PLAUR	2687	0.003
ENSP00000405041	POU5F1	1417	0.003
ENSP00000360076	SGIP1	1200	0.003
ENSP00000291842	SHKBP1	1213	0.003
ENSP00000294309	TPCN2	2	0.003
ENSP00000401435	VPS53	1211	0.003
ENSP00000370521	AIPL1	1210	0.004
ENSP00000252593	BST2	134	0.004

ENSP00000357025	CD48	134	0.004
ENSP00000370256	FOXC1	1209	0.004
ENSP00000229030	FZD10	1340	0.004
ENSP00000387662	GCG	5517	0.004
ENSP00000264009	HSF4	134	0.004
ENSP00000346879	NKX2-1	1611	0.004
ENSP00000221855	TBCB	2	0.004
ENSP00000256151	CCDC59	1212	0.005
ENSP00000261532	ESRRB	3	0.005
ENSP00000266659	GLIPR1	134	0.005
ENSP00000392828	GPSM1	134	0.005
ENSP00000290295	HOXB13	1212	0.005
ENSP00000408405	KCTD1	1	0.005
ENSP00000318406	KIAA0319L	134	0.005
ENSP00000310216	KLRC4	134	0.005
ENSP00000242152	NPY	1936	0.005
ENSP00000005587	SKAP2	134	0.005
ENSP00000406988	VPS52	1	0.005
ENSP00000295728	CRYBA2	134	0.006
ENSP00000252050	CUL9	134	0.006
ENSP00000369009	CXorf23	134	0.006
ENSP00000361668	EDN2	1	0.006

ENSP00000319118	GSX2	1212	0.006
ENSP00000352442	HIST1H2BM	134	0.006
ENSP00000301420	KLK1	134	0.006
ENSP00000392466	LDB1	1345	0.006
ENSP00000366347	NKX2-2	1338	0.006
ENSP00000269280	NLRP1	1337	0.006
ENSP00000364246	PLA2G2D	134	0.006
ENSP00000263431	PRKCG	1318	0.006
ENSP00000358497	RNGTT	1340	0.006
ENSP00000344822	S100A13	22	0.006
ENSP00000221283	STXBP2	134	0.006
ENSP00000365877	SUV39H1	134	0.006
ENSP00000373485	TSNAXIP1	134	0.006
ENSP00000333920	TTF1	134	0.006
ENSP00000278175	ADM	1	0.007
ENSP00000336666	AP1S1	238	0.007
ENSP00000338728	CCDC88A	134	0.007
ENSP00000310440	CHMP2A	1211	0.007
ENSP00000358576	DCLRE1B	134	0.007
ENSP00000410294	FGFR2	1750	0.007
ENSP00000238256	FKBP15	132	0.007
ENSP00000348897	GABRA2	1	0.007

ENSP00000283871	HGD	134	0.007
ENSP00000248071	KLF2	134	0.007
ENSP00000300417	LRSAM1	134	0.007
ENSP00000263369	MIA	134	0.007
ENSP00000361548	MPL	1213	0.007
ENSP00000350332	MYBPC2	134	0.007
ENSP00000261182	NAP1L1	134	0.007
ENSP00000395465	NCOA4	134	0.007
ENSP00000349275	NRG1	1225	0.007
ENSP00000233202	SLC11A1	134	0.007
ENSP00000341550	SLC24A5	1	0.007
ENSP00000267842	SLC27A2	132	0.007
ENSP00000375859	TMEM91	130	0.007
ENSP00000298552	TSC1	1198	0.007
ENSP00000320340	DGKZ	133	0.008
ENSP00000357656	FYN	9625	0.008
ENSP00000265294	GABRP	134	0.008
ENSP00000297107	GALNT10	134	0.008
ENSP00000331358	GAST	1911	0.008
ENSP00000310557	KCNE3	123	0.008
ENSP00000364946	MKX	1	0.008
ENSP00000245539	MRPS7	134	0.008

ENSP00000345752	MTMR2	2	0.008
ENSP00000395505	PATE3	2	0.008
ENSP00000355001	POU3F3	134	0.008
ENSP00000343924	PRELP	1	0.008
ENSP00000356906	SH2D1B	134	0.008
ENSP00000331791	TBX1	1211	0.008
ENSP00000225831	CCL2	1458	0.009
ENSP00000359594	CLCA4	134	0.009
ENSP00000354111	DNAJC5	1157	0.009
ENSP00000352785	DSG4	134	0.009
ENSP00000168712	FGF4	143	0.009
ENSP00000250448	FOXA1	1604	0.009
ENSP00000348815	HYLS1	1210	0.009
ENSP00000339801	IDS	2	0.009
ENSP00000398644	NUB1	1210	0.009
ENSP00000264708	POMC	3827	0.009
ENSP00000392349	RPS18	1	0.009
ENSP00000298386	RXFP2	134	0.009
ENSP00000281030	THRSP	134	0.009
ENSP00000219409	ARHGDIG	132	0.01
ENSP00000222212	CACNG7	27	0.01
ENSP00000376849	CASP5	1205	0.01

ENSP00000368450	CD83	134	0.01
ENSP00000362217	DACH2	1	0.01
ENSP00000354900	GJB1	134	0.01
ENSP00000374981	IGHA2	134	0.01
ENSP00000319591	KCND3	1	0.01
ENSP00000307218	NAT1	134	0.01
ENSP00000304767	P2RY1	270	0.01
ENSP00000293362	PSME3	1211	0.01
ENSP00000274938	SCUBE3	1	0.01
ENSP00000361867	SEMG1	134	0.01
ENSP00000219473	USP10	415	0.01
ENSP00000260600	ADCY3	134	0.011
ENSP00000297135	COG5	134	0.011
ENSP00000304414	CXCR6	1199	0.011
ENSP00000340396	GBP5	134	0.011
ENSP00000261170	GUCY2C	134	0.011
ENSP00000343819	OTX2	134	0.011
ENSP00000274793	PLA2G7	134	0.011
ENSP00000331057	TCF12	1212	0.011
ENSP00000359215	TLX1	1206	0.011
ENSP00000315173	ZNF41	134	0.011
ENSP00000298032	ARMC3	134	0.012

ENSP00000368683	EDN1	1715	0.012
ENSP00000226091	EFNB3	134	0.012
ENSP00000356162	KISS1	587	0.012
ENSP00000302114	PRELID1	134	0.012
ENSP00000348573	AKAP9	15	0.013
ENSP00000295600	MITF	1210	0.013
ENSP00000414598	MRVI1	134	0.013
ENSP00000354033	PCGF2	134	0.013
ENSP00000329968	PHKG2	134	0.013
ENSP00000355896	TGFB2	134	0.013
ENSP00000266085	TIMP3	134	0.013
ENSP00000219281	USB1	134	0.013
ENSP00000225941	ABI3	134	0.014
ENSP00000393887	AHSG	268	0.014
ENSP00000350256	CCR9	264	0.014
ENSP00000323280	CD6	262	0.014
ENSP00000199764	CEACAM6	268	0.014
ENSP00000222982	CYP3A5	9	0.014
ENSP00000278823	MTA2	134	0.014
ENSP00000361658	NUP188	134	0.014
ENSP00000408395	RBFOX3	134	0.014
ENSP00000274031	SETD7	134	0.014

ENSP00000343785	SPRY1	134	0.014
ENSP00000267859	BNIP2	134	0.015
ENSP00000384169	FBLN2	3	0.015
ENSP00000228837	FGF6	1989	0.015
ENSP00000263269	GRIN2D	2	0.015
ENSP00000294973	HAAO	134	0.015
ENSP00000260570	IFT172	158	0.015
ENSP00000262545	PCSK2	134	0.015
ENSP00000328968	SCN5A	268	0.015
ENSP00000216373	SOS2	134	0.015
ENSP00000276072	TAF1	134	0.015
ENSP00000316990	TRAPPC5	134	0.015
ENSP00000206474	HAUS4	134	0.016
ENSP00000339521	RSU1	134	0.016
ENSP00000340237	SH3BP4	134	0.016
ENSP00000231524	TRIM23	268	0.016
ENSP00000329757	ATP6V0C	134	0.017
ENSP00000242839	ATP7B	134	0.017
ENSP00000372815	C4A	402	0.017
ENSP00000259938	CLPS	134	0.017
ENSP00000241256	GHSR	241	0.017
ENSP00000348394	NCDN	134	0.017

ENSP00000389244	SLC44A4	1	0.017
ENSP00000360371	SSBP3	134	0.017
ENSP00000264637	THRA	310	0.017
ENSP00000389792	DCDC1	134	0.018
ENSP00000251337	GNAT2	1340	0.018
ENSP00000304604	MAGI3	132	0.018
ENSP00000300061	SCNN1G	134	0.018
ENSP00000019103	SCTR	292	0.018
ENSP00000220876	STMN2	134	0.018
ENSP00000162330	BCAR1	2212	0.019
ENSP00000270223	DMWD	134	0.019
ENSP00000276420	DOK2	682	0.019
ENSP00000273390	MAATS1	134	0.019
ENSP00000304553	MPLKIP	134	0.019
ENSP00000291386	SSU72	399	0.019
ENSP00000283147	BMP6	268	0.02
ENSP00000413720	CDKN1C	263	0.02
ENSP00000298472	SLC18A2	134	0.02
ENSP00000353165	TPK1	134	0.02
ENSP00000264234	UPK1B	268	0.02
ENSP00000358866	FLNA	1510	0.021
ENSP00000240093	FZD3	134	0.021

ENSP00000356737	GORAB	134	0.021
ENSP00000233809	IGFBP2	134	0.021
ENSP00000348986	INS-IGF2	3760	0.021
ENSP00000257818	LMO2	1358	0.021
ENSP00000229307	NANOG	1264	0.021
ENSP00000350447	SGOL2	134	0.021
ENSP00000201586	SULT2B1	402	0.021
ENSP00000366593	TMEM201	134	0.021
ENSP00000341032	WNT7B	1339	0.021
ENSP00000378326	ZP3	134	0.021
ENSP00000305988	ALCAM	262	0.022
ENSP00000357033	CD84	134	0.022
ENSP00000216338	GZMH	134	0.022
ENSP00000273221	IQSEC1	134	0.022
ENSP00000333496	KCND2	134	0.022
ENSP00000262105	MCM4	1550	0.022
ENSP00000393275	NANOS1	1	0.022
ENSP00000363431	NPY4R	132	0.022
ENSP00000260643	PREB	134	0.022
ENSP00000234739	BCL9	268	0.023
ENSP00000373091	HLA-E	23	0.023
ENSP00000375009	IGHV3-9	134	0.023

ENSP00000262776	LGALS3BP	268	0.023
ENSP00000223140	NOBOX	134	0.023
ENSP00000340944	PTPN11	3077	0.023
ENSP00000369756	PTPRA	134	0.023
ENSP00000309968	ADAM17	134	0.024
ENSP00000308496	ARL10	1	0.024
ENSP00000247170	DAAM1	268	0.024
ENSP00000391457	INO80C	134	0.024
ENSP00000262186	KCNH2	1894	0.024
ENSP00000269468	MBD1	134	0.024
ENSP00000341815	SOX18	134	0.024
ENSP00000286604	UGT2A1	1	0.024
ENSP00000361125	VEGFA	3862	0.024
ENSP00000258682	CAMK2B	134	0.025
ENSP00000289429	CD1A	134	0.025
ENSP00000305442	COG7	134	0.025
ENSP00000331902	COL4A5	267	0.025
ENSP00000266544	NDUFA9	134	0.025
ENSP00000216277	PAPOLA	134	0.025
ENSP00000317721	PIPOX	1	0.025
ENSP00000417164	ROBO2	134	0.025
ENSP00000229201	TIMELESS	134	0.025

ENSP00000265310	TRPV5	133	0.025
ENSP00000247843	YEATS4	1212	0.025
ENSP00000290551	BTG2	134	0.026
ENSP00000410076	CASP1	1735	0.026
ENSP00000357311	CENPW	134	0.026
ENSP00000401632	GSTT1	133	0.026
ENSP00000357283	LMNA	1345	0.026
ENSP00000380066	MAP4K1	1204	0.026
ENSP00000357721	S100A8	262	0.026
ENSP00000361554	TIE1	134	0.026
ENSP00000225844	CCL13	264	0.027
ENSP00000227507	CCND1	11214	0.027
ENSP00000292301	CCR2	36	0.027
ENSP00000161559	CEACAM1	402	0.027
ENSP00000356056	DYNLT1	134	0.027
ENSP00000264126	GPSM2	268	0.027
ENSP00000281938	HSPB8	134	0.027
ENSP00000303830	INSR	3549	0.027
ENSP00000374409	PKP4	134	0.027
ENSP00000295709	STK36	266	0.027
ENSP00000315167	ALOX12B	134	0.028
ENSP00000333769	BSG	1345	0.028

ENSP00000388340	CLINT1	134	0.028
ENSP00000353344	ETS2	134	0.028
ENSP00000388724	HLA-A	134	0.028
ENSP00000362071	JPH2	134	0.028
ENSP00000278198	LRRC4C	134	0.028
ENSP00000157812	PSMC4	1699	0.028
ENSP00000359285	CHRNA4	397	0.029
ENSP00000239223	DUSP1	155	0.029
ENSP00000370962	GGT6	134	0.029
ENSP00000257879	ITGA7	134	0.029
ENSP00000254351	SDC1	407	0.029
ENSP00000281821	EPHA4	402	0.03
ENSP00000289004	HPD	134	0.03
ENSP00000292174	CXCR5	1	0.031
ENSP00000338018	HIF1A	16357	0.031
ENSP00000345096	IMPDH1	133	0.031
ENSP00000347409	KEL	134	0.031
ENSP00000321239	RCHY1	134	0.031
ENSP00000292169	S100A1	135	0.031
ENSP00000398852	SLC44A4	132	0.031
ENSP00000350162	SYCP2	268	0.031
ENSP00000365048	TNFSF13B	134	0.031

ENSP00000295006	CAPN2	134	0.032
ENSP00000344353	LPAR6	134	0.032
ENSP00000257068	MTNR1B	9	0.032
ENSP00000303325	TACR3	8	0.032
ENSP00000260283	ARHGAP20	134	0.033
ENSP00000365569	FLOT1	134	0.033
ENSP00000363163	NLGN3	1	0.033
ENSP00000369325	CDKL5	134	0.034
ENSP00000371308	CENPJ	1210	0.034
ENSP00000331933	WSCD2	1	0.034
ENSP00000334424	AMACR	1	0.035
ENSP00000325136	HADHB	116	0.035
ENSP00000314080	HIC1	134	0.035
ENSP00000260950	MSTN	134	0.035
ENSP00000360502	PDE6C	4	0.035
ENSP00000005226	USH1C	268	0.035
ENSP00000353654	COL4A2	266	0.036
ENSP00000267085	CSAD	6	0.036
ENSP00000334458	GATA4	1469	0.036
ENSP00000329384	IL22	134	0.036
ENSP00000353408	MSN	268	0.036
ENSP00000274311	PELO	134	0.036

ENSP00000241463	RASL11A	1	0.036
ENSP00000354720	SMC3	1785	0.036
ENSP00000338345	SNCA	1706	0.036
ENSP00000350937	TES	134	0.036
ENSP00000356789	ATP1B1	402	0.037
ENSP00000357244	CCT3	134	0.037
ENSP00000219172	CENPT	134	0.037
ENSP00000305913	COL8A2	134	0.037
ENSP00000369962	IGSF5	133	0.037
ENSP00000383690	MASP2	402	0.037
ENSP00000318472	NCAM1	1608	0.037
ENSP00000236137	SLC19A2	2	0.037
ENSP00000309913	TBX5	137	0.037
ENSP00000262320	AXIN1	5746	0.038
ENSP00000357348	HEY2	134	0.038
ENSP00000293379	ITGA5	5082	0.038
ENSP00000378090	RAD51D	134	0.038
ENSP00000217381	SNTA1	268	0.038
ENSP00000340088	THEG	268	0.038
ENSP00000263640	ACVR1	133	0.039
ENSP00000272238	ATP6V1C2	266	0.039
ENSP00000260630	CYP1B1	258	0.039

ENSP00000259206	IL1RN	134	0.039
ENSP00000372991	LTA	268	0.039
ENSP00000316244	HTR1A	273	0.04
ENSP00000280193	VEGFC	268	0.04
ENSP00000321674	4-Sep	134	0.041
ENSP00000362353	GLP1R	1	0.041
ENSP00000231751	LTF	134	0.041
ENSP00000362768	RBL1	132	0.041
ENSP00000305714	BMP1	268	0.042
ENSP00000356694	FASLG	1209	0.042
ENSP00000381340	GGT5	1	0.042
ENSP00000319197	OR4K17	4	0.042
ENSP00000301258	PSCA	133	0.042
ENSP00000264998	TF	442	0.042
ENSP00000391490	AGR2	134	0.043
ENSP00000331172	CD8B	101	0.043
ENSP00000248996	GNAZ	263	0.043
ENSP00000324834	MUC3A	134	0.043
ENSP00000351284	RAD52	134	0.043
ENSP00000268864	RASL10B	134	0.043
ENSP00000323568	SLC2A2	267	0.043
ENSP00000255608	BTBD2	133	0.044

ENSP00000309052	CATSPER1	134	0.044
ENSP00000261769	CDH1	2119	0.044
ENSP00000356623	CITED2	268	0.044
ENSP00000311827	MSL2	134	0.044
ENSP00000351790	MYPN	4	0.044
ENSP00000396915	SCN1B	245	0.044
ENSP00000227135	SPA17	134	0.044
ENSP00000409231	TRAPPC13	134	0.044
ENSP00000236192	VAMP4	1209	0.044
ENSP00000293288	BAX	1293	0.045
ENSP00000320866	CALR	2011	0.045
ENSP00000354901	CXCL9	1311	0.045
ENSP00000303077	GOT1L1	133	0.045
ENSP00000170630	IL4R	1405	0.045
ENSP00000254301	LGALS3	268	0.045
ENSP00000354130	SOX10	269	0.045
ENSP00000261037	COL8A1	267	0.046
ENSP00000360968	CYP4X1	134	0.046
ENSP00000334145	F3	134	0.046
ENSP00000248594	PTPN12	134	0.046
ENSP00000345793	ZC3H7B	133	0.046
ENSP00000420418	ZNF398	132	0.046

ENSP00000335544	CCKBR	585	0.047
ENSP00000264001	CKLF	133	0.047
ENSP00000327417	GPR39	133	0.047
ENSP00000358421	HSD3B1	34	0.047
ENSP00000386165	CEBPD	390	0.048
ENSP00000232458	ECT2	134	0.048
ENSP00000276533	GINS4	10	0.048
ENSP00000351155	ATL1	134	0.049
ENSP00000380227	ITGA4	1829	0.049
ENSP00000200181	ITGB4	1314	0.049
ENSP00000265969	KCNC1	173	0.049
ENSP00000328570	GLRX5	4	0.05
ENSP00000338562	STX3	134	0.05
ENSP00000286827	TIAM1	268	0.05
ENSP00000335657	CCK	599	0.051
ENSP00000302665	IGF1	5133	0.051
ENSP00000381412	CAMK2A	134	0.052
ENSP00000304822	CSN3	95	0.052
ENSP00000262958	GNA15	134	0.052
ENSP00000417132	BAP1	1212	0.053
ENSP00000300574	CRK	2287	0.053
ENSP00000324831	OR4P4	1	0.053

ENSP00000228307	PXN	1458	0.053
ENSP00000260383	TUBGCP4	132	0.053
ENSP00000377148	AP1G1	975	0.054
ENSP00000395535	MECP2	268	0.054
ENSP00000325120	PGR	134	0.054
ENSP00000371341	TNK2	134	0.054
ENSP00000320130	DYNC1I1	268	0.055
ENSP00000386331	MYO7A	268	0.055
ENSP00000300289	PDIA3	1887	0.055
ENSP00000184183	ROPN1	134	0.055
ENSP00000310658	SCUBE2	133	0.055
ENSP00000353224	TFRC	459	0.055
ENSP00000246533	CAPNS1	134	0.056
ENSP00000405708	CCDC39	134	0.056
ENSP00000315011	EDNRA	267	0.056
ENSP00000369654	HBD	6	0.056
ENSP00000400842	HLA-B	6	0.056
ENSP00000259089	BLK	134	0.057
ENSP00000377958	CCT4	398	0.057
ENSP00000265986	IDE	3	0.057
ENSP00000358525	NGF	2137	0.057
ENSP00000256196	RRAS2	134	0.057

ENSP00000216064	SUN2	133	0.057
ENSP00000348234	TAT	134	0.057
ENSP00000301200	CDC42EP5	134	0.058
ENSP00000247655	COX7C	133	0.058
ENSP00000368686	E2F4	142	0.058
ENSP00000338799	IL6ST	1545	0.058
ENSP00000363390	TRIM63	73	0.058
ENSP00000380184	AGPAT6	135	0.059
ENSP00000387123	ALDH7A1	4	0.059
ENSP00000395546	CSNK2B	134	0.059
ENSP00000346901	FMO1	1	0.059
ENSP00000382791	GRIK1	134	0.059
ENSP00000386896	ITGA6	1336	0.059
ENSP00000263923	KDR	477	0.059
ENSP00000307445	LTB4R	1	0.059
ENSP00000362555	RNF19B	2	0.059
ENSP00000265727	ADAM22	134	0.06
ENSP00000247461	CANX	2280	0.06
ENSP00000265773	SMARCA2	1214	0.06
ENSP00000326261	SRRM1	134	0.06
ENSP00000293897	SSTR5	14	0.06
ENSP00000280326	CCT5	268	0.061

ENSP00000315955	FOXA2	490	0.061
ENSP00000356430	RGS18	1	0.061
ENSP00000400806	APTX	268	0.062
ENSP00000259407	BAAT	1	0.062
ENSP00000368754	BAZ2A	134	0.062
ENSP00000297518	CDK5	2647	0.062
ENSP00000280357	IL18	268	0.062
ENSP00000396439	RING1	134	0.062
ENSP00000360992	STAMBPL1	2	0.062
ENSP00000287139	NODAL	134	0.063
ENSP00000260130	SDCBP	410	0.063
ENSP00000263233	SYP	134	0.063
ENSP00000360302	GRIA3	132	0.064
ENSP00000155840	KCNQ1	266	0.064
ENSP00000369695	MLLT3	134	0.064
ENSP00000363216	OGDHL	134	0.064
ENSP00000348888	PIGR	134	0.064
ENSP00000277575	USP6NL	134	0.064
ENSP00000406335	CLIC1	134	0.065
ENSP00000181796	FAM107B	268	0.065
ENSP00000370223	IDH3B	1	0.065
ENSP00000296181	ITGB5	3	0.065

ENSP00000301972	MYRIP	268	0.065
ENSP00000285402	ODF1	134	0.065
ENSP00000248933	SEZ6L	1	0.065
ENSP00000418070	PVRL3	1	0.066
ENSP00000369647	AVP	425	0.067
ENSP00000325660	CNTN1	245	0.067
ENSP00000340820	MAPT	2632	0.067
ENSP00000363079	MBL2	535	0.067
ENSP00000273480	RNF7	1	0.067
ENSP00000229266	CHPT1	132	0.068
ENSP00000357392	EFNA1	390	0.068
ENSP00000343040	HMGB1	134	0.068
ENSP00000228280	KITLG	134	0.068
ENSP00000274711	LRRTM2	1	0.068
ENSP00000218388	TIMP1	302	0.068
ENSP00000269485	TNFRSF11A	134	0.068
ENSP00000230340	BYSL	135	0.069
ENSP00000369129	DSP	268	0.069
ENSP00000357753	IVL	268	0.069
ENSP00000341170	PTN	17	0.069
ENSP00000361646	ZMYND12	132	0.069
ENSP00000265708	ADAM2	134	0.07

ENSP00000362795	CXCR3	135	0.07
ENSP00000275764	STRA8	1	0.07
ENSP00000005279	SYNRG	971	0.07
ENSP00000355599	TSNAX	134	0.07
ENSP00000342510	CEP97	1	0.071
ENSP00000265012	GCNT2	5	0.071
ENSP00000231509	NR3C1	245	0.071
ENSP00000317128	PLXND1	268	0.071
ENSP00000258123	USP15	268	0.071
ENSP00000204604	CHRD	402	0.072
ENSP00000262629	TYROBP	7503	0.072
ENSP00000366006	UBIAD1	266	0.072
ENSP00000365643	DOCK9	125	0.073
ENSP00000261366	LMNB1	1345	0.073
ENSP00000275874	RAB19	134	0.073
ENSP00000308208	MMP14	135	0.074
ENSP00000394624	OPRM1	412	0.074
ENSP00000340677	WNT8B	1	0.074
ENSP00000257963	ACVR1B	134	0.075
ENSP00000289902	FCER1G	402	0.076
ENSP00000268171	FURIN	536	0.076
ENSP00000311605	OR4B1	133	0.076

ENSP00000043402	RTN4R	268	0.076
ENSP00000362095	SRPX2	131	0.076
ENSP00000296140	CCR1	402	0.077
ENSP00000307853	MUS81	134	0.077
ENSP00000364979	COL4A1	266	0.078
ENSP00000349436	ADAM15	132	0.079
ENSP00000318128	BLOC1S4	2	0.079
ENSP00000368104	BMP2	2458	0.079
ENSP00000370408	CDX2	134	0.079
ENSP00000321345	IL23R	19	0.079
ENSP00000296218	DNALI1	134	0.08
ENSP00000351755	LPAR1	1	0.08
ENSP00000303212	SEMA3E	268	0.08
ENSP00000355370	CNTF	134	0.081
ENSP00000397177	PBX2	134	0.081
ENSP00000392858	TNF	134	0.081
ENSP00000332247	ATP6V0A2	133	0.082
ENSP00000264741	ITGA9	134	0.082
ENSP00000371514	KCNV2	3	0.082
ENSP00000277541	NOTCH1	5491	0.082
ENSP00000360054	PHACTR3	133	0.082
ENSP00000308938	PLG	3999	0.082

ENSP00000275605	PSPH	134	0.082
ENSP00000244741	CDKN1A	1572	0.083
ENSP00000372023	CHEK2	136	0.083
ENSP00000255945	GIMAP4	133	0.083
ENSP00000160262	ICAM3	134	0.083
ENSP00000396622	MROH7	134	0.083
ENSP00000356000	PLXNA2	265	0.083
ENSP00000263642	IFIH1	134	0.084
ENSP00000249075	LIF	1380	0.084
ENSP00000364898	SYK	7881	0.085
ENSP00000390849	ABHD5	278	0.086
ENSP00000311219	TRIM59	133	0.086
ENSP00000254950	VPS4A	1211	0.086
ENSP00000358857	EMD	134	0.087
ENSP00000258301	STX6	1336	0.087
ENSP00000265132	AMBP	378	0.088
ENSP00000370503	CCM2	134	0.088
ENSP00000333275	NR2C1	136	0.088
ENSP00000307781	ODF2	134	0.088
ENSP00000327801	P4HB	268	0.088
ENSP00000375921	PAX3	397	0.088
ENSP00000350869	ZNF346	133	0.088

ENSP00000348107	C1D	134	0.089
ENSP00000261267	LYZ	167	0.089
ENSP00000205948	APOH	14	0.09
ENSP00000354623	DFNB31	134	0.09
ENSP00000354586	GLI2	826	0.09
ENSP00000324769	OR4C6	1	0.09
ENSP00000334050	TAS2R42	2	0.09
ENSP00000351905	TGFBR2	134	0.09
ENSP00000349708	ZMYM6	1	0.09
ENSP00000227667	APOC3	7	0.091
ENSP00000291554	CRYAA	286	0.091
ENSP00000298854	RAPSN	134	0.091
ENSP00000342554	STX2	1	0.091
ENSP00000331746	CALCA	571	0.092
ENSP00000273308	CNPY2	133	0.092
ENSP00000355596	DISC1	134	0.092
ENSP00000223642	C5	268	0.093
ENSP00000404179	DOCK4	133	0.093
ENSP00000308461	RND1	399	0.093
ENSP00000299293	FRS2	12	0.094
ENSP00000263093	SLC27A5	1	0.094
ENSP00000219548	STUB1	2419	0.094

ENSP00000358918	SUFU	306	0.094
ENSP00000263817	ABCB11	268	0.095
ENSP00000312697	DMAP1	1327	0.096
ENSP00000276414	GNRH1	876	0.096
ENSP00000265447	ANXA11	132	0.097
ENSP00000255465	CCNA1	134	0.097
ENSP00000245323	EFNB2	140	0.097
ENSP00000254854	GUCY2D	134	0.097
ENSP00000365402	HLA-C	262	0.097
ENSP00000320885	SPAST	134	0.097
ENSP00000311697	FGF5	133	0.098
ENSP00000359675	GNG5	1	0.098
ENSP00000355865	PARK2	134	0.098
ENSP00000324740	YES1	133	0.098
ENSP00000306330	YWHAG	134	0.098
ENSP00000250615	AANAT	3	0.099
ENSP00000352798	COL18A1	402	0.099
ENSP00000419361	ADCY5	5	0.1
ENSP00000380280	FGFR1	3200	0.1
ENSP00000360683	PTPN1	813	0.1
ENSP00000366453	TJP2	135	0.1
ENSP00000361423	ABL1	2103	0.101

ENSP00000295598	ATP1A1	535	0.101
ENSP00000283006	CENPH	134	0.101
ENSP00000258654	COG3	134	0.101
ENSP00000264010	CTCF	133	0.101
ENSP00000410007	FBXO46	134	0.101
ENSP00000370473	IGFBP3	4004	0.101
ENSP00000348273	MBP	134	0.102
ENSP00000254480	SMARCC1	1239	0.102
ENSP00000348762	LSS	132	0.103
ENSP00000354929	NOTCH2NL	268	0.103
ENSP00000383623	MLLT4	1339	0.104
ENSP00000350878	S1PR3	246	0.104
ENSP00000379310	CASC1	266	0.105
ENSP00000400717	GNA13	394	0.105
ENSP00000351926	AP2A1	1197	0.106
ENSP00000312244	MSL3	134	0.106
ENSP00000246672	NR1D1	1	0.106
ENSP00000364839	ASXL1	74	0.107
ENSP00000316338	BAIAP2	134	0.107
ENSP00000268296	ITGAX	10	0.107
ENSP00000361818	SDC4	134	0.107
ENSP00000219476	TSC2	2512	0.107

ENSP00000356771	F5	134	0.108
ENSP00000229794	MAPK14	2246	0.108
ENSP00000338785	STARD13	122	0.108
ENSP00000282344	USP12	134	0.108
ENSP00000233114	MDH1	133	0.109
ENSP00000226218	SEBOX	221	0.109
ENSP00000367124	SLC3A2	135	0.109
ENSP00000270458	CACNG8	105	0.11
ENSP00000358414	HMGCS2	115	0.11
ENSP00000244007	PLCG1	4155	0.11
ENSP00000251772	PLXNA1	134	0.11
ENSP00000265512	ADH4	2	0.111
ENSP00000242057	AHR	2451	0.111
ENSP00000290100	EPB41	135	0.111
ENSP00000417404	HFE	578	0.111
ENSP00000336701	RAD51C	134	0.111
ENSP00000360882	COL5A1	4	0.112
ENSP00000330284	NPBWR1	133	0.112
ENSP00000310170	FOSL1	402	0.113
ENSP00000370589	NOP56	133	0.113
ENSP00000291901	TNNT1	267	0.113
ENSP00000267845	HDC	131	0.114

ENSP00000260766	PLCE1	6	0.114
ENSP00000267890	TTBK2	132	0.114
ENSP00000415183	MUC2	268	0.116
ENSP00000334008	PARVA	134	0.116
ENSP00000249042	TST	1	0.116
ENSP00000290200	IL10RB	134	0.117
ENSP00000339992	MYB	1621	0.117
ENSP00000314774	HES7	123	0.118
ENSP00000206542	OSGEP	134	0.118
ENSP00000313752	SSNA1	134	0.118
ENSP00000340466	GANAB	134	0.119
ENSP00000305689	AFF1	134	0.12
ENSP00000326031	PPP1CA	1738	0.12
ENSP00000346294	S100A4	269	0.12
ENSP00000401303	SHC1	3359	0.12
ENSP00000347198	SRGAP1	266	0.12
ENSP00000315325	ARHGEF2	134	0.121
ENSP00000228682	GLI1	312	0.121
ENSP00000300055	PLIN1	278	0.121
ENSP00000010338	TRAF3IP3	268	0.121
ENSP00000314414	AP2B1	1	0.122
ENSP00000292303	CCR5	1682	0.122

ENSP00000264554	SHC2	454	0.123
ENSP00000253024	TRIM28	1212	0.123
ENSP00000372975	HLA-C	1347	0.124
ENSP00000234313	PLEK	134	0.124
ENSP00000279387	PPP4C	134	0.124
ENSP00000044462	PSMA4	525	0.124
ENSP00000262375	DNAJA3	18	0.125
ENSP00000413596	PPP1R18	134	0.125
ENSP00000249647	SNAP23	1462	0.125
ENSP00000346155	UCKL1	2	0.125
ENSP00000354960	COLGALT2	1	0.126
ENSP00000333640	EYA2	1	0.126
ENSP00000349252	ITGAL	134	0.126
ENSP00000328236	KNTC1	2	0.126
ENSP00000346032	ANXA2	397	0.127
ENSP00000263645	CD81	133	0.127
ENSP00000318445	ST3GAL1	134	0.127
ENSP00000351486	NTRK1	2922	0.128
ENSP00000312017	FAM57A	4	0.129
ENSP00000281708	FBXW7	444	0.129
ENSP00000339467	RHOG	132	0.129
ENSP00000361759	BEX2	5	0.13

ENSP00000256958	SLCO1B1	268	0.13
ENSP00000300134	STAT6	1656	0.13
ENSP00000257254	APLNR	8	0.131
ENSP00000316854	ATOX1	134	0.131
ENSP00000265276	GPAM	123	0.131
ENSP00000328269	HMG20B	132	0.131
ENSP00000308179	WDR3	3	0.131
ENSP00000380942	ARHGEF12	636	0.132
ENSP00000272430	RTKN	133	0.132
ENSP00000355629	COG2	134	0.133
ENSP00000264426	GRIA2	3	0.133
ENSP00000328708	RXFP3	1	0.133
ENSP00000343418	SEMA4D	157	0.133
ENSP00000247161	ELK1	57	0.134
ENSP00000308782	GP6	18	0.134
ENSP00000216392	PYGL	134	0.134
ENSP00000262839	TRPC5	138	0.134
ENSP00000175506	ASNS	264	0.135
ENSP00000222390	HGF	134	0.135
ENSP00000259633	CD72	124	0.136
ENSP00000364802	HSPA1A	133	0.137
ENSP00000256720	LPIN1	135	0.137

ENSP00000220809	PLAT	531	0.137
ENSP00000390427	PPIL2	34	0.137
ENSP00000227163	SPI1	136	0.137
ENSP00000301838	FADD	1193	0.139
ENSP00000356975	ADAMTS4	131	0.14
ENSP00000271620	PRUNE	6	0.14
ENSP00000310244	RASGRP1	24	0.14
ENSP00000368350	TPT1	535	0.14
ENSP00000262134	LPCAT2	134	0.141
ENSP00000343144	PARD6G	134	0.141
ENSP00000385019	CACNA1I	133	0.142
ENSP00000266970	CDK2	5860	0.144
ENSP00000365663	NPPA	529	0.144
ENSP00000347379	OCLN	268	0.144
ENSP00000326630	ZFPM1	12	0.144
ENSP00000368169	DVL1	668	0.145
ENSP00000327758	NKX2-5	259	0.145
ENSP00000264634	WNT5A	133	0.145
ENSP00000321326	F2R	136	0.147
ENSP00000293272	CCL5	647	0.148
ENSP00000374372	SPTB	134	0.148
ENSP00000356579	CEP350	134	0.149

ENSP00000266427	ETV6	133	0.149
ENSP00000337127	SOD2	402	0.149
ENSP00000265354	SRF	1590	0.149
ENSP00000363708	BMPR2	269	0.15
ENSP00000367462	OLAH	133	0.15
ENSP00000284384	PRKCA	1467	0.15
ENSP00000344468	SDC3	41	0.15
ENSP00000350009	USP33	9	0.15
ENSP00000256759	FST	134	0.151
ENSP00000360181	SH2D1A	134	0.151
ENSP00000337103	CHAT	134	0.152
ENSP00000359151	DBT	94	0.152
ENSP00000380349	CAPN3	134	0.153
ENSP00000331544	FBLN1	130	0.153
ENSP00000332973	SMAD3	1641	0.153
ENSP00000341551	SMAD4	2473	0.153
ENSP00000316460	FYB	268	0.154
ENSP00000372860	VARS2	121	0.155
ENSP00000312262	ADRBK1	334	0.157
ENSP00000263182	BBOX1	134	0.157
ENSP00000401397	DDR1	1	0.157
ENSP00000264039	GPC1	2	0.157

ENSP00000228850	AKAP3	535	0.158
ENSP00000280346	DLAT	134	0.158
ENSP00000267082	ITGB7	1045	0.158
ENSP00000261669	CAB39L	3	0.159
ENSP00000267101	ERBB3	1468	0.159
ENSP00000355541	HEATR1	1	0.159
ENSP00000349298	MYLIP	133	0.16
ENSP00000357177	ARHGEF11	268	0.161
ENSP00000296440	PLXNB1	399	0.161
ENSP00000323516	UTS2R	132	0.161
ENSP00000318351	BCKDHB	130	0.162
ENSP00000334122	FGF3	528	0.162
ENSP00000283195	RANBP2	129	0.162
ENSP00000307491	WDR48	134	0.162
ENSP00000298687	NDRG2	131	0.165
ENSP00000362082	CCND3	266	0.166
ENSP00000236147	SELL	134	0.166
ENSP00000294353	ZYG11B	236	0.167
ENSP00000360268	ALDH18A1	134	0.168
ENSP00000360916	VAV2	449	0.168
ENSP00000206249	ESR1	17141	0.169
ENSP00000403557	PPP1R11	1	0.17

ENSP00000311677	PPP1R8	1729	0.17
ENSP00000305422	CEBPB	75	0.171
ENSP00000338369	CELA3B	19	0.171
ENSP00000261207	PPP1R12A	134	0.171
ENSP00000225402	AATF	921	0.172
ENSP00000252945	CYP2E1	542	0.172
ENSP00000263360	EED	17	0.172
ENSP00000330393	LEPR	135	0.174
ENSP00000252674	MLLT1	134	0.174
ENSP00000248553	HSPB1	674	0.175
ENSP00000323580	IFT88	128	0.175
ENSP00000363435	ITPR3	50	0.175
ENSP00000244289	LIPE	285	0.175
ENSP00000301788	POLR2G	186	0.175
ENSP00000225396	TADA2A	134	0.175
ENSP00000265171	EGF	1313	0.176
ENSP00000312999	GNAI2	704	0.176
ENSP00000349594	ELAVL4	4	0.177
ENSP00000250495	NEDD8	1210	0.177
ENSP00000339861	ENY2	133	0.179
ENSP00000260356	THBS1	65	0.179
ENSP00000263281	GIPR	3	0.18

ENSP00000338297	IGF2	427	0.18
ENSP00000303522	TACR1	14	0.18
ENSP00000284818	LY96	262	0.181
ENSP00000270474	PDE4A	287	0.181
ENSP00000262304	PKD1	128	0.181
ENSP00000358272	NDUFAF4	132	0.182
ENSP00000304283	RAC3	133	0.182
ENSP00000292907	COX7A1	133	0.183
ENSP00000360316	DHCR24	134	0.183
ENSP00000350003	CCR3	228	0.186
ENSP00000270349	SLC6A3	2	0.186
ENSP00000305464	APLN	1	0.187
ENSP00000366563	PIK3CD	132	0.187
ENSP00000246032	STK35	133	0.187
ENSP00000229264	GNB3	127	0.188
ENSP00000354927	MAP3K3	134	0.188
ENSP00000318297	RUVBL1	1337	0.188
ENSP00000306881	SEC23A	134	0.188
ENSP00000283635	CD8A	152	0.189
ENSP00000286301	CSF1R	268	0.189
ENSP00000353483	MAPK8	2351	0.189
ENSP00000237014	TTR	417	0.19

ENSP00000378338	GIT1	279	0.191
ENSP00000309555	HCFC1	1137	0.191
ENSP00000313936	OR2AE1	2	0.191
ENSP00000290039	CACHD1	3	0.192
ENSP00000241052	CAT	268	0.192
ENSP00000262768	TIMP2	135	0.194
ENSP00000359206	BTRC	175	0.195
ENSP00000347197	C5AR1	2	0.195
ENSP00000053867	GRN	116	0.195
ENSP00000221770	POP4	2	0.195
ENSP00000377941	ACTN1	134	0.196
ENSP00000353154	NFASC	139	0.197
ENSP00000396620	NFYC	132	0.197
ENSP00000317327	UBASH3A	133	0.197
ENSP00000350199	AP1B1	2	0.198
ENSP00000245907	C3	669	0.199
ENSP00000302961	HSPA4	720	0.199
ENSP00000271526	PRCC	34	0.199
ENSP00000199280	AQP2	133	0.2
ENSP00000302150	PRL	1497	0.2
ENSP00000338173	PUM2	1	0.2
ENSP00000338964	GGT7	5	0.201

ENSP00000335074	GHRL	374	0.201
ENSP00000299299	PCBD1	133	0.202
ENSP00000342755	RNF41	266	0.204
ENSP00000370557	MIS12	133	0.205
ENSP00000362690	NR5A1	1	0.205
ENSP00000288266	APPL1	134	0.206
ENSP00000265563	PRKAR2A	803	0.206
ENSP00000363092	PRKG1	402	0.206
ENSP00000231572	RARS	135	0.206
ENSP00000413234	AP2A2	238	0.208
ENSP00000361186	TP53RK	134	0.208
ENSP00000360372	CYP2C19	1	0.21
ENSP00000281453	MLF1IP	251	0.211
ENSP00000391681	POU5F1	133	0.211
ENSP00000311469	GSTM1	339	0.212
ENSP00000257497	ANXA1	129	0.213
ENSP00000282588	ITGA1	8	0.214
ENSP00000316333	CD55	133	0.215
ENSP00000257555	HNF1A	135	0.216
ENSP00000355587	NTPCR	132	0.216
ENSP00000262052	SLC11A2	6	0.216
ENSP00000054668	UTS2	130	0.216

ENSP00000222005	CDC37	1372	0.217
ENSP00000264834	KLF1	133	0.217
ENSP00000260795	FGFR3	265	0.219
ENSP00000256010	NTS	148	0.219
ENSP00000361162	TOE1	8	0.219
ENSP00000261799	PDGFRB	682	0.22
ENSP00000263409	LIFR	134	0.221
ENSP00000360798	EPS15	1567	0.222
ENSP00000357708	S100A6	132	0.222
ENSP00000265371	NRP1	402	0.223
ENSP00000325690	CARM1	7	0.224
ENSP00000371138	FKBP1A	532	0.224
ENSP00000382819	DOM3Z	130	0.225
ENSP00000391349	DOM3Z	306	0.225
ENSP00000348786	RAP1A	296	0.225
ENSP00000265023	KNG1	1819	0.226
ENSP00000209875	CBX5	1478	0.228
ENSP00000339917	TAF9B	131	0.228
ENSP00000299421	ILK	752	0.229
ENSP00000337915	CYP3A4	141	0.23
ENSP00000341344	GGA1	976	0.23
ENSP00000357206	NES	6	0.23

ENSP00000297338	RAD21	95	0.23
ENSP00000333194	RGS19	82	0.23
ENSP00000245451	BMP4	717	0.232
ENSP00000233946	IL1R1	268	0.232
ENSP00000276571	CRH	207	0.233
ENSP00000384665	LPAR2	267	0.233
ENSP00000275603	CCT6A	8	0.235
ENSP00000262188	SMARCD3	122	0.235
ENSP00000354522	TOP1	133	0.235
ENSP00000247933	IDUA	2	0.237
ENSP00000340361	ACVR2B	1	0.24
ENSP00000278916	CHEK1	343	0.24
ENSP00000321259	TALDO1	134	0.24
ENSP00000369055	B4GALT1	266	0.241
ENSP00000301280	CHAF1A	1747	0.241
ENSP00000346659	OCA2	11	0.244
ENSP00000217244	CSNK2A1	803	0.245
ENSP00000307786	CYCS	134	0.245
ENSP00000263038	PHYH	132	0.245
ENSP00000303315	JUNB	30	0.247
ENSP00000276603	TERF1	264	0.248
ENSP00000337736	AKAP1	937	0.249

ENSP00000226359	AFP	3	0.25
ENSP00000366488	PRKACG	3	0.25
ENSP00000314151	KLK3	133	0.251
ENSP00000304350	PRPF8	140	0.251
ENSP00000287497	ITGAM	540	0.252
ENSP00000219070	MMP2	664	0.252
ENSP00000298937	ELP4	133	0.253
ENSP00000411286	GABBR1	1	0.253
ENSP00000342082	SLPI	116	0.253
ENSP00000355812	FGFR1OP	134	0.254
ENSP00000215909	LGALS1	133	0.254
ENSP00000326366	PSEN1	1545	0.255
ENSP00000272348	SNRPG	133	0.255
ENSP00000217961	STS	135	0.255
ENSP00000297564	COX6C	133	0.256
ENSP00000274335	PIK3R1	2891	0.256
ENSP00000391592	PTPN6	521	0.256
ENSP00000356991	PVRL4	133	0.256
ENSP00000270142	SOD1	402	0.257
ENSP00000001008	FKBP4	266	0.258
ENSP00000232014	BCL6	139	0.259
ENSP00000240055	NFYB	132	0.26

ENSP00000362131	RPA4	131	0.26
ENSP00000302707	FPR1	127	0.263
ENSP00000276033	SLC16A2	132	0.263
ENSP00000260010	TLR2	134	0.263
ENSP00000361151	CEL	134	0.265
ENSP00000369889	COL2A1	137	0.265
ENSP00000263967	PIK3CA	585	0.265
ENSP00000359401	C1orf146	1	0.266
ENSP00000264914	ARSB	133	0.269
ENSP00000379204	BMP7	137	0.269
ENSP00000347184	HTT	399	0.269
ENSP00000272167	EPHX1	117	0.27
ENSP00000242208	INHBA	1	0.27
ENSP00000296585	ITGA2	1453	0.27
ENSP00000371432	PRLR	1414	0.271
ENSP00000278616	ATM	2175	0.273
ENSP00000267996	TPM1	668	0.273
ENSP00000344579	FSCB	133	0.274
ENSP00000383558	GCGR	1	0.274
ENSP00000246891	CSN1S1	267	0.276
ENSP00000358022	MCL1	1856	0.276
ENSP00000380702	MYCBP	1205	0.276

ENSP00000382723	AGPAT1	4	0.277
ENSP00000359539	GOT1	1	0.277
ENSP00000259895	GTF2H4	133	0.279
ENSP00000261023	ITGAV	302	0.279
ENSP00000339428	SOCS2	2	0.279
ENSP00000211998	VCL	266	0.28
ENSP00000355325	PSMB5	522	0.281
ENSP00000263025	MAPK3	57	0.284
ENSP00000348551	NCOR2	350	0.284
ENSP00000351446	WDR5	1144	0.284
ENSP00000261783	ARG2	262	0.285
ENSP00000338548	FGF1	1	0.285
ENSP00000303532	DEFB4A	134	0.286
ENSP00000302269	VAV1	1536	0.286
ENSP00000363822	AR	1750	0.288
ENSP00000300161	YWHAB	401	0.288
ENSP00000356070	MAPKAPK2	764	0.289
ENSP00000361636	TNNC2	2	0.29
ENSP00000386284	ALAD	120	0.292
ENSP00000259808	RIPK1	1313	0.292
ENSP00000254998	NXT1	134	0.296
ENSP00000347942	RET	378	0.296

ENSP00000354621	SMURF1	264	0.299
ENSP00000400175	RHOA	1171	0.302
ENSP00000293970	TBC1D24	134	0.302
ENSP00000387699	CREB1	256	0.303
ENSP00000403154	DDAH2	2	0.303
ENSP00000340330	KAT5	2935	0.303
ENSP00000262367	CREBBP	6484	0.304
ENSP00000306497	KCNJ4	4	0.304
ENSP00000369643	GRPR	6	0.305
ENSP00000216341	GZMB	134	0.305
ENSP00000263918	STRN	15	0.305
ENSP00000348554	CDC16	214	0.306
ENSP00000381717	UBE2D2	134	0.306
ENSP00000172229	NGFR	632	0.307
ENSP00000291688	MCM3AP	133	0.31
ENSP00000354826	CALD1	668	0.311
ENSP00000227752	IL10RA	134	0.314
ENSP00000351896	TRAPPC4	268	0.314
ENSP00000374357	ARNTL	1	0.316
ENSP00000296145	TDGF1	132	0.316
ENSP00000296154	CASR	122	0.317
ENSP00000261755	FAH	134	0.317

ENSP00000309845	HRAS	2229	0.319
ENSP00000320940	NCOA1	2631	0.32
ENSP00000243349	ACVR1C	132	0.322
ENSP00000265165	LEF1	1	0.322
ENSP00000338868	PHF8	134	0.322
ENSP00000245479	SOX9	264	0.322
ENSP00000254942	TERF2	710	0.322
ENSP00000261837	GNB5	3	0.323
ENSP00000347719	TBCD	3	0.326
ENSP00000363081	DKK1	132	0.327
ENSP00000364709	F10	134	0.327
ENSP00000245206	GOT2	13	0.327
ENSP00000221972	CD79A	134	0.328
ENSP00000304736	ELOVL6	134	0.328
ENSP00000284981	APP	2341	0.329
ENSP00000367408	CASK	556	0.329
ENSP00000218348	USP11	134	0.329
ENSP00000262887	XRCC1	268	0.329
ENSP00000366396	XRN2	395	0.329
ENSP00000046794	LCP2	478	0.33
ENSP00000283131	SMARCA5	134	0.33
ENSP00000309259	ALAS1	120	0.331

ENSP00000266376	CACNA1C	134	0.331
ENSP00000359663	CD40LG	134	0.331
ENSP00000367830	PRKCZ	134	0.331
ENSP00000216456	VTI1B	133	0.331
ENSP00000285379	CA2	132	0.332
ENSP00000337761	RAB27A	268	0.332
ENSP00000278385	CD44	66	0.333
ENSP00000273588	AMT	10	0.334
ENSP00000335620	GSTA1	2	0.336
ENSP00000322570	POLE	133	0.336
ENSP00000286398	SMC2	133	0.336
ENSP00000384015	SUN1	2	0.338
ENSP00000006275	TRAPPC6A	134	0.338
ENSP00000362649	HDAC1	3543	0.339
ENSP00000264832	ICAM1	15	0.34
ENSP00000350249	POT1	131	0.34
ENSP00000261900	CCNT1	134	0.341
ENSP00000320180	GHRHR	5	0.342
ENSP00000352064	KLRC1	23	0.342
ENSP00000263033	SYTL4	266	0.342
ENSP00000391069	SRPK1	76	0.343
ENSP00000323076	NDUFAF3	132	0.345

ENSP00000296755	MAP1B	4	0.346
ENSP00000363641	TXN	268	0.346
ENSP00000381331	HDAC2	829	0.347
ENSP00000297268	COL1A2	137	0.348
ENSP00000262613	SLC9A3R1	3481	0.348
ENSP00000310551	LCLAT1	1	0.349
ENSP00000315442	NR1H4	134	0.35
ENSP00000378191	AIMP1	1	0.351
ENSP00000265517	MTTP	2	0.351
ENSP00000358994	MYO6	8	0.351
ENSP00000342952	ADCY2	32	0.352
ENSP00000349465	PICK1	137	0.352
ENSP00000406037	KAT8	134	0.354
ENSP00000311113	JUP	392	0.357
ENSP00000350616	DDC	390	0.358
ENSP00000307387	PDCD6IP	1477	0.361
ENSP00000299402	APBB1	1442	0.362
ENSP00000223029	AIMP2	1343	0.363
ENSP00000335304	DLST	137	0.363
ENSP00000270861	PLK4	1	0.363
ENSP00000305958	STIP1	133	0.365
ENSP00000222256	RAB3A	802	0.367

ENSP00000303977	INO80E	134	0.368
ENSP00000338934	EZR	3127	0.369
ENSP00000337224	LRAT	3	0.369
ENSP00000313350	RNASEH1	1	0.37
ENSP00000405934	ITPR1	402	0.373
ENSP00000312435	DAG1	1206	0.374
ENSP00000288135	KIT	134	0.374
ENSP00000352138	KIRREL	491	0.375
ENSP00000205402	DLD	360	0.377
ENSP00000381607	GSTP1	219	0.377
ENSP00000370343	IRF4	5	0.379
ENSP00000345731	DLG1	267	0.381
ENSP00000350720	SMARCA4	1375	0.385
ENSP00000265094	FBXW11	13	0.387
ENSP00000294304	LRP5	132	0.387
ENSP00000237527	GHRH	5	0.389
ENSP00000299518	IDH3A	1	0.389
ENSP00000316029	TLN1	2	0.39
ENSP00000313391	DAB2	2	0.391
ENSP00000301364	TSR1	1	0.394
ENSP00000288602	BRAF	131	0.396
ENSP00000264018	DDX6	131	0.396

ENSP00000223095	SERPINE1	605	0.397
ENSP00000322542	GTF2I	23	0.399
ENSP00000356587	NPHS2	625	0.399
ENSP00000357674	SNAPIN	1	0.399
ENSP00000257192	DSG1	115	0.401
ENSP00000254227	NR0B2	133	0.402
ENSP00000329357	SP1	1842	0.402
ENSP00000379625	MYD88	536	0.403
ENSP00000324248	PENK	74	0.403
ENSP00000261205	SYT1	988	0.403
ENSP00000323929	A2M	1	0.404
ENSP00000354394	STAT1	2155	0.405
ENSP00000332643	NDN	58	0.406
ENSP00000361878	CAP1	1	0.407
ENSP00000410321	LY6G5C	1	0.408
ENSP00000085219	CD22	25	0.409
ENSP00000351997	MAP2K6	642	0.409
ENSP00000264972	ZAP70	126	0.409
ENSP00000387942	SAPCD1	1	0.41
ENSP00000354782	CD247	133	0.412
ENSP00000287820	PPARG	278	0.413
ENSP00000339109	ANAPC1	1681	0.414

ENSP00000306614	PPIH	81	0.414
ENSP00000307188	ASL	2	0.415
ENSP00000351490	MAX	134	0.417
ENSP00000265335	RAD50	676	0.418
ENSP00000345206	RBPJ	402	0.418
ENSP00000242577	DYNLL1	1340	0.421
ENSP00000303242	ITGB2	669	0.422
ENSP00000361405	MMP9	192	0.424
ENSP00000373614	SELPLG	134	0.424
ENSP00000360031	RRP12	1	0.425
ENSP00000358335	MAP3K7	642	0.426
ENSP00000252444	LDLR	286	0.427
ENSP00000315644	TYMS	3387	0.43
ENSP00000356438	PTGS2	1475	0.431
ENSP00000256474	VHL	7840	0.431
ENSP00000241416	ACVR2A	1	0.433
ENSP00000417764	ALG2	268	0.433
ENSP00000299424	TAF10	131	0.434
ENSP00000353452	MYLK	802	0.436
ENSP00000265641	CPT1A	18	0.437
ENSP00000363763	EPHB2	15	0.438
ENSP00000231454	IL5	1	0.438

ENSP00000348827	THRB	5	0.438
ENSP00000374280	RTF1	133	0.439
ENSP00000267415	TINF2	194	0.44
ENSP00000316578	SUZ12	133	0.441
ENSP00000379330	NFATC2	153	0.442
ENSP00000357301	RXFP4	120	0.443
ENSP00000329380	GP1BA	1963	0.444
ENSP00000179259	C12orf5	134	0.445
ENSP00000408236	CYTH2	268	0.445
ENSP00000261819	ANAPC5	5	0.446
ENSP00000011653	CD4	2909	0.446
ENSP00000231449	IL4	1	0.447
ENSP00000263088	PLD2	267	0.448
ENSP00000346389	MEF2A	7	0.45
ENSP00000378529	FZR1	151	0.451
ENSP00000363851	EDA2R	128	0.452
ENSP00000367934	UQCRQ	1	0.453
ENSP00000257904	CDK4	752	0.454
ENSP00000339393	CCR6	134	0.455
ENSP00000269571	ERBB2	1462	0.455
ENSP00000355747	PSEN2	132	0.458
ENSP00000328777	EFNA5	6	0.461

ENSP00000308541	F2	1437	0.461
ENSP00000364893	ARHGEF7	166	0.463
ENSP00000396219	MEF2C	133	0.465
ENSP00000306512	IL8	1384	0.467
ENSP00000215832	MAPK1	2028	0.467
ENSP00000261479	PSMA6	9	0.47
ENSP00000321853	SERPINF2	3	0.471
ENSP00000354791	DCTN1	267	0.472
ENSP00000363921	PARD3	305	0.473
ENSP00000265709	ANK1	265	0.476
ENSP00000399968	NCOA2	1	0.478
ENSP00000380252	NFE2L2	82	0.482
ENSP00000313681	SPHK1	134	0.483
ENSP00000223129	RPA3	131	0.487
ENSP00000408005	SLC9A3R2	267	0.488
ENSP00000369871	HAUS6	134	0.489
ENSP00000382373	DAZL	2	0.49
ENSP00000380378	PAFAH1B1	3	0.491
ENSP00000309629	CFL1	2	0.493
ENSP00000370571	TH	390	0.493
ENSP00000251968	TSG101	1651	0.494
ENSP00000203407	UQCRC1	1	0.499

ENSP00000361021	PTEN	266	0.502
ENSP00000262477	RABEP1	818	0.502
ENSP00000339353	CPSF1	134	0.503
ENSP00000287766	SLC6A1	258	0.504
ENSP00000308741	CLOCK	126	0.508
ENSP00000356505	NCF2	134	0.51
ENSP00000353059	APAF1	134	0.512
ENSP00000009589	RPS20	134	0.513
ENSP00000332258	DGAT1	5	0.514
ENSP00000343745	DICER1	134	0.514
ENSP00000381282	VIMP	133	0.514
ENSP00000296871	CSF2	242	0.517
ENSP00000226574	NFKB1	13	0.517
ENSP00000232461	GNAT1	134	0.518
ENSP00000300738	RRM1	47	0.518
ENSP00000352264	CD2AP	625	0.52
ENSP00000007708	PDK2	137	0.52
ENSP00000316176	UBE2N	134	0.52
ENSP00000344936	PTTG1	134	0.524
ENSP00000361359	CD40	134	0.526
ENSP00000317955	EEA1	6	0.526
ENSP00000226730	IL2	147	0.527

ENSP00000358997	IRAK1	1058	0.528
ENSP00000351908	MAP3K5	606	0.528
ENSP00000316377	DLGAP1	259	0.53
ENSP00000400591	SNRPE	16	0.53
ENSP00000309503	YWHAZ	2001	0.53
ENSP00000220592	AGO2	134	0.536
ENSP00000333982	NDEL1	137	0.536
ENSP00000284957	RABGEF1	812	0.537
ENSP00000200135	ZW10	2	0.537
ENSP00000358105	APH1A	132	0.54
ENSP00000338130	KLRD1	23	0.541
ENSP00000348784	IGBP1	134	0.542
ENSP00000243077	LRP1	40	0.542
ENSP00000262027	MARS	1	0.545
ENSP00000263621	ELANE	116	0.546
ENSP00000318861	SF3B2	120	0.547
ENSP00000360141	GNAS	110	0.548
ENSP00000314004	ANAPC2	70	0.549
ENSP00000216951	GSS	1	0.549
ENSP00000391901	PHF1	134	0.549
ENSP00000319060	CAMK2G	113	0.552
ENSP00000304669	CTNNA1	152	0.553

ENSP00000237837	FGF23	265	0.555
ENSP00000267163	RB1	3603	0.556
ENSP00000298139	WRN	34	0.558
ENSP00000257899	BLOC1S1	2	0.56
ENSP00000356425	UCHL5	250	0.561
ENSP00000291700	S100B	266	0.562
ENSP00000222673	OGDH	4	0.563
ENSP00000219240	DHODH	1	0.564
ENSP00000349049	KDM1A	132	0.564
ENSP00000254066	RARA	194	0.566
ENSP00000386884	CXCR4	263	0.57
ENSP00000008527	CRY1	125	0.573
ENSP00000350941	SRC	7603	0.573
ENSP00000337759	DOM3Z	5	0.574
ENSP00000363998	ITCH	260	0.574
ENSP00000324806	GSK3B	1957	0.576
ENSP00000364133	TGFBR1	1797	0.577
ENSP00000363512	ALOX5	88	0.578
ENSP00000259512	DERL1	133	0.58
ENSP00000349437	IGF2R	163	0.587
ENSP00000367439	RPP38	2	0.588
ENSP00000370023	HADHA	1	0.589

ENSP00000234396	ATP6V1B1	133	0.59
ENSP00000245960	CDC25B	257	0.59
ENSP00000251849	RAF1	2820	0.591
ENSP00000334448	GNG2	138	0.596
ENSP00000232607	UMPS	1	0.598
ENSP00000316328	CIITA	6	0.6
ENSP00000289779	F11R	46	0.6
ENSP00000052754	DCN	532	0.601
ENSP00000360286	RAE1	268	0.601
ENSP00000221930	TGFB1	1174	0.602
ENSP00000342056	CS	6	0.607
ENSP00000343204	JAK1	651	0.607
ENSP00000216254	ACO2	1	0.609
ENSP00000351665	CLIP1	267	0.612
ENSP00000264515	RBBP5	11	0.612
ENSP00000348307	SIRPA	492	0.612
ENSP00000312122	SEC13	134	0.613
ENSP00000302728	GUSB	132	0.614
ENSP00000262158	SMAD7	928	0.62
ENSP00000303019	GPHN	134	0.621
ENSP00000216797	NFKBIA	117	0.623
ENSP00000246747	ARL2	1	0.624

ENSP00000352516	DNMT1	189	0.624
ENSP00000337825	LCK	3468	0.625
ENSP00000224337	BLNK	134	0.626
ENSP00000336740	LIMK1	2	0.626
ENSP00000384708	FSHR	30	0.627
ENSP00000368438	PCNA	6869	0.628
ENSP00000287727	ZFYVE9	492	0.631
ENSP00000367872	GNB1	9	0.632
ENSP00000262340	RPE65	1	0.633
ENSP00000382004	CTNND1	308	0.636
ENSP00000235090	WDR77	2331	0.636
ENSP00000254657	PER2	125	0.637
ENSP00000379110	CXCL1	132	0.639
ENSP00000244051	MOCS3	1	0.639
ENSP00000338983	MUC1	1070	0.641
ENSP00000314813	OAZ1	132	0.642
ENSP00000254122	FSHB	35	0.643
ENSP00000354876	MT-CO2	265	0.643
ENSP00000384675	SOS1	272	0.645
ENSP00000384053	CSF2RB	242	0.646
ENSP00000373715	DCP2	131	0.648
ENSP00000379933	TPI1	266	0.649

ENSP00000219255	PARD6A	310	0.65
ENSP00000410732	GABRG2	1	0.652
ENSP00000340698	GIPC1	76	0.653
ENSP00000314214	VAMP2	532	0.654
ENSP00000220584	FDFT1	132	0.655
ENSP00000308176	BTK	20	0.656
ENSP00000358595	CGA	167	0.656
ENSP00000367910	FANCG	134	0.657
ENSP00000361066	NCOA3	54	0.658
ENSP00000359074	L1CAM	262	0.659
ENSP00000316032	NUP98	134	0.659
ENSP00000350275	HIST1H3A	343	0.66
ENSP00000263774	NDUFS3	132	0.662
ENSP00000263754	KAT2B	169	0.664
ENSP00000337040	UNC119	1	0.665
ENSP00000229854	MCM3	142	0.666
ENSP00000372793	LTA	77	0.667
ENSP00000359301	MAGEA3	129	0.667
ENSP00000382177	MYO5A	2	0.668
ENSP00000355493	ADSS	5	0.669
ENSP00000216223	IL2RB	225	0.67
ENSP00000355759	PARP1	268	0.671

ENSP00000311344	PPP2R1B	29	0.674
ENSP00000337014	HFE2	1	0.676
ENSP00000352514	RUNX2	264	0.68
ENSP00000351273	CASP8	16	0.681
ENSP00000356248	PTPN7	133	0.686
ENSP00000302564	BCL2L1	182	0.688
ENSP00000358716	DDX20	134	0.689
ENSP00000348877	GPI	266	0.69
ENSP00000005257	RALA	155	0.693
ENSP00000290649	AMFR	132	0.697
ENSP00000317714	STX4	400	0.697
ENSP00000264246	CD80	22	0.701
ENSP00000253004	ASS1	5	0.703
ENSP00000233146	MSH2	134	0.703
ENSP00000308533	GEMIN2	134	0.704
ENSP00000265056	MCM2	4	0.704
ENSP00000314458	CDC42	1559	0.707
ENSP00000261733	ALDH2	2	0.708
ENSP00000272190	REN	132	0.709
ENSP00000357858	BUB3	2274	0.711
ENSP00000227524	PRPF19	176	0.712
ENSP00000357292	UBQLN4	2	0.718

ENSP00000295797	PRKCI	170	0.72
ENSP00000254719	RPA1	127	0.721
ENSP00000358554	BCAS2	27	0.723
ENSP00000384273	RELA	1356	0.724
ENSP00000288986	NCK1	268	0.725
ENSP00000367207	MYC	2338	0.726
ENSP00000356346	PTPRC	33	0.726
ENSP00000252486	APOE	28	0.727
ENSP00000334188	PFDN5	133	0.727
ENSP00000262735	PPARA	278	0.729
ENSP00000244769	ATXN1	133	0.732
ENSP00000264705	CAD	1	0.742
ENSP00000358622	IKBKG	134	0.743
ENSP00000375777	STRN4	133	0.743
ENSP00000315791	CSTF3	134	0.744
ENSP00000245414	IRF1	144	0.744
ENSP00000361850	PLAU	10	0.746
ENSP00000261461	PPP2R5A	237	0.749
ENSP00000262160	SMAD2	1065	0.749
ENSP00000251810	RRM2B	47	0.75
ENSP00000265838	ACAT1	1	0.752
ENSP00000223023	WASL	1274	0.752

ENSP00000253792	ACLY	133	0.753
ENSP00000419851	GMPS	251	0.753
ENSP00000349467	CALM1	2535	0.755
ENSP00000324804	PPP2R1A	153	0.755
ENSP00000404121	ILF3	279	0.759
ENSP00000274255	SKP2	1377	0.759
ENSP00000342793	PLD1	158	0.762
ENSP00000216194	ADSL	5	0.766
ENSP00000356671	SERPINC1	134	0.766
ENSP00000284811	TCEB1	264	0.766
ENSP00000217109	CSTF1	134	0.767
ENSP00000347858	XIAP	135	0.768
ENSP00000263864	VAMP8	133	0.771
ENSP00000350283	BRCA1	3055	0.773
ENSP00000349960	ACTB	1	0.774
ENSP00000356918	STX7	133	0.774
ENSP00000290541	PSMB4	132	0.777
ENSP00000297494	NOS3	2602	0.778
ENSP00000221413	RUVBL2	18	0.78
ENSP00000352929	CSNK1E	125	0.783
ENSP00000243776	CHPF	3	0.786
ENSP00000355518	FH	2	0.786

ENSP00000317904	GYS1	268	0.786
ENSP00000362361	CDK9	418	0.791
ENSP00000316042	HNRNPA0	66	0.791
ENSP00000304592	FASN	400	0.792
ENSP00000256857	GRP	274	0.8
ENSP00000262435	SMURF2	9	0.8
ENSP00000382595	PAICS	5	0.803
ENSP00000225655	PFN1	1	0.807
ENSP00000363313	PRPF4	3	0.809
ENSP00000229022	VDR	614	0.81
ENSP00000228872	CDKN1B	726	0.813
ENSP00000329411	IRF7	4	0.814
ENSP00000264606	HDAC4	3	0.815
ENSP00000313007	PABPC1	124	0.817
ENSP00000269300	PIK3R5	121	0.817
ENSP00000413493	CPSF3L	2	0.82
ENSP00000381064	INTS10	16	0.822
ENSP00000367797	SKI	4	0.824
ENSP00000326550	TACC3	3	0.824
ENSP00000253856	ATP6V0A4	533	0.825
ENSP00000355261	SMG5	3	0.828
ENSP00000216225	RBX1	1025	0.833

ENSP00000260947	BARD1	134	0.834
ENSP00000258962	SRSF1	78	0.838
ENSP00000369497	BRCA2	225	0.839
ENSP00000343535	USP7	385	0.839
ENSP00000407401	PEX5	1	0.842
ENSP00000358812	PDCD11	1	0.844
ENSP00000358541	SIKE1	85	0.844
ENSP00000309103	BAD	50	0.845
ENSP00000352121	PIK3CG	121	0.847
ENSP00000229239	GAPDH	266	0.849
ENSP00000173229	NTN1	3	0.849
ENSP00000334564	POLR3C	2	0.852
ENSP00000230354	TBP	1019	0.858
ENSP00000327070	MDH2	10	0.859
ENSP00000354476	SREBF2	266	0.86
ENSP00000300935	RAB8A	2	0.861
ENSP00000309166	RBM4	3	0.863
ENSP00000256443	CDK7	137	0.864
ENSP00000356087	IKBKE	70	0.864
ENSP00000300413	SNRPD1	134	0.867
ENSP00000295897	ALB	2091	0.868
ENSP00000419692	RXRA	252	0.872

ENSP00000215071	PSMD8	2	0.873
ENSP00000420168	GSTA2	1	0.874
ENSP00000285814	MKI67IP	1	0.874
ENSP00000273047	RAB5A	6	0.875
ENSP00000355153	CDKN2A	132	0.876
ENSP00000269397	CBX4	133	0.882
ENSP00000336790	ATF4	264	0.883
ENSP00000311032	CASP3	139	0.883
ENSP00000418447	PPP2CA	1206	0.884
ENSP00000229769	FANCE	8	0.885
ENSP00000343054	RBM5	168	0.886
ENSP00000302620	AGXT	1	0.888
ENSP00000303939	CTLA4	112	0.89
ENSP00000335153	HSP90AA1	5101	0.89
ENSP00000262238	YY1	225	0.89
ENSP00000330237	CASP9	1	0.893
ENSP00000351894	NCOA6	11	0.893
ENSP00000260762	EXOC6	2	0.896
ENSP00000324890	CD28	22	0.898
ENSP00000388526	HLA-A	129	0.899
ENSP00000355890	EPRS	132	0.901
ENSP00000269321	ARHGDIA	221	0.902

ENSP00000212015	SIRT1	126	0.902
ENSP00000298316	ARF6	545	0.903
ENSP00000397552	ACTL6A	24	0.906
ENSP00000225916	KAT2A	4	0.907
ENSP00000344352	ATF3	264	0.909
ENSP00000365891	WAS	276	0.916
ENSP00000401980	MAVS	134	0.92
ENSP00000364649	SDHB	2	0.921
ENSP00000313420	PRKDC	404	0.922
ENSP00000310127	IRF3	70	0.927
ENSP00000369213	DDX58	134	0.93
ENSP00000326804	CUL1	82	0.932
ENSP00000381339	GNAT3	2	0.933
ENSP00000260402	PLCB2	2	0.936
ENSP00000357879	PSMD4	269	0.936
ENSP00000374455	SQSTM1	295	0.936
ENSP00000389934	EXOC5	2	0.939
ENSP00000362592	RBBP4	268	0.939
ENSP00000299543	CTDP1	139	0.94
ENSP00000345571	E2F1	229	0.94
ENSP00000227758	BIRC2	133	0.943
ENSP00000352980	HIST1H4A	274	0.943

ENSP00000287647	FANCD2	32	0.946
ENSP00000302967	HDAC3	138	0.948
ENSP00000352400	NUP214	393	0.949
ENSP00000347733	TRRAP	4	0.949
ENSP00000271628	SF3B4	4	0.95
ENSP00000268182	IQGAP1	117	0.951
ENSP00000227378	HSPA8	1404	0.954
ENSP00000343274	INTS8	65	0.954
ENSP00000323050	RBBP8	65	0.955
ENSP00000361418	IPO13	61	0.959
ENSP00000217958	PSMD10	33	0.959
ENSP00000294172	NXF1	402	0.96
ENSP00000215587	POLR2E	158	0.96
ENSP00000332468	TRAF3	133	0.96
ENSP00000303088	POLR3D	2	0.962
ENSP00000351777	VCP	133	0.962
ENSP00000329623	BCL2	507	0.963
ENSP00000359300	CETN2	1	0.963
ENSP00000368880	FOXO1	1	0.964
ENSP00000371236	GART	5	0.966
ENSP00000325448	KARS	132	0.968
ENSP00000348461	RAC1	1945	0.968

ENSP00000230449	EXOC2	2	0.969
ENSP00000315702	MOB4	192	0.973
ENSP00000366013	GNB2L1	40	0.976
ENSP00000249299	NAA38	48	0.979
ENSP00000216605	MTHFD1	5	0.98
ENSP00000354554	MT-CYB	265	0.982
ENSP00000280665	DCP1B	15	0.983
ENSP00000354961	MT-ND4	264	0.983
ENSP00000223321	PSMA2	3	0.983
ENSP00000225577	RPS6KB1	1	0.983
ENSP00000350512	COPS5	229	0.984
ENSP00000292644	PSMC2	4022	0.984
ENSP00000369757	RPS6	1	0.984
ENSP00000268058	PML	100	0.985
ENSP00000377141	ARRB1	2318	0.986
ENSP00000296271	RHO	2254	0.986
ENSP00000359727	BAG2	78	0.987
ENSP00000285021	XPC	1	0.987
ENSP00000344818	UBC	83969	0.988
ENSP00000339007	GRB2	3355	0.989
ENSP00000314949	POLR2A	466	0.991
ENSP00000350708	RAD23B	1	0.991

ENSP00000247668	TRAF2	814	0.991
ENSP00000342374	SNRPD2	12	0.992
ENSP00000248566	SHFM1	1700	0.993
ENSP00000162749	TNFRSF1A	84	0.993
ENSP00000317159	CYC1	1	0.994
ENSP00000339151	IKBKB	1	0.994
ENSP00000362820	SRSF3	3	0.995
ENSP00000262803	UPF1	3	0.997
ENSP00000270202	AKT1	7608	0.998
ENSP00000316879	EIF4G1	144	0.999
ENSP00000414634	LSM2	79	0.999
ENSP00000350877	SRSF2	3	0.999
ENSP00000348708	UPF2	127	0.999
ENSP00000263309	CLNS1A	256	1
ENSP00000358563	DKC1	133	1
ENSP00000269349	EIF4A3	12	1
ENSP00000366135	EXOSC10	823	1
ENSP00000398597	EXOSC6	59	1
ENSP00000374354	EXOSC8	6	1
ENSP00000365439	HNRNPK	677	1
ENSP00000313829	KHDRBS1	884	1
ENSP00000310596	LSM1	15	1

ENSP00000252622	LSM7	160	1
ENSP00000417281	MDM2	2783	1
ENSP00000252102	NDUFA2	530	1
ENSP00000312735	POLR2B	166	1
ENSP00000319169	PRMT5	259	1
ENSP00000396127	RAN	384	1
ENSP00000348577	RANGAP1	389	1
ENSP00000363676	RPL11	133	1
ENSP00000221494	SF3A2	548	1
ENSP00000215829	SNRPD3	255	1
ENSP00000314491	SRRT	177	1
ENSP00000240185	TARDBP	1260	1
ENSP00000291552	U2AF1	6	1
ENSP00000307863	U2AF2	492	1
ENSP00000324897	UBE2I	564	1
ENSP00000276201	UPF3B	18	1
ENSP00000264951	XRN1	487	1
ENSP00000361626	YBX1	2	1

4. Candidate genes for microRNA target genes and somatic mutation genes

Ensembl ID	Gene symbol	betweenness	Permutation FDR
ENSP00000020926	SYT13	2	<0.001
ENSP00000222399	LAMB1	792	<0.001

ENSP00000230538	LAMA4	2	<0.001
ENSP00000261722	AP3B2	1	<0.001
ENSP00000265071	CDH6	800	<0.001
ENSP00000265335	RAD50	3568	<0.001
ENSP00000266579	SLC38A4	1	<0.001
ENSP00000267845	HDC	983	<0.001
ENSP00000270233	BCAM	2	<0.001
ENSP00000281708	FBXW7	1320	<0.001
ENSP00000296585	ITGA2	5281	<0.001
ENSP00000297268	COL1A2	2577	<0.001
ENSP00000325123	ZSCAN2	2	<0.001
ENSP00000329748	CPNE8	1	<0.001
ENSP00000330633	CNTN2	1556	<0.001
ENSP00000332151	DSE	2	<0.001
ENSP00000338217	ZNF532	1	<0.001
ENSP00000341170	PTN	800	<0.001
ENSP00000348444	TTN(titin)	3248	<0.001
ENSP00000350941	SRC	13874	<0.001
ENSP00000351486	NTRK1	6096	<0.001
ENSP00000356115	MFSD4	3	<0.001
ENSP00000358525	NGF	4923	<0.001
ENSP00000371138	FKBP1A	3140	<0.001

ENSP00000401303	SHC1	6291	<0.001
ENSP00000406027	EPM2AIP1	1	<0.001
ENSP00000412283	IER3	1	<0.001
ENSP00000023939	RTFDC1	1	0.001
ENSP00000225893	HNF1B	987	0.001
ENSP00000254942	TERF2	2914	0.001
ENSP00000265709	ANK1	2403	0.001
ENSP00000268171	FURIN	1787	0.001
ENSP00000276603	TERF1	1790	0.001
ENSP00000282041	EPG5	1	0.001
ENSP00000304669	CTNNA1	3384	0.001
ENSP00000312435	DAG1	4162	0.001
ENSP00000320324	NPEPPS	1	0.001
ENSP00000322898	EBF1	1599	0.001
ENSP00000337839	ZCCHC7	1	0.001
ENSP00000338785	STARD13	952	0.001
ENSP00000340698	GIPC1	1739	0.001
ENSP00000344456	CTNNB1	13813	0.001
ENSP00000359074	L1CAM	3182	0.001
ENSP00000363360	INIP	799	0.001
ENSP00000367651	PRDM16	1	0.001
ENSP00000370408	CDX2	989	0.001

ENSP00000372313	MSLN	800	0.001
ENSP00000215862	MORC2	1	0.002
ENSP00000228307	PXN	2070	0.002
ENSP00000242839	ATP7B	800	0.002
ENSP00000255448	DCLK1	1	0.002
ENSP00000293379	ITGA5	5785	0.002
ENSP00000297494	NOS3	7352	0.002
ENSP00000313084	EGFLAM	1	0.002
ENSP00000322020	SLC25A22	1	0.002
ENSP00000344468	SDC3	800	0.002
ENSP00000348634	MYH6	798	0.002
ENSP00000349960	ACTB	1393	0.002
ENSP00000352657	ME3	797	0.002
ENSP00000361850	PLAU	1785	0.002
ENSP00000372160	DOK6	800	0.002
ENSP00000375238	KRTAP2-1	1	0.002
ENSP00000382193	MYBPC3	803	0.002
ENSP00000383210	NEK3	798	0.002
ENSP00000400223	FJX1	3	0.002
ENSP00000261681	MPP5	795	0.003
ENSP00000262053	ATF1	969	0.003
ENSP00000286621	ADK	800	0.003

ENSP00000351209	EPHA2	2152	0.003
ENSP00000353557	SLC35F1	2	0.003
ENSP00000366563	PIK3CD	973	0.003
ENSP00000367714	HES5	800	0.003
ENSP00000379457	FAF1	799	0.003
ENSP00000162330	BCAR1	2263	0.004
ENSP00000233813	IGFBP5	800	0.004
ENSP00000351141	WTAP	191	0.004
ENSP00000360200	INADL	799	0.004
ENSP00000360310	SPO11	800	0.004
ENSP00000363642	BMS1	2	0.004
ENSP00000245323	EFNB2	990	0.005
ENSP00000256443	CDK7	2108	0.005
ENSP00000265362	SEMA3A	799	0.005
ENSP00000298310	NEMF	1	0.005
ENSP00000303634	LRP8	61	0.005
ENSP00000339390	CDH26	1	0.005
ENSP00000343313	ATG5	990	0.005
ENSP00000357392	EFNA1	1177	0.005
ENSP00000363727	STARD8	1	0.005
ENSP00000204615	THPO	191	0.006
ENSP00000222792	CHN2	191	0.006

ENSP00000295709	STK36	798	0.006
ENSP00000316854	ATOX1	990	0.006
ENSP00000320838	GSG1	191	0.006
ENSP00000332353	PTCH1	3399	0.006
ENSP00000352834	MYO1C	2	0.006
ENSP00000377941	ACTN1	990	0.006
ENSP00000228841	MYL2	984	0.007
ENSP00000254661	RAMP1	191	0.007
ENSP00000262188	SMARCD3	952	0.007
ENSP00000262219	ANXA13	1	0.007
ENSP00000268182	IQGAP1	615	0.007
ENSP00000302397	ATP1A3	5	0.007
ENSP00000312185	ELMO1	800	0.007
ENSP00000326432	CCR8	191	0.007
ENSP00000345656	VAPA	982	0.007
ENSP00000349467	CALM1	6884	0.007
ENSP00000350990	TNKS1BP1	191	0.007
ENSP00000357656	FYN	8142	0.007
ENSP00000372547	SRY	800	0.007
ENSP00000228641	MYF6	191	0.008
ENSP00000242577	DYNLL1	3547	0.008
ENSP00000254691	CARD6	191	0.008

ENSP00000255882	PI4KA	1	0.008
ENSP00000338934	EZR	5459	0.008
ENSP00000340688	LPHN1	190	0.008
ENSP00000358994	MYO6	988	0.008
ENSP00000363799	ACTL7B	1	0.008
ENSP00000366603	TGOLN2	191	0.008
ENSP00000406359	HSPA1A	191	0.008
ENSP00000307549	NPTX1	191	0.009
ENSP00000309622	TFDP2	191	0.009
ENSP00000312235	MUC13	4	0.009
ENSP00000328777	EFNA5	989	0.009
ENSP00000332592	SPAG16	986	0.009
ENSP00000354541	NLGN1	998	0.009
ENSP00000250111	ATP1B2	191	0.01
ENSP00000252456	CNN1	793	0.01
ENSP00000267415	TINF2	1446	0.01
ENSP00000287322	BAG4	176	0.01
ENSP00000349955	RPRD1A	191	0.01
ENSP00000247026	NSRP1	191	0.011
ENSP00000275603	CCT6A	191	0.011
ENSP00000300134	STAT6	2303	0.011
ENSP00000303522	TACR1	799	0.011

ENSP00000310723	DDX23	170	0.011
ENSP00000356641	RFWD2	794	0.011
ENSP00000358323	TXNIP	988	0.011
ENSP00000367486	MEIG1	798	0.011
ENSP00000231228	IL12B	191	0.012
ENSP00000308176	BTK	1008	0.012
ENSP00000317872	RBBP6	191	0.012
ENSP00000329715	DRG1	191	0.012
ENSP00000332504	CCR10	1	0.012
ENSP00000334940	GGN	191	0.012
ENSP00000352336	PLCG2	2	0.012
ENSP00000354777	TBKBP1	191	0.012
ENSP00000356652	CACYBP	2	0.012
ENSP00000358099	RGS10	1	0.012
ENSP00000203556	GMIP	1	0.013
ENSP00000265164	CASP6	191	0.013
ENSP00000309595	C10orf2	796	0.013
ENSP00000337194	PRPF4B	191	0.013
ENSP00000346886	GABPA	191	0.013
ENSP00000359297	NSDHL	980	0.013
ENSP00000360689	TNKS2	799	0.013
ENSP00000385450	MAGI1	796	0.013

ENSP00000223023	WASL	3683	0.014
ENSP00000262613	SLC9A3R1	5563	0.014
ENSP00000316460	FYB	957	0.014
ENSP00000341848	GOLGB1	191	0.014
ENSP00000357674	SNAPIN	197	0.014
ENSP00000364133	TGFBR1	4272	0.014
ENSP00000406674	MOGAT1	1	0.014
ENSP00000240652	IAPP	191	0.015
ENSP00000263856	CHMP3	1	0.015
ENSP00000313581	KLK2	191	0.015
ENSP00000365766	TIMM17B	191	0.015
ENSP00000258341	LAMC1	796	0.016
ENSP00000313391	DAB2	947	0.016
ENSP00000331327	WT1	989	0.016
ENSP00000360891	IFIT2	191	0.016
ENSP00000206249	ESR1	18453	0.017
ENSP00000252034	ELN	989	0.017
ENSP00000291525	TFF3	793	0.017
ENSP00000305595	B3GNT2	191	0.017
ENSP00000340507	TRIM24	191	0.017
ENSP00000351363	MSMB	191	0.017
ENSP00000254351	SDC1	798	0.018

ENSP00000277541	NOTCH1	5845	0.018
ENSP00000337397	DKFZP686J19100	799	0.018
ENSP00000347710	OPHN1	190	0.018
ENSP00000363533	MDH1B	1	0.018
ENSP00000220478	SCG3	1	0.019
ENSP00000261783	ARG2	1145	0.019
ENSP00000353731	DPP4	977	0.019
ENSP00000359506	FMR1	1105	0.019
ENSP00000200181	ITGB4	989	0.02
ENSP00000216410	GNPNAT1	190	0.02
ENSP00000348168	GTF2E2	789	0.02
ENSP00000226091	EFNB3	191	0.021
ENSP00000335153	HSP90AA1	8635	0.021
ENSP00000360916	VAV2	1308	0.021
ENSP00000364398	HABP4	191	0.021
ENSP00000394033	KCNK2	191	0.021
ENSP00000266085	TIMP3	191	0.022
ENSP00000349204	CRB3	795	0.022
ENSP00000354033	PCGF2	191	0.022
ENSP00000357692	S100A16	191	0.022
ENSP00000218652	NDFIP2	190	0.023
ENSP00000227618	ANAPC15	191	0.023

ENSP00000363021	RPA2	979	0.023
ENSP00000367747	PLCH2	190	0.023
ENSP00000385021	FANCL	191	0.023
ENSP00000386171	ESRRG	191	0.023
ENSP00000257068	MTNR1B	16	0.024
ENSP00000261507	MSMO1	978	0.024
ENSP00000288986	NCK1	1504	0.024
ENSP00000345206	RBPJ	1886	0.024
ENSP00000348089	ERCC6	772	0.024
ENSP00000355566	TOMM20	382	0.024
ENSP00000252575	NCAN	4	0.025
ENSP00000324549	CYFIP1	1103	0.025
ENSP00000375809	ERCC2	977	0.025
ENSP00000264499	BBS7	382	0.026
ENSP00000285398	ERCC3	187	0.026
ENSP00000296802	NSA2	1	0.026
ENSP00000308549	ADORA1	190	0.026
ENSP00000361125	VEGFA	3113	0.026
ENSP00000362744	RPS4X	190	0.026
ENSP00000384675	SOS1	836	0.026
ENSP00000225298	UTP18	2	0.027
ENSP00000240874	KALRN	191	0.027

ENSP00000250894	MAPK8IP3	191	0.027
ENSP00000293831	EIF4A1	382	0.027
ENSP00000305913	COL8A2	197	0.027
ENSP00000365811	SPAG6	191	0.027
ENSP00000374372	SPTB	804	0.027
ENSP00000256689	SLC38A2	189	0.028
ENSP00000314458	CDC42	4161	0.028
ENSP00000352514	RUNX2	1781	0.028
ENSP00000362058	NDUFS5	797	0.028
ENSP00000274711	LRRTM2	190	0.029
ENSP00000361777	SET	799	0.029
ENSP00000400591	SNRPE	219	0.029
ENSP00000266427	ETV6	191	0.03
ENSP00000297562	AP5Z1	191	0.03
ENSP00000319060	CAMK2G	969	0.03
ENSP00000347198	SRGAP1	979	0.03
ENSP00000358022	MCL1	2927	0.03
ENSP00000361965	ADA	788	0.03
ENSP00000371682	DCAF16	196	0.03
ENSP00000403557	PPP1R11	191	0.03
ENSP00000404232	EFHC2	191	0.03
ENSP00000302234	CCL11	972	0.031

ENSP00000311579	TNKS	191	0.031
ENSP00000356529	RGS16	191	0.031
ENSP00000386896	ITGA6	1011	0.031
ENSP00000266376	CACNA1C	864	0.032
ENSP00000299163	HIF1AN	191	0.032
ENSP00000309968	ADAM17	191	0.032
ENSP00000315173	ZNF41	190	0.032
ENSP00000334594	SLC10A7	191	0.032
ENSP00000339007	GRB2	7741	0.032
ENSP00000346440	TCF4	191	0.032
ENSP00000272233	RHOB	191	0.033
ENSP00000286548	GNAQ	769	0.033
ENSP00000299339	CLDN10	190	0.033
ENSP00000303423	FNTA	191	0.033
ENSP00000339861	ENY2	793	0.033
ENSP00000354376	RAB25	191	0.033
ENSP00000361405	MMP9	1187	0.033
ENSP00000380349	CAPN3	798	0.033
ENSP00000381634	SLC38A1	1	0.033
ENSP00000397181	RGS4	2	0.033
ENSP00000283977	PGM3	190	0.034
ENSP00000322542	GTF2I	801	0.034

ENSP00000344742	STAMBP	795	0.034
ENSP00000352673	ELF3	189	0.034
ENSP00000001008	FKBP4	988	0.035
ENSP00000303147	MAT2A	1	0.035
ENSP00000419361	ADCY5	12	0.035
ENSP00000168216	HSD17B10	191	0.036
ENSP00000260950	MSTN	191	0.036
ENSP00000251849	RAF1	4700	0.037
ENSP00000318775	ANAPC4	191	0.037
ENSP00000348786	RAP1A	1159	0.037
ENSP00000354251	NCKAP1	1153	0.037
ENSP00000360645	MAN1B1	191	0.037
ENSP00000377958	CCT4	799	0.037
ENSP00000385057	APOBEC3G	191	0.037
ENSP00000414237	INTS2	191	0.037
ENSP00000225698	C1QBP	800	0.038
ENSP00000261531	SNW1	903	0.038
ENSP00000321826	STXBP5	190	0.038
ENSP00000339845	DROSHA	190	0.038
ENSP00000291582	AIRE	191	0.039
ENSP00000297338	RAD21	249	0.039
ENSP00000322229	FADS1	191	0.039

ENSP00000220584	FDFT1	1165	0.04
ENSP00000265080	RASGRF2	1	0.04
ENSP00000284154	GRAP	176	0.04
ENSP00000293441	SHANK1	189	0.04
ENSP00000225655	PFN1	792	0.041
ENSP00000281928	MED13L	191	0.041
ENSP00000301200	CDC42EP5	793	0.041
ENSP00000359245	ABCA4	1	0.041
ENSP00000374323	EPHA6	190	0.041
ENSP00000255688	RARRES3	172	0.042
ENSP00000269485	TNFRSF11A	382	0.042
ENSP00000310448	SART1	800	0.042
ENSP00000326830	CLK1	191	0.042
ENSP00000361548	MPL	191	0.042
ENSP00000371475	TP53BP1	191	0.042
ENSP00000257430	APC	983	0.043
ENSP00000261769	CDH1	2136	0.043
ENSP00000307939	GCC2	191	0.043
ENSP00000346148	PRKAA1	191	0.043
ENSP00000359910	PSMA7	190	0.043
ENSP00000372853	C2	4	0.043
ENSP00000378288	MYLK3	191	0.043

ENSP00000265529	KIF9	189	0.044
ENSP00000278198	LRRC4C	191	0.044
ENSP00000304102	COPS6	190	0.044
ENSP00000304188	OR8U1	9	0.044
ENSP00000357244	CCT3	191	0.044
ENSP00000184266	NDUFB4	2	0.045
ENSP00000230895	DAP	189	0.045
ENSP00000296785	ANKRA2	49	0.045
ENSP00000354003	GYPA	340	0.045
ENSP00000357311	CENPW	191	0.045
ENSP00000361473	KDM4A	191	0.045
ENSP00000362578	RNF8	191	0.045
ENSP00000366746	STAM	794	0.045
ENSP00000255380	CHRM3	2	0.046
ENSP00000256497	EDEM1	191	0.046
ENSP00000273398	ATP6V1A	176	0.046
ENSP00000328216	ORAI1	191	0.046
ENSP00000338207	LMO1	191	0.046
ENSP00000246194	RALY	184	0.047
ENSP00000297373	PHKG1	190	0.047
ENSP00000320567	MRPS33	191	0.047
ENSP00000345728	ATP7A	192	0.047

ENSP00000419923	KLF6	191	0.047
ENSP00000246041	AP5S1	191	0.048
ENSP00000380378	PAFAH1B1	199	0.048
ENSP00000382791	GRIK1	191	0.048
ENSP00000390849	ABHD5	792	0.048
ENSP00000417864	ANP32A	799	0.048
ENSP00000252699	ACTN4	1	0.049
ENSP00000258774	HUS1	191	0.049
ENSP00000261037	COL8A1	197	0.049
ENSP00000326261	SRRM1	191	0.049
ENSP00000347839	RAB11FIP2	382	0.049
ENSP00000349275	NRG1	404	0.049
ENSP00000417052	EBP	1	0.049
ENSP00000284995	TSEN2	191	0.05
ENSP00000331514	ACTG1	192	0.05
ENSP00000346294	S100A4	806	0.05
ENSP00000003100	CYP51A1	1	0.051
ENSP00000281950	GEMIN6	191	0.051
ENSP00000284690	DHX32	184	0.051
ENSP00000322142	ING5	382	0.051
ENSP00000360183	STX16	381	0.051
ENSP00000367959	HTR2A	3	0.051

ENSP00000263025	MAPK3	237	0.052
ENSP00000335632	CHP1	191	0.052
ENSP00000368678	AGRN	5	0.052
ENSP00000377840	CACNB1	1	0.052
ENSP00000248933	SEZ6L	1	0.053
ENSP00000339916	LIMK2	189	0.053
ENSP00000344871	MYO1F	189	0.053
ENSP00000351894	NCOA6	865	0.053
ENSP00000369962	IGSF5	2	0.053
ENSP00000283875	GTF2E1	791	0.054
ENSP00000291688	MCM3AP	793	0.054
ENSP00000317379	GLS	1	0.054
ENSP00000361824	SPTAN1	5	0.054
ENSP00000223095	SERPINE1	1759	0.055
ENSP00000331201	HGS	804	0.055
ENSP00000223642	C5	799	0.056
ENSP00000236671	CTSD	191	0.056
ENSP00000297261	SHH	1392	0.056
ENSP00000323880	FOXJ1	1	0.056
ENSP00000358497	RNGTT	191	0.057
ENSP00000360217	RHAG	340	0.057
ENSP00000362166	MEAF6	382	0.057

ENSP00000271411	POU2F1	191	0.058
ENSP00000318641	INTS3	799	0.058
ENSP00000343479	NBR1	789	0.058
ENSP00000347978	ERCC5	2	0.058
ENSP00000356602	VTA1	794	0.058
ENSP00000371512	SGCZ	794	0.058
ENSP00000233057	EIF2AK2	387	0.059
ENSP00000256379	MED6	203	0.059
ENSP00000259455	GABBR2	1	0.059
ENSP00000323858	DDX54	191	0.059
ENSP00000339428	SOCS2	10	0.059
ENSP00000219172	CENPT	191	0.06
ENSP00000263205	MED15	191	0.06
ENSP00000294053	CLPB	190	0.06
ENSP00000356623	CITED2	382	0.06
ENSP00000352522	ATP6V1H	191	0.061
ENSP00000365851	BMI1	1176	0.061
ENSP00000384015	SUN1	43	0.061
ENSP00000400806	APTX	382	0.062
ENSP00000337103	CHAT	382	0.063
ENSP00000219244	CCL17	190	0.064
ENSP00000324274	ZCCHC10	191	0.064

ENSP00000342011	XRCC4	573	0.064
ENSP00000261558	AP5M1	191	0.065
ENSP00000280193	VEGFC	191	0.065
ENSP00000304704	CLP1	191	0.065
ENSP00000350283	BRCA1	5979	0.065
ENSP00000362095	SRPX2	2	0.065
ENSP00000257963	ACVR1B	191	0.066
ENSP00000264079	MCOLN1	189	0.066
ENSP00000363019	UBE2D1	191	0.066
ENSP00000260130	SDCBP	789	0.067
ENSP00000262367	CREBBP	7365	0.067
ENSP00000300055	PLIN1	792	0.067
ENSP00000302564	BCL2L1	1239	0.067
ENSP00000343782	ADRB3	1	0.067
ENSP00000352708	TRAPPC2	191	0.067
ENSP00000377969	GTF2F1	6	0.067
ENSP00000246032	STK35	191	0.068
ENSP00000255465	CCNA1	191	0.068
ENSP00000269397	CBX4	1366	0.068
ENSP00000257497	ANXA1	191	0.069
ENSP00000295066	DPY30	191	0.069
ENSP00000332973	SMAD3	1822	0.069

ENSP00000355599	TSNAX	191	0.069
ENSP00000228958	EIF2B1	191	0.07
ENSP00000222005	CDC37	1582	0.071
ENSP00000261636	ARL1	191	0.071
ENSP00000296490	WDR82	191	0.071
ENSP00000331358	GAST	990	0.071
ENSP00000254719	RPA1	964	0.072
ENSP00000348965	DYNC1H1	194	0.072
ENSP00000357292	UBQLN4	800	0.072
ENSP00000375863	HNRNPUL1	191	0.072
ENSP00000222139	EPOR	191	0.073
ENSP00000257789	ORC3	191	0.073
ENSP00000312999	GNAI2	1158	0.073
ENSP00000369129	DSP	798	0.073
ENSP00000304915	IL13	191	0.074
ENSP00000265171	EGF	2176	0.075
ENSP00000354532	PNP	7	0.075
ENSP00000355361	CD47	340	0.075
ENSP00000298281	PCF11	191	0.076
ENSP00000225603	CBX1	191	0.077
ENSP00000244289	LIPE	801	0.077
ENSP00000249373	SMO	191	0.077

ENSP00000261167	WBP11	1	0.077
ENSP00000298852	PSMC3	191	0.078
ENSP00000317300	LPCAT4	1	0.078
ENSP00000377446	SUCLG1	382	0.078
ENSP00000381822	CDH23	2	0.078
ENSP00000257254	APLNR	19	0.079
ENSP00000339151	IKBKB	385	0.079
ENSP00000261267	LYZ	191	0.08
ENSP00000389184	MARK2	191	0.08
ENSP00000322016	PUF60	191	0.081
ENSP00000337722	ARL6	190	0.081
ENSP00000358867	GNAI3	12	0.081
ENSP00000256474	VHL	9464	0.083
ENSP00000263209	DGCR8	190	0.083
ENSP00000308236	COMMD1	191	0.083
ENSP00000327145	FLNC	191	0.083
ENSP00000238112	CPSF3	191	0.084
ENSP00000298532	SNAPC4	190	0.084
ENSP00000355302	KRTAP1-5	2	0.084
ENSP00000263980	SLC9A1	191	0.086
ENSP00000313829	KHDRBS1	6378	0.086
ENSP00000348107	C1D	191	0.086

ENSP00000360882	COL5A1	3	0.086
ENSP00000263710	CLASP1	4	0.087
ENSP00000320604	FAXDC2	186	0.087
ENSP00000245479	SOX9	803	0.088
ENSP00000273320	ZKSCAN7	2	0.089
ENSP00000344460	CBS	188	0.089
ENSP00000350198	SSTR2	189	0.089
ENSP00000254227	NR0B2	382	0.09
ENSP00000315167	ALOX12B	190	0.09
ENSP00000361423	ABL1	1855	0.09
ENSP00000295685	ARPC2	191	0.091
ENSP00000358857	EMD	191	0.091
ENSP00000234590	ENO1	191	0.092
ENSP00000306881	SEC23A	381	0.092
ENSP00000318374	LENG8	190	0.092
ENSP00000349052	OSCP1	125	0.092
ENSP00000356791	DPT	1	0.092
ENSP00000366843	ATXN2	191	0.092
ENSP00000265729	SRI	191	0.093
ENSP00000326819	FANCB	190	0.093
ENSP00000361759	BEX2	3	0.093
ENSP00000260867	TIMM23	301	0.094

ENSP00000300737	STIM1	191	0.094
ENSP00000261890	RAB11A	382	0.095
ENSP00000307078	KIF5B	191	0.095
ENSP00000349577	PRODH	1	0.095
ENSP00000416753	MUC15	19	0.095
ENSP00000243077	LRP1	230	0.096
ENSP00000352425	WASF1	51	0.096
ENSP00000359174	SLC35A3	189	0.096
ENSP00000367408	CASK	1470	0.096
ENSP00000370503	CCM2	191	0.096
ENSP00000252997	GATA5	190	0.097
ENSP00000380227	ITGA4	1102	0.097
ENSP00000263697	DNAJC8	190	0.098
ENSP00000272645	POLR2D	1	0.098
ENSP00000226574	NFKB1	382	0.099
ENSP00000247866	NDUFB2	191	0.099
ENSP00000261740	TRPV4	10	0.099
ENSP00000262968	TJP3	4	0.099
ENSP00000264039	GPC1	3	0.099
ENSP00000300574	CRK	1963	0.099
ENSP00000311005	MAPK7	1	0.1
ENSP00000355050	CTNNBL1	191	0.1

ENSP00000354791	DCTN1	988	0.101
ENSP00000359151	DBT	165	0.101
ENSP00000337053	SEL1L	191	0.102
ENSP00000386733	PLEKHG2	3	0.102
ENSP00000282344	USP12	191	0.103
ENSP00000313691	FTMT	1	0.103
ENSP00000196061	PLOD1	191	0.104
ENSP00000264839	RIMS1	383	0.105
ENSP00000310244	RASGRP1	55	0.105
ENSP00000354586	GLI2	798	0.105
ENSP00000356975	ADAMTS4	2	0.105
ENSP00000303686	NTSR2	1	0.106
ENSP00000376822	STEAP3	189	0.106
ENSP00000263073	SMG6	1	0.107
ENSP00000254480	SMARCC1	951	0.108
ENSP00000223208	CEP41	187	0.109
ENSP00000257899	BLOC1S1	570	0.109
ENSP00000303325	TACR3	3	0.109
ENSP00000320940	NCOA1	3030	0.109
ENSP00000322570	POLE	544	0.11
ENSP00000221573	SNAPC2	190	0.111
ENSP00000263281	GIPR	6	0.111

ENSP00000297564	COX6C	191	0.111
ENSP00000360035	PPP1R3D	5	0.111
ENSP00000361310	POLH	191	0.111
ENSP00000387662	GCG	2568	0.111
ENSP00000265641	CPT1A	191	0.112
ENSP00000363641	TXN	987	0.113
ENSP00000367766	RPGR	1	0.113
ENSP00000309629	CFL1	199	0.114
ENSP00000344741	INSIG1	191	0.114
ENSP00000413234	AP2A2	528	0.114
ENSP00000301838	FADD	986	0.116
ENSP00000296084	RYK	187	0.117
ENSP00000335333	PIP5K1C	190	0.117
ENSP00000234831	TMEM59	2	0.118
ENSP00000265351	XPO5	190	0.118
ENSP00000354119	LAT	176	0.118
ENSP00000278385	CD44	211	0.119
ENSP00000287766	SLC6A1	799	0.119
ENSP00000346566	CKAP5	191	0.119
ENSP00000266744	ASCL1	189	0.12
ENSP00000305529	SIRPG	310	0.12
ENSP00000247161	ELK1	47	0.121

ENSP00000263026	EEF2K	26	0.121
ENSP00000236147	SELL	191	0.122
ENSP00000242067	BBS9	572	0.123
ENSP00000298139	WRN	141	0.123
ENSP00000305464	APLN	4	0.123
ENSP00000315955	FOXA2	386	0.123
ENSP00000333633	MTA1	564	0.123
ENSP00000324740	YES1	185	0.124
ENSP00000367802	TAF1C	191	0.124
ENSP00000308021	CEP290	797	0.125
ENSP00000358242	PNISR	191	0.125
ENSP00000359518	MRGBP	191	0.125
ENSP00000312262	ADRBK1	716	0.126
ENSP00000265459	NRXN2	163	0.127
ENSP00000370719	ITSN1	191	0.127
ENSP00000164133	PPP2R5B	189	0.128
ENSP00000329380	GP1BA	2726	0.128
ENSP00000272298	CALM2	4	0.129
ENSP00000276689	NDUFB9	195	0.129
ENSP00000290974	ZFYVE28	191	0.129
ENSP00000298130	SPTSSA	190	0.129
ENSP00000370938	CDK8	553	0.129

ENSP00000417132	BAP1	382	0.129
ENSP00000250617	ARHGEF6	190	0.13
ENSP00000262958	GNA15	170	0.132
ENSP00000305892	TMEM208	188	0.132
ENSP00000363435	ITPR3	52	0.132
ENSP00000366819	UCHL3	190	0.132
ENSP00000370748	IDI1	1	0.132
ENSP00000371169	RCL1	1	0.132
ENSP00000301457	NDUFA7	2	0.133
ENSP00000309591	PRKACA	6	0.133
ENSP00000254584	ARFIP2	191	0.134
ENSP00000322170	PFN4	1	0.134
ENSP00000171214	RDH8	1	0.135
ENSP00000254950	VPS4A	794	0.135
ENSP00000239940	PFN2	1	0.136
ENSP00000276420	DOK2	160	0.136
ENSP00000296412	ADH5	2	0.136
ENSP00000313922	OAZ3	190	0.136
ENSP00000405890	PBX1	191	0.136
ENSP00000220751	RIPK2	191	0.137
ENSP00000261813	PFDN1	191	0.137
ENSP00000357753	IVL	191	0.137

ENSP00000043402	RTN4R	191	0.139
ENSP00000327541	BLZF1	169	0.139
ENSP00000353099	HLA-DRB1	800	0.139
ENSP00000388241	KIF26A	189	0.139
ENSP00000259631	CCL27	1	0.14
ENSP00000353701	DPP3	190	0.14
ENSP00000204604	CHRD	191	0.141
ENSP00000362795	CXCR3	186	0.142
ENSP00000394794	PTPN13	19	0.142
ENSP00000356541	SF3B5	190	0.143
ENSP00000359206	BTRC	235	0.143
ENSP00000378338	GIT1	703	0.143
ENSP00000265354	SRF	1179	0.144
ENSP00000316578	SUZ12	191	0.144
ENSP00000216832	PNN	382	0.145
ENSP00000351446	WDR5	1427	0.145
ENSP00000175506	ASNS	378	0.146
ENSP00000256759	FST	191	0.146
ENSP00000261708	UTP6	1	0.146
ENSP00000307491	WDR48	191	0.146
ENSP00000313921	MSRA	1	0.146
ENSP00000355536	MTR	193	0.146

ENSP00000396620	NFYC	189	0.146
ENSP00000229854	MCM3	806	0.147
ENSP00000319664	NUDC	3	0.147
ENSP00000354497	FAN1	188	0.147
ENSP00000266970	CDK2	5360	0.148
ENSP00000317468	CHMP6	1	0.148
ENSP00000251102	CNGB1	191	0.149
ENSP00000317714	STX4	1489	0.149
ENSP00000333194	RGS19	97	0.15
ENSP00000361311	TMEM53	1	0.15
ENSP00000249344	STRIP2	2	0.151
ENSP00000340330	KAT5	3033	0.151
ENSP00000360672	PARD6B	190	0.151
ENSP00000222725	LFNG	191	0.152
ENSP00000227524	PRPF19	830	0.153
ENSP00000232458	ECT2	9	0.153
ENSP00000265517	MTTP	10	0.154
ENSP00000368464	KIF24	1	0.154
ENSP00000293288	BAX	716	0.155
ENSP00000320401	UGT2B17	2	0.155
ENSP00000347197	C5AR1	3	0.156
ENSP00000365775	MTHFR	193	0.157

ENSP00000396439	RING1	190	0.157
ENSP00000254998	NXT1	382	0.158
ENSP00000259089	BLK	189	0.158
ENSP00000394400	SKIV2L	1	0.158
ENSP00000236192	VAMP4	191	0.159
ENSP00000260356	THBS1	60	0.159
ENSP00000262305	RAB11FIP3	382	0.159
ENSP00000264126	GPSM2	1	0.16
ENSP00000229135	IFNG	382	0.161
ENSP00000330237	CASP9	714	0.162
ENSP00000354927	MAP3K3	191	0.162
ENSP00000364519	TFDP1	191	0.162
ENSP00000248996	GNAZ	179	0.163
ENSP00000262519	SETD1A	191	0.163
ENSP00000281821	EPHA4	9	0.163
ENSP00000304236	CD14	2	0.163
ENSP00000361202	IRS4	186	0.164
ENSP00000407964	NEDD1	182	0.164
ENSP00000362014	DNM1	573	0.165
ENSP00000338862	BRIX1	191	0.166
ENSP00000340766	SMG7	191	0.166
ENSP00000384515	PARVB	190	0.166

ENSP00000419692	RXRA	1152	0.166
ENSP00000363079	MBL2	191	0.167
ENSP00000223114	MOGAT3	2	0.168
ENSP00000324422	ZYX	2	0.17
ENSP00000245157	BBS2	572	0.171
ENSP00000263923	KDR	234	0.171
ENSP00000316029	TLN1	189	0.171
ENSP00000301012	MVD	1	0.172
ENSP00000345708	KCNJ11	2	0.172
ENSP00000288602	BRAF	191	0.174
ENSP00000311113	JUP	821	0.174
ENSP00000354720	SMC3	1042	0.174
ENSP00000360268	ALDH18A1	190	0.174
ENSP00000364839	ASXL1	16	0.176
ENSP00000316338	BAIAP2	49	0.177
ENSP00000357748	BCCIP	184	0.177
ENSP00000327070	MDH2	795	0.178
ENSP00000170630	IL4R	513	0.179
ENSP00000265371	NRP1	799	0.179
ENSP00000172229	NGFR	1097	0.181
ENSP00000257904	CDK4	1334	0.181
ENSP00000342952	ADCY2	157	0.181

ENSP00000265734	CDK6	189	0.182
ENSP00000310771	GRB7	117	0.182
ENSP00000337088	MEN1	2147	0.182
ENSP00000216540	SLC10A1	190	0.183
ENSP00000304592	FASN	1758	0.183
ENSP00000302707	FPR1	186	0.184
ENSP00000156109	GPKOW	189	0.185
ENSP00000262133	RBL2	3	0.185
ENSP00000084795	RPL18	190	0.186
ENSP00000258301	STX6	763	0.187
ENSP00000260810	TOPBP1	317	0.188
ENSP00000264554	SHC2	512	0.188
ENSP00000313199	HNRNPD	571	0.188
ENSP00000351665	CLIP1	983	0.188
ENSP00000352257	XRCC6	382	0.188
ENSP00000369855	ASB9	191	0.188
ENSP00000204961	EFNB1	93	0.189
ENSP00000323967	SMARCE1	1	0.19
ENSP00000326550	TACC3	206	0.19
ENSP00000269571	ERBB2	2038	0.191
ENSP00000314813	OAZ1	570	0.191
ENSP00000246071	SNRPB2	188	0.193

ENSP00000300935	RAB8A	382	0.193
ENSP00000309503	YWHAZ	2812	0.193
ENSP00000368350	TPT1	563	0.194
ENSP00000356713	IFNGR1	382	0.195
ENSP00000252818	JUND	2147	0.196
ENSP00000366395	SYVN1	191	0.196
ENSP00000261837	GNB5	175	0.197
ENSP00000303864	OR8I2	2	0.197
ENSP00000362082	CCND3	191	0.197
ENSP00000391490	AGR2	1	0.198
ENSP00000417257	FNDC3A	1	0.198
ENSP00000361892	STK4	191	0.2
ENSP00000272521	TMEM177	2	0.201
ENSP00000296140	CCR1	191	0.201
ENSP00000308541	F2	1934	0.201
ENSP00000302021	MUC7	1	0.202
ENSP00000407401	PEX5	191	0.202
ENSP00000314214	VAMP2	1482	0.204
ENSP00000361151	CEL	191	0.205
ENSP00000366976	KRTAP1-4	190	0.205
ENSP00000264409	AGPAT9	1	0.206
ENSP00000291700	S100B	960	0.206

ENSP00000323050	RBBP8	435	0.206
ENSP00000364893	ARHGEF7	475	0.206
ENSP00000240055	NFYB	189	0.207
ENSP00000268261	PMM2	1	0.207
ENSP00000295598	ATP1A1	377	0.207
ENSP00000301071	TUBA1A	191	0.207
ENSP00000355325	PSMB5	920	0.208
ENSP00000252999	LAMA5	2	0.21
ENSP00000336740	LIMK1	371	0.21
ENSP00000381064	INTS10	746	0.21
ENSP00000307188	ASL	5	0.211
ENSP00000368914	PSTPIP1	191	0.211
ENSP00000227507	CCND1	8745	0.212
ENSP00000312697	DMAP1	382	0.212
ENSP00000226218	SEBOX	149	0.213
ENSP00000263464	BIRC3	190	0.213
ENSP00000297579	DCAF13	1	0.214
ENSP00000331817	ALYREF	191	0.214
ENSP00000381293	NSF	128	0.214
ENSP00000239451	SLC25A2	3	0.215
ENSP00000301843	CTTN	190	0.215
ENSP00000338868	PHF8	191	0.215

ENSP00000347733	TRRAP	589	0.216
ENSP00000222256	RAB3A	1179	0.217
ENSP00000052754	DCN	1348	0.218
ENSP00000258962	SRSF1	466	0.218
ENSP00000356256	TIMM17A	301	0.218
ENSP00000234160	GORASP2	169	0.219
ENSP00000282588	ITGA1	7	0.219
ENSP00000317992	NOC2L	190	0.219
ENSP00000364649	SDHB	389	0.219
ENSP00000369889	COL2A1	192	0.219
ENSP00000232461	GNAT1	382	0.22
ENSP00000256383	EIF2S1	757	0.22
ENSP00000363763	EPHB2	95	0.22
ENSP00000304283	RAC3	186	0.222
ENSP00000358617	PHTF1	2	0.222
ENSP00000255764	MED10	191	0.223
ENSP00000306330	YWHAG	35	0.223
ENSP00000035307	CHPF2	189	0.224
ENSP00000338983	MUC1	2169	0.224
ENSP00000262304	PKD1	180	0.225
ENSP00000296223	POLR2H	125	0.225
ENSP00000344352	ATF3	1436	0.225

ENSP00000351926	AP2A1	382	0.225
ENSP00000354623	DFNB31	190	0.226
ENSP00000378130	CFI	2	0.226
ENSP00000221930	TGFB1	2139	0.227
ENSP00000305480	FEN1	191	0.227
ENSP00000348827	THRB	8	0.227
ENSP00000231790	MLH1	373	0.228
ENSP00000324173	HSPA5	191	0.228
ENSP00000346389	MEF2A	188	0.228
ENSP00000363092	PRKG1	388	0.228
ENSP00000408910	DCTN2	2	0.228
ENSP00000252486	APOE	94	0.229
ENSP00000334061	HDAC6	191	0.229
ENSP00000219255	PARD6A	883	0.23
ENSP00000314897	ANGPT2	31	0.231
ENSP00000374455	SQSTM1	1514	0.231
ENSP00000405965	SUMO2	191	0.231
ENSP00000262854	HUWE1	184	0.232
ENSP00000303019	GPHN	573	0.232
ENSP00000224337	BLNK	395	0.233
ENSP00000257818	LMO2	193	0.233
ENSP00000401508	NAPRT1	185	0.233

ENSP00000360154	OCRL	191	0.236
ENSP00000173229	NTN1	617	0.237
ENSP00000293330	HCRT	191	0.237
ENSP00000263918	STRN	24	0.238
ENSP00000356671	SERPINC1	798	0.238
ENSP00000368190	NPHS1	12	0.238
ENSP00000338018	HIF1A	12071	0.239
ENSP00000357283	LMNA	191	0.24
ENSP00000382004	CTNND1	788	0.24
ENSP00000316042	HNRNPA0	188	0.241
ENSP00000332643	NDN	129	0.241
ENSP00000264663	NNT	1	0.242
ENSP00000313681	SPHK1	382	0.242
ENSP00000359663	CD40LG	191	0.243
ENSP00000265023	KNG1	1658	0.244
ENSP00000331746	CALCA	259	0.245
ENSP00000227752	IL10RA	191	0.246
ENSP00000311360	RAD9A	191	0.246
ENSP00000290039	CACHD1	1	0.247
ENSP00000311449	RAB6A	191	0.247
ENSP00000361878	CAP1	3	0.247
ENSP00000388107	UBA52	191	0.247

ENSP00000290541	PSMB4	758	0.248
ENSP00000296755	MAP1B	45	0.248
ENSP00000343054	RBM5	813	0.248
ENSP00000005226	USH1C	9	0.249
ENSP00000234296	ORC2	14	0.249
ENSP00000375777	STRN4	760	0.249
ENSP00000299402	APBB1	1550	0.25
ENSP00000324944	MBOAT1	1	0.25
ENSP00000267973	WDR61	1	0.252
ENSP00000357708	S100A6	1	0.252
ENSP00000334188	PFDN5	568	0.253
ENSP00000336790	ATF4	1245	0.253
ENSP00000411672	ATP6V0E2	20	0.254
ENSP00000290663	MED8	191	0.255
ENSP00000413625	FNBP1	181	0.255
ENSP00000262134	LPCAT2	188	0.256
ENSP00000309845	HRAS	2119	0.257
ENSP00000252487	TOMM40	272	0.258
ENSP00000361219	GTF3C4	191	0.258
ENSP00000240316	COIL	3	0.26
ENSP00000317337	CD300LB	186	0.26
ENSP00000350720	SMARCA4	1507	0.26

ENSP00000368119	GALT	191	0.26
ENSP00000414982	KLC1	191	0.26
ENSP00000044462	PSMA4	739	0.262
ENSP00000356520	DHX9	17	0.262
ENSP00000261195	GYS2	4	0.264
ENSP00000313420	PRKDC	1705	0.264
ENSP00000318318	MPI	1	0.264
ENSP00000320949	CNOT1	71	0.264
ENSP00000216341	GZMB	191	0.265
ENSP00000321345	IL23R	19	0.265
ENSP00000253792	ACLY	774	0.266
ENSP00000366565	VPS28	382	0.266
ENSP00000206542	OSGEP	188	0.267
ENSP00000245451	BMP4	574	0.267
ENSP00000263168	CAPZA1	191	0.267
ENSP00000372326	FECH	5	0.267
ENSP00000263036	OPTN	340	0.268
ENSP00000311344	PPP2R1B	71	0.268
ENSP00000302665	IGF1	3019	0.269
ENSP00000340409	SMPD1	191	0.269
ENSP00000355747	PSEN2	190	0.269
ENSP00000345530	NEDD4	190	0.27

ENSP00000298854	RAPSN	1	0.271
ENSP00000326366	PSEN1	1646	0.272
ENSP00000349465	PICK1	191	0.273
ENSP00000259512	DERL1	191	0.275
ENSP00000276062	NDUFB11	7	0.275
ENSP00000318472	NCAM1	762	0.275
ENSP00000265333	VDAC1	378	0.276
ENSP00000321326	F2R	149	0.276
ENSP00000401445	ERN1	186	0.276
ENSP00000006275	TRAPPC6A	191	0.277
ENSP00000295797	PRKCI	575	0.277
ENSP00000318585	BACE1	191	0.278
ENSP00000365891	WAS	1147	0.278
ENSP00000262584	RPL8	165	0.279
ENSP00000265094	FBXW11	28	0.279
ENSP00000396308	DHFR	191	0.279
ENSP00000343392	XRCC3	3	0.281
ENSP00000364336	TBXA2R	74	0.281
ENSP00000339933	PKLR	2	0.282
ENSP00000278616	ATM	1956	0.283
ENSP00000254322	DNAJB1	186	0.285
ENSP00000351049	PAK4	362	0.285

ENSP00000313007	PABPC1	382	0.287
ENSP00000334100	EXOC7	197	0.287
ENSP00000373614	SELPLG	191	0.287
ENSP00000354476	SREBF2	1165	0.289
ENSP00000257192	DSG1	1	0.29
ENSP00000262554	SPTLC1	190	0.292
ENSP00000262160	SMAD2	2229	0.293
ENSP00000285379	CA2	32	0.293
ENSP00000336701	RAD51C	187	0.293
ENSP00000349547	RASSF1	166	0.293
ENSP00000233146	MSH2	564	0.298
ENSP00000231449	IL4	169	0.299
ENSP00000362900	SRSF4	93	0.301
ENSP00000359300	CETN2	794	0.302
ENSP00000363868	ABCA1	389	0.303
ENSP00000296218	DNALI1	1	0.304
ENSP00000360532	CDC5L	4	0.304
ENSP00000404121	ILF3	792	0.304
ENSP00000259808	RIPK1	1162	0.305
ENSP00000347719	TBCD	1	0.305
ENSP00000368686	E2F4	16	0.305
ENSP00000370557	MIS12	11	0.305

ENSP00000246891	CSN1S1	191	0.306
ENSP00000306512	IL8	1573	0.306
ENSP00000329357	SP1	2006	0.306
ENSP00000216223	IL2RB	610	0.307
ENSP00000263817	ABCB11	190	0.307
ENSP00000261205	SYT1	1168	0.308
ENSP00000386331	MYO7A	7	0.309
ENSP00000347858	XIAP	710	0.311
ENSP00000333982	NDEL1	372	0.312
ENSP00000269300	PIK3R5	366	0.313
ENSP00000310040	EIF3F	170	0.313
ENSP00000345731	DLG1	381	0.313
ENSP00000264972	ZAP70	180	0.314
ENSP00000270538	TIMM44	301	0.314
ENSP00000326630	ZFPM1	1	0.315
ENSP00000340691	EIF4EBP1	801	0.315
ENSP00000310551	LCLAT1	1	0.317
ENSP00000263354	NAPA	128	0.318
ENSP00000351777	VCP	1179	0.318
ENSP00000334122	FGF3	191	0.319
ENSP00000287647	FANCD2	570	0.323
ENSP00000316377	DLGAP1	379	0.324

ENSP00000216797	NFKBIA	309	0.325
ENSP00000293970	TBC1D24	187	0.325
ENSP00000312122	SEC13	381	0.325
ENSP00000249647	SNAP23	763	0.327
ENSP00000313752	SSNA1	1	0.329
ENSP00000315859	RNPS1	592	0.33
ENSP00000285021	XPC	794	0.331
ENSP00000307235	EIF2AK3	679	0.333
ENSP00000360569	SCP2	2	0.334
ENSP00000342755	RNF41	190	0.338
ENSP00000262013	SPAG9	191	0.34
ENSP00000280704	LDHC	1	0.34
ENSP00000316032	NUP98	381	0.34
ENSP00000230340	BYSL	1	0.341
ENSP00000320171	PKM	193	0.341
ENSP00000354782	CD247	185	0.341
ENSP00000383059	ARGLU1	66	0.341
ENSP00000007708	PDK2	199	0.342
ENSP00000219476	TSC2	1673	0.342
ENSP00000260762	EXOC6	38	0.342
ENSP00000315791	CSTF3	573	0.342
ENSP00000221265	PAF1	190	0.343

ENSP00000301972	MYRIP	6	0.343
ENSP00000329623	BCL2	1336	0.345
ENSP00000307387	PDCD6IP	1361	0.346
ENSP00000358105	APH1A	190	0.346
ENSP00000264705	CAD	14	0.348
ENSP00000334448	GNG2	373	0.348
ENSP00000347942	RET	381	0.349
ENSP00000229769	FANCE	310	0.352
ENSP00000244741	CDKN1A	597	0.354
ENSP00000352516	DNMT1	394	0.354
ENSP00000358554	BCAS2	84	0.355
ENSP00000217109	CSTF1	573	0.356
ENSP00000256996	DDB2	47	0.358
ENSP00000324806	GSK3B	2475	0.358
ENSP00000220764	DECR1	187	0.359
ENSP00000245932	VASP	192	0.359
ENSP00000256857	GRP	717	0.359
ENSP00000303191	PLRG1	4	0.359
ENSP00000351273	CASP8	193	0.362
ENSP00000231454	IL5	1	0.363
ENSP00000293272	CCL5	346	0.363
ENSP00000361186	TP53RK	188	0.365

ENSP00000264033	CBL	23978	0.366
ENSP00000302177	MBOAT2	1	0.367
ENSP00000260947	BARD1	764	0.368
ENSP00000225983	HDAC5	37	0.37
ENSP00000261819	ANAPC5	4	0.37
ENSP00000308938	PLG	2356	0.37
ENSP00000316176	UBE2N	191	0.371
ENSP00000309166	RBM4	16	0.373
ENSP00000377262	SRPK2	66	0.373
ENSP00000350003	CCR3	153	0.377
ENSP00000299543	CTDP1	673	0.378
ENSP00000303507	BCR	15	0.378
ENSP00000304350	PRPF8	86	0.378
ENSP00000355518	FH	11	0.379
ENSP00000256958	SLCO1B1	190	0.38
ENSP00000327801	P4HB	186	0.38
ENSP00000265038	ERCC8	32	0.381
ENSP00000401980	MAVS	985	0.381
ENSP00000361359	CD40	191	0.382
ENSP00000387699	CREB1	191	0.382
ENSP00000394624	OPRM1	175	0.382
ENSP00000262193	PSMB1	3	0.388

ENSP00000301788	POLR2G	15	0.388
ENSP00000354901	CXCL9	186	0.388
ENSP00000352121	PIK3CG	366	0.389
ENSP00000353154	NFASC	9	0.389
ENSP00000346236	DDX46	66	0.394
ENSP00000264220	PPAT	10	0.397
ENSP00000367910	FANCG	262	0.397
ENSP00000281453	MLF1IP	166	0.398
ENSP00000391901	PHF1	210	0.399
ENSP00000261366	LMNB1	315	0.4
ENSP00000405934	ITPR1	388	0.401
ENSP00000267169	DIABLO	1	0.407
ENSP00000324729	SAV1	25	0.407
ENSP00000389934	EXOC5	38	0.407
ENSP00000382177	MYO5A	6	0.408
ENSP00000267101	ERBB3	594	0.409
ENSP00000264414	CUL3	410	0.41
ENSP00000264335	YWHAE	3	0.411
ENSP00000339353	CPSF1	191	0.411
ENSP00000353452	MYLK	992	0.411
ENSP00000367934	UQCRQ	1	0.411
ENSP00000351908	MAP3K5	988	0.412

ENSP00000363822	AR	1205	0.413
ENSP00000264708	POMC	973	0.414
ENSP00000297151	SLU7	583	0.415
ENSP00000221972	CD79A	190	0.417
ENSP00000257181	PRPF38A	270	0.417
ENSP00000299421	ILK	477	0.417
ENSP00000312652	LEP	2708	0.419
ENSP00000341538	SEC61G	382	0.421
ENSP00000350275	HIST1H3A	513	0.422
ENSP00000370151	RAD17	51	0.423
ENSP00000360515	SUPT3H	181	0.425
ENSP00000381098	GRIP1	190	0.425
ENSP00000341838	TNNI3	17	0.426
ENSP00000351896	TRAPPC4	191	0.426
ENSP00000309103	BAD	207	0.427
ENSP00000367830	PRKCZ	190	0.427
ENSP00000216254	ACO2	5	0.429
ENSP00000278568	PAK1	551	0.429
ENSP00000360798	EPS15	910	0.429
ENSP00000306497	KCNJ4	2	0.43
ENSP00000406037	KAT8	190	0.433
ENSP00000267430	FANCM	155	0.434

ENSP00000271620	PRUNE	1	0.434
ENSP00000288135	KIT	190	0.437
ENSP00000310170	FOSL1	190	0.437
ENSP00000315702	MOB4	548	0.439
ENSP00000251968	TSG101	1746	0.44
ENSP00000287727	ZFYVE9	914	0.441
ENSP00000302620	AGXT	190	0.442
ENSP00000302961	HSPA4	406	0.442
ENSP00000253024	TRIM28	191	0.443
ENSP00000366410	NMNAT1	185	0.443
ENSP00000221233	EXOSC5	189	0.444
ENSP00000363216	OGDHL	1	0.444
ENSP00000302967	HDAC3	648	0.446
ENSP00000276414	GNRH1	187	0.447
ENSP00000343204	JAK1	1041	0.449
ENSP00000216392	PYGL	3	0.451
ENSP00000358918	SUFU	12	0.451
ENSP00000179259	C12orf5	187	0.452
ENSP00000343274	INTS8	391	0.453
ENSP00000367797	SKI	4	0.454
ENSP00000354673	CNOT4	71	0.456
ENSP00000286827	TIAM1	1	0.457

ENSP00000284818	LY96	1	0.458
ENSP00000372793	LTA	137	0.463
ENSP00000261799	PDGFRB	374	0.467
ENSP00000384273	RELA	2007	0.471
ENSP00000229022	VDR	917	0.473
ENSP00000260010	TLR2	2	0.473
ENSP00000263331	POLR1B	189	0.473
ENSP00000334564	POLR3C	191	0.476
ENSP00000339328	PLAUR	191	0.477
ENSP00000360316	DHCR24	1	0.477
ENSP00000250495	NEDD8	390	0.479
ENSP00000263694	SNRNP40	3	0.481
ENSP00000379110	CXCL1	189	0.482
ENSP00000226279	CD38	2	0.483
ENSP00000228872	CDKN1B	1339	0.483
ENSP00000356918	STX7	319	0.487
ENSP00000309555	HCFC1	367	0.488
ENSP00000317159	CYC1	573	0.49
ENSP00000335074	GHRL	188	0.49
ENSP00000360286	RAE1	381	0.49
ENSP00000350708	RAD23B	747	0.492
ENSP00000228682	GLI1	14	0.493

ENSP00000332468	TRAF3	963	0.493
ENSP00000342056	CS	9	0.493
ENSP00000356087	IKBKE	159	0.494
ENSP00000005257	RALA	228	0.495
ENSP00000270202	AKT1	12815	0.495
ENSP00000263967	PIK3CA	412	0.496
ENSP00000370473	IGFBP3	2256	0.496
ENSP00000362441	ATRX	190	0.5
ENSP00000263864	VAMP8	319	0.502
ENSP00000216225	RBX1	1498	0.503
ENSP00000261023	ITGAV	184	0.503
ENSP00000222673	OGDH	4	0.507
ENSP00000300161	YWHAB	248	0.508
ENSP00000397552	ACTL6A	180	0.508
ENSP00000302150	PRL	623	0.509
ENSP00000216442	ATP6V1D	176	0.514
ENSP00000265056	MCM2	15	0.514
ENSP00000358997	IRAK1	1179	0.515
ENSP00000369497	BRCA2	438	0.515
ENSP00000290649	AMFR	190	0.518
ENSP00000361021	PTEN	198	0.519
ENSP00000046794	LCP2	365	0.52

ENSP00000246747	ARL2	1	0.521
ENSP00000264515	RBBP5	12	0.521
ENSP00000253004	ASS1	12	0.524
ENSP00000262633	RBM42	230	0.525
ENSP00000335657	CCK	10	0.526
ENSP00000366006	UBIAD1	1	0.526
ENSP00000359211	DPYD	1	0.527
ENSP00000310127	IRF3	264	0.531
ENSP00000381717	UBE2D2	71	0.531
ENSP00000250448	FOXA1	19	0.534
ENSP00000318297	RUVBL1	380	0.535
ENSP00000360683	PTPN1	315	0.536
ENSP00000262735	PPARA	409	0.539
ENSP00000203407	UQCRC1	1	0.54
ENSP00000400717	GNA13	74	0.542
ENSP00000417764	ALG2	191	0.542
ENSP00000418447	PPP2CA	2403	0.542
ENSP00000262965	TCF3	381	0.543
ENSP00000212015	SIRT1	371	0.548
ENSP00000280346	DLAT	2	0.55
ENSP00000332258	DGAT1	2	0.551
ENSP00000347379	OCLN	4	0.551

ENSP00000345571	E2F1	725	0.552
ENSP00000300738	RRM1	7	0.553
ENSP00000256010	NTS	1	0.554
ENSP00000335544	CCKBR	9	0.555
ENSP00000307863	U2AF2	3671	0.556
ENSP00000366013	GNB2L1	274	0.557
ENSP00000390500	STK3	25	0.558
ENSP00000260970	PPIG	270	0.559
ENSP00000262238	YY1	505	0.559
ENSP00000341344	GGA1	395	0.559
ENSP00000226730	IL2	191	0.561
ENSP00000295897	ALB	3159	0.563
ENSP00000230354	TBP	1733	0.564
ENSP00000350512	COPS5	1035	0.566
ENSP00000344936	PTTG1	187	0.567
ENSP00000352980	HIST1H4A	658	0.567
ENSP00000359727	BAG2	580	0.568
ENSP00000261479	PSMA6	53	0.569
ENSP00000262105	MCM4	113	0.57
ENSP00000245206	GOT2	3	0.572
ENSP00000337040	UNC119	1	0.572
ENSP00000361066	NCOA3	93	0.572

ENSP00000259469	RPL35	382	0.576
ENSP00000356480	RNF2	1	0.578
ENSP00000270142	SOD1	190	0.581
ENSP00000371432	PRLR	617	0.581
ENSP00000396127	RAN	2220	0.581
ENSP00000265773	SMARCA2	8	0.582
ENSP00000383199	NEDD4L	190	0.583
ENSP00000252945	CYP2E1	191	0.585
ENSP00000010338	TRAF3IP3	7	0.586
ENSP00000343745	DICER1	71	0.586
ENSP00000216194	ADSL	13	0.587
ENSP00000303088	POLR3D	382	0.588
ENSP00000317904	GYS1	560	0.589
ENSP00000356248	PTPN7	182	0.592
ENSP00000264657	STAT3	6716	0.594
ENSP00000305790	SF3B3	3	0.595
ENSP00000223129	RPA3	15	0.596
ENSP00000356425	UCHL5	191	0.597
ENSP00000264832	ICAM1	5	0.598
ENSP00000299022	LIPC	2	0.599
ENSP00000314949	POLR2A	1540	0.6
ENSP00000355493	ADSS	5	0.602

ENSP00000368880	FOXO1	36	0.603
ENSP00000348577	RANGAP1	2217	0.606
ENSP00000320180	GHRHR	1	0.607
ENSP00000219240	DHODH	1	0.608
ENSP00000289779	F11R	35	0.608
ENSP00000228916	SCNN1A	190	0.609
ENSP00000237527	GHRH	1	0.612
ENSP00000261461	PPP2R5A	206	0.613
ENSP00000360125	PGM1	1	0.615
ENSP00000378529	FZR1	122	0.616
ENSP00000295767	CHCHD4	272	0.62
ENSP00000228140	RPS13	4	0.621
ENSP00000220592	AGO2	71	0.625
ENSP00000257770	NT5E	1	0.625
ENSP00000358541	SIKE1	158	0.625
ENSP00000287497	ITGAM	191	0.626
ENSP00000273047	RAB5A	191	0.627
ENSP00000371973	SAP18	6	0.632
ENSP00000231509	NR3C1	26	0.634
ENSP00000306866	GABARAP	4	0.635
ENSP00000359939	EXOSC1	1	0.635
ENSP00000365439	HNRNPK	4913	0.636

ENSP00000264606	HDAC4	14	0.643
ENSP00000284384	PRKCA	379	0.644
ENSP00000262435	SMURF2	11	0.645
ENSP00000411698	USO1	191	0.649
ENSP00000232607	UMPS	1	0.65
ENSP00000354876	MT-CO2	191	0.65
ENSP00000297185	HSPA9	301	0.652
ENSP00000230449	EXOC2	32	0.655
ENSP00000355153	CDKN2A	199	0.659
ENSP00000367276	CKAP2	190	0.663
ENSP00000266000	DAXX	211	0.668
ENSP00000382595	PAICS	9	0.668
ENSP00000367872	GNB1	4	0.67
ENSP00000211998	VCL	19	0.671
ENSP00000339393	CCR6	1	0.675
ENSP00000215587	POLR2E	578	0.676
ENSP00000314004	ANAPC2	39	0.677
ENSP00000334458	GATA4	1	0.677
ENSP00000221494	SF3A2	3415	0.682
ENSP00000357879	PSMD4	747	0.682
ENSP00000276682	EIF3H	21	0.683
ENSP00000413493	CPSF3L	7	0.684

ENSP00000337761	RAB27A	9	0.686
ENSP00000385269	ELAVL1	41	0.686
ENSP00000342793	PLD1	193	0.692
ENSP00000217244	CSNK2A1	191	0.693
ENSP00000271628	SF3B4	23	0.695
ENSP00000349437	IGF2R	103	0.695
ENSP00000380942	ARHGEF12	74	0.695
ENSP00000251810	RRM2B	7	0.699
ENSP00000248566	SHFM1	3814	0.7
ENSP00000291552	U2AF1	264	0.7
ENSP00000294172	NXF1	817	0.701
ENSP00000396219	MEF2C	11	0.702
ENSP00000259075	TANK	2	0.703
ENSP00000318861	SF3B2	60	0.704
ENSP00000284811	TCEB1	283	0.706
ENSP00000358716	DDX20	71	0.706
ENSP00000248114	GFER	272	0.709
ENSP00000284957	RABGEF1	665	0.71
ENSP00000263033	SYTL4	6	0.711
ENSP00000269321	ARHGDIA	425	0.711
ENSP00000225577	RPS6KB1	34	0.715
ENSP00000294117	GNG3	2	0.717

ENSP00000308533	GEMIN2	71	0.718
ENSP00000320866	CALR	368	0.719
ENSP00000339992	MYB	191	0.72
ENSP00000285814	MKI67IP	6	0.726
ENSP00000348554	CDC16	83	0.726
ENSP00000355261	SMG5	3	0.726
ENSP00000324804	PPP2R1A	98	0.727
ENSP00000276571	CRH	4	0.73
ENSP00000352400	NUP214	797	0.734
ENSP00000371308	CENPJ	1	0.735
ENSP00000245960	CDC25B	219	0.736
ENSP00000352929	CSNK1E	1	0.736
ENSP00000335304	DLST	3	0.737
ENSP00000416097	GOLGA2	360	0.738
ENSP00000351997	MAP2K6	224	0.74
ENSP00000247668	TRAF2	2204	0.741
ENSP00000354554	MT-CYB	763	0.741
ENSP00000205402	DLD	179	0.742
ENSP00000300289	PDIA3	353	0.742
ENSP00000368699	ISG15	191	0.745
ENSP00000339109	ANAPC1	1051	0.748
ENSP00000220849	EIF3E	16	0.751

ENSP00000264818	TYK2	33	0.753
ENSP00000348877	GPI	191	0.756
ENSP00000354961	MT-ND4	762	0.757
ENSP00000338297	IGF2	103	0.758
ENSP00000361418	IPO13	238	0.758
ENSP00000348461	RAC1	3123	0.762
ENSP00000219252	POLR2C	188	0.767
ENSP00000263754	KAT2B	170	0.769
ENSP00000292303	CCR5	343	0.77
ENSP00000349049	KDM1A	1	0.771
ENSP00000419851	GMPS	189	0.773
ENSP00000298316	ARF6	768	0.785
ENSP00000162749	TNFRSF1A	513	0.786
ENSP00000379933	TPI1	187	0.786
ENSP00000263253	EP300	6151	0.787
ENSP00000311032	CASP3	193	0.787
ENSP00000316879	EIF4G1	778	0.787
ENSP00000262477	RABEP1	487	0.791
ENSP00000244769	ATXN1	5	0.792
ENSP00000358335	MAP3K7	224	0.793
ENSP00000362361	CDK9	382	0.794
ENSP00000348442	PSMD12	16	0.795

ENSP00000326804	CUL1	176	0.798
ENSP00000215832	MAPK1	1196	0.805
ENSP00000301764	DDB1	271	0.807
ENSP00000306245	FOS	3356	0.807
ENSP00000333001	RBM8A	30	0.807
ENSP00000252444	LDLR	43	0.808
ENSP00000302269	VAV1	614	0.808
ENSP00000348986	INS-IGF2	685	0.813
ENSP00000352138	KIRREL	253	0.814
ENSP00000324897	UBE2I	2392	0.816
ENSP00000245907	C3	3	0.818
ENSP00000346437	ATG7	4	0.818
ENSP00000381331	HDAC2	335	0.819
ENSP00000262803	UPF1	197	0.82
ENSP00000274255	SKP2	1222	0.825
ENSP00000311677	PPP1R8	379	0.825
ENSP00000357858	BUB3	2011	0.826
ENSP00000209875	CBX5	191	0.829
ENSP00000296871	CSF2	82	0.829
ENSP00000360141	GNAS	3	0.83
ENSP00000284981	APP	1003	0.834
ENSP00000215071	PSMD8	2	0.838

ENSP00000225916	KAT2A	8	0.838
ENSP00000296581	LSM6	1	0.839
ENSP00000274459	ATG12	4	0.84
ENSP00000369213	DDX58	191	0.84
ENSP00000297518	CDK5	349	0.842
ENSP00000247461	CANX	353	0.843
ENSP00000307288	MCM7	64	0.843
ENSP00000268058	PML	234	0.844
ENSP00000300413	SNRPD1	71	0.846
ENSP00000254940	NIP7	190	0.847
ENSP00000400175	RHOA	264	0.847
ENSP00000219548	STUB1	475	0.85
ENSP00000356587	NPHS2	253	0.861
ENSP00000340820	MAPT	349	0.863
ENSP00000254066	RARA	87	0.864
ENSP00000299424	TAF10	3	0.868
ENSP00000303830	INSR	634	0.87
ENSP00000267163	RB1	2596	0.871
ENSP00000319169	PRMT5	1090	0.872
ENSP00000384053	CSF2RB	82	0.874
ENSP00000252102	NDUFA2	1936	0.877
ENSP00000253856	ATP6V0A4	387	0.878

ENSP00000267996	TPM1	17	0.88
ENSP00000361626	YBX1	60	0.883
ENSP00000227758	BIRC2	189	0.884
ENSP00000280665	DCP1B	21	0.885
ENSP00000312735	POLR2B	1372	0.886
ENSP00000371236	GART	17	0.886
ENSP00000011653	CD4	1508	0.888
ENSP00000340944	PTPN11	343	0.888
ENSP00000362592	RBBP4	253	0.891
ENSP00000242057	AHR	377	0.892
ENSP00000244007	PLCG1	1236	0.894
ENSP00000276201	UPF3B	280	0.894
ENSP00000367207	MYC	1638	0.895
ENSP00000249299	NAA38	77	0.896
ENSP00000353483	MAPK8	756	0.897
ENSP00000362649	HDAC1	1847	0.898
ENSP00000368104	BMP2	370	0.898
ENSP00000354826	CALD1	17	0.901
ENSP00000229794	MAPK14	349	0.903
ENSP00000356438	PTGS2	381	0.904
ENSP00000264951	XRN1	1529	0.906
ENSP00000342374	SNRPD2	28	0.906

ENSP00000363921	PARD3	35	0.906
ENSP00000262158	SMAD7	396	0.907
ENSP00000292644	PSMC2	5017	0.907
ENSP00000229239	GAPDH	187	0.908
ENSP00000354394	STAT1	950	0.908
ENSP00000398597	EXOSC6	391	0.91
ENSP00000352264	CD2AP	241	0.913
ENSP00000235090	WDR77	1640	0.914
ENSP00000326031	PPP1CA	189	0.915
ENSP00000348551	NCOR2	37	0.915
ENSP00000223029	AIMP2	191	0.916
ENSP00000337825	LCK	2259	0.917
ENSP00000264279	NOP58	188	0.921
ENSP00000350877	SRSF2	26	0.923
ENSP00000374354	EXOSC8	192	0.926
ENSP00000351407	ARNT	359	0.927
ENSP00000215829	SNRPD3	1115	0.931
ENSP00000346022	RPL9	190	0.931
ENSP00000379625	MYD88	1	0.931
ENSP00000343535	USP7	189	0.932
ENSP00000216605	MTHFD1	17	0.933
ENSP00000242152	NPY	4	0.933

ENSP00000239223	DUSP1	11	0.938
ENSP00000263309	CLNS1A	906	0.938
ENSP00000303242	ITGB2	186	0.939
ENSP00000269349	EIF4A3	197	0.941
ENSP00000315644	TYMS	1470	0.942
ENSP00000380280	FGFR1	441	0.942
ENSP00000356070	MAPKAPK2	6	0.944
ENSP00000314491	SRRT	168	0.948
ENSP00000341551	SMAD4	378	0.948
ENSP00000229264	GNB3	2	0.949
ENSP00000310596	LSM1	83	0.956
ENSP00000337915	CYP3A4	1	0.957
ENSP00000227378	HSPA8	934	0.966
ENSP00000262320	AXIN1	1228	0.968
ENSP00000301280	CHAF1A	191	0.968
ENSP00000368438	PCNA	4300	0.973
ENSP00000414634	LSM2	211	0.974
ENSP00000348708	UPF2	310	0.976
ENSP00000344818	UBC	81033	0.982
ENSP00000252622	LSM7	570	0.984
ENSP00000330393	LEPR	5	0.984
ENSP00000358563	DKC1	379	0.984

ENSP00000369757	RPS6	8	0.986
ENSP00000366135	EXOSC10	2993	0.987
ENSP00000274335	PIK3R1	713	0.988
ENSP00000362820	SRSF3	8	0.989
ENSP00000359345	RPL5	372	0.99
ENSP00000240185	TARDBP	3031	0.991
ENSP00000278916	CHEK1	14	0.991
ENSP00000363676	RPL11	737	0.995
ENSP00000262629	TYROBP	186	1
ENSP00000264998	TF	3	1
ENSP00000296271	RHO	378	1
ENSP00000364898	SYK	382	1
ENSP00000377141	ARRB1	370	1
ENSP00000417281	MDM2	2196	1

5. Candidate genes for microRNA target genes and mRNA genes

Ensembl ID	Gene symbol	Betweenness	Permutation FDR
ENSP00000162330	BCAR1	10607	<0.001
ENSP00000196169	TDRD3	2	<0.001
ENSP00000220478	SCG3	24	<0.001
ENSP00000222792	CHN2	2009	<0.001
ENSP00000248958	SDF2L1	1	<0.001
ENSP00000263253	EP300	60273	<0.001

ENSP00000273258	ARL6IP5	3	<0.001
ENSP00000276420	DOK2	3413	<0.001
ENSP00000280155	ADRA2A	6	<0.001
ENSP00000293831	EIF4A1	2426	<0.001
ENSP00000297261	SHH	10778	<0.001
ENSP00000297784	TMC1	2	<0.001
ENSP00000310723	DDX23	1054	<0.001
ENSP00000317872	RBBP6	1213	<0.001
ENSP00000322791	KIF1A	1	<0.001
ENSP00000330509	DEXI	1	<0.001
ENSP00000332353	PTCH1	17300	<0.001
ENSP00000338217	ZNF532	2	<0.001
ENSP00000339007	GRB2	48282	<0.001
ENSP00000344140	PPP1R32	2	<0.001
ENSP00000349336	FAM163B	1	<0.001
ENSP00000351486	NTRK1	20928	<0.001
ENSP00000356951	C1orf192	5	<0.001
ENSP00000357392	EFNA1	4775	<0.001
ENSP00000360065	WDR78	2	<0.001
ENSP00000361681	SLC25A53	1	<0.001
ENSP00000363727	STARD8	4	<0.001
ENSP00000389427	CEP44	1	<0.001

ENSP00000400806	APTX	4009	<0.001
ENSP00000401303	SHC1	22221	<0.001
ENSP00000231228	IL12B	1213	0.001
ENSP00000240652	IAPP	1213	0.001
ENSP00000254691	CARD6	1213	0.001
ENSP00000266085	TIMP3	2009	0.001
ENSP00000267845	HDC	1998	0.001
ENSP00000269703	CYP4F22	1	0.001
ENSP00000278772	ZNF343	1	0.001
ENSP00000307549	NPTX1	1213	0.001
ENSP00000315173	ZNF41	2006	0.001
ENSP00000329715	DRG1	1213	0.001
ENSP00000331746	CALCA	5064	0.001
ENSP00000337194	PRPF4B	1213	0.001
ENSP00000341848	GOLGB1	1213	0.001
ENSP00000354720	SMC3	10413	0.001
ENSP00000356623	CITED2	4019	0.001
ENSP00000358853	SH3BGRL2	1	0.001
ENSP00000363162	ATP6V1G1	2	0.001
ENSP00000383295	NBEA	17	0.001
ENSP00000384700	PAPOLB	1	0.001
ENSP00000394382	PDHA1	6	0.001

ENSP00000175091	LAPTM4A	1	0.002
ENSP00000204615	THPO	1213	0.002
ENSP00000226091	EFNB3	2011	0.002
ENSP00000250113	FXR2	1	0.002
ENSP00000261037	COL8A1	2816	0.002
ENSP00000264499	BBS7	2432	0.002
ENSP00000266579	SLC38A4	1	0.002
ENSP00000287322	BAG4	1106	0.002
ENSP00000295598	ATP1A1	5585	0.002
ENSP00000296585	ITGA2	12251	0.002
ENSP00000302397	ATP1A3	13	0.002
ENSP00000314810	EDDM3B	1	0.002
ENSP00000315955	FOXA2	4855	0.002
ENSP00000318753	WFDC11	1	0.002
ENSP00000368350	TPT1	6746	0.002
ENSP00000415026	PRRT4	1	0.002
ENSP00000216327	ABHD4	1	0.003
ENSP00000228641	MYF6	1213	0.003
ENSP00000257776	MRAP2	1	0.003
ENSP00000258111	KCNMB4	2	0.003
ENSP00000264554	SHC2	6001	0.003
ENSP00000291386	SSU72	2386	0.003

ENSP00000351141	WTAP	1213	0.003
ENSP00000355566	TOMM20	2418	0.003
ENSP00000406027	EPM2AIP1	1	0.003
ENSP00000161559	CEACAM1	2400	0.004
ENSP00000195654	DOPEY1	3	0.004
ENSP00000228307	PXN	8030	0.004
ENSP00000247026	NSRP1	1212	0.004
ENSP00000256649	TRIM45	1	0.004
ENSP00000269485	TNFRSF11A	3222	0.004
ENSP00000275603	CCT6A	1281	0.004
ENSP00000280193	VEGFC	2807	0.004
ENSP00000293379	ITGA5	25513	0.004
ENSP00000308549	ADORA1	1208	0.004
ENSP00000329748	CPNE8	2	0.004
ENSP00000335612	SPATA21	2	0.004
ENSP00000349960	ACTB	3667	0.004
ENSP00000354033	PCGF2	2010	0.004
ENSP00000354541	NLGN1	4438	0.004
ENSP00000355927	RPS6KC1	2	0.004
ENSP00000357540	FUOM	1	0.004
ENSP00000369003	TRPC4	2	0.004
ENSP00000221086	MTMR9	1	0.005

ENSP00000245451	BMP4	7662	0.005
ENSP00000250111	ATP1B2	1213	0.005
ENSP00000268171	FURIN	4407	0.005
ENSP00000305913	COL8A2	2022	0.005
ENSP00000320303	DNAJC28	1	0.005
ENSP00000328426	FAM208B	1	0.005
ENSP00000335388	KIR3DX1	2	0.005
ENSP00000340672	ARMCX3	1	0.005
ENSP00000351209	EPHA2	7562	0.005
ENSP00000351608	PRRT2	1	0.005
ENSP00000352673	ELF3	1211	0.005
ENSP00000358525	NGF	12630	0.005
ENSP00000360916	VAV2	5999	0.005
ENSP00000372815	C4A	2382	0.005
ENSP00000228567	SYT10	1	0.006
ENSP00000302269	VAV1	12687	0.006
ENSP00000322570	POLE	4229	0.006
ENSP00000328968	SCN5A	1599	0.006
ENSP00000332812	PTGDR2	2	0.006
ENSP00000334940	GGN	1211	0.006
ENSP00000340688	LPHN1	1206	0.006
ENSP00000357311	CENPW	2012	0.006

ENSP00000357674	SNAPIN	1246	0.006
ENSP00000362744	RPS4X	1211	0.006
ENSP00000384169	FBLN2	12	0.006
ENSP00000398930	SGCE	1	0.006
ENSP00000407818	USP46	1	0.006
ENSP00000201586	SULT2B1	2398	0.007
ENSP00000203556	GMIP	5	0.007
ENSP00000245934	SYMPK	3	0.007
ENSP00000248996	GNAZ	2723	0.007
ENSP00000254712	INPP5K	1	0.007
ENSP00000260950	MSTN	2012	0.007
ENSP00000263923	KDR	3782	0.007
ENSP00000293441	SHANK1	1211	0.007
ENSP00000320838	GSG1	1213	0.007
ENSP00000325123	ZSCAN2	1	0.007
ENSP00000327453	ACSM2B	1	0.007
ENSP00000336888	SLC44A2	1	0.007
ENSP00000406674	MOGAT1	3	0.007
ENSP00000231524	TRIM23	1597	0.008
ENSP00000258301	STX6	5633	0.008
ENSP00000261722	AP3B2	1	0.008
ENSP00000300527	COL6A2	1	0.008

ENSP00000324740	YES1	1997	0.008
ENSP00000326200	ZBTB11	1	0.008
ENSP00000326432	CCR8	1213	0.008
ENSP00000338785	STARD13	1916	0.008
ENSP00000349955	RPRD1A	1207	0.008
ENSP00000350256	CCR9	1571	0.008
ENSP00000352413	TMC3	1	0.008
ENSP00000352834	MYO1C	7	0.008
ENSP00000354003	GYPA	2281	0.008
ENSP00000354586	GLI2	4805	0.008
ENSP00000355045	FAM179B	2	0.008
ENSP00000361236	RSPH9	3	0.008
ENSP00000408405	KCTD1	4	0.008
ENSP00000199764	CEACAM6	1600	0.009
ENSP00000266970	CDK2	36082	0.009
ENSP00000298596	STOX1	1	0.009
ENSP00000332504	CCR10	5	0.009
ENSP00000385025	SMCR8	2	0.009
ENSP00000393887	AHSG	1600	0.009
ENSP00000264657	STAT3	59543	0.01
ENSP00000285238	ABCC3	1	0.01
ENSP00000288462	C9orf43	1	0.01

ENSP00000304767	P2RY1	1604	0.01
ENSP00000316329	SCD5	1	0.01
ENSP00000337103	CHAT	3212	0.01
ENSP00000351363	MSMB	1212	0.01
ENSP00000355963	LPGAT1	4	0.01
ENSP00000359819	MOSPD1	2	0.01
ENSP00000363079	MBL2	4376	0.01
ENSP00000407497	SPINK8	3	0.01
ENSP00000220244	KIAA1199	2	0.011
ENSP00000222212	CACNG7	149	0.011
ENSP00000248706	RASL11B	1	0.011
ENSP00000266427	ETV6	2009	0.011
ENSP00000284690	DHX32	1203	0.011
ENSP00000312999	GNAI2	6217	0.011
ENSP00000318472	NCAM1	7212	0.011
ENSP00000322020	SLC25A22	1	0.011
ENSP00000323087	WSCD1	1	0.011
ENSP00000367408	CASK	9142	0.011
ENSP00000406359	HSPA1A	1212	0.011
ENSP00000219091	ZNF205	3	0.012
ENSP00000297290	BRI3	1	0.012
ENSP00000306881	SEC23A	3222	0.012

ENSP00000313581	KLK2	1213	0.012
ENSP00000356652	CACYBP	9	0.012
ENSP00000357453	MAN1A1	29	0.012
ENSP00000364685	MFAP2	4	0.012
ENSP00000366603	TGOLN2	1212	0.012
ENSP00000389813	ACAD10	1	0.012
ENSP00000255305	XPO4	1	0.013
ENSP00000257899	BLOC1S1	3620	0.013
ENSP00000262958	GNA15	1927	0.013
ENSP00000281821	EPHA4	2412	0.013
ENSP00000283977	PGM3	1212	0.013
ENSP00000291536	RSPH1	5	0.013
ENSP00000295727	FEV	1	0.013
ENSP00000297338	RAD21	1899	0.013
ENSP00000327349	TMCC1	5	0.013
ENSP00000347839	RAB11FIP2	2420	0.013
ENSP00000360183	STX16	2419	0.013
ENSP00000365160	NUDT11	1	0.013
ENSP00000382713	UBE2QL1	1	0.013
ENSP00000175506	ASNS	3954	0.014
ENSP00000230895	DAP	1211	0.014
ENSP00000247170	DAAM1	1600	0.014

ENSP00000270001	ZFP14	1	0.014
ENSP00000278198	LRRC4C	2011	0.014
ENSP00000283147	BMP6	1593	0.014
ENSP00000339845	DROSHA	1211	0.014
ENSP00000340507	TRIM24	1213	0.014
ENSP00000352936	SPINK5	1	0.014
ENSP00000356789	ATP1B1	2395	0.014
ENSP00000359505	C6orf57	1	0.014
ENSP00000359910	PSMA7	1212	0.014
ENSP00000364700	OMD	1	0.014
ENSP00000369344	GPR150	1	0.014
ENSP00000302150	PRL	9777	0.015
ENSP00000309968	ADAM17	2002	0.015
ENSP00000323280	CD6	1568	0.015
ENSP00000326261	SRRM1	2011	0.015
ENSP00000328397	VMO1	1	0.015
ENSP00000357244	CCT3	2007	0.015
ENSP00000365426	GGACT	1	0.015
ENSP00000406219	PPT2	2	0.015
ENSP00000222725	LFNG	3573	0.016
ENSP00000299333	SCN3B	1	0.016
ENSP00000299578	C16orf46	1	0.016

ENSP00000332171	DMTF1	1	0.016
ENSP00000346886	GABPA	1212	0.016
ENSP00000204604	CHRD	3603	0.017
ENSP00000219172	CENPT	2011	0.017
ENSP00000273347	NXPE3	2	0.017
ENSP00000309622	TFDP2	1212	0.017
ENSP00000359151	DBT	1603	0.017
ENSP00000360891	IFIT2	1210	0.017
ENSP00000369292	SAMD9	1	0.017
ENSP00000332674	LRRC8B	1	0.018
ENSP00000341151	ZNF81	4	0.018
ENSP00000344431	DNAJB13	2	0.018
ENSP00000348349	MYO9A	2	0.018
ENSP00000350990	TNKS1BP1	1211	0.018
ENSP00000394624	OPRM1	3526	0.018
ENSP00000229729	SLC44A4	1	0.019
ENSP00000244007	PLCG1	22700	0.019
ENSP00000249363	LRRC4	2	0.019
ENSP00000285039	MYO5B	6	0.019
ENSP00000288139	CACNA1D	3	0.019
ENSP00000295709	STK36	1595	0.019
ENSP00000315167	ALOX12B	2003	0.019

ENSP00000328983	CHST6	1	0.019
ENSP00000359285	CHRNA4	2390	0.019
ENSP00000384179	ZFPM2	2	0.019
ENSP00000265164	CASP6	1213	0.02
ENSP00000286760	WHAMM	1	0.02
ENSP00000333633	MTA1	3583	0.02
ENSP00000360217	RHAG	2276	0.02
ENSP00000363229	SLC18A3	2	0.02
ENSP00000370408	CDX2	2011	0.02
ENSP00000375907	PID1	1	0.02
ENSP00000242067	BBS9	3623	0.021
ENSP00000306407	DBNDD1	3	0.021
ENSP00000316476	DEGS1	4	0.021
ENSP00000342011	XRCC4	3612	0.021
ENSP00000344460	CBS	1982	0.021
ENSP00000361423	ABL1	11508	0.021
ENSP00000225655	PFN1	3014	0.022
ENSP00000233057	EIF2AK2	2456	0.022
ENSP00000257963	ACVR1B	2012	0.022
ENSP00000297562	AP5Z1	1213	0.022
ENSP00000413234	AP2A2	3367	0.022
ENSP00000254227	NR0B2	3221	0.023

ENSP00000255688	RARRES3	1119	0.023
ENSP00000256689	SLC38A2	1198	0.023
ENSP00000261407	LPCAT3	4	0.023
ENSP00000264033	CBL	164841	0.023
ENSP00000283415	LPCAT1	9	0.023
ENSP00000298292	DNAAF2	1	0.023
ENSP00000346440	TCF4	1213	0.023
ENSP00000354777	TBKBP1	1211	0.023
ENSP00000413720	CDKN1C	1589	0.023
ENSP00000254661	RAMP1	1208	0.024
ENSP00000255194	AP3B1	1	0.024
ENSP00000262776	LGALS3BP	1600	0.024
ENSP00000267116	ANKRD52	1	0.024
ENSP00000269571	ERBB2	14171	0.024
ENSP00000305595	B3GNT2	1205	0.024
ENSP00000346550	ANXA6	3	0.024
ENSP00000407674	HLA-DPB1	2	0.024
ENSP00000226413	GNRHR	1	0.025
ENSP00000273398	ATP6V1A	1155	0.025
ENSP00000331474	SYNDIG1L	2	0.025
ENSP00000358497	RNGTT	2011	0.025
ENSP00000365766	TIMM17B	1206	0.025

ENSP00000223023	WASL	15013	0.026
ENSP00000257497	ANXA1	2002	0.026
ENSP00000322142	ING5	2426	0.026
ENSP00000333019	ADSSL1	1	0.026
ENSP00000337396	CCNB1IP1	1	0.026
ENSP00000347710	OPHN1	1204	0.026
ENSP00000350941	SRC	59670	0.026
ENSP00000019103	SCTR	1587	0.027
ENSP00000222250	ARRDC2	3	0.027
ENSP00000261483	MAN2A1	4	0.027
ENSP00000276185	FRMPD3	1	0.027
ENSP00000303423	FNTA	1213	0.027
ENSP00000306512	IL8	13870	0.027
ENSP00000316794	ZCCHC5	2	0.027
ENSP00000330005	RGMA	4	0.027
ENSP00000341963	RSC1A1	1	0.027
ENSP00000404524	HSPA1A	1	0.027
ENSP00000261326	MOCOS	1	0.028
ENSP00000296140	CCR1	3607	0.028
ENSP00000339151	IKBKB	2463	0.028
ENSP00000357656	FYN	39355	0.028
ENSP00000363092	PRKG1	4861	0.028

ENSP00000367125	GPR153	1	0.028
ENSP00000367959	HTR2A	40	0.028
ENSP00000400513	FASTKD1	1	0.028
ENSP00000403557	PPP1R11	1213	0.028
ENSP00000225844	CCL13	1571	0.029
ENSP00000240874	KALRN	1213	0.029
ENSP00000259089	BLK	2005	0.029
ENSP00000299421	ILK	7099	0.029
ENSP00000300417	LRSAM1	800	0.029
ENSP00000340944	PTPN11	11563	0.029
ENSP00000405738	ESRP1	1	0.029
ENSP00000043402	RTN4R	2806	0.03
ENSP00000216410	GNPNAT1	1202	0.03
ENSP00000315137	SGPP2	1	0.03
ENSP00000352442	HIST1H2BM	800	0.03
ENSP00000357692	S100A16	1209	0.03
ENSP00000357753	IVL	2804	0.03
ENSP00000361125	VEGFA	14779	0.03
ENSP00000366563	PIK3CD	1982	0.03
ENSP00000383690	MASP2	2382	0.03
ENSP00000258711	CHST12	2	0.031
ENSP00000261622	SLC7A5	2	0.031

ENSP00000274711	LRRTM2	1208	0.031
ENSP00000331902	COL4A5	1595	0.031
ENSP00000340083	KRCC1	1	0.031
ENSP00000362795	CXCR3	1971	0.031
ENSP00000170630	IL4R	4399	0.032
ENSP00000260382	LRRC49	2	0.032
ENSP00000284154	GRAP	1127	0.032
ENSP00000323880	FOXJ1	6	0.032
ENSP00000359206	BTRC	2439	0.032
ENSP00000361548	MPL	1213	0.032
ENSP00000367747	PLCH2	1203	0.032
ENSP00000221283	STXBP2	800	0.033
ENSP00000238256	FKBP15	785	0.033
ENSP00000245539	MRPS7	799	0.033
ENSP00000252593	BST2	799	0.033
ENSP00000310658	SCUBE2	799	0.033
ENSP00000382791	GRIK1	1997	0.033
ENSP00000316460	FYB	2864	0.034
ENSP00000327916	ACSM5	5	0.034
ENSP00000339390	CDH26	1	0.034
ENSP00000344352	ATF3	10864	0.034
ENSP00000379203	ATP6V1C1	1	0.034

ENSP00000386171	ESRRG	1213	0.034
ENSP00000253669	HAUS8	3	0.035
ENSP00000355361	CD47	2272	0.035
ENSP00000356529	RGS16	1213	0.035
ENSP00000357025	CD48	800	0.035
ENSP00000357721	S100A8	1582	0.035
ENSP00000381634	SLC38A1	8	0.035
ENSP00000387662	GCG	15143	0.035
ENSP00000241463	RASL11A	5	0.036
ENSP00000246032	STK35	2006	0.036
ENSP00000261267	LYZ	2207	0.036
ENSP00000278616	ATM	14772	0.036
ENSP00000321826	STXBP5	1208	0.036
ENSP00000354900	GJB1	799	0.036
ENSP00000355731	CDC42BPA	6	0.036
ENSP00000371682	DCAF16	1215	0.036
ENSP00000396439	RING1	2006	0.036
ENSP00000264009	HSF4	800	0.037
ENSP00000264234	UPK1B	1600	0.037
ENSP00000322427	ZNF611	2	0.037
ENSP00000386165	CEBPD	2320	0.037
ENSP00000205636	CMTM6	2	0.038

ENSP00000206249	ESR1	101905	0.038
ENSP00000250894	MAPK8IP3	1213	0.038
ENSP00000257829	NAT10	447	0.038
ENSP00000260630	CYP1B1	1585	0.038
ENSP00000262188	SMARCD3	1916	0.038
ENSP00000311313	CST6	2	0.038
ENSP00000343819	OTX2	800	0.038
ENSP00000350332	MYBPC2	800	0.038
ENSP00000377840	CACNB1	801	0.038
ENSP00000218652	NDFIP2	1206	0.039
ENSP00000219409	ARHGDIG	787	0.039
ENSP00000277541	NOTCH1	27278	0.039
ENSP00000285398	ERCC3	1166	0.039
ENSP00000293272	CCL5	5309	0.039
ENSP00000320604	FAXDC2	1209	0.039
ENSP00000355599	TSNAX	2009	0.039
ENSP00000384675	SOS1	4306	0.039
ENSP00000233202	SLC11A1	800	0.04
ENSP00000260867	TIMM23	2050	0.04
ENSP00000263025	MAPK3	1387	0.04
ENSP00000264001	CKLF	800	0.04
ENSP00000278499	SESN3	1	0.04

ENSP00000305988	ALCAM	1568	0.04
ENSP00000314813	OAZ1	4394	0.04
ENSP00000356906	SH2D1B	800	0.04
ENSP00000369009	CXorf23	800	0.04
ENSP00000227618	ANAPC15	1208	0.041
ENSP00000268704	SPG7	800	0.041
ENSP00000276533	GINS4	102	0.041
ENSP00000304283	RAC3	1997	0.041
ENSP00000304915	IL13	2009	0.041
ENSP00000319851	CHDH	5	0.041
ENSP00000344456	CTNNB1	60756	0.041
ENSP00000363431	NPY4R	794	0.041
ENSP00000365877	SUV39H1	798	0.041
ENSP00000369962	IGSF5	799	0.041
ENSP00000370503	CCM2	2010	0.041
ENSP00000400591	SNRPE	1314	0.041
ENSP00000216373	SOS2	800	0.042
ENSP00000246194	RALY	1153	0.042
ENSP00000264126	GPSM2	1600	0.042
ENSP00000265529	KIF9	1202	0.042
ENSP00000302961	HSPA4	5886	0.042
ENSP00000324944	MBOAT1	18	0.042

ENSP00000338728	CCDC88A	799	0.042
ENSP00000339428	SOCS2	61	0.042
ENSP00000347198	SRGAP1	2787	0.042
ENSP00000352785	DSG4	798	0.042
ENSP00000362166	MEAF6	2426	0.042
ENSP00000377446	SUCLG1	2415	0.042
ENSP00000301420	KLK1	800	0.043
ENSP00000362298	SGPL1	3	0.043
ENSP00000368686	E2F4	1016	0.043
ENSP00000264079	MCOLN1	1210	0.044
ENSP00000290541	PSMB4	5586	0.044
ENSP00000292301	CCR2	102	0.044
ENSP00000299163	HIF1AN	1212	0.044
ENSP00000330289	TRAPPC6B	1	0.044
ENSP00000241256	GHSR	1360	0.045
ENSP00000265310	TRPV5	797	0.045
ENSP00000283871	HGD	800	0.045
ENSP00000334122	FGF3	4397	0.045
ENSP00000339916	LIMK2	1209	0.045
ENSP00000346148	PRKAA1	1213	0.045
ENSP00000350012	ACSL3	5	0.045
ENSP00000359719	PRKACB	1	0.045

ENSP00000385021	FANCL	1210	0.045
ENSP00000017003	XYLT2	1	0.046
ENSP00000265294	GABRP	800	0.046
ENSP00000267842	SLC27A2	772	0.046
ENSP00000298310	NEMF	1	0.046
ENSP00000301258	PSCA	799	0.046
ENSP00000302707	FPR1	1957	0.046
ENSP00000312652	LEP	22731	0.046
ENSP00000334594	SLC10A7	1210	0.046
ENSP00000335632	CHP1	1213	0.046
ENSP00000340396	GBP5	804	0.046
ENSP00000361507	HYI	2	0.046
ENSP00000405890	PBX1	2805	0.046
ENSP00000248071	KLF2	799	0.047
ENSP00000255465	CCNA1	2010	0.047
ENSP00000261783	ARG2	3922	0.047
ENSP00000297494	NOS3	30057	0.047
ENSP00000304604	MAGI3	785	0.047
ENSP00000316990	TRAPPC5	799	0.047
ENSP00000355560	TBCE	1	0.047
ENSP00000356433	UST	1	0.047
ENSP00000364246	PLA2G2D	793	0.047

ENSP00000364398	HABP4	1211	0.047
ENSP00000168712	FGF4	803	0.048
ENSP00000233616	MOGS	799	0.048
ENSP00000261047	GUCA1C	3	0.048
ENSP00000281928	MED13L	1212	0.048
ENSP00000284995	TSEN2	1213	0.048
ENSP00000300134	STAT6	8134	0.048
ENSP00000337722	ARL6	1211	0.048
ENSP00000373485	TSNAXIP1	798	0.048
ENSP00000244625	TBCC	1	0.049
ENSP00000281030	THRSP	799	0.049
ENSP00000299140	SPATA19	2	0.049
ENSP00000328938	AFMID	1	0.049
ENSP00000358857	EMD	2009	0.049
ENSP00000365756	KIAA2013	1	0.049
ENSP00000365811	SPAG6	1212	0.049
ENSP00000420418	ZNF398	796	0.049
ENSP00000245121	KATNAL2	1	0.05
ENSP00000345793	ZC3H7B	798	0.05
ENSP00000353408	MSN	1600	0.05
ENSP00000355001	POU3F3	799	0.05
ENSP00000370192	SLC46A3	1	0.05

ENSP00000371432	PRLR	9466	0.05
ENSP00000226574	NFKB1	2492	0.051
ENSP00000288986	NCK1	6515	0.051
ENSP00000294954	LHCGR	3	0.051
ENSP00000298032	ARMC3	799	0.051
ENSP00000320340	DGKZ	781	0.051
ENSP00000339057	GPRASP2	5	0.051
ENSP00000359594	CLCA4	800	0.051
ENSP00000245323	EFNB2	2049	0.052
ENSP00000263209	DGCR8	1211	0.052
ENSP00000291582	AIRE	1211	0.052
ENSP00000310978	AGXT2L2	1	0.052
ENSP00000353654	COL4A2	1588	0.052
ENSP00000361726	WFDC10A	2	0.052
ENSP00000368450	CD83	799	0.052
ENSP00000262848	PRKX	1	0.053
ENSP00000298386	RXFP2	800	0.053
ENSP00000318406	KIAA0319L	798	0.053
ENSP00000395465	NCOA4	799	0.053
ENSP00000005587	SKAP2	800	0.054
ENSP00000258774	HUS1	1213	0.054
ENSP00000345728	ATP7A	1214	0.054

ENSP00000360302	GRIA3	798	0.054
ENSP00000362095	SRPX2	800	0.054
ENSP00000396620	NFYC	1988	0.054
ENSP00000310447	GLS2	4	0.055
ENSP00000348107	C1D	2007	0.055
ENSP00000394033	KCNK2	1211	0.055
ENSP00000225893	HNF1B	1209	0.056
ENSP00000264452	TMEM33	1	0.056
ENSP00000274793	PLA2G7	799	0.056
ENSP00000297107	GALNT10	798	0.056
ENSP00000297373	PHKG1	1204	0.056
ENSP00000337675	EBAG9	2	0.056
ENSP00000349275	NRG1	2678	0.056
ENSP00000350198	SSTR2	1211	0.056
ENSP00000354130	SOX10	1597	0.056
ENSP00000360696	LCN12	1	0.056
ENSP00000361917	ARMCX1	1	0.056
ENSP00000206542	OSGEP	2008	0.057
ENSP00000255380	CHRM3	7	0.057
ENSP00000329757	ATP6V0C	800	0.057
ENSP00000336790	ATF4	9670	0.057
ENSP00000354376	RAB25	1208	0.057

ENSP00000375777	STRN4	5622	0.057
ENSP00000375859	TMEM91	735	0.057
ENSP00000266659	GLIPR1	799	0.058
ENSP00000278823	MTA2	797	0.058
ENSP00000297564	COX6C	2010	0.058
ENSP00000327417	GPR39	798	0.058
ENSP00000328216	ORAI1	1213	0.058
ENSP00000371475	TP53BP1	1213	0.058
ENSP00000374981	IGHA2	799	0.058
ENSP00000262304	PKD1	1915	0.059
ENSP00000272238	ATP6V1C2	1582	0.059
ENSP00000307218	NAT1	799	0.059
ENSP00000315465	DSCAML1	1	0.059
ENSP00000326830	CLK1	1212	0.059
ENSP00000327801	P4HB	2791	0.059
ENSP00000340088	THEG	1596	0.059
ENSP00000355316	GRM3	1	0.059
ENSP00000396915	SCN1B	1515	0.059
ENSP00000156626	ST6GALNAC1	3	0.06
ENSP00000228872	CDKN1B	12300	0.06
ENSP00000322229	FADS1	1210	0.06
ENSP00000328422	PP13439	1	0.06

ENSP00000343331	LIPI	1	0.06
ENSP00000347942	RET	4665	0.06
ENSP00000351790	MYPN	39	0.06
ENSP00000263817	ABCB11	2802	0.061
ENSP00000302114	PRELID1	800	0.061
ENSP00000320623	RMND5B	1	0.061
ENSP00000328364	MAFA	799	0.061
ENSP00000369647	AVP	2504	0.061
ENSP00000398852	SLC44A4	790	0.061
ENSP00000005226	USH1C	1614	0.062
ENSP00000168216	HSD17B10	1210	0.062
ENSP00000249647	SNAP23	6416	0.062
ENSP00000254301	LGALS3	1600	0.062
ENSP00000265717	PRKAR2B	19	0.062
ENSP00000311579	TNKS	1211	0.062
ENSP00000362353	GLP1R	4	0.063
ENSP00000319664	NUDC	44	0.064
ENSP00000348786	RAP1A	3997	0.064
ENSP00000352097	SPTSSB	1	0.064
ENSP00000374323	EPHA6	1204	0.064
ENSP00000317300	LPCAT4	6	0.065
ENSP00000337144	AQP12A	1	0.065

ENSP00000385057	APOBEC3G	1213	0.065
ENSP00000246041	AP5S1	1212	0.066
ENSP00000289359	MITD1	2	0.066
ENSP00000410294	FGFR2	3200	0.066
ENSP00000261170	GUCY2C	800	0.067
ENSP00000263640	ACVR1	795	0.067
ENSP00000288912	WDR66	1	0.067
ENSP00000304102	COPS6	1201	0.067
ENSP00000310170	FOSL1	3599	0.067
ENSP00000362014	DNM1	3639	0.067
ENSP00000363616	EDEM2	1	0.067
ENSP00000377958	CCT4	2386	0.067
ENSP00000254998	NXT1	3223	0.068
ENSP00000263697	DNAJC8	1209	0.068
ENSP00000310216	KLRC4	799	0.068
ENSP00000347046	PDE5A	27	0.068
ENSP00000414237	INTS2	1209	0.068
ENSP00000303325	TACR3	39	0.069
ENSP00000305714	BMP1	1598	0.069
ENSP00000320567	MRPS33	1212	0.069
ENSP00000321345	IL23R	239	0.069
ENSP00000365643	DOCK9	772	0.069

ENSP00000370938	CDK8	3536	0.069
ENSP00000375921	PAX3	2391	0.069
ENSP00000262545	PCSK2	800	0.07
ENSP00000234739	BCL9	1596	0.071
ENSP00000282344	USP12	2006	0.071
ENSP00000304704	CLP1	1213	0.071
ENSP00000306245	FOS	35920	0.071
ENSP00000313829	KHDRBS1	38590	0.071
ENSP00000342554	STX2	12	0.071
ENSP00000349467	CALM1	28487	0.071
ENSP00000316244	HTR1A	1607	0.072
ENSP00000323568	SLC2A2	1592	0.072
ENSP00000352035	KCNQ2	2	0.072
ENSP00000404232	EFHC2	1210	0.072
ENSP00000247655	COX7C	800	0.073
ENSP00000344871	MYO1F	1196	0.073
ENSP00000260643	PREB	800	0.074
ENSP00000265459	NRXN2	1119	0.074
ENSP00000310557	KCNE3	705	0.074
ENSP00000353910	FUT8	7	0.074
ENSP00000358099	RGS10	2	0.074
ENSP00000240093	FZD3	800	0.075

ENSP00000256759	FST	2021	0.075
ENSP00000261890	RAB11A	2413	0.075
ENSP00000263369	MIA	798	0.075
ENSP00000295728	CRYBA2	795	0.075
ENSP00000311218	C1orf100	1	0.075
ENSP00000350162	SYCP2	1595	0.075
ENSP00000219244	CCL17	1205	0.076
ENSP00000236147	SELL	2011	0.076
ENSP00000236671	CTSD	1213	0.076
ENSP00000360268	ALDH18A1	1988	0.076
ENSP00000372991	LTA	1600	0.076
ENSP00000419923	KLF6	1212	0.076
ENSP00000255945	GIMAP4	800	0.077
ENSP00000260383	TUBGCP4	796	0.077
ENSP00000339521	RSU1	800	0.077
ENSP00000354623	DFNB31	2007	0.077
ENSP00000361473	KDM4A	1212	0.077
ENSP00000362082	CCND3	2801	0.077
ENSP00000006053	CX3CL1	1584	0.078
ENSP00000286604	UGT2A1	4	0.078
ENSP00000305529	SIRPG	2080	0.078
ENSP00000320130	DYNC1I1	1598	0.078

ENSP00000333920	TTF1	799	0.078
ENSP00000345096	IMPDH1	797	0.078
ENSP00000361867	SEMG1	799	0.078
ENSP00000242839	ATP7B	800	0.079
ENSP00000245157	BBS2	3619	0.079
ENSP00000295066	DPY30	1213	0.079
ENSP00000339016	ATAD1	2	0.079
ENSP00000363390	TRIM63	470	0.079
ENSP00000217381	SNTA1	1598	0.08
ENSP00000252945	CYP2E1	4415	0.08
ENSP00000256379	MED6	1283	0.08
ENSP00000289429	CD1A	800	0.08
ENSP00000303019	GPHN	4429	0.08
ENSP00000308496	ARL10	2	0.08
ENSP00000340328	NYX	1	0.08
ENSP00000352522	ATP6V1H	1211	0.08
ENSP00000228837	FGF6	4793	0.081
ENSP00000321326	F2R	1738	0.081
ENSP00000348211	SLC25A46	1	0.081
ENSP00000358576	DCLRE1B	798	0.081
ENSP00000414598	MRVI1	798	0.081
ENSP00000219281	USB1	799	0.082

ENSP00000261558	AP5M1	1212	0.082
ENSP00000303077	GOT1L1	796	0.082
ENSP00000316854	ATOX1	2009	0.082
ENSP00000376822	STEAP3	1210	0.082
ENSP00000216129	TTLL12	1	0.083
ENSP00000227507	CCND1	55513	0.083
ENSP00000251849	RAF1	23475	0.083
ENSP00000296802	NSA2	3	0.083
ENSP00000311697	FGF5	800	0.083
ENSP00000252050	CUL9	795	0.084
ENSP00000258682	CAMK2B	799	0.084
ENSP00000276072	TAF1	795	0.084
ENSP00000294973	HAAO	800	0.084
ENSP00000319197	OR4K17	12	0.084
ENSP00000348986	INS-IGF2	12656	0.084
ENSP00000368688	LYRM7	1	0.084
ENSP00000256594	GSTM3	12	0.085
ENSP00000259938	CLPS	800	0.085
ENSP00000342755	RNF41	2795	0.085
ENSP00000389792	DCDC1	800	0.085
ENSP00000392828	GPSM1	799	0.085
ENSP00000260600	ADCY3	797	0.086

ENSP00000278385	CD44	1650	0.086
ENSP00000286827	TIAM1	1616	0.086
ENSP00000356104	PM20D1	1	0.086
ENSP00000357033	CD84	800	0.086
ENSP00000184266	NDUFB4	11	0.087
ENSP00000240055	NFYB	1986	0.087
ENSP00000262965	TCF3	6380	0.087
ENSP00000274787	HIGD2A	1	0.087
ENSP00000318775	ANAPC4	1208	0.087
ENSP00000242152	NPY	4065	0.088
ENSP00000296084	RYK	1202	0.088
ENSP00000334424	AMACR	10	0.088
ENSP00000348573	AKAP9	23	0.088
ENSP00000363822	AR	10634	0.088
ENSP00000381822	CDH23	14	0.088
ENSP00000232458	ECT2	867	0.089
ENSP00000265351	XPO5	1213	0.089
ENSP00000265944	MYO3A	1	0.089
ENSP00000359174	SLC35A3	1206	0.089
ENSP00000366593	TMEM201	799	0.089
ENSP00000380378	PAFAH1B1	1230	0.089
ENSP00000299339	CLDN10	1193	0.09

ENSP00000304553	MPLKIP	799	0.09
ENSP00000223208	CEP41	1197	0.091
ENSP00000276603	TERF1	2818	0.091
ENSP00000331172	CD8B	602	0.091
ENSP00000265132	AMBP	2332	0.092
ENSP00000297135	COG5	798	0.092
ENSP00000346693	ELOVL2	2	0.092
ENSP00000361855	SEMG2	1	0.092
ENSP00000380227	ITGA4	6263	0.092
ENSP00000388724	HLA-A	800	0.092
ENSP00000220876	STMN2	799	0.093
ENSP00000264839	RIMS1	2432	0.093
ENSP00000300061	SCNN1G	798	0.093
ENSP00000316578	SUZ12	2012	0.093
ENSP00000331514	ACTG1	1213	0.093
ENSP00000335203	ATPIF1	1	0.093
ENSP00000344741	INSIG1	1213	0.093
ENSP00000371070	ATP8A2	1	0.093
ENSP00000239223	DUSP1	541	0.094
ENSP00000261182	NAP1L1	797	0.094
ENSP00000292823	PCYT1A	1	0.094
ENSP00000378326	ZP3	800	0.094

ENSP00000401632	GSTT1	791	0.094
ENSP00000304822	CSN3	572	0.095
ENSP00000314458	CDC42	18124	0.095
ENSP00000332247	ATP6V0A2	799	0.095
ENSP00000350869	ZNF346	799	0.095
ENSP00000355896	TGFB2	800	0.095
ENSP00000247461	CANX	9221	0.096
ENSP00000264010	CTCF	800	0.096
ENSP00000273390	MAATS1	799	0.096
ENSP00000319591	KCND3	1	0.096
ENSP00000325660	CNTN1	1515	0.096
ENSP00000356375	ZBTB41	2	0.096
ENSP00000360992	STAMBPL1	2	0.096
ENSP00000400175	RHOA	8404	0.096
ENSP00000272233	RHOB	1211	0.097
ENSP00000368678	AGRN	13	0.097
ENSP00000245457	PTGER2	8	0.098
ENSP00000266744	ASCL1	1207	0.098
ENSP00000331302	TCEB3B	1	0.098
ENSP00000359892	SLC44A5	4	0.098
ENSP00000362372	BRWD3	798	0.098
ENSP00000229201	TIMELESS	799	0.099

ENSP00000259455	GABBR2	12	0.099
ENSP00000262053	ATF1	1189	0.099
ENSP00000343785	SPRY1	799	0.099
ENSP00000255882	PI4KA	1	0.1
ENSP00000270257	GEMIN7	1	0.1
ENSP00000274311	PELO	799	0.1
ENSP00000315011	EDNRA	1590	0.1
ENSP00000353701	DPP3	1214	0.1
ENSP00000244741	CDKN1A	5622	0.101
ENSP00000252997	GATA5	1210	0.101
ENSP00000274031	SETD7	799	0.101
ENSP00000303686	NTSR2	13	0.101
ENSP00000347733	TRRAP	3752	0.101
ENSP00000350447	SGOL2	794	0.101
ENSP00000368169	DVL1	3999	0.101
ENSP00000281950	GEMIN6	1212	0.102
ENSP00000298281	PCF11	1207	0.102
ENSP00000298472	SLC18A2	799	0.102
ENSP00000355180	COL6A1	3	0.102
ENSP00000238112	CPSF3	1213	0.103
ENSP00000329968	PHKG2	794	0.103
ENSP00000357066	ARG1	2	0.103

ENSP00000360371	SSBP3	788	0.103
ENSP00000360645	MAN1B1	1179	0.103
ENSP00000363642	BMS1	1	0.103
ENSP00000386331	MYO7A	1604	0.103
ENSP00000262764	PGS1	3	0.104
ENSP00000289902	FCER1G	2397	0.104
ENSP00000301096	ZNF83	1	0.104
ENSP00000324274	ZCCHC10	1209	0.104
ENSP00000350878	S1PR3	1550	0.104
ENSP00000356975	ADAMTS4	800	0.104
ENSP00000393275	NANOS1	1	0.104
ENSP00000248933	SEZ6L	1	0.105
ENSP00000296412	ADH5	52	0.105
ENSP00000345656	VAPA	1213	0.105
ENSP00000388340	CLINT1	797	0.105
ENSP00000232461	GNAT1	3220	0.106
ENSP00000268182	IQGAP1	2556	0.106
ENSP00000287727	ZFYVE9	8486	0.106
ENSP00000311219	TRIM59	799	0.106
ENSP00000324834	MUC3A	798	0.106
ENSP00000361824	SPTAN1	6	0.106
ENSP00000243077	LRP1	1460	0.107

ENSP00000215832	MAPK1	16427	0.108
ENSP00000225603	CBX1	1213	0.108
ENSP00000255389	PEMT	3	0.108
ENSP00000308576	RHOD	4	0.108
ENSP00000349293	B4GALT2	8	0.108
ENSP00000354119	LAT	1127	0.108
ENSP00000354497	FAN1	1203	0.108
ENSP00000354532	PNP	40	0.108
ENSP00000382166	CX3CR1	1584	0.108
ENSP00000164133	PPP2R5B	1200	0.109
ENSP00000295006	CAPN2	799	0.109
ENSP00000306003	ATP5I	1	0.109
ENSP00000334188	PFDN5	4387	0.109
ENSP00000271411	POU2F1	1208	0.11
ENSP00000343782	ADRB3	8	0.11
ENSP00000256383	EIF2S1	4809	0.111
ENSP00000264708	POMC	9280	0.111
ENSP00000265689	CHKA	3	0.111
ENSP00000265729	SRI	1213	0.111
ENSP00000281923	MGAT5	2	0.111
ENSP00000292174	CXCR5	2	0.111
ENSP00000298532	SNAPC4	1205	0.111

ENSP00000325136	HADHB	655	0.111
ENSP00000417164	ROBO2	800	0.111
ENSP00000262613	SLC9A3R1	23414	0.112
ENSP00000307491	WDR48	2006	0.112
ENSP00000327541	BLZF1	1104	0.112
ENSP00000281708	FBXW7	2599	0.113
ENSP00000283109	RIOK2	1	0.113
ENSP00000290551	BTG2	800	0.113
ENSP00000314080	HIC1	800	0.113
ENSP00000361658	NUP188	795	0.113
ENSP00000363533	MDH1B	1	0.113
ENSP00000313199	HNRNPD	3613	0.114
ENSP00000365048	TNFSF13B	800	0.114
ENSP00000378288	MYLK3	1211	0.114
ENSP00000311010	ELF5	1	0.115
ENSP00000345731	DLG1	4016	0.115
ENSP00000360054	PHACTR3	799	0.115
ENSP00000363163	NLGN3	2	0.115
ENSP00000376921	NTNG2	2	0.115
ENSP00000250615	AANAT	25	0.116
ENSP00000284562	GSTA5	2	0.116
ENSP00000318852	BPNT1	2	0.116

ENSP00000340677	WNT8B	2	0.116
ENSP00000155840	KCNQ1	1519	0.117
ENSP00000272430	RTKN	800	0.117
ENSP00000292169	S100A1	809	0.117
ENSP00000360672	PARD6B	1212	0.117
ENSP00000265447	ANXA11	798	0.118
ENSP00000307939	GCC2	1204	0.118
ENSP00000327145	FLNC	1215	0.118
ENSP00000220966	PYCRL	1	0.119
ENSP00000223140	NOBOX	796	0.119
ENSP00000294053	CLPB	1189	0.119
ENSP00000358994	MYO6	1235	0.119
ENSP00000366977	PLEKHG5	7	0.119
ENSP00000216223	IL2RB	5109	0.12
ENSP00000356000	PLXNA2	1585	0.12
ENSP00000365569	FLOT1	800	0.12
ENSP00000216832	PNN	2426	0.121
ENSP00000272252	GALM	2	0.121
ENSP00000357748	BCCIP	1203	0.121
ENSP00000257789	ORC3	1212	0.122
ENSP00000257879	ITGA7	800	0.122
ENSP00000303634	LRP8	135	0.122

ENSP00000352708	TRAPPC2	1211	0.122
ENSP00000356056	DYNLT1	799	0.122
ENSP00000360871	FCN1	1	0.122
ENSP00000391457	INO80C	799	0.122
ENSP00000280357	IL18	1597	0.123
ENSP00000308461	RND1	2387	0.123
ENSP00000349436	ADAM15	789	0.123
ENSP00000265080	RASGRF2	2	0.124
ENSP00000347409	KEL	800	0.124
ENSP00000351284	RAD52	800	0.124
ENSP00000375863	HNRNPUL1	1213	0.124
ENSP00000309052	CATSPER1	800	0.125
ENSP00000362578	RNF8	1205	0.125
ENSP00000380942	ARHGEF12	3987	0.125
ENSP00000222139	EPOR	1213	0.126
ENSP00000255608	BTBD2	786	0.126
ENSP00000256958	SLCO1B1	2798	0.126
ENSP00000259206	IL1RN	800	0.126
ENSP00000320866	CALR	7117	0.126
ENSP00000329684	GALR2	1	0.126
ENSP00000336701	RAD51C	1988	0.126
ENSP00000225941	ABI3	799	0.127

ENSP00000389184	MARK2	1213	0.127
ENSP00000417132	BAP1	2426	0.127
ENSP00000265986	IDE	4	0.128
ENSP00000260570	IFT172	793	0.129
ENSP00000348394	NCDN	797	0.129
ENSP00000361646	ZMYND12	784	0.129
ENSP00000370223	IDH3B	7	0.129
ENSP00000335153	HSP90AA1	44969	0.13
ENSP00000338369	CELA3B	197	0.13
ENSP00000350937	TES	800	0.13
ENSP00000357177	ARHGEF11	1601	0.13
ENSP00000370962	GGT6	800	0.13
ENSP00000406751	CRY2	1	0.13
ENSP00000282903	PLOD2	2	0.131
ENSP00000296490	WDR82	1210	0.131
ENSP00000303211	ACHE	3	0.131
ENSP00000314444	WDR35	1	0.131
ENSP00000216064	SUN2	794	0.132
ENSP00000247866	NDUFB2	1213	0.132
ENSP00000265969	KCNC1	1025	0.132
ENSP00000298130	SPTSSA	1207	0.132
ENSP00000303830	INSR	12111	0.132

ENSP00000353165	TPK1	800	0.132
ENSP00000380702	MYCBP	7141	0.132
ENSP00000263980	SLC9A1	1213	0.133
ENSP00000273480	RNF7	2	0.133
ENSP00000278947	SCN2B	2	0.133
ENSP00000221232	CNOT3	2	0.134
ENSP00000263026	EEF2K	171	0.134
ENSP00000328269	HMG20B	797	0.134
ENSP00000365402	HLA-C	1591	0.134
ENSP00000407964	NEDD1	1165	0.134
ENSP00000286657	ADAMTS3	1	0.135
ENSP00000301972	MYRIP	1599	0.135
ENSP00000255784	CCDC134	2	0.136
ENSP00000269468	MBD1	800	0.136
ENSP00000322016	PUF60	1213	0.136
ENSP00000363216	OGDHL	815	0.136
ENSP00000309913	TBX5	803	0.137
ENSP00000336740	LIMK1	2409	0.137
ENSP00000351327	AKAP4	4	0.137
ENSP00000355747	PSEN2	2007	0.137
ENSP00000388241	KIF26A	1202	0.137
ENSP00000229030	FZD10	800	0.138

ENSP00000298852	PSMC3	1207	0.138
ENSP00000351602	FUT4	3	0.138
ENSP00000365663	NPPA	3270	0.138
ENSP00000220809	PLAT	3184	0.139
ENSP00000275764	STRA8	1	0.139
ENSP00000231751	LTF	800	0.14
ENSP00000260766	PLCE1	39	0.14
ENSP00000305442	COG7	797	0.14
ENSP00000355140	HOXB1	795	0.14
ENSP00000361202	IRS4	1198	0.14
ENSP00000408395	RBFOX3	799	0.14
ENSP00000366843	ATXN2	1213	0.141
ENSP00000265734	CDK6	1208	0.142
ENSP00000300289	PDIA3	6835	0.142
ENSP00000317721	PIPOX	1	0.142
ENSP00000323516	UTS2R	800	0.142
ENSP00000326340	ATG16L2	1	0.142
ENSP00000364979	COL4A1	1588	0.142
ENSP00000366453	TJP2	834	0.142
ENSP00000414330	RIMKLA	1	0.142
ENSP00000238994	PPP1R3C	2	0.143
ENSP00000300161	YWHAB	3416	0.143

ENSP00000340237	SH3BP4	799	0.143
ENSP00000261799	PDGFRB	4294	0.144
ENSP00000291572	AGPAT3	1	0.144
ENSP00000314774	HES7	767	0.144
ENSP00000356737	GORAB	796	0.144
ENSP00000366620	H6PD	13	0.144
ENSP00000226299	LAP3	18	0.145
ENSP00000270223	DMWD	796	0.145
ENSP00000300574	CRK	9046	0.145
ENSP00000318374	LENG8	1206	0.145
ENSP00000329384	IL22	800	0.145
ENSP00000333496	KCND2	799	0.145
ENSP00000342626	EYA1	1	0.145
ENSP00000354927	MAP3K3	2010	0.145
ENSP00000366006	UBIAD1	1584	0.145
ENSP00000296161	DTX3L	1	0.146
ENSP00000341815	SOX18	799	0.146
ENSP00000361151	CEL	2008	0.146
ENSP00000361186	TP53RK	2007	0.146
ENSP00000252936	TUBGCP2	4	0.147
ENSP00000263205	MED15	1210	0.147
ENSP00000338562	STX3	800	0.147

ENSP00000384690	MMD2	1	0.147
ENSP00000338207	LMO1	1210	0.148
ENSP00000348965	DYNC1H1	1214	0.148
ENSP00000273308	CNPY2	800	0.149
ENSP00000290100	EPB41	823	0.149
ENSP00000352798	COL18A1	2342	0.149
ENSP00000400717	GNA13	2376	0.149
ENSP00000401018	GINS3	798	0.149
ENSP00000265517	MTTP	51	0.15
ENSP00000395535	MECP2	1595	0.15
ENSP00000244204	NAGK	8	0.151
ENSP00000262134	LPCAT2	1971	0.151
ENSP00000308236	COMMD1	1213	0.151
ENSP00000264193	CPOX	2	0.152
ENSP00000280326	CCT5	1596	0.152
ENSP00000288602	BRAF	2000	0.152
ENSP00000321674	4-Sep	800	0.152
ENSP00000206474	HAUS4	796	0.153
ENSP00000220584	FDFT1	3201	0.153
ENSP00000298198	PGM2L1	2	0.153
ENSP00000305892	TMEM208	1196	0.153
ENSP00000323858	DDX54	1204	0.153

ENSP00000380184	AGPAT6	808	0.153
ENSP00000268296	ITGAX	36	0.154
ENSP00000372023	CHEK2	817	0.154
ENSP00000372202	N6AMT2	2	0.154
ENSP00000225298	UTP18	4	0.155
ENSP00000256497	EDEM1	1177	0.155
ENSP00000334134	RGS9BP	1	0.155
ENSP00000258962	SRSF1	3307	0.156
ENSP00000334145	F3	800	0.156
ENSP00000335333	PIP5K1C	1201	0.156
ENSP00000297323	ADCY1	1	0.157
ENSP00000335074	GHRL	3346	0.158
ENSP00000342952	ADCY2	1145	0.158
ENSP00000221573	SNAPC2	1205	0.159
ENSP00000262305	RAB11FIP3	2413	0.159
ENSP00000295685	ARPC2	1213	0.159
ENSP00000367207	MYC	22687	0.159
ENSP00000368683	EDN1	3135	0.159
ENSP00000369889	COL2A1	2025	0.159
ENSP00000396774	MUC20	3	0.159
ENSP00000326366	PSEN1	10191	0.16
ENSP00000326819	FANCB	1192	0.16

ENSP00000337088	MEN1	11920	0.16
ENSP00000346032	ANXA2	2394	0.16
ENSP00000348762	LSS	793	0.16
ENSP00000374409	PKP4	800	0.16
ENSP00000181796	FAM107B	1600	0.161
ENSP00000227135	SPA17	799	0.161
ENSP00000263666	PDZRN3	1	0.161
ENSP00000343943	PPOX	2	0.161
ENSP00000353344	ETS2	785	0.161
ENSP00000375009	IGHV3-9	800	0.161
ENSP00000381412	CAMK2A	800	0.161
ENSP00000267859	BNIP2	798	0.162
ENSP00000348775	ACOX3	11	0.162
ENSP00000354782	CD247	1996	0.162
ENSP00000289004	HPD	799	0.164
ENSP00000300737	STIM1	1213	0.164
ENSP00000313681	SPHK1	3215	0.164
ENSP00000355050	CTNNBL1	1212	0.164
ENSP00000357731	LOR	1	0.164
ENSP00000359675	GNG5	3	0.164
ENSP00000409231	TRAPPC13	797	0.164
ENSP00000270458	CACNG8	644	0.165

ENSP00000305459	COG8	2	0.165
ENSP00000334050	TAS2R42	10	0.165
ENSP00000343204	JAK1	8537	0.165
ENSP00000344353	LPAR6	797	0.166
ENSP00000358867	GNAI3	33	0.166
ENSP00000377941	ACTN1	2006	0.166
ENSP00000378090	RAD51D	800	0.166
ENSP00000232375	PFKFB4	1	0.167
ENSP00000307078	KIF5B	1213	0.167
ENSP00000323580	IFT88	788	0.167
ENSP00000327758	NKX2-5	1593	0.167
ENSP00000343657	MCCC2	6	0.167
ENSP00000364839	ASXL1	148	0.167
ENSP00000265171	EGF	7954	0.168
ENSP00000282611	CATSPER3	1	0.168
ENSP00000352425	WASF1	290	0.168
ENSP00000356438	PTGS2	11127	0.168
ENSP00000356541	SF3B5	1208	0.168
ENSP00000216277	PAPOLA	799	0.169
ENSP00000233146	MSH2	4400	0.169
ENSP00000250448	FOXA1	2492	0.169
ENSP00000261636	ARL1	1209	0.169

ENSP00000362768	RBL1	756	0.169
ENSP00000370557	MIS12	899	0.169
ENSP00000380280	FGFR1	13324	0.169
ENSP00000250617	ARHGEF6	1204	0.17
ENSP00000273221	IQSEC1	796	0.17
ENSP00000295240	BBS5	9	0.17
ENSP00000346566	CKAP5	1214	0.17
ENSP00000358272	NDUFAF4	798	0.17
ENSP00000218388	TIMP1	1631	0.171
ENSP00000253571	RLIM	799	0.171
ENSP00000260682	CYP2C9	1	0.171
ENSP00000265641	CPT1A	1333	0.171
ENSP00000273550	FTH1	1	0.171
ENSP00000300935	RAB8A	2434	0.171
ENSP00000405934	ITPR1	4822	0.171
ENSP00000001008	FKBP4	2774	0.172
ENSP00000228841	MYL2	1211	0.172
ENSP00000276414	GNRH1	2750	0.172
ENSP00000316338	BAIAP2	872	0.172
ENSP00000360968	CYP4X1	797	0.172
ENSP00000010338	TRAF3IP3	1649	0.173
ENSP00000304350	PRPF8	1111	0.173

ENSP00000361405	MMP9	3294	0.173
ENSP00000311605	OR4B1	795	0.174
ENSP00000317128	PLXND1	1596	0.174
ENSP00000348982	MEGF6	2	0.174
ENSP00000257254	APLNR	70	0.175
ENSP00000307423	MGAT2	4	0.175
ENSP00000364802	HSPA1A	799	0.175
ENSP00000369756	PTPRA	795	0.175
ENSP00000200181	ITGB4	2011	0.177
ENSP00000338018	HIF1A	74473	0.177
ENSP00000230340	BYSL	804	0.178
ENSP00000316377	DLGAP1	3960	0.178
ENSP00000416753	MUC15	90	0.178
ENSP00000173229	NTN1	3856	0.179
ENSP00000257904	CDK4	7337	0.179
ENSP00000266544	NDUFA9	793	0.179
ENSP00000356791	DPT	4	0.179
ENSP00000184183	ROPN1	802	0.18
ENSP00000217244	CSNK2A1	5993	0.18
ENSP00000324806	GSK3B	16704	0.18
ENSP00000338191	SNTB2	1	0.18
ENSP00000346667	WNK3	1	0.18

ENSP00000367802	TAF1C	1213	0.18
ENSP00000233809	IGFBP2	799	0.181
ENSP00000293970	TBC1D24	1989	0.181
ENSP00000314414	AP2B1	4	0.181
ENSP00000324769	OR4C6	4	0.181
ENSP00000333982	NDEL1	3178	0.181
ENSP00000379310	CASC1	1587	0.181
ENSP00000222256	RAB3A	7163	0.183
ENSP00000261669	CAB39L	38	0.183
ENSP00000361859	TCEAL6	1	0.183
ENSP00000248553	HSPB1	3969	0.184
ENSP00000346879	NKX2-1	2401	0.184
ENSP00000261837	GNB5	1118	0.185
ENSP00000328181	NOG	1593	0.185
ENSP00000349052	OSCP1	689	0.185
ENSP00000368104	BMP2	9969	0.185
ENSP00000404179	DOCK4	796	0.185
ENSP00000405708	CCDC39	798	0.185
ENSP00000007708	PDK2	2142	0.186
ENSP00000242057	AHR	10228	0.186
ENSP00000246337	UROD	2	0.186
ENSP00000281453	MLF1IP	2396	0.186

ENSP00000303740	B3GALT1	2	0.186
ENSP00000361310	POLH	1213	0.186
ENSP00000260283	ARHGAP20	796	0.187
ENSP00000297268	COL1A2	2034	0.187
ENSP00000311344	PPP2R1B	493	0.187
ENSP00000363019	UBE2D1	1206	0.187
ENSP00000369695	MLLT3	800	0.187
ENSP00000228958	EIF2B1	1211	0.188
ENSP00000234590	ENO1	1208	0.188
ENSP00000244096	MAGEA10	1	0.188
ENSP00000256474	VHL	50066	0.188
ENSP00000278840	FADS2	5	0.188
ENSP00000303246	ACOT12	4	0.188
ENSP00000222005	CDC37	6007	0.189
ENSP00000199280	AQP2	802	0.19
ENSP00000252818	JUND	11920	0.19
ENSP00000265563	PRKAR2A	4780	0.191
ENSP00000281938	HSPB8	793	0.191
ENSP00000306330	YWHAG	1022	0.191
ENSP00000355961	INTS7	852	0.191
ENSP00000225831	CCL2	1692	0.192
ENSP00000239940	PFN2	9	0.192

ENSP00000308541	F2	10821	0.192
ENSP00000310244	RASGRP1	111	0.192
ENSP00000216540	SLC10A1	1210	0.193
ENSP00000309595	C10orf2	2	0.194
ENSP00000348827	THRB	65	0.194
ENSP00000349577	PRODH	4	0.194
ENSP00000356430	RGS18	2	0.194
ENSP00000410396	CMSS1	3	0.194
ENSP00000246533	CAPNS1	799	0.195
ENSP00000256196	RRAS2	800	0.195
ENSP00000314897	ANGPT2	1106	0.195
ENSP00000234160	GORASP2	1102	0.196
ENSP00000246891	CSN1S1	2806	0.196
ENSP00000264426	GRIA2	24	0.196
ENSP00000309629	CFL1	1224	0.196
ENSP00000316333	CD55	800	0.196
ENSP00000216341	GZMB	2007	0.197
ENSP00000317659	GUK1	2	0.197
ENSP00000313922	OAZ3	1195	0.198
ENSP00000329380	GP1BA	13990	0.198
ENSP00000390849	ABHD5	1622	0.198
ENSP00000216797	NFKBIA	2631	0.199

ENSP00000246071	SNRPB2	1203	0.199
ENSP00000295314	TMOD4	1	0.2
ENSP00000303212	SEMA3E	1596	0.2
ENSP00000220772	SFRP1	2	0.201
ENSP00000370381	SLC12A1	1	0.201
ENSP00000249373	SMO	1211	0.202
ENSP00000254854	GUCY2D	803	0.202
ENSP00000248846	TUBGCP6	4	0.203
ENSP00000223127	PLOD3	9	0.204
ENSP00000258123	USP15	1597	0.204
ENSP00000349298	MYLIP	800	0.204
ENSP00000371514	KCNV2	6	0.204
ENSP00000156109	GPKOW	1198	0.205
ENSP00000262105	MCM4	2986	0.205
ENSP00000262186	KCNH2	4123	0.205
ENSP00000350003	CCR3	1685	0.206
ENSP00000384573	DAZ1	6	0.206
ENSP00000387699	CREB1	2706	0.206
ENSP00000248594	PTPN12	799	0.207
ENSP00000318115	TIMM50	3	0.207
ENSP00000326630	ZFPM1	23	0.207
ENSP00000340330	KAT5	17104	0.207

ENSP00000358414	HMGCS2	647	0.207
ENSP00000366976	KRTAP1-4	1211	0.207
ENSP00000406335	CLIC1	799	0.207
ENSP00000035307	CHPF2	1207	0.208
ENSP00000054668	UTS2	791	0.208
ENSP00000249042	TST	4	0.208
ENSP00000301843	CTTN	1211	0.208
ENSP00000308782	GP6	66	0.208
ENSP00000344822	S100A13	26	0.209
ENSP00000385019	CACNA1I	797	0.209
ENSP00000301021	TRAPPC2L	1	0.21
ENSP00000312235	MUC13	2	0.21
ENSP00000346389	MEF2A	1210	0.21
ENSP00000377717	ST3GAL6	3	0.21
ENSP00000391490	AGR2	799	0.21
ENSP00000229135	IFNG	2425	0.211
ENSP00000268057	BBS4	8	0.211
ENSP00000296218	DNALI1	801	0.211
ENSP00000311827	MSL2	795	0.211
ENSP00000357708	S100A6	808	0.211
ENSP00000405574	TBL1XR1	2	0.211
ENSP00000381293	NSF	831	0.212

ENSP00000264634	WNT5A	798	0.213
ENSP00000298687	NDRG2	793	0.213
ENSP00000319060	CAMK2G	1920	0.213
ENSP00000358866	FLNA	3116	0.213
ENSP00000365441	CACNA1F	3	0.213
ENSP00000245907	C3	4003	0.214
ENSP00000312435	DAG1	8387	0.214
ENSP00000348234	TAT	800	0.214
ENSP00000231509	NR3C1	1133	0.215
ENSP00000263431	PRKCG	798	0.215
ENSP00000305255	STX8	12	0.215
ENSP00000338934	EZR	20145	0.215
ENSP00000342557	IL4I1	2	0.215
ENSP00000262375	DNAJA3	66	0.216
ENSP00000301200	CDC42EP5	800	0.216
ENSP00000366819	UCHL3	1189	0.216
ENSP00000391227	MGAT5B	1	0.216
ENSP00000216338	GZMH	798	0.217
ENSP00000348888	PIGR	801	0.217
ENSP00000272937	HES6	3	0.218
ENSP00000276689	NDUFB9	1209	0.218
ENSP00000287497	ITGAM	4404	0.218

ENSP00000340466	GANAB	822	0.218
ENSP00000294353	ZYG11B	1545	0.219
ENSP00000317714	STX4	6831	0.219
ENSP00000338862	BRIX1	1212	0.219
ENSP00000361554	TIE1	799	0.219
ENSP00000044462	PSMA4	3022	0.22
ENSP00000260810	TOPBP1	2003	0.22
ENSP00000290949	ATP6V0D1	1	0.22
ENSP00000340766	SMG7	1210	0.22
ENSP00000358242	PNISR	1213	0.22
ENSP00000267890	TTBK2	785	0.221
ENSP00000249005	A4GALT	1	0.222
ENSP00000256443	CDK7	4442	0.222
ENSP00000263360	EED	141	0.222
ENSP00000265708	ADAM2	800	0.222
ENSP00000291901	TNNT1	1590	0.222
ENSP00000331544	FBLN1	785	0.222
ENSP00000261205	SYT1	7931	0.223
ENSP00000298854	RAPSN	802	0.223
ENSP00000344468	SDC3	159	0.223
ENSP00000355370	CNTF	800	0.223
ENSP00000355596	DISC1	803	0.223

ENSP00000337736	AKAP1	5555	0.224
ENSP00000363021	RPA2	1213	0.224
ENSP00000220751	RIPK2	1213	0.225
ENSP00000236137	SLC19A2	4	0.225
ENSP00000261740	TRPV4	27	0.225
ENSP00000325423	INPP1	1	0.225
ENSP00000349708	ZMYM6	2	0.225
ENSP00000359518	MRGBP	1213	0.225
ENSP00000384515	PARVB	1204	0.225
ENSP00000395546	CSNK2B	799	0.225
ENSP00000397177	PBX2	800	0.225
ENSP00000301729	ECI1	2	0.226
ENSP00000312697	DMAP1	3175	0.226
ENSP00000257818	LMO2	2012	0.227
ENSP00000361759	BEX2	3	0.227
ENSP00000263645	CD81	789	0.228
ENSP00000316029	TLN1	1197	0.228
ENSP00000317337	CD300LB	1170	0.228
ENSP00000362071	JPH2	793	0.228
ENSP00000362555	RNF19B	3	0.228
ENSP00000369129	DSP	1594	0.228
ENSP00000295461	NIPAL1	1	0.229

ENSP00000332062	SNX20	1	0.229
ENSP00000343313	ATG5	1212	0.229
ENSP00000259407	BAAT	2	0.23
ENSP00000321239	RCHY1	796	0.23
ENSP00000260947	BARD1	5636	0.231
ENSP00000290597	ALDH4A1	1	0.231
ENSP00000326550	TACC3	1366	0.231
ENSP00000352561	CALCR	1	0.231
ENSP00000358105	APH1A	2006	0.231
ENSP00000358918	SUFU	1671	0.231
ENSP00000263710	CLASP1	4	0.232
ENSP00000275605	PSPH	800	0.232
ENSP00000353512	PARP9	1	0.232
ENSP00000308610	GPD2	1	0.233
ENSP00000339467	RHOG	782	0.233
ENSP00000356903	UAP1	2	0.233
ENSP00000357283	LMNA	2009	0.233
ENSP00000205948	APOH	28	0.235
ENSP00000305995	PGK2	3	0.235
ENSP00000356256	TIMM17A	2049	0.235
ENSP00000223642	C5	1603	0.236
ENSP00000359506	FMR1	1952	0.236

ENSP00000382697	ROCK1	26	0.236
ENSP00000242577	DYNLL1	10034	0.237
ENSP00000292303	CCR5	6513	0.237
ENSP00000384273	RELA	15494	0.237
ENSP00000401508	NAPRT1	1171	0.237
ENSP00000228850	AKAP3	3177	0.238
ENSP00000275428	GGCT	1	0.238
ENSP00000301050	CACNB3	18	0.238
ENSP00000407401	PEX5	1212	0.238
ENSP00000084795	RPL18	1198	0.239
ENSP00000343040	HMGB1	800	0.239
ENSP00000267101	ERBB3	5515	0.24
ENSP00000355325	PSMB5	4139	0.24
ENSP00000262133	RBL2	21	0.241
ENSP00000265175	SEC24B	1	0.241
ENSP00000352516	DNMT1	3456	0.241
ENSP00000053867	GRN	734	0.242
ENSP00000160262	ICAM3	800	0.242
ENSP00000285402	ODF1	800	0.242
ENSP00000351049	PAK4	2398	0.242
ENSP00000257068	MTNR1B	16	0.243
ENSP00000346901	FMO1	1	0.244

ENSP00000396622	MROH7	800	0.244
ENSP00000227752	IL10RA	2011	0.245
ENSP00000259631	CCL27	5	0.245
ENSP00000330284	NPBWR1	794	0.245
ENSP00000357775	UROS	2	0.245
ENSP00000359663	CD40LG	2010	0.245
ENSP00000370719	ITSN1	1212	0.245
ENSP00000256646	NOTCH2	2	0.246
ENSP00000289779	F11R	604	0.246
ENSP00000351125	ECHDC2	1	0.246
ENSP00000263036	OPTN	2266	0.248
ENSP00000307853	MUS81	800	0.248
ENSP00000308208	MMP14	800	0.248
ENSP00000348273	MBP	800	0.248
ENSP00000352257	XRCC6	2425	0.248
ENSP00000305464	APLN	11	0.249
ENSP00000370589	NOP56	787	0.249
ENSP00000314214	VAMP2	7592	0.25
ENSP00000345206	RBPJ	4814	0.25
ENSP00000373640	AGPHD1	1	0.25
ENSP00000196061	PLOD1	1201	0.251
ENSP00000306497	KCNJ4	32	0.251

ENSP00000331933	WSCD2	1	0.251
ENSP00000359297	NSDHL	1205	0.252
ENSP00000229022	VDR	7253	0.253
ENSP00000270142	SOD1	3605	0.253
ENSP00000275874	RAB19	800	0.253
ENSP00000248923	GGT1	13	0.254
ENSP00000279058	SPINT4	1	0.254
ENSP00000307280	MYL1	5	0.254
ENSP00000347379	OCLN	1622	0.254
ENSP00000373979	TRAPPC9	1	0.254
ENSP00000172229	NGFR	5257	0.255
ENSP00000242208	INHBA	21	0.255
ENSP00000264709	DNMT3A	15	0.255
ENSP00000251102	CNGB1	1213	0.256
ENSP00000312606	AKR1A1	1	0.256
ENSP00000334448	GNG2	3156	0.256
ENSP00000369325	CDKL5	795	0.256
ENSP00000217901	IDH3G	9	0.257
ENSP00000316042	HNRNPA0	1511	0.257
ENSP00000351905	TGFBR2	800	0.257
ENSP00000265727	ADAM22	799	0.258
ENSP00000287139	NODAL	799	0.258

ENSP00000301457	NDUFA7	10	0.258
ENSP00000231790	MLH1	2409	0.259
ENSP00000268864	RASL10B	793	0.259
ENSP00000292907	COX7A1	799	0.259
ENSP00000234296	ORC2	121	0.26
ENSP00000264741	ITGA9	800	0.26
ENSP00000285379	CA2	931	0.26
ENSP00000300055	PLIN1	1622	0.26
ENSP00000256857	GRP	5151	0.261
ENSP00000307235	EIF2AK3	4543	0.261
ENSP00000309262	USP48	3	0.261
ENSP00000331327	WT1	1213	0.261
ENSP00000349465	PICK1	2013	0.261
ENSP00000392858	TNF	799	0.261
ENSP00000286621	ADK	5	0.262
ENSP00000314441	METTL1	6	0.262
ENSP00000327077	PCM1	2	0.262
ENSP00000267085	CSAD	2	0.263
ENSP00000287078	TYSND1	1	0.263
ENSP00000351755	LPAR1	1	0.263
ENSP00000276033	SLC16A2	797	0.264
ENSP00000291554	CRYAA	1591	0.264

ENSP00000309591	PRKACA	15	0.264
ENSP00000261813	PFDN1	1213	0.265
ENSP00000251772	PLXNA1	800	0.267
ENSP00000265276	GPAM	719	0.267
ENSP00000315791	CSTF3	4425	0.267
ENSP00000405041	POU5F1	1305	0.267
ENSP00000219473	USP10	352	0.269
ENSP00000260356	THBS1	186	0.269
ENSP00000317327	UBASH3A	798	0.269
ENSP00000325120	PGR	799	0.269
ENSP00000348815	HYLS1	5	0.27
ENSP00000261769	CDH1	6817	0.271
ENSP00000351407	ARNT	9619	0.271
ENSP00000216392	PYGL	804	0.273
ENSP00000254942	TERF2	5402	0.273
ENSP00000264039	GPC1	8	0.273
ENSP00000353224	TFRC	1287	0.273
ENSP00000394794	PTPN13	100	0.273
ENSP00000229264	GNB3	797	0.274
ENSP00000229266	CHPT1	765	0.274
ENSP00000264834	KLF1	799	0.274
ENSP00000296440	PLXNB1	2387	0.274

ENSP00000351155	ATL1	797	0.274
ENSP00000236192	VAMP4	1212	0.275
ENSP00000365891	WAS	6294	0.275
ENSP00000369099	ADPRM	1	0.275
ENSP00000252675	FUT5	1	0.276
ENSP00000413596	PPP1R18	795	0.276
ENSP00000260795	FGFR3	1597	0.277
ENSP00000261819	ANAPC5	57	0.277
ENSP00000328062	KIAA1033	2	0.277
ENSP00000347197	C5AR1	7	0.277
ENSP00000354929	NOTCH2NL	1600	0.277
ENSP00000284818	LY96	1584	0.278
ENSP00000313007	PABPC1	3186	0.278
ENSP00000338868	PHF8	2003	0.278
ENSP00000262646	RAB2A	2	0.279
ENSP00000320940	NCOA1	15004	0.279
ENSP00000318351	BCKDHB	735	0.28
ENSP00000307781	ODF2	800	0.281
ENSP00000328236	KNTC1	3	0.282
ENSP00000249760	IVD	2	0.283
ENSP00000262854	HUWE1	1138	0.283
ENSP00000264409	AGPAT9	7	0.283

ENSP00000338297	IGF2	2172	0.283
ENSP00000346827	ALDH9A1	1	0.283
ENSP00000353731	DPP4	1233	0.283
ENSP00000271139	MOB3C	4	0.284
ENSP00000357348	HEY2	798	0.285
ENSP00000271657	PI4KB	2	0.288
ENSP00000318445	ST3GAL1	801	0.288
ENSP00000381657	DOT1L	1	0.288
ENSP00000252034	ELN	1220	0.289
ENSP00000262160	SMAD2	13236	0.289
ENSP00000312122	SEC13	3220	0.289
ENSP00000313391	DAB2	1143	0.289
ENSP00000343274	INTS8	3068	0.289
ENSP00000356771	F5	800	0.289
ENSP00000261167	WBP11	2	0.29
ENSP00000262367	CREBBP	37688	0.29
ENSP00000269397	CBX4	4427	0.29
ENSP00000337127	SOD2	2395	0.29
ENSP00000287713	NMNAT2	4	0.292
ENSP00000324549	CYFIP1	1952	0.292
ENSP00000364133	TGFBR1	14003	0.292
ENSP00000254322	DNAJB1	1187	0.293

ENSP00000346294	S100A4	1602	0.293
ENSP00000354901	CXCL9	1959	0.293
ENSP00000278715	HMBS	2	0.294
ENSP00000290974	ZFYVE28	1205	0.294
ENSP00000301788	POLR2G	1203	0.294
ENSP00000228280	KITLG	799	0.295
ENSP00000328708	RXFP3	5	0.296
ENSP00000355536	MTR	1214	0.296
ENSP00000278916	CHEK1	2191	0.297
ENSP00000310771	GRB7	738	0.297
ENSP00000217893	TAF9	5	0.298
ENSP00000337053	SEL1L	1177	0.298
ENSP00000415183	MUC2	1595	0.298
ENSP00000272298	CALM2	10	0.299
ENSP00000283006	CENPH	799	0.299
ENSP00000359539	GOT1	9	0.299
ENSP00000360798	EPS15	6583	0.299
ENSP00000272348	SNRPG	800	0.3
ENSP00000298937	ELP4	799	0.301
ENSP00000363435	ITPR3	96	0.301
ENSP00000375809	ERCC2	1213	0.301
ENSP00000217109	CSTF1	4424	0.302

ENSP00000262839	TRPC5	803	0.302
ENSP00000287647	FANCD2	3803	0.302
ENSP00000352138	KIRREL	4496	0.302
ENSP00000364649	SDHB	2423	0.302
ENSP00000382819	DOM3Z	788	0.302
ENSP00000356587	NPHS2	5282	0.304
ENSP00000361878	CAP1	20	0.304
ENSP00000367124	SLC3A2	803	0.304
ENSP00000340691	EIF4EBP1	5095	0.305
ENSP00000406037	KAT8	2007	0.306
ENSP00000293275	CCL16	3	0.307
ENSP00000333194	RGS19	337	0.307
ENSP00000354251	NCKAP1	2242	0.307
ENSP00000371341	TNK2	795	0.307
ENSP00000267415	TINF2	2343	0.308
ENSP00000331817	ALYREF	1212	0.308
ENSP00000343392	XRCC3	19	0.308
ENSP00000368754	BAZ2A	799	0.308
ENSP00000355607	GNPAT	1	0.309
ENSP00000262519	SETD1A	1210	0.31
ENSP00000279230	PLCB3	5	0.31
ENSP00000350009	USP33	36	0.31

ENSP00000313936	OR2AE1	10	0.311
ENSP00000351926	AP2A1	2422	0.313
ENSP00000366488	PRKACG	21	0.313
ENSP00000229794	MAPK14	8377	0.314
ENSP00000312262	ADRBK1	1870	0.314
ENSP00000269300	PIK3R5	3006	0.315
ENSP00000279387	PPP4C	800	0.315
ENSP00000371138	FKBP1A	3235	0.315
ENSP00000386896	ITGA6	2097	0.315
ENSP00000247161	ELK1	177	0.316
ENSP00000288135	KIT	2008	0.316
ENSP00000367830	PRKCZ	2005	0.316
ENSP00000296181	ITGB5	7	0.317
ENSP00000332592	SPAG16	1196	0.317
ENSP00000231449	IL4	1015	0.318
ENSP00000351896	TRAPPC4	2803	0.318
ENSP00000356713	IFNGR1	2421	0.319
ENSP00000264972	ZAP70	1864	0.32
ENSP00000304236	CD14	13	0.321
ENSP00000349252	ITGAL	800	0.321
ENSP00000280346	DLAT	808	0.322
ENSP00000379836	SLC30A5	1	0.322

ENSP00000006275	TRAPPC6A	2005	0.323
ENSP00000052754	DCN	6749	0.323
ENSP00000263093	SLC27A5	1	0.323
ENSP00000290039	CACHD1	8	0.323
ENSP00000355587	NTPCR	793	0.323
ENSP00000363384	ZNF593	4	0.323
ENSP00000419361	ADCY5	12	0.323
ENSP00000258091	CCT7	5	0.324
ENSP00000262554	SPTLC1	1207	0.324
ENSP00000302234	CCL11	1224	0.324
ENSP00000316032	NUP98	3219	0.324
ENSP00000227667	APOC3	12	0.325
ENSP00000342082	SLPI	734	0.325
ENSP00000323479	B3GALNT1	3	0.326
ENSP00000357858	BUB3	19358	0.327
ENSP00000365775	MTHFR	1205	0.327
ENSP00000376204	ASH1L	1	0.327
ENSP00000263464	BIRC3	1205	0.328
ENSP00000299299	PCBD1	796	0.328
ENSP00000339109	ANAPC1	10622	0.328
ENSP00000296871	CSF2	2084	0.329
ENSP00000314151	KLK3	799	0.329

ENSP00000361818	SDC4	798	0.329
ENSP00000373614	SELPLG	2010	0.329
ENSP00000299626	ALG8	4	0.33
ENSP00000318016	KIAA0196	1	0.33
ENSP00000295266	PDHA2	4	0.331
ENSP00000256720	LPIN1	799	0.332
ENSP00000268261	PMM2	9	0.333
ENSP00000369855	ASB9	1211	0.333
ENSP00000205402	DLD	3272	0.334
ENSP00000358323	TXNIP	1206	0.334
ENSP00000367910	FANCG	2450	0.334
ENSP00000003100	CYP51A1	1	0.335
ENSP00000315325	ARHGEF2	786	0.335
ENSP00000315615	AKAP5	16	0.335
ENSP00000303864	OR8I2	13	0.336
ENSP00000304188	OR8U1	7	0.336
ENSP00000228682	GLI1	1572	0.337
ENSP00000269280	NLRP1	792	0.337
ENSP00000299543	CTDP1	4775	0.337
ENSP00000339861	ENY2	793	0.337
ENSP00000358043	ECM1	1	0.337
ENSP00000371813	DEFB132	1	0.337

ENSP00000254584	ARFIP2	1209	0.338
ENSP00000204961	EFNB1	577	0.339
ENSP00000226359	AFP	18	0.339
ENSP00000286317	MED7	14	0.339
ENSP00000356579	CEP350	799	0.339
ENSP00000369643	GRPR	76	0.339
ENSP00000263354	NAPA	829	0.34
ENSP00000360181	SH2D1A	800	0.34
ENSP00000361892	STK4	1213	0.34
ENSP00000255266	PDE6A	3	0.341
ENSP00000365492	DEFB119	2	0.342
ENSP00000262584	RPL8	1042	0.345
ENSP00000417404	HFE	1997	0.345
ENSP00000274353	BHMT	1	0.346
ENSP00000363708	BMPR2	1603	0.346
ENSP00000345708	KCNJ11	2	0.347
ENSP00000358022	MCL1	9856	0.348
ENSP00000390722	SLC25A17	1	0.348
ENSP00000219070	MMP2	3932	0.349
ENSP00000261507	MSMO1	1206	0.349
ENSP00000290200	IL10RB	800	0.349
ENSP00000328777	EFNA5	1211	0.349

ENSP00000346921	AK2	1	0.349
ENSP00000367462	OLAH	789	0.35
ENSP00000346155	UCKL1	2	0.351
ENSP00000345530	NEDD4	1206	0.352
ENSP00000358223	PNLIP	4	0.352
ENSP00000263642	IFIH1	798	0.353
ENSP00000272521	TMEM177	12	0.353
ENSP00000358421	HSD3B1	58	0.353
ENSP00000413625	FNBP1	1132	0.353
ENSP00000349320	CACNA2D1	1	0.354
ENSP00000221972	CD79A	1995	0.355
ENSP00000365682	TLE1	1777	0.355
ENSP00000371169	RCL1	1	0.355
ENSP00000384015	SUN1	43	0.356
ENSP00000277575	USP6NL	787	0.357
ENSP00000296875	GDF9	1	0.357
ENSP00000360154	OCRL	1212	0.357
ENSP00000360882	COL5A1	5	0.357
ENSP00000311005	MAPK7	1	0.358
ENSP00000378338	GIT1	1801	0.358
ENSP00000179259	C12orf5	1983	0.36
ENSP00000264637	THRA	153	0.36

ENSP00000374372	SPTB	803	0.36
ENSP00000221930	TGFB1	10585	0.361
ENSP00000263038	PHYH	768	0.361
ENSP00000305689	AFF1	799	0.361
ENSP00000222390	HGF	800	0.362
ENSP00000263233	SYP	796	0.362
ENSP00000360316	DHCR24	805	0.362
ENSP00000309503	YWHAZ	14315	0.364
ENSP00000402065	POLR2J2	1	0.364
ENSP00000419692	RXRA	5109	0.364
ENSP00000219548	STUB1	8298	0.365
ENSP00000272167	EPHX1	754	0.365
ENSP00000265333	VDAC1	2399	0.366
ENSP00000344666	NF2	1	0.366
ENSP00000410076	CASP1	3185	0.366
ENSP00000323076	NDUFAF3	798	0.367
ENSP00000315859	RNPS1	3772	0.368
ENSP00000381331	HDAC2	6006	0.368
ENSP00000264998	TF	1174	0.369
ENSP00000356070	MAPKAPK2	4529	0.369
ENSP00000368464	KIF24	1	0.369
ENSP00000308012	PABPC5	1	0.37

ENSP00000309845	HRAS	11676	0.37
ENSP00000311469	GSTM1	2137	0.37
ENSP00000299518	IDH3A	15	0.371
ENSP00000363641	TXN	2801	0.371
ENSP00000215909	LGALS1	796	0.372
ENSP00000270474	PDE4A	1631	0.372
ENSP00000355541	HEATR1	1	0.372
ENSP00000400842	HLA-B	9	0.372
ENSP00000302177	MBOAT2	11	0.373
ENSP00000264018	DDX6	800	0.374
ENSP00000338345	SNCA	3177	0.375
ENSP00000418447	PPP2CA	17081	0.375
ENSP00000333212	CYP2U1	2	0.376
ENSP00000391681	POU5F1	784	0.376
ENSP00000411672	ATP6V0E2	155	0.376
ENSP00000227495	ST3GAL4	1	0.377
ENSP00000244289	LIPE	1687	0.377
ENSP00000308268	CARNS1	2	0.377
ENSP00000309166	RBM4	182	0.377
ENSP00000318128	BLOC1S4	3	0.377
ENSP00000366347	NKX2-2	789	0.377
ENSP00000373106	RNF39	2	0.377

ENSP00000263281	GIPR	6	0.378
ENSP00000312244	MSL3	795	0.378
ENSP00000259512	DERL1	2000	0.379
ENSP00000329357	SP1	11257	0.379
ENSP00000339917	TAF9B	784	0.379
ENSP00000363868	ABCA1	2444	0.379
ENSP00000268695	GALNS	4	0.38
ENSP00000283195	RANBP2	726	0.38
ENSP00000261024	SLC40A1	4	0.381
ENSP00000305480	FEN1	1213	0.381
ENSP00000362131	RPA4	783	0.381
ENSP00000265354	SRF	4796	0.382
ENSP00000313420	PRKDC	8715	0.382
ENSP00000338173	PUM2	1	0.382
ENSP00000341032	WNT7B	800	0.382
ENSP00000301012	MVD	1	0.383
ENSP00000233114	MDH1	778	0.384
ENSP00000366565	VPS28	2426	0.384
ENSP00000357535	ECHS1	17	0.385
ENSP00000356918	STX7	2836	0.386
ENSP00000366927	ALDH1B1	1	0.386
ENSP00000302564	BCL2L1	3394	0.387

ENSP00000308179	WDR3	7	0.387
ENSP00000278568	PAK1	3855	0.388
ENSP00000284981	APP	13015	0.388
ENSP00000262320	AXIN1	19608	0.389
ENSP00000296145	TDGF1	796	0.39
ENSP00000354522	TOP1	786	0.39
ENSP00000313752	SSNA1	797	0.391
ENSP00000211998	VCL	1665	0.392
ENSP00000283635	CD8A	654	0.392
ENSP00000363827	HSPG2	18	0.392
ENSP00000317992	NOC2L	1208	0.393
ENSP00000372860	VARS2	618	0.393
ENSP00000225396	TADA2A	801	0.394
ENSP00000360286	RAE1	4011	0.396
ENSP00000363763	EPHB2	445	0.396
ENSP00000368119	GALT	1213	0.396
ENSP00000252486	APOE	402	0.397
ENSP00000352121	PIK3CG	3004	0.397
ENSP00000303315	JUNB	45	0.398
ENSP00000410007	FBXO46	797	0.398
ENSP00000263073	SMG6	2	0.401
ENSP00000337014	HFE2	70	0.401

ENSP00000370748	IDI1	1	0.401
ENSP00000310551	LCLAT1	9	0.402
ENSP00000380349	CAPN3	799	0.402
ENSP00000408910	DCTN2	7	0.402
ENSP00000256010	NTS	1005	0.404
ENSP00000272902	SUMF1	4	0.404
ENSP00000296154	CASR	775	0.404
ENSP00000361021	PTEN	2859	0.404
ENSP00000258654	COG3	796	0.406
ENSP00000282588	ITGA1	34	0.406
ENSP00000311360	RAD9A	1222	0.406
ENSP00000320885	SPAST	797	0.406
ENSP00000361636	TNNC2	10	0.406
ENSP00000263918	STRN	81	0.407
ENSP00000276062	NDUFB11	53	0.407
ENSP00000304811	UGT2B7	8	0.407
ENSP00000364519	TFDP1	1209	0.407
ENSP00000227163	SPI1	800	0.408
ENSP00000299402	APBB1	8325	0.408
ENSP00000339328	PLAUR	2809	0.408
ENSP00000325690	CARM1	22	0.409
ENSP00000326948	FTSJ1	2	0.409

ENSP00000340698	GIPC1	1458	0.409
ENSP00000310040	EIF3F	1090	0.41
ENSP00000254351	SDC1	163	0.411
ENSP00000263864	VAMP8	2829	0.411
ENSP00000360502	PDE6C	3	0.411
ENSP00000362463	GLO1	1	0.411
ENSP00000234313	PLEK	798	0.412
ENSP00000261741	RBM19	1	0.412
ENSP00000263182	BBOX1	799	0.412
ENSP00000391901	PHF1	2173	0.412
ENSP00000259895	GTF2H4	798	0.415
ENSP00000334008	PARVA	799	0.415
ENSP00000356520	DHX9	96	0.415
ENSP00000369055	B4GALT1	1591	0.415
ENSP00000340361	ACVR2B	3	0.418
ENSP00000046794	LCP2	2797	0.419
ENSP00000221265	PAF1	1211	0.42
ENSP00000297151	SLU7	3725	0.42
ENSP00000372326	FECH	47	0.42
ENSP00000386284	ALAD	721	0.42
ENSP00000296755	MAP1B	253	0.421
ENSP00000237014	TTR	2397	0.422

ENSP00000262052	SLC11A2	15	0.422
ENSP00000265174	PAPSS1	5	0.422
ENSP00000368190	NPHS1	58	0.422
ENSP00000243253	SEC61A1	2	0.423
ENSP00000357301	RXFP4	776	0.423
ENSP00000293872	LUC7L	3	0.424
ENSP00000321706	GEMIN4	1	0.424
ENSP00000337915	CYP3A4	832	0.424
ENSP00000359401	C1orf146	2	0.424
ENSP00000414982	KLC1	1217	0.424
ENSP00000338548	FGF1	1	0.425
ENSP00000384665	LPAR2	1776	0.426
ENSP00000387123	ALDH7A1	5	0.427
ENSP00000249344	STRIP2	5	0.428
ENSP00000359998	GSTA4	3	0.428
ENSP00000343418	SEMA4D	771	0.429
ENSP00000361359	CD40	2007	0.429
ENSP00000264775	PPAP2A	5	0.43
ENSP00000230792	NUDT12	1	0.431
ENSP00000361066	NCOA3	817	0.433
ENSP00000383263	CCHCR1	2	0.433
ENSP00000270538	TIMM44	2049	0.434

ENSP00000348554	CDC16	1342	0.435
ENSP00000381717	UBE2D2	1192	0.435
ENSP00000405635	CNEP1R1	3	0.435
ENSP00000323967	SMARCE1	1	0.436
ENSP00000320949	CNOT1	396	0.437
ENSP00000224337	BLNK	1976	0.438
ENSP00000306614	PPIH	622	0.439
ENSP00000341551	SMAD4	10036	0.439
ENSP00000219476	TSC2	9396	0.44
ENSP00000265094	FBXW11	198	0.44
ENSP00000252487	TOMM40	1574	0.441
ENSP00000252674	MLLT1	799	0.441
ENSP00000405965	SUMO2	1213	0.442
ENSP00000262735	PPARA	4231	0.443
ENSP00000264832	ICAM1	66	0.443
ENSP00000255764	MED10	1212	0.444
ENSP00000363081	DKK1	796	0.444
ENSP00000157812	PSMC4	3080	0.445
ENSP00000223095	SERPINE1	4542	0.445
ENSP00000253401	ARHGEF9	7	0.447
ENSP00000350249	POT1	795	0.447
ENSP00000235090	WDR77	17491	0.448

ENSP00000241052	CAT	1596	0.448
ENSP00000301071	TUBA1A	1206	0.448
ENSP00000388107	UBA52	1213	0.448
ENSP00000251337	GNAT2	808	0.449
ENSP00000293330	HCRT	1212	0.449
ENSP00000261708	UTP6	2	0.45
ENSP00000298296	MAGEC3	1	0.452
ENSP00000226730	IL2	2054	0.453
ENSP00000316605	CNGB3	12	0.453
ENSP00000334100	EXOC7	1234	0.453
ENSP00000417764	ALG2	2801	0.453
ENSP00000347184	HTT	2395	0.454
ENSP00000360761	AGPAT2	2	0.454
ENSP00000392466	LDB1	799	0.454
ENSP00000260372	HAUS2	2	0.455
ENSP00000265335	RAD50	5057	0.455
ENSP00000309092	GMPPB	4	0.455
ENSP00000261531	SNW1	1222	0.458
ENSP00000354791	DCTN1	2797	0.458
ENSP00000355629	COG2	796	0.458
ENSP00000368914	PSTPIP1	1212	0.458
ENSP00000296223	POLR2H	690	0.459

ENSP00000280704	LDHC	12	0.46
ENSP00000414258	TRIM40	1	0.46
ENSP00000331358	GAST	2053	0.461
ENSP00000286301	CSF1R	1579	0.462
ENSP00000312017	FAM57A	12	0.463
ENSP00000356991	PVRL4	792	0.463
ENSP00000379110	CXCL1	1988	0.463
ENSP00000229769	FANCE	2004	0.464
ENSP00000243349	ACVR1C	792	0.464
ENSP00000267996	TPM1	3990	0.464
ENSP00000358997	IRAK1	8714	0.464
ENSP00000321732	CTDNEP1	3	0.465
ENSP00000350199	AP1B1	3	0.465
ENSP00000361219	GTF3C4	1213	0.465
ENSP00000296792	UTP15	1	0.466
ENSP00000324173	HSPA5	1212	0.466
ENSP00000360515	SUPT3H	1180	0.467
ENSP00000406157	PAPSS2	3	0.467
ENSP00000317904	GYS1	5115	0.468
ENSP00000265594	MCCC1	1	0.47
ENSP00000371152	ASAH1	2	0.47
ENSP00000217961	STS	811	0.472

ENSP00000261900	CCNT1	844	0.473
ENSP00000309259	ALAS1	719	0.473
ENSP00000318318	MPI	7	0.473
ENSP00000361965	ADA	26	0.473
ENSP00000367615	APRT	12	0.474
ENSP00000336747	HIP1	15	0.475
ENSP00000257430	APC	1204	0.476
ENSP00000300692	CD3D	1	0.476
ENSP00000363779	IKBKAP	2	0.476
ENSP00000236051	EBNA1BP2	2	0.477
ENSP00000263409	LIFR	800	0.477
ENSP00000290663	MED8	1212	0.477
ENSP00000341268	TRADD	2	0.477
ENSP00000380150	CARD11	2	0.478
ENSP00000276571	CRH	917	0.48
ENSP00000302021	MUC7	2	0.48
ENSP00000231572	RARS	802	0.482
ENSP00000262158	SMAD7	8209	0.484
ENSP00000265371	NRP1	2393	0.484
ENSP00000340409	SMPD1	1202	0.485
ENSP00000301141	CYP2A6	8	0.487
ENSP00000347329	SEC31A	1	0.488

ENSP00000391069	SRPK1	436	0.488
ENSP00000254719	RPA1	1935	0.49
ENSP00000339353	CPSF1	2011	0.49
ENSP00000283875	GTF2E1	24	0.491
ENSP00000324729	SAV1	199	0.491
ENSP00000334061	HDAC6	1206	0.491
ENSP00000233946	IL1R1	1600	0.492
ENSP00000369497	BRCA2	3412	0.492
ENSP00000353452	MYLK	5982	0.494
ENSP00000219789	CDIPT	2	0.495
ENSP00000307567	QARS	2	0.495
ENSP00000343144	PARD6G	793	0.495
ENSP00000381098	GRIP1	1211	0.496
ENSP00000321259	TALDO1	800	0.498
ENSP00000353154	NFASC	807	0.498
ENSP00000294304	LRP5	798	0.499
ENSP00000305725	CHST11	2	0.499
ENSP00000372793	LTA	1317	0.499
ENSP00000257192	DSG1	790	0.5
ENSP00000366395	SYVN1	1177	0.5
ENSP00000391349	DOM3Z	1762	0.5
ENSP00000245479	SOX9	1594	0.501

ENSP00000324422	ZYX	2	0.501
ENSP00000352980	HIST1H4A	5277	0.501
ENSP00000350512	COPS5	7769	0.502
ENSP00000290649	AMFR	1992	0.503
ENSP00000355865	PARK2	797	0.503
ENSP00000271620	PRUNE	11	0.504
ENSP00000311113	JUP	2436	0.505
ENSP00000338964	GGT7	15	0.505
ENSP00000353804	ACSS2	1	0.505
ENSP00000383059	ARGLU1	432	0.505
ENSP00000260010	TLR2	812	0.506
ENSP00000262768	TIMP2	799	0.506
ENSP00000354960	COLGALT2	2	0.506
ENSP00000370151	RAD17	301	0.506
ENSP00000216442	ATP6V1D	1150	0.507
ENSP00000286398	SMC2	789	0.507
ENSP00000291700	S100B	2806	0.507
ENSP00000354560	KIFAP3	2	0.507
ENSP00000355812	FGFR1OP	799	0.509
ENSP00000304669	CTNNA1	2050	0.51
ENSP00000340684	MAOA	2	0.511
ENSP00000216456	VTI1B	793	0.512

ENSP00000291688	MCM3AP	793	0.512
ENSP00000347719	TBCD	5	0.512
ENSP00000351273	CASP8	1229	0.512
ENSP00000261207	PPP1R12A	792	0.513
ENSP00000349547	RASSF1	1013	0.514
ENSP00000261366	LMNB1	2050	0.515
ENSP00000245960	CDC25B	1899	0.517
ENSP00000318585	BACE1	1213	0.517
ENSP00000288266	APPL1	799	0.518
ENSP00000333275	NR2C1	17	0.52
ENSP00000338983	MUC1	10019	0.52
ENSP00000344936	PTTG1	1981	0.521
ENSP00000348551	NCOR2	2023	0.521
ENSP00000354826	CALD1	3989	0.521
ENSP00000332643	NDN	548	0.522
ENSP00000223114	MOGAT3	3	0.523
ENSP00000316176	UBE2N	2004	0.523
ENSP00000351410	PRKAR1A	7	0.523
ENSP00000212015	SIRT1	3121	0.524
ENSP00000331815	TBL3	1	0.524
ENSP00000257555	HNF1A	796	0.525
ENSP00000263168	CAPZA1	1210	0.525

ENSP00000264914	ARSB	794	0.525
ENSP00000334458	GATA4	1598	0.525
ENSP00000420295	PDE6B	4	0.525
ENSP00000266376	CACNA1C	815	0.527
ENSP00000303242	ITGB2	5166	0.527
ENSP00000242592	ACADS	5	0.53
ENSP00000262013	SPAG9	1217	0.53
ENSP00000365851	BMI1	2423	0.532
ENSP00000366410	NMNAT1	1171	0.533
ENSP00000298139	WRN	339	0.534
ENSP00000394400	SKIV2L	2	0.534
ENSP00000290299	ATP5O	1	0.535
ENSP00000356425	UCHL5	2645	0.535
ENSP00000352264	CD2AP	5222	0.536
ENSP00000327583	RANBP1	9	0.537
ENSP00000307241	PDHB	8	0.539
ENSP00000320171	PKM	1202	0.541
ENSP00000361850	PLAU	1200	0.541
ENSP00000307863	U2AF2	24145	0.542
ENSP00000354621	SMURF1	1584	0.542
ENSP00000257181	PRPF38A	1754	0.543
ENSP00000330032	UPP1	1	0.543

ENSP00000382177	MYO5A	41	0.543
ENSP00000293288	BAX	1992	0.544
ENSP00000320180	GHRHR	19	0.544
ENSP00000223129	RPA3	830	0.545
ENSP00000232014	BCL6	813	0.545
ENSP00000241416	ACVR2A	8	0.545
ENSP00000220764	DECR1	1183	0.548
ENSP00000311677	PPP1R8	5595	0.548
ENSP00000346001	RPL3	5	0.549
ENSP00000379204	BMP7	813	0.549
ENSP00000401397	DDR1	1	0.549
ENSP00000221233	EXOSC5	1208	0.551
ENSP00000317331	SSR4	1	0.551
ENSP00000254480	SMARCC1	1913	0.554
ENSP00000307786	CYCS	800	0.554
ENSP00000260762	EXOC6	149	0.555
ENSP00000286548	GNAQ	4	0.555
ENSP00000360035	PPP1R3D	1	0.555
ENSP00000256216	HSD17B4	8	0.556
ENSP00000347443	RASSF5	1	0.556
ENSP00000317039	RMI1	21	0.557
ENSP00000359727	BAG2	4194	0.558

ENSP00000251968	TSG101	10025	0.559
ENSP00000377262	SRPK2	432	0.561
ENSP00000296785	ANKRA2	16	0.562
ENSP00000360149	ALG6	7	0.562
ENSP00000252575	NCAN	2	0.563
ENSP00000314508	GBA	2	0.563
ENSP00000349049	KDM1A	822	0.563
ENSP00000226218	SEBOX	451	0.564
ENSP00000259633	CD72	455	0.564
ENSP00000315442	NR1H4	815	0.564
ENSP00000005257	RALA	2163	0.565
ENSP00000311449	RAB6A	1204	0.565
ENSP00000210313	PSMD5	4	0.568
ENSP00000215375	ATP5D	9	0.568
ENSP00000305958	STIP1	796	0.568
ENSP00000337761	RAB27A	1616	0.568
ENSP00000343745	DICER1	1271	0.568
ENSP00000228027	DGAT2	2	0.569
ENSP00000374357	ARNTL	1	0.569
ENSP00000384053	CSF2RB	2084	0.569
ENSP00000237527	GHRH	19	0.571
ENSP00000240316	COIL	1	0.571

ENSP00000345571	E2F1	5203	0.571
ENSP00000262887	XRCC1	1604	0.573
ENSP00000299424	TAF10	816	0.574
ENSP00000264663	NNT	2	0.575
ENSP00000350616	DDC	2344	0.577
ENSP00000358635	SYNCRIP	3	0.577
ENSP00000238738	RHOQ	4	0.578
ENSP00000358554	BCAS2	407	0.58
ENSP00000335304	DLST	827	0.581
ENSP00000350275	HIST1H3A	3346	0.581
ENSP00000267430	FANCM	978	0.583
ENSP00000356248	PTPN7	1958	0.583
ENSP00000381607	GSTP1	1247	0.583
ENSP00000356087	IKBKE	1223	0.584
ENSP00000401445	ERN1	1139	0.584
ENSP00000363313	PRPF4	133	0.585
ENSP00000399968	NCOA2	12	0.585
ENSP00000276390	ATP6V1B2	3	0.586
ENSP00000358617	PHTF1	6	0.587
ENSP00000354876	MT-CO2	2800	0.588
ENSP00000364893	ARHGEF7	1203	0.588
ENSP00000323929	A2M	7	0.59

ENSP00000344579	FSCB	782	0.59
ENSP00000396308	DHFR	1209	0.591
ENSP00000346236	DDX46	432	0.592
ENSP00000302620	AGXT	1205	0.593
ENSP00000367934	UQCRQ	10	0.594
ENSP00000019317	RALBP1	1	0.595
ENSP00000374280	RTF1	797	0.595
ENSP00000303532	DEFB4A	800	0.596
ENSP00000339992	MYB	3728	0.596
ENSP00000319501	UGDH	19	0.597
ENSP00000318297	RUVBL1	3316	0.598
ENSP00000230354	TBP	11722	0.6
ENSP00000345774	ALDH3A2	13	0.601
ENSP00000265564	EXOSC7	41	0.603
ENSP00000318861	SF3B2	776	0.604
ENSP00000261461	PPP2R5A	1870	0.605
ENSP00000297579	DCAF13	1	0.606
ENSP00000365439	HNRNPK	32826	0.607
ENSP00000257770	NT5E	20	0.608
ENSP00000261755	FAH	797	0.608
ENSP00000396219	MEF2C	816	0.61
ENSP00000284384	PRKCA	3794	0.614

ENSP00000364709	F10	800	0.614
ENSP00000229307	NANOG	293	0.615
ENSP00000358727	GSTO1	1	0.615
ENSP00000263033	SYTL4	1586	0.617
ENSP00000263331	POLR1B	1200	0.618
ENSP00000308741	CLOCK	778	0.62
ENSP00000349142	ATP5C1	1	0.62
ENSP00000301364	TSR1	4	0.621
ENSP00000351908	MAP3K5	4933	0.621
ENSP00000360125	PGM1	17	0.621
ENSP00000261416	HEXB	5	0.622
ENSP00000333769	BSG	801	0.624
ENSP00000363921	PARD3	2195	0.624
ENSP00000380252	NFE2L2	506	0.624
ENSP00000299293	FRS2	12	0.625
ENSP00000338799	IL6ST	2059	0.625
ENSP00000237530	RPN2	1	0.626
ENSP00000300738	RRM1	329	0.626
ENSP00000204726	GOLGA3	1	0.627
ENSP00000245932	VASP	1226	0.627
ENSP00000261023	ITGAV	1293	0.628
ENSP00000263341	IL1B	2	0.631

ENSP00000312288	HADH	15	0.631
ENSP00000261479	PSMA6	159	0.632
ENSP00000354673	CNOT4	395	0.632
ENSP00000297518	CDK5	5395	0.633
ENSP00000222304	HAMP	4	0.635
ENSP00000341538	SEC61G	2423	0.636
ENSP00000361047	ALG13	3	0.637
ENSP00000361707	TCEAL1	2	0.637
ENSP00000221494	SF3A2	23998	0.638
ENSP00000303191	PLRG1	7	0.638
ENSP00000303507	BCR	111	0.638
ENSP00000332973	SMAD3	4371	0.64
ENSP00000382373	DAZL	5	0.641
ENSP00000220592	AGO2	1255	0.644
ENSP00000350720	SMARCA4	6276	0.644
ENSP00000351446	WDR5	4198	0.644
ENSP00000029410	B4GALT7	1	0.645
ENSP00000326031	PPP1CA	4391	0.645
ENSP00000343318	B3GALT5	3	0.645
ENSP00000269321	ARHGDIA	4063	0.646
ENSP00000352514	RUNX2	2782	0.648
ENSP00000389934	EXOC5	160	0.649

ENSP00000351665	CLIP1	2790	0.65
ENSP00000315702	MOB4	2992	0.651
ENSP00000347232	BLM	28	0.652
ENSP00000348877	GPI	2803	0.653
ENSP00000350967	ELP2	2	0.655
ENSP00000378529	FZR1	805	0.655
ENSP00000298316	ARF6	8093	0.656
ENSP00000303977	INO80E	799	0.656
ENSP00000304364	C1GALT1C1	6	0.656
ENSP00000267082	ITGB7	629	0.657
ENSP00000309555	HCFC1	2279	0.657
ENSP00000287934	FZD1	800	0.658
ENSP00000366396	XRN2	2195	0.658
ENSP00000284811	TCEB1	3321	0.66
ENSP00000360683	PTPN1	2097	0.66
ENSP00000373715	DCP2	800	0.661
ENSP00000260130	SDCBP	174	0.663
ENSP00000266987	TARBP2	12	0.666
ENSP00000310127	IRF3	1781	0.666
ENSP00000262193	PSMB1	28	0.667
ENSP00000263621	ELANE	734	0.667
ENSP00000263754	KAT2B	2050	0.667

ENSP00000364252	PLA2G2A	1	0.667
ENSP00000245206	GOT2	30	0.668
ENSP00000294172	NXF1	7481	0.668
ENSP00000397552	ACTL6A	1169	0.67
ENSP00000342374	SNRPD2	547	0.671
ENSP00000261304	GALC	2	0.672
ENSP00000359224	ALG14	3	0.672
ENSP00000360141	GNAS	746	0.672
ENSP00000216124	ARSA	3	0.673
ENSP00000267973	WDR61	2	0.673
ENSP00000330393	LEPR	208	0.674
ENSP00000264414	CUL3	2428	0.675
ENSP00000287766	SLC6A1	1579	0.675
ENSP00000371973	SAP18	118	0.676
ENSP00000216254	ACO2	25	0.677
ENSP00000223122	C1GALT1	2	0.678
ENSP00000340820	MAPT	5380	0.678
ENSP00000216465	GSTZ1	2	0.679
ENSP00000252444	LDLR	1731	0.682
ENSP00000303522	TACR1	4	0.682
ENSP00000231454	IL5	2	0.683
ENSP00000411286	GABBR1	2	0.683

ENSP00000265023	KNG1	5984	0.684
ENSP00000317159	CYC1	3609	0.686
ENSP00000350283	BRCA1	22363	0.688
ENSP00000250495	NEDD8	2468	0.689
ENSP00000282397	FLT1	2	0.689
ENSP00000360532	CDC5L	7	0.689
ENSP00000264170	KYNU	1	0.69
ENSP00000360372	CYP2C19	2	0.691
ENSP00000307188	ASL	11	0.693
ENSP00000342793	PLD1	2003	0.693
ENSP00000256398	ELP3	2	0.695
ENSP00000283131	SMARCA5	799	0.697
ENSP00000379625	MYD88	3201	0.697
ENSP00000218348	USP11	797	0.698
ENSP00000304736	ELOVL6	799	0.698
ENSP00000008527	CRY1	773	0.702
ENSP00000253363	RBM39	2	0.702
ENSP00000265709	ANK1	1590	0.71
ENSP00000360676	KTI12	1	0.71
ENSP00000362361	CDK9	5008	0.712
ENSP00000349594	ELAVL4	3	0.713
ENSP00000203407	UQCRC1	11	0.718

ENSP00000363851	EDA2R	708	0.719
ENSP00000221130	GSR	1	0.72
ENSP00000292807	AP2M1	1	0.723
ENSP00000381282	VIMP	793	0.723
ENSP00000287820	PPARG	1664	0.724
ENSP00000379933	TPI1	2752	0.726
ENSP00000390427	PPIL2	63	0.727
ENSP00000222673	OGDH	22	0.728
ENSP00000219255	PARD6A	2481	0.729
ENSP00000263774	NDUFS3	798	0.729
ENSP00000263694	SNRNP40	13	0.731
ENSP00000226279	CD38	12	0.733
ENSP00000253024	TRIM28	1213	0.733
ENSP00000342056	CS	44	0.734
ENSP00000363512	ALOX5	507	0.734
ENSP00000366013	GNB2L1	1832	0.734
ENSP00000370571	TH	2342	0.734
ENSP00000413493	CPSF3L	118	0.734
ENSP00000334564	POLR3C	1258	0.735
ENSP00000403154	DDAH2	4	0.736
ENSP00000390500	STK3	199	0.737
ENSP00000234396	ATP6V1B1	796	0.739

ENSP00000293217	ACOX1	2	0.741
ENSP00000351894	NCOA6	321	0.741
ENSP00000352400	NUP214	7109	0.741
ENSP00000264156	MCM6	2	0.743
ENSP00000265056	MCM2	59	0.743
ENSP00000355279	CNOT7	3	0.743
ENSP00000351490	MAX	800	0.744
ENSP00000354476	SREBF2	3992	0.744
ENSP00000249075	LIF	1085	0.746
ENSP00000341344	GGA1	2500	0.746
ENSP00000387942	SAPCD1	2	0.746
ENSP00000313432	GRHPR	1	0.748
ENSP00000354554	MT-CYB	6395	0.748
ENSP00000360031	RRP12	1	0.748
ENSP00000379330	NFATC2	862	0.748
ENSP00000231887	EHHADH	4	0.751
ENSP00000271526	PRCC	63	0.752
ENSP00000241337	GSTM2	12	0.753
ENSP00000355153	CDKN2A	2023	0.753
ENSP00000408236	CYTH2	1595	0.753
ENSP00000358716	DDX20	1254	0.754
ENSP00000271628	SF3B4	293	0.755

ENSP00000239891	ALG5	3	0.757
ENSP00000355493	ADSS	51	0.757
ENSP00000408005	SLC9A3R2	1776	0.758
ENSP00000339393	CCR6	802	0.759
ENSP00000362441	ATRX	1213	0.759
ENSP00000311430	RPL4	1	0.76
ENSP00000253004	ASS1	51	0.761
ENSP00000264335	YWHAE	9	0.761
ENSP00000319169	PRMT5	8979	0.761
ENSP00000360248	ENTPD1	3	0.762
ENSP00000348461	RAC1	22663	0.763
ENSP00000324804	PPP2R1A	1321	0.765
ENSP00000342070	CTSB	1	0.766
ENSP00000218516	GLA	3	0.767
ENSP00000308533	GEMIN2	1254	0.767
ENSP00000260970	PPIG	1754	0.768
ENSP00000359301	MAGEA3	789	0.768
ENSP00000225614	GALK1	7	0.769
ENSP00000310448	SART1	14	0.771
ENSP00000354518	ZNF830	1	0.772
ENSP00000302728	GUSB	804	0.773
ENSP00000254657	PER2	773	0.774

ENSP00000419851	GMPS	2645	0.775
ENSP00000259075	TANK	29	0.776
ENSP00000356346	PTPRC	316	0.776
ENSP00000377148	AP1G1	3	0.776
ENSP00000215071	PSMD8	65	0.777
ENSP00000263088	PLD2	1582	0.777
ENSP00000368880	FOXO1	231	0.777
ENSP00000200135	ZW10	3	0.78
ENSP00000354961	MT-ND4	6383	0.781
ENSP00000355261	SMG5	99	0.781
ENSP00000246747	ARL2	3	0.783
ENSP00000274335	PIK3R1	11701	0.784
ENSP00000339933	PKLR	4	0.784
ENSP00000353483	MAPK8	7927	0.784
ENSP00000307387	PDCD6IP	5221	0.788
ENSP00000374990	IGHG1	1	0.789
ENSP00000362592	RBBP4	3289	0.791
ENSP00000215587	POLR2E	4546	0.792
ENSP00000348784	IGBP1	800	0.793
ENSP00000264005	LCAT	9	0.794
ENSP00000296273	RFC4	14	0.794
ENSP00000316328	CIITA	26	0.796

ENSP00000348069	SREBF1	24	0.799
ENSP00000244051	MOCS3	4	0.802
ENSP00000273047	RAB5A	1227	0.802
ENSP00000216951	GSS	3	0.803
ENSP00000324648	CYP2B6	12	0.803
ENSP00000360569	SCP2	6	0.803
ENSP00000225983	HDAC5	7	0.804
ENSP00000314004	ANAPC2	255	0.805
ENSP00000361162	TOE1	4	0.805
ENSP00000410732	GABRG2	4	0.806
ENSP00000009589	RPS20	797	0.809
ENSP00000278407	SERPING1	1	0.809
ENSP00000362649	HDAC1	16120	0.813
ENSP00000356480	RNF2	5	0.814
ENSP00000216194	ADSL	54	0.815
ENSP00000264474	CSTA	1	0.816
ENSP00000267169	DIABLO	2	0.816
ENSP00000291552	U2AF1	1811	0.816
ENSP00000301838	FADD	1213	0.817
ENSP00000259469	RPL35	2423	0.818
ENSP00000367797	SKI	40	0.818
ENSP00000261733	ALDH2	20	0.819

ENSP00000367872	GNB1	40	0.82
ENSP00000270202	AKT1	75111	0.821
ENSP00000382004	CTNND1	1882	0.821
ENSP00000262027	MARS	1	0.822
ENSP00000264220	PPAT	12	0.822
ENSP00000285093	ACAA2	2	0.822
ENSP00000264444	MXD1	3	0.824
ENSP00000378191	AIMP1	1	0.824
ENSP00000253856	ATP6V0A4	5675	0.825
ENSP00000299022	LIPC	14	0.825
ENSP00000237837	FGF23	1590	0.827
ENSP00000274255	SKP2	9802	0.828
ENSP00000370023	HADHA	9	0.828
ENSP00000337224	LRAT	3	0.829
ENSP00000198939	CHERP	3	0.83
ENSP00000305790	SF3B3	12	0.831
ENSP00000229854	MCM3	886	0.835
ENSP00000297185	HSPA9	2049	0.835
ENSP00000386884	CXCR4	1580	0.835
ENSP00000303088	POLR3D	2459	0.836
ENSP00000348307	SIRPA	3124	0.836
ENSP00000353059	APAF1	796	0.836

ENSP00000358541	SIKE1	913	0.836
ENSP00000337040	UNC119	3	0.837
ENSP00000262633	RBM42	1211	0.838
ENSP00000272190	REN	796	0.839
ENSP00000222382	CYP3A43	6	0.841
ENSP00000233156	TFPI	1	0.841
ENSP00000351997	MAP2K6	1339	0.842
ENSP00000362413	PGK1	7	0.843
ENSP00000225916	KAT2A	117	0.844
ENSP00000383199	NEDD4L	1208	0.845
ENSP00000263967	PIK3CA	2164	0.847
ENSP00000364289	NCBP1	5	0.848
ENSP00000356505	NCF2	798	0.85
ENSP00000252455	PRKCSH	1	0.851
ENSP00000330720	KRTAP11-1	2	0.857
ENSP00000295797	PRKCI	1139	0.858
ENSP00000317955	EEA1	15	0.858
ENSP00000404121	ILF3	2181	0.858
ENSP00000221413	RUVBL2	165	0.859
ENSP00000341838	TNNI3	2	0.86
ENSP00000368438	PCNA	39989	0.86
ENSP00000265773	SMARCA2	17	0.861

ENSP00000359074	L1CAM	1578	0.861
ENSP00000251810	RRM2B	329	0.862
ENSP00000332258	DGAT1	7	0.863
ENSP00000307288	MCM7	398	0.865
ENSP00000225567	GOSR2	1	0.866
ENSP00000349437	IGF2R	588	0.869
ENSP00000295897	ALB	17286	0.87
ENSP00000301280	CHAF1A	4499	0.87
ENSP00000259808	RIPK1	3167	0.871
ENSP00000382595	PAICS	46	0.871
ENSP00000228916	SCNN1A	1207	0.872
ENSP00000243776	CHPF	60	0.872
ENSP00000285930	AKR1B1	6	0.872
ENSP00000321853	SERPINF2	5	0.873
ENSP00000227524	PRPF19	1175	0.874
ENSP00000236959	ATIC	1	0.874
ENSP00000370343	IRF4	14	0.874
ENSP00000294724	AGL	8	0.875
ENSP00000335544	CCKBR	31	0.875
ENSP00000335657	CCK	32	0.877
ENSP00000369871	HAUS6	794	0.878
ENSP00000290122	CELA3A	1	0.88

ENSP00000284957	RABGEF1	4287	0.882
ENSP00000315644	TYMS	15230	0.883
ENSP00000367276	CKAP2	1199	0.883
ENSP00000377303	RENBP	1	0.883
ENSP00000359939	EXOSC1	4	0.884
ENSP00000244769	ATXN1	799	0.885
ENSP00000352929	CSNK1E	789	0.885
ENSP00000196551	RPS5	1	0.886
ENSP00000355759	PARP1	1604	0.888
ENSP00000209875	CBX5	2811	0.89
ENSP00000346142	DPAGT1	3	0.891
ENSP00000363998	ITCH	1505	0.891
ENSP00000225577	RPS6KB1	183	0.892
ENSP00000311032	CASP3	2012	0.892
ENSP00000230449	EXOC2	138	0.893
ENSP00000266000	DAXX	1312	0.893
ENSP00000329411	IRF7	44	0.893
ENSP00000358335	MAP3K7	1339	0.893
ENSP00000264705	CAD	12	0.896
ENSP00000355778	H3F3A	1	0.897
ENSP00000354511	COMT	1	0.899
ENSP00000343535	USP7	3438	0.9

ENSP00000247668	TRAF2	13829	0.902
ENSP00000268097	HEXA	3	0.903
ENSP00000295767	CHCHD4	1572	0.903
ENSP00000306920	GLB1	13	0.906
ENSP00000416097	GOLGA2	2313	0.906
ENSP00000308176	BTK	30	0.907
ENSP00000351777	VCP	3175	0.907
ENSP00000364188	DDOST	1	0.907
ENSP00000383623	MLLT4	792	0.907
ENSP00000339399	CRYZ	2	0.908
ENSP00000308938	PLG	8998	0.909
ENSP00000253792	ACLY	802	0.911
ENSP00000371308	CENPJ	4	0.911
ENSP00000248566	SHFM1	22781	0.913
ENSP00000322542	GTF2I	30	0.913
ENSP00000304845	UGT1A1	25	0.914
ENSP00000309103	BAD	790	0.914
ENSP00000409612	ACADM	11	0.914
ENSP00000314949	POLR2A	7489	0.915
ENSP00000262238	YY1	2163	0.916
ENSP00000263309	CLNS1A	7781	0.916
ENSP00000264515	RBBP5	42	0.916

ENSP00000396127	RAN	11615	0.916
ENSP00000370473	IGFBP3	9456	0.918
ENSP00000254066	RARA	934	0.919
ENSP00000327070	MDH2	91	0.92
ENSP00000276682	EIF3H	122	0.921
ENSP00000361418	IPO13	1813	0.921
ENSP00000264606	HDAC4	28	0.922
ENSP00000294117	GNG3	15	0.922
ENSP00000367309	MAOB	1	0.922
ENSP00000300413	SNRPD1	1254	0.923
ENSP00000411698	USO1	1212	0.923
ENSP00000304592	FASN	3588	0.924
ENSP00000223369	YKT6	1	0.925
ENSP00000245414	IRF1	852	0.925
ENSP00000274606	NHP2	3	0.925
ENSP00000333664	ACAA1	6	0.925
ENSP00000265838	ACAT1	6	0.927
ENSP00000355518	FH	29	0.927
ENSP00000358595	CGA	811	0.927
ENSP00000391592	PTPN6	1195	0.927
ENSP00000348577	RANGAP1	11649	0.928
ENSP00000216225	RBX1	6854	0.929

ENSP00000264818	TYK2	224	0.929
ENSP00000401980	MAVS	2006	0.93
ENSP00000343054	RBM5	1479	0.931
ENSP00000229239	GAPDH	2742	0.933
ENSP00000268379	UQCRC2	6	0.933
ENSP00000323050	RBBP8	519	0.933
ENSP00000356671	SERPINC1	799	0.934
ENSP00000262803	UPF1	1332	0.935
ENSP00000382723	AGPAT1	1	0.935
ENSP00000254940	NIP7	1203	0.936
ENSP00000162749	TNFRSF1A	3637	0.937
ENSP00000381064	INTS10	42	0.937
ENSP00000225402	AATF	441	0.938
ENSP00000285814	MKI67IP	12	0.938
ENSP00000267950	ETFA	6	0.939
ENSP00000347858	XIAP	804	0.939
ENSP00000358622	IKBKG	797	0.939
ENSP00000219252	POLR2C	1199	0.94
ENSP00000228140	RPS13	6	0.94
ENSP00000354125	EIF3B	1	0.94
ENSP00000303939	CTLA4	791	0.941
ENSP00000306866	GABARAP	5	0.942

ENSP00000248114	GFER	1572	0.944
ENSP00000329623	BCL2	4572	0.944
ENSP00000262477	RABEP1	3151	0.945
ENSP00000302665	IGF1	10244	0.946
ENSP00000384708	FSHR	14	0.946
ENSP00000264246	CD80	125	0.949
ENSP00000417864	ANP32A	6	0.949
ENSP00000085219	CD22	41	0.951
ENSP00000254122	FSHB	16	0.951
ENSP00000219240	DHODH	3	0.954
ENSP00000372975	HLA-C	809	0.954
ENSP00000388526	HLA-A	789	0.954
ENSP00000262056	EIF4B	2	0.955
ENSP00000349016	PEX14	3	0.955
ENSP00000354394	STAT1	7483	0.955
ENSP00000220849	EIF3E	92	0.956
ENSP00000357051	PEX19	3	0.957
ENSP00000227758	BIRC2	1991	0.958
ENSP00000282050	ATP5A1	4	0.958
ENSP00000355890	EPRS	797	0.958
ENSP00000369213	DDX58	2008	0.958
ENSP00000326804	CUL1	722	0.96

ENSP00000335620	GSTA1	1	0.96
ENSP00000368699	ISG15	1210	0.96
ENSP00000268058	PML	1729	0.961
ENSP00000337825	LCK	17053	0.961
ENSP00000294179	STX5	1	0.962
ENSP00000337759	DOM3Z	12	0.962
ENSP00000330237	CASP9	11	0.964
ENSP00000359211	DPYD	1	0.964
ENSP00000260402	PLCB2	22	0.965
ENSP00000316879	EIF4G1	5200	0.965
ENSP00000374455	SQSTM1	2887	0.965
ENSP00000267163	RB1	17290	0.966
ENSP00000348442	PSMD12	92	0.966
ENSP00000232607	UMPS	3	0.968
ENSP00000312735	POLR2B	9689	0.97
ENSP00000362688	EIF3I	1	0.97
ENSP00000262435	SMURF2	38	0.971
ENSP00000354632	MT-ATP6	4	0.971
ENSP00000420168	GSTA2	16	0.971
ENSP00000332468	TRAF3	1971	0.972
ENSP00000292475	ATP5J2	1	0.973
ENSP00000296581	LSM6	14	0.973

ENSP00000354982	MT-CO3	4	0.974
ENSP00000358812	PDCD11	3	0.974
ENSP00000381339	GNAT3	13	0.974
ENSP00000385269	ELAVL1	2	0.976
ENSP00000262030	ATP5B	2	0.977
ENSP00000361626	YBX1	452	0.977
ENSP00000414634	LSM2	1953	0.979
ENSP00000249289	ATP6V1F	1	0.98
ENSP00000361005	ATPAF1	1	0.98
ENSP00000398597	EXOSC6	2324	0.98
ENSP00000342215	KIR2DL3	10129	0.981
ENSP00000215829	SNRPD3	8427	0.982
ENSP00000280665	DCP1B	217	0.983
ENSP00000274459	ATG12	2	0.984
ENSP00000357292	UBQLN4	5	0.984
ENSP00000292644	PSMC2	33135	0.985
ENSP00000407431	HLA-C	8555	0.985
ENSP00000223029	AIMP2	2004	0.986
ENSP00000248150	GNG13	3	0.986
ENSP00000301764	DDB1	1215	0.986
ENSP00000324897	UBE2I	14088	0.986
ENSP00000371236	GART	57	0.986

ENSP00000374354	EXOSC8	1268	0.986
ENSP00000264279	NOP58	1206	0.987
ENSP00000276201	UPF3B	1731	0.987
ENSP00000346437	ATG7	2	0.987
ENSP00000346022	RPL9	1210	0.988
ENSP00000359300	CETN2	21	0.988
ENSP00000362820	SRSF3	162	0.988
ENSP00000011653	CD4	9463	0.989
ENSP00000217958	PSMD10	107	0.989
ENSP00000216605	MTHFD1	57	0.99
ENSP00000223321	PSMA2	53	0.991
ENSP00000324890	CD28	125	0.991
ENSP00000249299	NAA38	408	0.992
ENSP00000358737	ATP5F1	2	0.992
ENSP00000310596	LSM1	668	0.993
ENSP00000302967	HDAC3	314	0.995
ENSP00000340858	B2M	8265	0.995
ENSP00000348708	UPF2	2747	0.995
ENSP00000252102	NDUFA2	10431	0.996
ENSP00000362900	SRSF4	23	0.996
ENSP00000278572	RPS3	1	0.997
ENSP00000325448	KARS	793	0.997

ENSP00000364898	SYK	18812	0.997
ENSP00000227378	HSPA8	7341	0.998
ENSP00000262629	TYROBP	15206	0.998
ENSP00000264951	XRN1	8217	0.998
ENSP00000285021	XPC	21	0.998
ENSP00000350877	SRSF2	162	0.998
ENSP00000357879	PSMD4	1616	0.998
ENSP00000358563	DKC1	3199	0.999
ENSP00000240185	TARDBP	22203	1
ENSP00000252622	LSM7	3810	1
ENSP00000256996	DDB2	1	1
ENSP00000269349	EIF4A3	902	1
ENSP00000296271	RHO	8701	1
ENSP00000314491	SRRT	1978	1
ENSP00000333001	RBM8A	76	1
ENSP00000344818	UBC	507880	1
ENSP00000350708	RAD23B	20	1
ENSP00000359345	RPL5	2395	1
ENSP00000363676	RPL11	5455	1
ENSP00000366135	EXOSC10	19794	1
ENSP00000369757	RPS6	19	1
ENSP00000377141	ARRB1	9427	1

ENSP00000417281	MDM2	22134	1

6. **Candidate genes for somatic mutation genes and mRNA genes**

Ensembl ID	Gene symbol	Betweenness	Permutation FDR
ENSP00000020926	SYT13	4	<0.001
ENSP00000228307	PXN	3913	<0.001
ENSP00000242577	DYNLL1	6746	<0.001
ENSP00000242839	ATP7B	1402	<0.001
ENSP00000253354	BPIFB1	1	<0.001
ENSP00000254942	TERF2	5203	<0.001
ENSP00000257068	MTNR1B	71	<0.001
ENSP00000265071	CDH6	1217	<0.001
ENSP00000265335	RAD50	6120	<0.001
ENSP00000265709	ANK1	3990	<0.001
ENSP00000268171	FURIN	3175	<0.001
ENSP00000276218	GPR119	10	<0.001
ENSP00000277541	NOTCH1	11069	<0.001
ENSP00000293379	ITGA5	10741	<0.001
ENSP00000295709	STK36	1583	<0.001
ENSP00000296585	ITGA2	9109	<0.001
ENSP00000297268	COL1A2	3811	<0.001
ENSP00000299367	C2	2	<0.001
ENSP00000303634	LRP8	201	<0.001

ENSP00000312435	DAG1	7678	<0.001
ENSP00000329318	CECR6	2	<0.001
ENSP00000330633	CNTN2	2303	<0.001
ENSP00000335038	VSTM2B	1	<0.001
ENSP00000337209	CCDC68	1	<0.001
ENSP00000338512	AGTPBP1	3	<0.001
ENSP00000339823	ZNF546	1	<0.001
ENSP00000344456	CTNNB1	22241	<0.001
ENSP00000349568	WDR96	1	<0.001
ENSP00000351486	NTRK1	9120	<0.001
ENSP00000355316	GRM3	3	<0.001
ENSP00000356652	CACYBP	8	<0.001
ENSP00000357986	PLEKHA1	4	<0.001
ENSP00000358525	NGF	7072	<0.001
ENSP00000360107	EFHC1	3	<0.001
ENSP00000367164	PRR20A	1	<0.001
ENSP00000371138	FKBP1A	5082	<0.001
ENSP00000371932	TAS2R1	1	<0.001
ENSP00000401303	SHC1	9326	<0.001
ENSP00000401514	DNAH1	5	<0.001
ENSP00000177648	ALPK1	1	0.001
ENSP00000216139	ACR	1	0.001

ENSP00000265080	RASGRF2	4	0.001
ENSP00000267845	HDC	1386	0.001
ENSP00000297494	NOS3	11594	0.001
ENSP00000300527	COL6A2	1	0.001
ENSP00000301200	CDC42EP5	1401	0.001
ENSP00000304669	CTNNA1	5040	0.001
ENSP00000312235	MUC13	11	0.001
ENSP00000322898	EBF1	2426	0.001
ENSP00000325123	ZSCAN2	1	0.001
ENSP00000349467	CALM1	11118	0.001
ENSP00000359074	L1CAM	5157	0.001
ENSP00000360200	INADL	1213	0.001
ENSP00000377958	CCT4	1752	0.001
ENSP00000383210	NEK3	1210	0.001
ENSP00000266376	CACNA1C	1866	0.002
ENSP00000276603	TERF1	2796	0.002
ENSP00000313084	EGFLAM	1	0.002
ENSP00000323880	FOXJ1	6	0.002
ENSP00000338217	ZNF532	1	0.002
ENSP00000338785	STARD13	1326	0.002
ENSP00000363360	INIP	1212	0.002
ENSP00000366477	C10orf112	1	0.002

ENSP00000370408	CDX2	1403	0.002
ENSP00000397181	RGS4	7	0.002
ENSP00000398930	SGCE	1	0.002
ENSP00000262219	ANXA13	2	0.003
ENSP00000298923	SLC6A5	1	0.003
ENSP00000331678	PKP3	1	0.003
ENSP00000331827	TNFAIP8L1	1	0.003
ENSP00000332353	PTCH1	6035	0.003
ENSP00000340698	GIPC1	2409	0.003
ENSP00000344468	SDC3	1252	0.003
ENSP00000352657	ME3	1208	0.003
ENSP00000352834	MYO1C	4	0.003
ENSP00000357656	FYN	13261	0.003
ENSP00000372313	MSLN	1213	0.003
ENSP00000375907	PID1	1	0.003
ENSP00000391594	ERV3-1	1	0.003
ENSP00000286621	ADK	1212	0.004
ENSP00000288986	NCK1	3228	0.004
ENSP00000308176	BTK	1931	0.004
ENSP00000310878	KLF14	1	0.004
ENSP00000351209	EPHA2	3344	0.004
ENSP00000352011	CACNA1G	1	0.004

ENSP00000372160	DOK6	1211	0.004
ENSP00000379457	FAF1	1212	0.004
ENSP00000223642	C5	1592	0.005
ENSP00000254351	SDC1	1257	0.005
ENSP00000260600	ADCY3	191	0.005
ENSP00000312185	ELMO1	1213	0.005
ENSP00000324651	TMEM108	1	0.005
ENSP00000328983	CHST6	1	0.005
ENSP00000339390	CDH26	1	0.005
ENSP00000352177	ADAM29	1	0.005
ENSP00000354586	GLI2	2358	0.005
ENSP00000358022	MCL1	5287	0.005
ENSP00000367714	HES5	1213	0.005
ENSP00000382193	MYBPC3	1217	0.005
ENSP00000390849	ABHD5	1594	0.005
ENSP00000222792	CHN2	191	0.006
ENSP00000244625	TBCC	1	0.006
ENSP00000252456	CNN1	1207	0.006
ENSP00000303077	GOT1L1	191	0.006
ENSP00000350941	SRC	17546	0.006
ENSP00000354541	NLGN1	1932	0.006
ENSP00000361125	VEGFA	5926	0.006

ENSP00000368450	CD83	191	0.006
ENSP00000369346	ARSK	2	0.006
ENSP00000162330	BCAR1	3249	0.007
ENSP00000233813	IGFBP5	1213	0.007
ENSP00000300055	PLIN1	1592	0.007
ENSP00000300417	LRSAM1	191	0.007
ENSP00000316990	TRAPPC5	191	0.007
ENSP00000328364	MAFA	191	0.007
ENSP00000352336	PLCG2	4	0.007
ENSP00000355560	TBCE	1	0.007
ENSP00000357392	EFNA1	1768	0.007
ENSP00000168712	FGF4	194	0.008
ENSP00000263666	PDZRN3	6	0.008
ENSP00000264009	HSF4	191	0.008
ENSP00000310216	KLRC4	191	0.008
ENSP00000317224	SAMD4B	1	0.008
ENSP00000333920	TTF1	191	0.008
ENSP00000354900	GJB1	191	0.008
ENSP00000361850	PLAU	2413	0.008
ENSP00000369129	DSP	1582	0.008
ENSP00000377840	CACNB1	191	0.008
ENSP00000221283	STXBP2	191	0.009

ENSP00000222399	LAMB1	1186	0.009
ENSP00000256474	VHL	16557	0.009
ENSP00000265310	TRPV5	191	0.009
ENSP00000267842	SLC27A2	186	0.009
ENSP00000268182	IQGAP1	1063	0.009
ENSP00000310658	SCUBE2	191	0.009
ENSP00000328422	PP13439	1	0.009
ENSP00000346537	SMOC2	1	0.009
ENSP00000348349	MYO9A	1	0.009
ENSP00000352785	DSG4	191	0.009
ENSP00000355001	POU3F3	191	0.009
ENSP00000357025	CD48	191	0.009
ENSP00000358576	DCLRE1B	191	0.009
ENSP00000362095	SRPX2	196	0.009
ENSP00000362372	BRWD3	191	0.009
ENSP00000368994	ARHGAP44	2	0.009
ENSP00000369009	CXorf23	191	0.009
ENSP00000375859	TMEM91	187	0.009
ENSP00000395465	NCOA4	191	0.009
ENSP00000400223	FJX1	3	0.009
ENSP00000216373	SOS2	191	0.01
ENSP00000225941	ABI3	191	0.01

ENSP00000233202	SLC11A1	191	0.01
ENSP00000233616	MOGS	191	0.01
ENSP00000245323	EFNB2	1411	0.01
ENSP00000263640	ACVR1	191	0.01
ENSP00000267415	TINF2	2390	0.01
ENSP00000268704	SPG7	191	0.01
ENSP00000281708	FBXW7	1645	0.01
ENSP00000283871	HGD	191	0.01
ENSP00000298032	ARMC3	195	0.01
ENSP00000316854	ATOX1	1401	0.01
ENSP00000348634	MYH6	1208	0.01
ENSP00000352442	HIST1H2BM	191	0.01
ENSP00000355731	CDC42BPA	3	0.01
ENSP00000359594	CLCA4	191	0.01
ENSP00000360310	SPO11	1213	0.01
ENSP00000365877	SUV39H1	191	0.01
ENSP00000374372	SPTB	1407	0.01
ENSP00000383219	KRTAP10-6	1	0.01
ENSP00000005587	SKAP2	191	0.011
ENSP00000248071	KLF2	191	0.011
ENSP00000252593	BST2	191	0.011
ENSP00000262188	SMARCD3	1326	0.011

ENSP00000265171	EGF	3260	0.011
ENSP00000295728	CRYBA2	191	0.011
ENSP00000303522	TACR1	1213	0.011
ENSP00000309595	C10orf2	1210	0.011
ENSP00000317300	LPCAT4	4	0.011
ENSP00000341170	PTN	1210	0.011
ENSP00000356641	RFWD2	1204	0.011
ENSP00000219409	ARHGDIG	188	0.012
ENSP00000266659	GLIPR1	191	0.012
ENSP00000316460	FYB	1527	0.012
ENSP00000419361	ADCY5	26	0.012
ENSP00000252050	CUL9	191	0.013
ENSP00000274793	PLA2G7	191	0.013
ENSP00000281030	THRSP	191	0.013
ENSP00000298386	RXFP2	191	0.013
ENSP00000333097	FIGLA	1	0.013
ENSP00000339861	ENY2	1389	0.013
ENSP00000345206	RBPJ	2933	0.013
ENSP00000364946	MKX	1	0.013
ENSP00000369962	IGSF5	196	0.013
ENSP00000385450	MAGI1	1208	0.013
ENSP00000392828	GPSM1	191	0.013

ENSP00000225893	HNF1B	1208	0.014
ENSP00000262545	PCSK2	191	0.014
ENSP00000265294	GABRP	191	0.014
ENSP00000297135	COG5	191	0.014
ENSP00000302114	PRELID1	191	0.014
ENSP00000329380	GP1BA	6357	0.014
ENSP00000345824	BZRAP1	1	0.014
ENSP00000350332	MYBPC2	191	0.014
ENSP00000363533	MDH1B	2	0.014
ENSP00000366563	PIK3CD	1375	0.014
ENSP00000238256	FKBP15	187	0.015
ENSP00000264033	CBL	44101	0.015
ENSP00000301420	KLK1	191	0.015
ENSP00000304188	OR8U1	24	0.015
ENSP00000347198	SRGAP1	1574	0.015
ENSP00000361867	SEMG1	191	0.015
ENSP00000365643	DOCK9	187	0.015
ENSP00000367486	MEIG1	1209	0.015
ENSP00000244289	LIPE	1601	0.016
ENSP00000261769	CDH1	3971	0.016
ENSP00000329757	ATP6V0C	191	0.016
ENSP00000340237	SH3BP4	191	0.016

ENSP00000374981	IGHA2	191	0.016
ENSP00000261681	MPP5	1201	0.017
ENSP00000264039	GPC1	9	0.017
ENSP00000338018	HIF1A	22458	0.017
ENSP00000340328	NYX	1	0.017
ENSP00000350256	CCR9	377	0.017
ENSP00000353557	SLC35F1	2	0.017
ENSP00000378326	ZP3	193	0.017
ENSP00000203556	GMIP	1	0.018
ENSP00000259938	CLPS	191	0.018
ENSP00000283147	BMP6	381	0.018
ENSP00000335203	ATPIF1	1	0.018
ENSP00000341815	SOX18	191	0.018
ENSP00000222212	CACNG7	38	0.019
ENSP00000311219	TRIM59	191	0.019
ENSP00000335153	HSP90AA1	13512	0.019
ENSP00000346294	S100A4	1592	0.019
ENSP00000348168	GTF2E2	1195	0.019
ENSP00000363390	TRIM63	121	0.019
ENSP00000231524	TRIM23	381	0.02
ENSP00000240093	FZD3	191	0.02
ENSP00000258341	LAMC1	1212	0.02

ENSP00000260643	PREB	191	0.02
ENSP00000261170	GUCY2C	191	0.02
ENSP00000309503	YWHAZ	6510	0.02
ENSP00000314458	CDC42	6523	0.02
ENSP00000332247	ATP6V0A2	191	0.02
ENSP00000358994	MYO6	1225	0.02
ENSP00000364246	PLA2G2D	190	0.02
ENSP00000393887	AHSG	381	0.02
ENSP00000223095	SERPINE1	3129	0.021
ENSP00000266682	SLC6A15	1	0.021
ENSP00000278823	MTA2	191	0.021
ENSP00000291688	MCM3AP	1389	0.021
ENSP00000328968	SCN5A	381	0.021
ENSP00000331172	CD8B	150	0.021
ENSP00000343785	SPRY1	191	0.021
ENSP00000355896	TGFB2	191	0.021
ENSP00000363431	NPY4R	188	0.021
ENSP00000363799	ACTL7B	1	0.021
ENSP00000227495	ST3GAL4	3	0.022
ENSP00000265362	SEMA3A	1206	0.022
ENSP00000273390	MAATS1	191	0.022
ENSP00000294973	HAAO	191	0.022

ENSP00000351790	MYPN	11	0.022
ENSP00000356975	ADAMTS4	195	0.022
ENSP00000360916	VAV2	1844	0.022
ENSP00000361777	SET	1212	0.022
ENSP00000380349	CAPN3	1390	0.022
ENSP00000389244	SLC44A4	1	0.022
ENSP00000200181	ITGB4	1402	0.023
ENSP00000286657	ADAMTS3	1	0.023
ENSP00000289429	CD1A	191	0.023
ENSP00000360689	TNKS2	1210	0.023
ENSP00000372547	SRY	1212	0.023
ENSP00000374323	EPHA6	1	0.023
ENSP00000389792	DCDC1	191	0.023
ENSP00000401632	GSTT1	191	0.023
ENSP00000206474	HAUS4	191	0.024
ENSP00000226091	EFNB3	191	0.024
ENSP00000247655	COX7C	191	0.024
ENSP00000255945	GIMAP4	191	0.024
ENSP00000262053	ATF1	1189	0.024
ENSP00000311697	FGF5	191	0.024
ENSP00000322542	GTF2I	1229	0.024
ENSP00000323280	CD6	375	0.024

ENSP00000340396	GBP5	191	0.024
ENSP00000365048	TNFSF13B	191	0.024
ENSP00000199764	CEACAM6	379	0.025
ENSP00000258682	CAMK2B	191	0.025
ENSP00000262776	LGALS3BP	382	0.025
ENSP00000291386	SSU72	566	0.025
ENSP00000297261	SHH	2942	0.025
ENSP00000338934	EZR	7746	0.025
ENSP00000360054	PHACTR3	191	0.025
ENSP00000408395	RBFOX3	191	0.025
ENSP00000262613	SLC9A3R1	8174	0.026
ENSP00000276420	DOK2	553	0.026
ENSP00000304767	P2RY1	383	0.026
ENSP00000350869	ZNF346	191	0.026
ENSP00000251849	RAF1	7254	0.027
ENSP00000256443	CDK7	2574	0.027
ENSP00000329968	PHKG2	190	0.027
ENSP00000333496	KCND2	191	0.027
ENSP00000353165	TPK1	191	0.027
ENSP00000413720	CDKN1C	377	0.027
ENSP00000264634	WNT5A	191	0.028
ENSP00000271620	PRUNE	17	0.028

ENSP00000291525	TFF3	1178	0.028
ENSP00000304604	MAGI3	187	0.028
ENSP00000326340	ATG16L2	1	0.028
ENSP00000357066	ARG1	1	0.028
ENSP00000372815	C4A	564	0.028
ENSP00000216064	SUN2	191	0.029
ENSP00000305442	COG7	191	0.029
ENSP00000377941	ACTN1	1399	0.029
ENSP00000381822	CDH23	7	0.029
ENSP00000220876	STMN2	191	0.03
ENSP00000266970	CDK2	9692	0.03
ENSP00000300574	CRK	3509	0.03
ENSP00000352514	RUNX2	2755	0.03
ENSP00000358323	TXNIP	1213	0.03
ENSP00000388724	HLA-A	191	0.03
ENSP00000001008	FKBP4	1583	0.031
ENSP00000201586	SULT2B1	570	0.031
ENSP00000339521	RSU1	191	0.031
ENSP00000345096	IMPDH1	190	0.031
ENSP00000348089	ERCC6	1150	0.031
ENSP00000281821	EPHA4	578	0.032
ENSP00000344742	STAMBP	1208	0.032

ENSP00000019103	SCTR	376	0.033
ENSP00000289004	HPD	191	0.033
ENSP00000337397	DKFZP686J19100	1210	0.033
ENSP00000348786	RAP1A	1619	0.033
ENSP00000362058	NDUFS5	1200	0.033
ENSP00000247170	DAAM1	377	0.034
ENSP00000260130	SDCBP	1253	0.034
ENSP00000305988	ALCAM	375	0.034
ENSP00000312999	GNAI2	2100	0.034
ENSP00000361965	ADA	1183	0.034
ENSP00000370962	GGT6	191	0.034
ENSP00000380942	ARHGEF12	1784	0.034
ENSP00000161559	CEACAM1	570	0.035
ENSP00000216277	PAPOLA	191	0.035
ENSP00000216338	GZMH	191	0.035
ENSP00000308541	F2	4446	0.035
ENSP00000327417	GPR39	190	0.035
ENSP00000328777	EFNA5	1213	0.035
ENSP00000345656	VAPA	1210	0.035
ENSP00000356056	DYNLT1	191	0.035
ENSP00000364685	MFAP2	1	0.035
ENSP00000225844	CCL13	377	0.036

ENSP00000257879	ITGA7	191	0.036
ENSP00000276072	TAF1	190	0.036
ENSP00000349204	CRB3	1201	0.036
ENSP00000366746	STAM	1208	0.036
ENSP00000396774	MUC20	2	0.036
ENSP00000260630	CYP1B1	380	0.037
ENSP00000261783	ARG2	1563	0.037
ENSP00000274311	PELO	191	0.037
ENSP00000301258	PSCA	190	0.037
ENSP00000314080	HIC1	191	0.037
ENSP00000359245	ABCA4	2	0.037
ENSP00000386896	ITGA6	1438	0.037
ENSP00000234739	BCL9	382	0.038
ENSP00000241256	GHSR	341	0.038
ENSP00000262764	PGS1	1	0.038
ENSP00000264234	UPK1B	382	0.038
ENSP00000347409	KEL	191	0.038
ENSP00000350937	TES	192	0.038
ENSP00000359285	CHRNA4	571	0.038
ENSP00000362353	GLP1R	2	0.038
ENSP00000391457	INO80C	191	0.038
ENSP00000248996	GNAZ	372	0.039

ENSP00000264126	GPSM2	382	0.039
ENSP00000283875	GTF2E1	1205	0.039
ENSP00000286604	UGT2A1	1	0.039
ENSP00000353408	MSN	382	0.039
ENSP00000357721	S100A8	375	0.039
ENSP00000400717	GNA13	1385	0.039
ENSP00000417864	ANP32A	1216	0.039
ENSP00000420418	ZNF398	189	0.039
ENSP00000264010	CTCF	191	0.04
ENSP00000266427	ETV6	191	0.04
ENSP00000286548	GNAQ	1157	0.04
ENSP00000290551	BTG2	191	0.04
ENSP00000345793	ZC3H7B	190	0.04
ENSP00000356430	RGS18	2	0.04
ENSP00000365569	FLOT1	191	0.04
ENSP00000260570	IFT172	190	0.041
ENSP00000261037	COL8A1	382	0.041
ENSP00000292169	S100A1	196	0.041
ENSP00000295006	CAPN2	191	0.041
ENSP00000300134	STAT6	2832	0.041
ENSP00000305913	COL8A2	191	0.041
ENSP00000315173	ZNF41	190	0.041

ENSP00000331902	COL4A5	378	0.041
ENSP00000340088	THEG	382	0.041
ENSP00000363163	NLGN3	1	0.041
ENSP00000225698	C1QBP	1213	0.042
ENSP00000231751	LTF	191	0.042
ENSP00000253571	RLIM	191	0.042
ENSP00000265371	NRP1	1775	0.042
ENSP00000228841	MYL2	1203	0.043
ENSP00000254301	LGALS3	382	0.043
ENSP00000260283	ARHGAP20	191	0.043
ENSP00000296181	ITGB5	8	0.043
ENSP00000349320	CACNA2D1	2	0.043
ENSP00000259206	IL1RN	191	0.044
ENSP00000319060	CAMK2G	1357	0.044
ENSP00000364802	HSPA1A	191	0.044
ENSP00000269468	MBD1	191	0.045
ENSP00000311113	JUP	1796	0.045
ENSP00000329384	IL22	191	0.045
ENSP00000386165	CEBPD	559	0.045
ENSP00000278198	LRRC4C	191	0.046
ENSP00000398852	SLC44A4	186	0.046
ENSP00000417164	ROBO2	191	0.046

ENSP00000005226	USH1C	393	0.047
ENSP00000225298	UTP18	2	0.047
ENSP00000260950	MSTN	191	0.047
ENSP00000265447	ANXA11	190	0.047
ENSP00000292174	CXCR5	1	0.047
ENSP00000319197	OR4K17	5	0.047
ENSP00000358272	NDUFAF4	191	0.047
ENSP00000281938	HSPB8	191	0.048
ENSP00000309103	BAD	635	0.048
ENSP00000323568	SLC2A2	381	0.048
ENSP00000347046	PDE5A	9	0.048
ENSP00000380227	ITGA4	2277	0.048
ENSP00000332592	SPAG16	1209	0.049
ENSP00000350162	SYCP2	382	0.049
ENSP00000357311	CENPW	191	0.049
ENSP00000387662	GCG	4459	0.049
ENSP00000252575	NCAN	4	0.05
ENSP00000264001	CKLF	190	0.05
ENSP00000265969	KCNC1	250	0.05
ENSP00000307218	NAT1	190	0.05
ENSP00000320401	UGT2B17	8	0.05
ENSP00000374409	PKP4	191	0.05

ENSP00000375009	IGHV3-9	191	0.05
ENSP00000261182	NAP1L1	190	0.051
ENSP00000316338	BAIAP2	248	0.051
ENSP00000343313	ATG5	1212	0.051
ENSP00000371341	TNK2	191	0.051
ENSP00000378090	RAD51D	191	0.051
ENSP00000308208	MMP14	200	0.052
ENSP00000334424	AMACR	1	0.052
ENSP00000351284	RAD52	191	0.052
ENSP00000357348	HEY2	191	0.052
ENSP00000396915	SCN1B	371	0.052
ENSP00000223023	WASL	3797	0.053
ENSP00000260383	TUBGCP4	187	0.053
ENSP00000354130	SOX10	382	0.053
ENSP00000272238	ATP6V1C2	381	0.054
ENSP00000304553	MPLKIP	190	0.054
ENSP00000318406	KIAA0319L	190	0.054
ENSP00000325136	HADHB	161	0.054
ENSP00000356789	ATP1B1	572	0.054
ENSP00000420174	ERVFRD-1	1	0.054
ENSP00000306095	OR10H2	1	0.055
ENSP00000309913	TBX5	192	0.055

ENSP00000310557	KCNE3	157	0.055
ENSP00000324831	OR4P4	2	0.055
ENSP00000364336	TBXA2R	822	0.055
ENSP00000366593	TMEM201	188	0.055
ENSP00000245479	SOX9	1567	0.056
ENSP00000257829	NAT10	95	0.056
ENSP00000259089	BLK	191	0.056
ENSP00000324834	MUC3A	190	0.056
ENSP00000360371	SSBP3	187	0.056
ENSP00000245457	PTGER2	2	0.057
ENSP00000276533	GINS4	13	0.057
ENSP00000280193	VEGFC	381	0.057
ENSP00000320340	DGKZ	184	0.057
ENSP00000349960	ACTB	1215	0.057
ENSP00000353654	COL4A2	376	0.057
ENSP00000372023	CHEK2	200	0.057
ENSP00000373485	TSNAXIP1	188	0.057
ENSP00000383690	MASP2	564	0.057
ENSP00000391490	AGR2	193	0.057
ENSP00000229030	FZD10	191	0.058
ENSP00000245539	MRPS7	189	0.058
ENSP00000268296	ITGAX	35	0.058

ENSP00000274711	LRRTM2	5	0.059
ENSP00000313391	DAB2	1146	0.059
ENSP00000315011	EDNRA	382	0.059
ENSP00000338562	STX3	191	0.059
ENSP00000227135	SPA17	191	0.06
ENSP00000256196	RRAS2	191	0.06
ENSP00000287766	SLC6A1	1580	0.06
ENSP00000298854	RAPSN	206	0.06
ENSP00000304822	CSN3	131	0.06
ENSP00000321674	4-Sep	191	0.06
ENSP00000332973	SMAD3	2943	0.06
ENSP00000357708	S100A6	196	0.06
ENSP00000368686	E2F4	208	0.06
ENSP00000388340	CLINT1	190	0.06
ENSP00000219172	CENPT	191	0.061
ENSP00000248933	SEZ6L	1	0.061
ENSP00000334145	F3	191	0.061
ENSP00000356602	VTA1	1184	0.061
ENSP00000357292	UBQLN4	1216	0.061
ENSP00000359297	NSDHL	1190	0.061
ENSP00000252034	ELN	1212	0.062
ENSP00000297107	GALNT10	186	0.062

ENSP00000331933	WSCD2	1	0.062
ENSP00000343479	NBR1	1195	0.062
ENSP00000343819	OTX2	190	0.062
ENSP00000358497	RNGTT	191	0.062
ENSP00000371512	SGCZ	1203	0.062
ENSP00000217381	SNTA1	381	0.063
ENSP00000262958	GNA15	190	0.063
ENSP00000318641	INTS3	1212	0.063
ENSP00000331327	WT1	1211	0.063
ENSP00000372991	LTA	382	0.063
ENSP00000405708	CCDC39	191	0.063
ENSP00000310448	SART1	1211	0.064
ENSP00000316244	HTR1A	384	0.064
ENSP00000326261	SRRM1	191	0.064
ENSP00000356623	CITED2	382	0.064
ENSP00000287139	NODAL	191	0.065
ENSP00000302665	IGF1	6563	0.065
ENSP00000305714	BMP1	381	0.065
ENSP00000250615	AANAT	10	0.066
ENSP00000310447	GLS2	1	0.066
ENSP00000347055	MYL4	3	0.066
ENSP00000351894	NCOA6	1224	0.066

ENSP00000296218	DNALI1	202	0.067
ENSP00000338728	CCDC88A	186	0.067
ENSP00000355627	AGT	1	0.067
ENSP00000386331	MYO7A	385	0.067
ENSP00000360302	GRIA3	188	0.068
ENSP00000369695	MLLT3	191	0.068
ENSP00000395546	CSNK2B	191	0.068
ENSP00000325120	PGR	191	0.069
ENSP00000350447	SGOL2	189	0.069
ENSP00000265727	ADAM22	191	0.07
ENSP00000414598	MRVI1	190	0.07
ENSP00000264834	KLF1	191	0.071
ENSP00000355587	NTPCR	191	0.071
ENSP00000356906	SH2D1B	187	0.071
ENSP00000363641	TXN	1592	0.071
ENSP00000261479	PSMA6	1208	0.072
ENSP00000264708	POMC	3104	0.072
ENSP00000325660	CNTN1	371	0.072
ENSP00000414330	RIMKLA	1	0.072
ENSP00000246533	CAPNS1	191	0.073
ENSP00000326366	PSEN1	3228	0.073
ENSP00000348234	TAT	191	0.073

ENSP00000400806	APTX	382	0.073
ENSP00000006053	CX3CL1	377	0.074
ENSP00000263369	MIA	187	0.074
ENSP00000267383	CDH24	2	0.074
ENSP00000298139	WRN	274	0.074
ENSP00000317128	PLXND1	382	0.074
ENSP00000245960	CDC25B	911	0.075
ENSP00000280326	CCT5	382	0.075
ENSP00000308938	PLG	5331	0.075
ENSP00000314897	ANGPT2	238	0.075
ENSP00000342554	STX2	3	0.075
ENSP00000351602	FUT4	1	0.075
ENSP00000381412	CAMK2A	191	0.075
ENSP00000229201	TIMELESS	190	0.076
ENSP00000321426	OR52E4	7	0.076
ENSP00000384015	SUN1	129	0.076
ENSP00000392858	TNF	191	0.076
ENSP00000311605	OR4B1	190	0.077
ENSP00000355180	COL6A1	3	0.077
ENSP00000361405	MMP9	1463	0.077
ENSP00000368754	BAZ2A	191	0.077
ENSP00000375809	ERCC2	1201	0.077

ENSP00000406335	CLIC1	191	0.077
ENSP00000215909	LGALS1	191	0.078
ENSP00000219476	TSC2	3497	0.078
ENSP00000320130	DYNC1I1	380	0.078
ENSP00000328269	HMG20B	190	0.078
ENSP00000342510	CEP97	1	0.078
ENSP00000360882	COL5A1	13	0.078
ENSP00000368314	NRCAM	1	0.078
ENSP00000286827	TIAM1	380	0.079
ENSP00000349436	ADAM15	187	0.079
ENSP00000353731	DPP4	1183	0.079
ENSP00000369654	HBD	1	0.079
ENSP00000160262	ICAM3	191	0.08
ENSP00000172229	NGFR	2075	0.08
ENSP00000380184	AGPAT6	193	0.08
ENSP00000397177	PBX2	192	0.08
ENSP00000219479	NME4	4	0.081
ENSP00000261507	MSMO1	1190	0.081
ENSP00000269485	TNFRSF11A	191	0.081
ENSP00000296785	ANKRA2	32	0.081
ENSP00000324769	OR4C6	3	0.081
ENSP00000356737	GORAB	190	0.081

ENSP00000226218	SEBOX	386	0.082
ENSP00000229854	MCM3	1435	0.082
ENSP00000301972	MYRIP	382	0.082
ENSP00000366453	TJP2	198	0.082
ENSP00000263233	SYP	191	0.083
ENSP00000265023	KNG1	3365	0.083
ENSP00000298472	SLC18A2	190	0.083
ENSP00000348888	PIGR	191	0.083
ENSP00000351327	AKAP4	2	0.083
ENSP00000357731	LOR	1	0.083
ENSP00000303212	SEMA3E	382	0.085
ENSP00000359506	FMR1	1209	0.085
ENSP00000263923	KDR	683	0.086
ENSP00000277575	USP6NL	191	0.086
ENSP00000285039	MYO5B	1	0.086
ENSP00000218388	TIMP1	404	0.087
ENSP00000355140	HOXB1	190	0.087
ENSP00000363021	RPA2	1183	0.087
ENSP00000204604	CHRD	572	0.088
ENSP00000252486	APOE	294	0.088
ENSP00000291572	AGPAT3	1	0.088
ENSP00000361658	NUP188	189	0.088

ENSP00000221930	TGFB1	4064	0.089
ENSP00000261531	SNW1	1154	0.089
ENSP00000355370	CNTF	191	0.089
ENSP00000263642	IFIH1	191	0.09
ENSP00000273221	IQSEC1	190	0.09
ENSP00000339428	SOCS2	10	0.09
ENSP00000369647	AVP	589	0.09
ENSP00000302234	CCL11	1182	0.091
ENSP00000382166	CX3CR1	377	0.091
ENSP00000231509	NR3C1	316	0.092
ENSP00000275874	RAB19	191	0.092
ENSP00000298687	NDRG2	190	0.092
ENSP00000314151	KLK3	191	0.092
ENSP00000366006	UBIAD1	380	0.092
ENSP00000362555	RNF19B	2	0.093
ENSP00000404179	DOCK4	190	0.093
ENSP00000205948	APOH	20	0.094
ENSP00000350003	CCR3	571	0.094
ENSP00000362795	CXCR3	191	0.094
ENSP00000219281	USB1	190	0.095
ENSP00000258123	USP15	382	0.095
ENSP00000278568	PAK1	1724	0.095

ENSP00000315955	FOXA2	604	0.095
ENSP00000355961	INTS7	199	0.095
ENSP00000364133	TGFBR1	4880	0.095
ENSP00000365402	HLA-C	377	0.095
ENSP00000181796	FAM107B	382	0.096
ENSP00000260766	PLCE1	13	0.096
ENSP00000285402	ODF1	191	0.097
ENSP00000394624	OPRM1	566	0.097
ENSP00000400175	RHOA	2543	0.097
ENSP00000363216	OGDHL	189	0.098
ENSP00000395535	MECP2	380	0.098
ENSP00000358918	SUFU	412	0.099
ENSP00000360968	CYP4X1	190	0.099
ENSP00000363763	EPHB2	226	0.099
ENSP00000410294	FGFR2	770	0.099
ENSP00000261267	LYZ	232	0.1
ENSP00000264426	GRIA2	7	0.1
ENSP00000355596	DISC1	191	0.1
ENSP00000257430	APC	1204	0.102
ENSP00000257963	ACVR1B	191	0.102
ENSP00000312262	ADRBK1	793	0.102
ENSP00000331358	GAST	1418	0.102

ENSP00000343040	HMGB1	191	0.102
ENSP00000344353	LPAR6	190	0.103
ENSP00000363079	MBL2	755	0.103
ENSP00000155840	KCNQ1	353	0.104
ENSP00000222725	LFNG	553	0.104
ENSP00000265132	AMBP	538	0.104
ENSP00000266085	TIMP3	190	0.104
ENSP00000362768	RBL1	178	0.104
ENSP00000296140	CCR1	573	0.105
ENSP00000306330	YWHAG	191	0.105
ENSP00000307853	MUS81	191	0.105
ENSP00000361818	SDC4	193	0.105
ENSP00000054668	UTS2	189	0.106
ENSP00000255465	CCNA1	191	0.106
ENSP00000273308	CNPY2	190	0.106
ENSP00000274031	SETD7	190	0.106
ENSP00000275605	PSPH	191	0.107
ENSP00000358857	EMD	191	0.107
ENSP00000267859	BNIP2	190	0.108
ENSP00000331201	HGS	1208	0.108
ENSP00000348273	MBP	191	0.108
ENSP00000258654	COG3	191	0.109

ENSP00000262968	TJP3	4	0.109
ENSP00000324549	CYFIP1	1209	0.109
ENSP00000364979	COL4A1	376	0.109
ENSP00000372853	C2	1	0.109
ENSP00000375921	PAX3	567	0.109
ENSP00000289902	FCER1G	573	0.11
ENSP00000353224	TFRC	399	0.11
ENSP00000409231	TRAPPC13	190	0.11
ENSP00000219255	PARD6A	1793	0.111
ENSP00000247161	ELK1	78	0.111
ENSP00000312652	LEP	5915	0.111
ENSP00000351905	TGFBR2	191	0.111
ENSP00000227667	APOC3	8	0.112
ENSP00000290100	EPB41	194	0.112
ENSP00000356000	PLXNA2	376	0.112
ENSP00000232458	ECT2	190	0.113
ENSP00000307781	ODF2	191	0.113
ENSP00000323516	UTS2R	190	0.113
ENSP00000331746	CALCA	800	0.113
ENSP00000355865	PARK2	191	0.113
ENSP00000371514	KCNV2	2	0.113
ENSP00000270458	CACNG8	149	0.114

ENSP00000291554	CRYAA	382	0.114
ENSP00000346032	ANXA2	573	0.114
ENSP00000361646	ZMYND12	183	0.114
ENSP00000396622	MROH7	191	0.114
ENSP00000254854	GUCY2D	191	0.115
ENSP00000334050	TAS2R42	2	0.115
ENSP00000350283	BRCA1	7912	0.115
ENSP00000350878	S1PR3	362	0.116
ENSP00000354623	DFNB31	192	0.116
ENSP00000299293	FRS2	12	0.117
ENSP00000356771	F5	191	0.117
ENSP00000369756	PTPRA	189	0.117
ENSP00000328181	NOG	381	0.118
ENSP00000354033	PCGF2	190	0.118
ENSP00000370473	IGFBP3	5205	0.118
ENSP00000348394	NCDN	187	0.119
ENSP00000362071	JPH2	189	0.119
ENSP00000324740	YES1	189	0.121
ENSP00000358414	HMGCS2	160	0.121
ENSP00000220584	FDFT1	1374	0.122
ENSP00000263817	ABCB11	382	0.122
ENSP00000354791	DCTN1	1571	0.122

ENSP00000378338	GIT1	737	0.122
ENSP00000401018	GINS3	189	0.122
ENSP00000254719	RPA1	1315	0.123
ENSP00000228027	DGAT2	2	0.124
ENSP00000272430	RTKN	190	0.124
ENSP00000280357	IL18	379	0.124
ENSP00000309052	CATSPER1	190	0.124
ENSP00000370223	IDH3B	1	0.124
ENSP00000184183	ROPN1	190	0.125
ENSP00000222256	RAB3A	2332	0.125
ENSP00000225655	PFN1	609	0.125
ENSP00000283006	CENPH	191	0.125
ENSP00000225831	CCL2	386	0.126
ENSP00000294304	LRP5	191	0.126
ENSP00000299413	TRIM44	1	0.126
ENSP00000348775	ACOX3	1	0.126
ENSP00000353344	ETS2	183	0.126
ENSP00000206542	OSGEP	191	0.127
ENSP00000261669	CAB39L	15	0.127
ENSP00000310244	RASGRP1	67	0.127
ENSP00000349252	ITGAL	191	0.127
ENSP00000270223	DMWD	185	0.128

ENSP00000314774	HES7	171	0.128
ENSP00000344460	CBS	188	0.128
ENSP00000357033	CD84	187	0.128
ENSP00000264638	CNTNAP1	2	0.129
ENSP00000370410	MPDZ	4	0.129
ENSP00000255380	CHRM3	1	0.13
ENSP00000279387	PPP4C	191	0.13
ENSP00000327758	NKX2-5	380	0.13
ENSP00000338983	MUC1	3899	0.13
ENSP00000251772	PLXNA1	191	0.131
ENSP00000361759	BEX2	5	0.131
ENSP00000227524	PRPF19	1422	0.132
ENSP00000272348	SNRPG	191	0.132
ENSP00000308461	RND1	565	0.132
ENSP00000354929	NOTCH2NL	382	0.132
ENSP00000357753	IVL	380	0.132
ENSP00000385019	CACNA1I	190	0.132
ENSP00000413596	PPP1R18	191	0.132
ENSP00000305689	AFF1	191	0.133
ENSP00000322784	OR5AK2	1	0.133
ENSP00000336701	RAD51C	191	0.133
ENSP00000346879	NKX2-1	572	0.133

ENSP00000410007	FBXO46	191	0.133
ENSP00000010338	TRAF3IP3	382	0.134
ENSP00000223140	NOBOX	188	0.134
ENSP00000263431	PRKCG	190	0.134
ENSP00000266544	NDUFA9	188	0.134
ENSP00000309968	ADAM17	190	0.135
ENSP00000365811	SPAG6	16	0.135
ENSP00000257254	APLNR	59	0.136
ENSP00000296145	TDGF1	191	0.136
ENSP00000308021	CEP290	1209	0.136
ENSP00000267890	TTBK2	187	0.137
ENSP00000302564	BCL2L1	1579	0.137
ENSP00000308782	GP6	33	0.137
ENSP00000234313	PLEK	191	0.138
ENSP00000261205	SYT1	2512	0.138
ENSP00000323076	NDUFAF3	191	0.138
ENSP00000340466	GANAB	191	0.138
ENSP00000348762	LSS	187	0.138
ENSP00000264657	STAT3	13347	0.139
ENSP00000295598	ATP1A1	761	0.139
ENSP00000321239	RCHY1	190	0.139
ENSP00000323580	IFT88	187	0.139

ENSP00000330237	CASP9	1134	0.139
ENSP00000228682	GLI1	403	0.14
ENSP00000254480	SMARCC1	1324	0.14
ENSP00000254950	VPS4A	1184	0.14
ENSP00000338369	CELA3B	39	0.14
ENSP00000386733	PLEKHG2	5	0.14
ENSP00000043402	RTN4R	381	0.141
ENSP00000315167	ALOX12B	190	0.141
ENSP00000355629	COG2	191	0.141
ENSP00000233114	MDH1	189	0.142
ENSP00000261708	UTP6	2	0.142
ENSP00000333194	RGS19	205	0.142
ENSP00000339467	RHOG	188	0.142
ENSP00000220809	PLAT	756	0.144
ENSP00000282588	ITGA1	41	0.144
ENSP00000263281	GIPR	10	0.145
ENSP00000268864	RASL10B	190	0.145
ENSP00000300061	SCNN1G	185	0.145
ENSP00000347379	OCLN	386	0.145
ENSP00000367446	EXT1	1	0.145
ENSP00000262839	TRPC5	195	0.146
ENSP00000330032	UPP1	1	0.146

ENSP00000340944	PTPN11	2617	0.146
ENSP00000367124	SLC3A2	191	0.146
ENSP00000350009	USP33	14	0.147
ENSP00000222390	HGF	191	0.148
ENSP00000250448	FOXA1	572	0.148
ENSP00000263710	CLASP1	5	0.148
ENSP00000304592	FASN	2952	0.148
ENSP00000369325	CDKL5	190	0.148
ENSP00000373106	RNF39	1	0.149
ENSP00000354251	NCKAP1	1209	0.15
ENSP00000377717	ST3GAL6	1	0.15
ENSP00000318445	ST3GAL1	191	0.151
ENSP00000334008	PARVA	191	0.151
ENSP00000345708	KCNJ11	4	0.151
ENSP00000262367	CREBBP	10010	0.152
ENSP00000351155	ATL1	190	0.152
ENSP00000352798	COL18A1	541	0.152
ENSP00000366620	H6PD	1	0.152
ENSP00000301050	CACNB3	9	0.153
ENSP00000346155	UCKL1	2	0.155
ENSP00000353099	HLA-DRB1	1213	0.156
ENSP00000379310	CASC1	373	0.156

ENSP00000382697	ROCK1	8	0.156
ENSP00000233809	IGFBP2	190	0.157
ENSP00000248594	PTPN12	190	0.158
ENSP00000290200	IL10RB	191	0.158
ENSP00000276033	SLC16A2	190	0.159
ENSP00000053867	GRN	180	0.16
ENSP00000280346	DLAT	194	0.161
ENSP00000294353	ZYG11B	362	0.161
ENSP00000357244	CCT3	190	0.161
ENSP00000262768	TIMP2	197	0.162
ENSP00000311827	MSL2	189	0.162
ENSP00000405890	PBX1	381	0.162
ENSP00000328878	OR52E6	1	0.163
ENSP00000348573	AKAP9	4	0.163
ENSP00000256958	SLCO1B1	382	0.164
ENSP00000265276	GPAM	172	0.165
ENSP00000337103	CHAT	191	0.165
ENSP00000308268	CARNS1	1	0.166
ENSP00000327801	P4HB	381	0.166
ENSP00000263645	CD81	187	0.168
ENSP00000264998	TF	375	0.168
ENSP00000219473	USP10	96	0.169

ENSP00000263360	EED	30	0.169
ENSP00000264554	SHC2	652	0.169
ENSP00000358421	HSD3B1	19	0.169
ENSP00000291700	S100B	1560	0.17
ENSP00000301788	POLR2G	283	0.17
ENSP00000292301	CCR2	8	0.171
ENSP00000306512	IL8	3028	0.171
ENSP00000318351	BCKDHB	187	0.171
ENSP00000262375	DNAJA3	11	0.172
ENSP00000290039	CACHD1	6	0.172
ENSP00000263182	BBOX1	191	0.173
ENSP00000368683	EDN1	745	0.173
ENSP00000175506	ASNS	376	0.174
ENSP00000292907	COX7A1	190	0.174
ENSP00000331544	FBLN1	185	0.174
ENSP00000361554	TIE1	190	0.174
ENSP00000365682	TLE1	413	0.174
ENSP00000353452	MYLK	2322	0.175
ENSP00000313921	MSRA	6	0.176
ENSP00000321326	F2R	189	0.176
ENSP00000381064	INTS10	1206	0.176
ENSP00000392466	LDB1	191	0.176

ENSP00000349298	MYLIP	190	0.177
ENSP00000366488	PRKACG	10	0.177
ENSP00000317327	UBASH3A	190	0.178
ENSP00000400842	HLA-B	5	0.178
ENSP00000236147	SELL	191	0.179
ENSP00000353154	NFASC	223	0.179
ENSP00000370557	MIS12	190	0.179
ENSP00000293272	CCL5	1141	0.18
ENSP00000255608	BTBD2	181	0.181
ENSP00000283195	RANBP2	176	0.181
ENSP00000305255	STX8	3	0.181
ENSP00000360035	PPP1R3D	3	0.181
ENSP00000227163	SPI1	191	0.182
ENSP00000299299	PCBD1	190	0.182
ENSP00000230340	BYSL	185	0.183
ENSP00000261797	NDST1	1	0.183
ENSP00000367408	CASK	1663	0.183
ENSP00000216392	PYGL	190	0.184
ENSP00000303325	TACR3	4	0.184
ENSP00000307280	MYL1	8	0.184
ENSP00000368169	DVL1	950	0.184
ENSP00000415183	MUC2	380	0.184

ENSP00000309591	PRKACA	10	0.185
ENSP00000347197	C5AR1	9	0.185
ENSP00000359151	DBT	130	0.185
ENSP00000261023	ITGAV	542	0.186
ENSP00000310170	FOSL1	569	0.186
ENSP00000356579	CEP350	191	0.186
ENSP00000368350	TPT1	758	0.186
ENSP00000374455	SQSTM1	2353	0.186
ENSP00000256720	LPIN1	191	0.187
ENSP00000260356	THBS1	115	0.187
ENSP00000287713	NMNAT2	1	0.188
ENSP00000334122	FGF3	760	0.188
ENSP00000337127	SOD2	572	0.188
ENSP00000343054	RBM5	1391	0.188
ENSP00000327070	MDH2	1201	0.189
ENSP00000356671	SERPINC1	1403	0.189
ENSP00000305464	APLN	9	0.19
ENSP00000361381	SLC6A9	2	0.19
ENSP00000404121	ILF3	1551	0.19
ENSP00000229264	GNB3	185	0.191
ENSP00000265354	SRF	1776	0.191
ENSP00000363435	ITPR3	75	0.191

ENSP00000171214	RDH8	1	0.192
ENSP00000252674	MLLT1	191	0.192
ENSP00000265708	ADAM2	190	0.192
ENSP00000351908	MAP3K5	2103	0.192
ENSP00000228850	AKAP3	757	0.193
ENSP00000351665	CLIP1	1568	0.193
ENSP00000306881	SEC23A	191	0.194
ENSP00000330284	NPBWR1	188	0.194
ENSP00000364893	ARHGEF7	477	0.194
ENSP00000365663	NPPA	772	0.194
ENSP00000384675	SOS1	725	0.194
ENSP00000417404	HFE	555	0.194
ENSP00000360268	ALDH18A1	191	0.195
ENSP00000269280	NLRP1	190	0.196
ENSP00000313936	OR2AE1	4	0.196
ENSP00000256759	FST	191	0.197
ENSP00000256010	NTS	224	0.198
ENSP00000275603	CCT6A	15	0.198
ENSP00000341032	WNT7B	191	0.2
ENSP00000363708	BMPR2	382	0.2
ENSP00000257497	ANXA1	189	0.201
ENSP00000044462	PSMA4	727	0.203

ENSP00000301838	FADD	1196	0.203
ENSP00000223114	MOGAT3	3	0.204
ENSP00000284818	LY96	376	0.204
ENSP00000199280	AQP2	190	0.205
ENSP00000253792	ACLY	1375	0.205
ENSP00000344822	S100A13	6	0.205
ENSP00000363092	PRKG1	585	0.205
ENSP00000225396	TADA2A	191	0.206
ENSP00000405041	POU5F1	310	0.206
ENSP00000243349	ACVR1C	190	0.207
ENSP00000270474	PDE4A	397	0.207
ENSP00000366347	NKX2-2	190	0.207
ENSP00000295797	PRKCI	1012	0.208
ENSP00000262134	LPCAT2	190	0.209
ENSP00000316578	SUZ12	191	0.21
ENSP00000365851	BMI1	1210	0.21
ENSP00000264637	THRA	41	0.211
ENSP00000328708	RXFP3	1	0.211
ENSP00000289779	F11R	161	0.212
ENSP00000348815	HYLS1	1	0.212
ENSP00000316333	CD55	190	0.213
ENSP00000242152	NPY	946	0.214

ENSP00000272167	EPHX1	186	0.214
ENSP00000296440	PLXNB1	563	0.214
ENSP00000320940	NCOA1	4174	0.214
ENSP00000360316	DHCR24	193	0.214
ENSP00000368965	HGSNAT	1	0.214
ENSP00000417257	FNDC3A	3	0.214
ENSP00000228837	FGF6	1139	0.215
ENSP00000305595	B3GNT2	4	0.215
ENSP00000358866	FLNA	742	0.215
ENSP00000362403	TACR2	1	0.215
ENSP00000052754	DCN	1953	0.216
ENSP00000260795	FGFR3	380	0.216
ENSP00000357177	ARHGEF11	381	0.217
ENSP00000315325	ARHGEF2	188	0.218
ENSP00000363384	ZNF593	1	0.218
ENSP00000396620	NFYC	188	0.218
ENSP00000342082	SLPI	180	0.22
ENSP00000248553	HSPB1	934	0.221
ENSP00000263918	STRN	45	0.222
ENSP00000264741	ITGA9	190	0.222
ENSP00000412788	PTBP2	1	0.222
ENSP00000321345	IL23R	17	0.223

ENSP00000311469	GSTM1	517	0.224
ENSP00000347858	XIAP	1322	0.224
ENSP00000359206	BTRC	228	0.224
ENSP00000361186	TP53RK	191	0.225
ENSP00000361636	TNNC2	17	0.225
ENSP00000252945	CYP2E1	762	0.229
ENSP00000227507	CCND1	13059	0.23
ENSP00000206249	ESR1	23288	0.231
ENSP00000291901	TNNT1	366	0.232
ENSP00000345728	ATP7A	3	0.232
ENSP00000355747	PSEN2	191	0.233
ENSP00000380702	MYCBP	1709	0.233
ENSP00000396439	RING1	189	0.233
ENSP00000229266	CHPT1	181	0.234
ENSP00000241052	CAT	381	0.236
ENSP00000293288	BAX	865	0.237
ENSP00000382819	DOM3Z	186	0.237
ENSP00000269397	CBX4	1399	0.238
ENSP00000358223	PNLIP	1	0.239
ENSP00000362082	CCND3	377	0.239
ENSP00000288266	APPL1	191	0.24
ENSP00000335074	GHRL	531	0.241

ENSP00000370503	CCM2	190	0.241
ENSP00000308012	PABPC5	1	0.242
ENSP00000394400	SKIV2L	1	0.242
ENSP00000224337	BLNK	486	0.243
ENSP00000288602	BRAF	191	0.244
ENSP00000342656	EXT2	1	0.244
ENSP00000361878	CAP1	7	0.244
ENSP00000222005	CDC37	1518	0.246
ENSP00000304350	PRPF8	219	0.246
ENSP00000378130	CFI	9	0.247
ENSP00000382004	CTNND1	1031	0.247
ENSP00000298937	ELP4	190	0.248
ENSP00000343418	SEMA4D	156	0.248
ENSP00000374467	ABCC8	1	0.248
ENSP00000339917	TAF9B	187	0.249
ENSP00000342755	RNF41	377	0.249
ENSP00000380280	FGFR1	3180	0.249
ENSP00000262105	MCM4	558	0.25
ENSP00000263409	LIFR	191	0.25
ENSP00000301532	OR5I1	1	0.25
ENSP00000349275	NRG1	30	0.25
ENSP00000237014	TTR	575	0.251

ENSP00000302021	MUC7	3	0.251
ENSP00000317714	STX4	1779	0.251
ENSP00000313752	SSNA1	190	0.252
ENSP00000382791	GRIK1	186	0.252
ENSP00000304915	IL13	190	0.253
ENSP00000309845	HRAS	3202	0.254
ENSP00000322570	POLE	191	0.254
ENSP00000233946	IL1R1	382	0.255
ENSP00000264832	ICAM1	25	0.255
ENSP00000320885	SPAST	190	0.255
ENSP00000344666	NF2	1	0.255
ENSP00000384665	LPAR2	424	0.255
ENSP00000257904	CDK4	1770	0.256
ENSP00000354621	SMURF1	382	0.256
ENSP00000363081	DKK1	190	0.256
ENSP00000369889	COL2A1	202	0.256
ENSP00000263038	PHYH	186	0.257
ENSP00000265517	MTTP	6	0.257
ENSP00000314036	AGPAT4	1	0.258
ENSP00000310551	LCLAT1	4	0.259
ENSP00000372860	VARS2	148	0.26
ENSP00000251337	GNAT2	191	0.261

ENSP00000370589	NOP56	183	0.261
ENSP00000358997	IRAK1	2704	0.262
ENSP00000261303	PSMC1	4	0.263
ENSP00000262186	KCNH2	979	0.263
ENSP00000302707	FPR1	185	0.264
ENSP00000270202	AKT1	21066	0.265
ENSP00000304283	RAC3	186	0.266
ENSP00000283635	CD8A	172	0.267
ENSP00000297564	COX6C	190	0.267
ENSP00000221770	POP4	2	0.269
ENSP00000278916	CHEK1	454	0.269
ENSP00000228280	KITLG	189	0.271
ENSP00000323929	A2M	6	0.271
ENSP00000354532	PNP	3	0.271
ENSP00000245907	C3	959	0.273
ENSP00000254227	NR0B2	191	0.273
ENSP00000259633	CD72	89	0.273
ENSP00000267082	ITGB7	147	0.273
ENSP00000363779	IKBKAP	1	0.273
ENSP00000231572	RARS	191	0.274
ENSP00000265563	PRKAR2A	1138	0.274
ENSP00000286301	CSF1R	378	0.274

ENSP00000367462	OLAH	186	0.276
ENSP00000391681	POU5F1	183	0.276
ENSP00000272298	CALM2	3	0.277
ENSP00000260010	TLR2	191	0.279
ENSP00000264914	ARSB	190	0.279
ENSP00000410076	CASP1	760	0.279
ENSP00000302269	VAV1	2163	0.28
ENSP00000321259	TALDO1	191	0.282
ENSP00000240055	NFYB	188	0.283
ENSP00000244007	PLCG1	4457	0.283
ENSP00000314214	VAMP2	1960	0.283
ENSP00000219789	CDIPT	1	0.284
ENSP00000245451	BMP4	974	0.285
ENSP00000262052	SLC11A2	4	0.287
ENSP00000246891	CSN1S1	381	0.288
ENSP00000360676	KTI12	1	0.288
ENSP00000276414	GNRH1	379	0.292
ENSP00000264409	AGPAT9	1	0.293
ENSP00000337915	CYP3A4	196	0.294
ENSP00000348551	NCOR2	543	0.294
ENSP00000246032	STK35	186	0.295
ENSP00000263025	MAPK3	78	0.295

ENSP00000355812	FGFR1OP	191	0.295
ENSP00000259895	GTF2H4	190	0.296
ENSP00000276571	CRH	212	0.296
ENSP00000299421	ILK	1183	0.297
ENSP00000380378	PAFAH1B1	13	0.298
ENSP00000295897	ALB	6081	0.299
ENSP00000046794	LCP2	704	0.3
ENSP00000312244	MSL3	189	0.3
ENSP00000226359	AFP	3	0.303
ENSP00000301141	CYP2A6	2	0.303
ENSP00000325690	CARM1	5	0.303
ENSP00000307786	CYCS	191	0.305
ENSP00000157812	PSMC4	752	0.306
ENSP00000362131	RPA4	184	0.306
ENSP00000219070	MMP2	913	0.308
ENSP00000386284	ALAD	171	0.308
ENSP00000217961	STS	191	0.309
ENSP00000262304	PKD1	168	0.309
ENSP00000287497	ITGAM	773	0.309
ENSP00000293970	TBC1D24	195	0.309
ENSP00000204961	EFNB1	2	0.31
ENSP00000334458	GATA4	382	0.311

ENSP00000354720	SMC3	936	0.311
ENSP00000359300	CETN2	1196	0.311
ENSP00000348827	THRB	10	0.312
ENSP00000357283	LMNA	191	0.315
ENSP00000369055	B4GALT1	378	0.315
ENSP00000211998	VCL	381	0.316
ENSP00000232014	BCL6	194	0.316
ENSP00000303532	DEFB4A	191	0.316
ENSP00000337736	AKAP1	1329	0.317
ENSP00000355599	TSNAX	188	0.317
ENSP00000257818	LMO2	195	0.318
ENSP00000302961	HSPA4	808	0.318
ENSP00000396219	MEF2C	207	0.318
ENSP00000239451	SLC25A2	2	0.319
ENSP00000296154	CASR	182	0.319
ENSP00000270142	SOD1	572	0.32
ENSP00000281453	MLF1IP	344	0.321
ENSP00000343144	PARD6G	190	0.321
ENSP00000338345	SNCA	751	0.322
ENSP00000347184	HTT	569	0.324
ENSP00000347719	TBCD	9	0.326
ENSP0000007708	PDK2	250	0.327

ENSP00000326630	ZFPM1	2	0.327
ENSP00000286317	MED7	2	0.328
ENSP00000285021	XPC	1196	0.329
ENSP00000303315	JUNB	6	0.329
ENSP00000356991	PVRL4	188	0.33
ENSP00000408910	DCTN2	2	0.33
ENSP00000419692	RXRA	1240	0.331
ENSP00000261799	PDGFRB	752	0.333
ENSP00000369643	GRPR	4	0.333
ENSP00000257555	HNF1A	190	0.336
ENSP00000302177	MBOAT2	2	0.336
ENSP00000300161	YWHAB	598	0.337
ENSP00000410321	LY6G5C	6	0.337
ENSP00000347942	RET	535	0.339
ENSP00000411286	GABBR1	1	0.34
ENSP00000261366	LMNB1	486	0.341
ENSP00000364709	F10	192	0.341
ENSP00000333275	NR2C1	5	0.343
ENSP00000337761	RAB27A	383	0.343
ENSP00000216456	VTI1B	190	0.344
ENSP00000350249	POT1	186	0.344
ENSP00000267973	WDR61	1	0.347

ENSP00000218348	USP11	191	0.349
ENSP00000315442	NR1H4	194	0.349
ENSP00000217244	CSNK2A1	1144	0.35
ENSP00000254998	NXT1	191	0.35
ENSP00000391349	DOM3Z	389	0.352
ENSP00000364320	MRTO4	1	0.353
ENSP00000262965	TCF3	947	0.354
ENSP00000309259	ALAS1	171	0.354
ENSP00000263033	SYTL4	380	0.355
ENSP00000354522	TOP1	181	0.356
ENSP00000354476	SREBF2	1561	0.357
ENSP00000356070	MAPKAPK2	1059	0.357
ENSP00000264018	DDX6	189	0.358
ENSP00000381717	UBE2D2	191	0.358
ENSP00000227752	IL10RA	191	0.359
ENSP00000285379	CA2	186	0.359
ENSP00000306614	PPIH	148	0.359
ENSP00000391069	SRPK1	101	0.36
ENSP00000261900	CCNT1	194	0.361
ENSP00000243077	LRP1	154	0.362
ENSP00000267996	TPM1	950	0.362
ENSP00000305958	STIP1	190	0.362

ENSP00000283131	SMARCA5	191	0.363
ENSP00000349049	KDM1A	196	0.364
ENSP00000357301	RXFP4	182	0.364
ENSP00000359663	CD40LG	191	0.364
ENSP00000363921	PARD3	560	0.365
ENSP00000285398	ERCC3	1	0.366
ENSP00000302150	PRL	1389	0.366
ENSP00000252444	LDLR	498	0.367
ENSP00000278385	CD44	88	0.367
ENSP00000216341	GZMB	191	0.368
ENSP00000350967	ELP2	1	0.369
ENSP00000360569	SCP2	3	0.37
ENSP00000329623	BCL2	1919	0.371
ENSP00000247461	CANX	1618	0.375
ENSP00000257192	DSG1	188	0.375
ENSP00000292303	CCR5	1432	0.375
ENSP00000355325	PSMB5	715	0.375
ENSP00000361423	ABL1	1932	0.375
ENSP00000261207	PPP1R12A	189	0.376
ENSP00000358105	APH1A	191	0.376
ENSP00000223208	CEP41	3	0.377
ENSP00000338297	IGF2	386	0.377

ENSP00000223129	RPA3	231	0.379
ENSP00000261755	FAH	190	0.379
ENSP00000348107	C1D	187	0.379
ENSP00000348069	SREBF1	24	0.383
ENSP00000405934	ITPR1	585	0.383
ENSP00000367797	SKI	18	0.385
ENSP00000333769	BSG	192	0.387
ENSP00000264005	LCAT	7	0.39
ENSP00000299402	APBB1	1935	0.39
ENSP00000306245	FOS	7274	0.39
ENSP00000170630	IL4R	315	0.392
ENSP00000347839	RAB11FIP2	1	0.392
ENSP00000387699	CREB1	343	0.394
ENSP00000286398	SMC2	187	0.395
ENSP00000349465	PICK1	193	0.395
ENSP00000320866	CALR	1125	0.401
ENSP00000361151	CEL	190	0.401
ENSP00000381607	GSTP1	274	0.402
ENSP00000351446	WDR5	1213	0.403
ENSP00000332643	NDN	216	0.404
ENSP00000282344	USP12	185	0.405
ENSP00000401980	MAVS	1399	0.405

ENSP00000265094	FBXW11	24	0.406
ENSP00000303977	INO80E	191	0.406
ENSP00000379204	BMP7	191	0.406
ENSP00000354826	CALD1	936	0.407
ENSP00000261837	GNB5	1	0.409
ENSP00000205402	DLD	508	0.411
ENSP00000345731	DLG1	381	0.411
ENSP00000264972	ZAP70	185	0.414
ENSP00000354927	MAP3K3	190	0.417
ENSP00000315702	MOB4	862	0.418
ENSP00000360248	ENTPD1	1	0.418
ENSP00000351896	TRAPPC4	381	0.419
ENSP00000350616	DDC	552	0.421
ENSP00000366396	XRN2	536	0.421
ENSP00000356438	PTGS2	2094	0.423
ENSP00000327583	RANBP1	1	0.424
ENSP00000371432	PRLR	1340	0.424
ENSP00000312017	FAM57A	1	0.426
ENSP00000316377	DLGAP1	379	0.426
ENSP00000384273	RELA	3188	0.43
ENSP00000287934	FZD1	191	0.432
ENSP00000259808	RIPK1	1373	0.433

ENSP00000313350	RNASEH1	1	0.433
ENSP00000265641	CPT1A	30	0.434
ENSP00000330393	LEPR	72	0.435
ENSP00000300289	PDIA3	1052	0.436
ENSP00000368104	BMP2	1770	0.436
ENSP00000262887	XRCC1	380	0.438
ENSP00000299424	TAF10	189	0.44
ENSP00000318472	NCAM1	566	0.443
ENSP00000373614	SELPLG	191	0.444
ENSP00000338799	IL6ST	497	0.445
ENSP00000361021	PTEN	441	0.447
ENSP00000263774	NDUFS3	191	0.449
ENSP00000338868	PHF8	190	0.449
ENSP00000354782	CD247	189	0.451
ENSP00000335304	DLST	192	0.452
ENSP00000316029	TLN1	2	0.453
ENSP00000307491	WDR48	185	0.455
ENSP00000222382	CYP3A43	3	0.46
ENSP00000256398	ELP3	1	0.461
ENSP00000307188	ASL	3	0.463
ENSP00000351490	MAX	191	0.463
ENSP00000225983	HDAC5	14	0.464

ENSP00000363851	EDA2R	183	0.464
ENSP00000381331	HDAC2	1153	0.464
ENSP00000362900	SRSF4	57	0.465
ENSP00000242057	AHR	1888	0.466
ENSP00000360181	SH2D1A	187	0.466
ENSP00000344579	FSCB	185	0.468
ENSP00000229307	NANOG	70	0.469
ENSP00000265038	ERCC8	42	0.469
ENSP00000379625	MYD88	763	0.47
ENSP00000231454	IL5	1	0.472
ENSP00000262160	SMAD2	2695	0.472
ENSP00000303242	ITGB2	974	0.472
ENSP00000297338	RAD21	49	0.475
ENSP00000321853	SERPINF2	5	0.478
ENSP00000256996	DDB2	56	0.48
ENSP00000261195	GYS2	1	0.481
ENSP00000348986	INS-IGF2	2023	0.483
ENSP00000350708	RAD23B	1140	0.484
ENSP00000361066	NCOA3	180	0.485
ENSP00000234396	ATP6V1B1	191	0.486
ENSP00000354901	CXCL9	186	0.486
ENSP00000380252	NFE2L2	124	0.486

ENSP00000299518	IDH3A	1	0.487
ENSP00000264705	CAD	16	0.49
ENSP00000303830	INSR	2013	0.49
ENSP00000351777	VCP	1399	0.491
ENSP00000231449	IL4	1	0.494
ENSP00000339393	CCR6	191	0.494
ENSP00000335657	CCK	41	0.495
ENSP00000417764	ALG2	382	0.495
ENSP00000265056	MCM2	50	0.498
ENSP00000287820	PPARG	398	0.5
ENSP00000318861	SF3B2	200	0.501
ENSP00000261819	ANAPC5	7	0.502
ENSP00000374280	RTF1	189	0.503
ENSP00000408236	CYTH2	381	0.504
ENSP00000258301	STX6	191	0.507
ENSP00000261461	PPP2R5A	463	0.509
ENSP00000332468	TRAF3	1382	0.511
ENSP00000350720	SMARCA4	1518	0.511
ENSP00000256216	HSD17B4	1	0.513
ENSP00000408005	SLC9A3R2	424	0.513
ENSP00000263621	ELANE	180	0.514
ENSP00000313681	SPHK1	191	0.514

ENSP00000351407	ARNT	1788	0.515
ENSP00000264156	MCM6	2	0.518
ENSP00000360141	GNAS	175	0.518
ENSP00000006275	TRAPPC6A	190	0.519
ENSP00000309629	CFL1	3	0.522
ENSP00000276689	NDUFB9	4	0.525
ENSP00000335544	CCKBR	41	0.525
ENSP00000249075	LIF	258	0.526
ENSP00000348965	DYNC1H1	1	0.526
ENSP00000230354	TBP	2787	0.527
ENSP00000219240	DHODH	3	0.53
ENSP00000261890	RAB11A	1	0.53
ENSP00000333982	NDEL1	197	0.53
ENSP00000306497	KCNJ4	2	0.531
ENSP00000218099	F9	1	0.532
ENSP00000313420	PRKDC	1729	0.532
ENSP00000382723	AGPAT1	1	0.538
ENSP00000342011	XRCC4	2	0.539
ENSP00000356505	NCF2	191	0.539
ENSP00000369871	HAUS6	191	0.539
ENSP00000226574	NFKB1	20	0.54
ENSP00000229794	MAPK14	1443	0.541

ENSP00000300738	RRM1	57	0.543
ENSP00000233156	TFPI	1	0.545
ENSP00000307387	PDCD6IP	1564	0.546
ENSP00000338964	GGT7	1	0.546
ENSP00000339328	PLAUR	380	0.547
ENSP00000308741	CLOCK	172	0.549
ENSP00000371308	CENPJ	10	0.549
ENSP00000339353	CPSF1	191	0.551
ENSP00000356587	NPHS2	764	0.552
ENSP00000290122	CELA3A	1	0.553
ENSP00000367934	UQCRQ	1	0.554
ENSP00000221972	CD79A	190	0.555
ENSP00000263088	PLD2	375	0.555
ENSP00000316176	UBE2N	191	0.555
ENSP00000343745	DICER1	191	0.558
ENSP00000353059	APAF1	191	0.558
ENSP00000379330	NFATC2	199	0.559
ENSP00000341838	TNNI3	25	0.56
ENSP00000232461	GNAT1	191	0.561
ENSP00000264220	PPAT	10	0.562
ENSP00000348784	IGBP1	191	0.564
ENSP00000367830	PRKCZ	190	0.564

ENSP00000339992	MYB	598	0.565
ENSP00000352138	KIRREL	573	0.565
ENSP00000357879	PSMD4	1519	0.568
ENSP00000220592	AGO2	191	0.569
ENSP00000361359	CD40	191	0.57
ENSP00000370571	TH	551	0.57
ENSP00000232607	UMPS	3	0.573
ENSP00000344936	PTTG1	191	0.574
ENSP00000348554	CDC16	191	0.574
ENSP00000263967	PIK3CA	578	0.576
ENSP00000337224	LRAT	1	0.576
ENSP00000329357	SP1	2106	0.577
ENSP00000386884	CXCR4	377	0.579
ENSP00000269300	PIK3R5	187	0.585
ENSP00000363512	ALOX5	124	0.585
ENSP00000179259	C12orf5	188	0.586
ENSP00000391901	PHF1	191	0.588
ENSP00000341551	SMAD4	1777	0.591
ENSP00000269571	ERBB2	1923	0.592
ENSP00000381282	VIMP	188	0.596
ENSP00000363998	ITCH	370	0.597
ENSP00000302728	GUSB	191	0.598

ENSP00000304736	ELOVL6	189	0.599
ENSP00000237837	FGF23	381	0.601
ENSP00000356425	UCHL5	356	0.602
ENSP00000334448	GNG2	198	0.606
ENSP00000008527	CRY1	172	0.61
ENSP00000354518	ZNF830	1	0.61
ENSP00000226730	IL2	204	0.612
ENSP00000379110	CXCL1	189	0.614
ENSP00000334188	PFDN5	191	0.616
ENSP00000244769	ATXN1	203	0.619
ENSP00000372793	LTA	119	0.622
ENSP00000373715	DCP2	189	0.622
ENSP00000418447	PPP2CA	3148	0.622
ENSP00000229022	VDR	1147	0.624
ENSP00000246747	ARL2	7	0.625
ENSP00000249647	SNAP23	381	0.625
ENSP00000326031	PPP1CA	763	0.631
ENSP00000340330	KAT5	2801	0.631
ENSP00000288135	KIT	189	0.638
ENSP00000359211	DPYD	1	0.638
ENSP00000355536	MTR	1	0.64
ENSP00000370343	IRF4	3	0.64

ENSP00000215832	MAPK1	2392	0.642
ENSP00000203407	UQCRC1	1	0.643
ENSP00000262158	SMAD7	1461	0.644
ENSP00000354876	MT-CO2	380	0.644
ENSP00000406037	KAT8	189	0.645
ENSP00000324804	PPP2R1A	273	0.649
ENSP00000365775	MTHFR	1	0.649
ENSP00000222673	OGDH	4	0.651
ENSP00000245206	GOT2	4	0.651
ENSP00000348307	SIRPA	721	0.653
ENSP00000359301	MAGEA3	187	0.654
ENSP00000312122	SEC13	191	0.656
ENSP00000216254	ACO2	1	0.657
ENSP00000330720	KRTAP11-1	1	0.659
ENSP00000262305	RAB11FIP3	1	0.66
ENSP00000337040	UNC119	7	0.66
ENSP00000367872	GNB1	11	0.662
ENSP00000257770	NT5E	2	0.663
ENSP00000284811	TCEB1	498	0.665
ENSP00000259512	DERL1	188	0.666
ENSP00000254657	PER2	172	0.667
ENSP00000400591	SNRPE	13	0.669

ENSP00000262435	SMURF2	23	0.671
ENSP00000356346	PTPRC	67	0.672
ENSP00000410732	GABRG2	1	0.673
ENSP00000348877	GPI	383	0.675
ENSP00000379933	TPI1	379	0.676
ENSP00000311344	PPP2R1B	48	0.677
ENSP00000320180	GHRHR	1	0.681
ENSP00000251968	TSG101	1830	0.682
ENSP00000365891	WAS	745	0.683
ENSP00000267101	ERBB3	411	0.685
ENSP00000356918	STX7	193	0.686
ENSP00000316032	NUP98	191	0.689
ENSP00000263864	VAMP8	193	0.69
ENSP00000352264	CD2AP	764	0.691
ENSP00000352121	PIK3CG	187	0.692
ENSP00000296871	CSF2	275	0.693
ENSP00000413493	CPSF3L	12	0.696
ENSP00000009589	RPS20	189	0.699
ENSP00000264515	RBBP5	14	0.699
ENSP00000290649	AMFR	189	0.699
ENSP00000237527	GHRH	1	0.7
ENSP00000358554	BCAS2	47	0.701

ENSP00000356480	RNF2	1	0.702
ENSP00000332258	DGAT1	2	0.703
ENSP00000264246	CD80	35	0.704
ENSP00000337088	MEN1	1555	0.705
ENSP00000269321	ARHGDIA	641	0.709
ENSP00000302967	HDAC3	524	0.709
ENSP00000219548	STUB1	1255	0.711
ENSP00000272190	REN	189	0.713
ENSP00000317955	EEA1	4	0.713
ENSP00000216797	NFKBIA	152	0.715
ENSP00000314813	OAZ1	185	0.715
ENSP00000360683	PTPN1	331	0.715
ENSP00000367439	RPP38	2	0.715
ENSP00000351273	CASP8	16	0.716
ENSP00000367910	FANCG	190	0.718
ENSP00000388526	HLA-A	205	0.718
ENSP00000311677	PPP1R8	763	0.719
ENSP00000216225	RBX1	1748	0.72
ENSP00000287727	ZFYVE9	679	0.72
ENSP00000303019	GPHN	190	0.722
ENSP00000360286	RAE1	380	0.722
ENSP00000252818	JUND	1555	0.723

ENSP00000253004	ASS1	7	0.723
ENSP00000358716	DDX20	191	0.724
ENSP00000297518	CDK5	748	0.726
ENSP00000260810	TOPBP1	1	0.728
ENSP00000262320	AXIN1	3303	0.729
ENSP00000367207	MYC	3382	0.73
ENSP00000264606	HDAC4	20	0.732
ENSP00000370023	HADHA	1	0.732
ENSP00000299022	LIPC	4	0.733
ENSP00000363313	PRPF4	10	0.733
ENSP00000265773	SMARCA2	5	0.734
ENSP00000311360	RAD9A	1	0.736
ENSP00000308533	GEMIN2	191	0.737
ENSP00000216194	ADSL	13	0.739
ENSP00000262735	PPARA	404	0.741
ENSP00000355493	ADSS	4	0.741
ENSP00000239223	DUSP1	53	0.742
ENSP00000342056	CS	6	0.742
ENSP00000263754	KAT2B	224	0.746
ENSP00000324806	GSK3B	2527	0.747
ENSP00000337759	DOM3Z	1	0.747
ENSP00000216223	IL2RB	276	0.748

ENSP00000314949	POLR2A	1822	0.748
ENSP00000356248	PTPN7	183	0.749
ENSP00000326550	TACC3	10	0.755
ENSP00000312697	DMAP1	183	0.758
ENSP00000309166	RBM4	11	0.759
ENSP00000355518	FH	5	0.761
ENSP00000233146	MSH2	191	0.765
ENSP00000251810	RRM2B	57	0.765
ENSP00000315791	CSTF3	191	0.766
ENSP00000340820	MAPT	742	0.767
ENSP00000390427	PPIL2	5	0.768
ENSP00000355759	PARP1	380	0.77
ENSP00000358622	IKBKG	191	0.772
ENSP00000271526	PRCC	5	0.781
ENSP00000278616	ATM	1594	0.781
ENSP00000350275	HIST1H3A	399	0.783
ENSP00000382177	MYO5A	2	0.785
ENSP00000348461	RAC1	4658	0.786
ENSP00000284981	APP	1698	0.787
ENSP00000384053	CSF2RB	275	0.787
ENSP00000419851	GMPS	355	0.788
ENSP00000217109	CSTF1	191	0.79

ENSP00000243776	CHPF	4	0.79
ENSP00000372975	HLA-C	207	0.791
ENSP00000316042	HNRNPA0	77	0.793
ENSP00000352516	DNMT1	211	0.796
ENSP00000375777	STRN4	189	0.797
ENSP00000352929	CSNK1E	172	0.799
ENSP00000383623	MLLT4	188	0.8
ENSP00000221413	RUVBL2	25	0.801
ENSP00000265564	EXOSC7	4	0.802
ENSP00000263253	EP300	9331	0.803
ENSP00000244741	CDKN1A	479	0.805
ENSP00000362361	CDK9	612	0.805
ENSP00000355890	EPRS	191	0.806
ENSP00000371973	SAP18	5	0.806
ENSP00000382595	PAICS	9	0.808
ENSP00000011653	CD4	2742	0.811
ENSP00000358595	CGA	191	0.816
ENSP00000301280	CHAF1A	764	0.817
ENSP00000303939	CTLA4	193	0.818
ENSP00000262238	YY1	461	0.819
ENSP00000306866	GABARAP	2	0.82
ENSP00000005257	RALA	193	0.822

ENSP00000320171	PKM	8	0.822
ENSP00000253856	ATP6V0A4	763	0.823
ENSP00000265838	ACAT1	1	0.823
ENSP00000317904	GYS1	380	0.823
ENSP00000245932	VASP	1	0.824
ENSP00000284384	PRKCA	368	0.824
ENSP00000313007	PABPC1	177	0.825
ENSP00000258962	SRSF1	132	0.826
ENSP00000339007	GRB2	6704	0.826
ENSP00000369497	BRCA2	373	0.827
ENSP00000209875	CBX5	382	0.829
ENSP00000385269	ELAVL1	18	0.832
ENSP00000256857	GRP	371	0.834
ENSP00000294117	GNG3	2	0.837
ENSP00000228872	CDKN1B	1096	0.843
ENSP00000329411	IRF7	8	0.843
ENSP00000343535	USP7	546	0.843
ENSP00000356087	IKBKE	121	0.852
ENSP00000260947	BARD1	191	0.853
ENSP00000290541	PSMB4	185	0.854
ENSP00000355261	SMG5	4	0.854
ENSP00000229239	GAPDH	379	0.862

ENSP00000362649	HDAC1	2737	0.865
ENSP00000355153	CDKN2A	192	0.87
ENSP00000334564	POLR3C	7	0.871
ENSP00000215071	PSMD8	4	0.873
ENSP00000363822	AR	782	0.874
ENSP00000225402	AATF	94	0.875
ENSP00000245414	IRF1	188	0.875
ENSP00000318297	RUVBL1	207	0.876
ENSP00000342793	PLD1	171	0.882
ENSP00000225916	KAT2A	8	0.884
ENSP00000274335	PIK3R1	1907	0.889
ENSP00000212015	SIRT1	187	0.89
ENSP00000285814	MKI67IP	2	0.891
ENSP00000300413	SNRPD1	191	0.891
ENSP00000324890	CD28	35	0.891
ENSP00000366013	GNB2L1	156	0.892
ENSP00000336790	ATF4	376	0.895
ENSP00000337825	LCK	3595	0.897
ENSP00000343204	JAK1	539	0.899
ENSP00000247668	TRAF2	2384	0.9
ENSP00000304845	UGT1A1	2	0.901
ENSP00000342215	KIR2DL3	2327	0.901

ENSP00000339109	ANAPC1	1162	0.905
ENSP00000262633	RBM42	15	0.906
ENSP00000296581	LSM6	1	0.911
ENSP00000300935	RAB8A	2	0.911
ENSP00000311032	CASP3	194	0.913
ENSP00000264414	CUL3	51	0.916
ENSP00000398597	EXOSC6	414	0.918
ENSP00000345571	E2F1	423	0.92
ENSP00000346437	ATG7	2	0.92
ENSP00000229769	FANCE	9	0.922
ENSP00000323050	RBBP8	135	0.922
ENSP00000391592	PTPN6	184	0.923
ENSP00000420168	GSTA2	1	0.923
ENSP00000274459	ATG12	2	0.924
ENSP00000344352	ATF3	376	0.924
ENSP00000358541	SIKE1	90	0.924
ENSP00000363868	ABCA1	8	0.924
ENSP00000273047	RAB5A	4	0.929
ENSP00000364649	SDHB	5	0.929
ENSP00000342952	ADCY2	5	0.93
ENSP00000310127	IRF3	109	0.931
ENSP00000260762	EXOC6	2	0.933

ENSP00000368880	FOXO1	7	0.934
ENSP00000407431	HLA-C	1956	0.934
ENSP00000248150	GNG13	1	0.935
ENSP00000298316	ARF6	777	0.937
ENSP00000340858	B2M	1983	0.939
ENSP00000325448	KARS	191	0.941
ENSP00000280665	DCP1B	48	0.945
ENSP00000344818	UBC	125114	0.945
ENSP00000353483	MAPK8	1148	0.945
ENSP00000249299	NAA38	93	0.946
ENSP00000396127	RAN	1668	0.946
ENSP00000299543	CTDP1	220	0.947
ENSP00000360798	EPS15	191	0.948
ENSP00000347733	TRRAP	4	0.953
ENSP00000248566	SHFM1	3828	0.954
ENSP00000348577	RANGAP1	1677	0.954
ENSP00000371236	GART	19	0.955
ENSP00000381339	GNAT3	2	0.957
ENSP00000343274	INTS8	97	0.958
ENSP00000260402	PLCB2	1	0.96
ENSP00000271628	SF3B4	5	0.96
ENSP00000287647	FANCD2	43	0.96

ENSP00000294172	NXF1	576	0.96
ENSP00000227758	BIRC2	188	0.961
ENSP00000369213	DDX58	190	0.962
ENSP00000352400	NUP214	561	0.963
ENSP00000267163	RB1	3371	0.964
ENSP00000354394	STAT1	1124	0.966
ENSP00000216605	MTHFD1	19	0.968
ENSP00000223029	AIMP2	191	0.968
ENSP00000215587	POLR2E	235	0.969
ENSP00000389934	EXOC5	2	0.969
ENSP00000362592	RBBP4	342	0.97
ENSP00000303088	POLR3D	7	0.971
ENSP00000223321	PSMA2	10	0.975
ENSP00000252102	NDUFA2	1954	0.975
ENSP00000266000	DAXX	16	0.975
ENSP00000342374	SNRPD2	27	0.976
ENSP00000354554	MT-CYB	383	0.976
ENSP00000361418	IPO13	69	0.977
ENSP00000364898	SYK	3894	0.979
ENSP00000301764	DDB1	98	0.98
ENSP00000354961	MT-ND4	382	0.981
ENSP00000217958	PSMD10	8	0.982

ENSP00000350512	COPS5	322	0.982
ENSP00000352980	HIST1H4A	304	0.983
ENSP00000359727	BAG2	138	0.983
ENSP00000262629	TYROBP	3334	0.984
ENSP00000312735	POLR2B	994	0.988
ENSP00000230449	EXOC2	2	0.989
ENSP00000333001	RBM8A	14	0.989
ENSP00000225577	RPS6KB1	4	0.991
ENSP00000254066	RARA	89	0.992
ENSP00000264951	XRN1	1557	0.992
ENSP00000326804	CUL1	25	0.992
ENSP00000397552	ACTL6A	5	0.992
ENSP00000162749	TNFRSF1A	127	0.994
ENSP00000235090	WDR77	1880	0.994
ENSP00000369757	RPS6	4	0.994
ENSP00000292644	PSMC2	5364	0.996
ENSP00000313199	HNRNPD	1	0.996
ENSP00000317159	CYC1	4	0.996
ENSP00000324897	UBE2I	1758	0.996
ENSP00000350877	SRSF2	12	0.996
ENSP00000357858	BUB3	1863	0.996
ENSP00000315644	TYMS	1395	0.997

ENSP00000252622	LSM7	524	0.998
ENSP00000262803	UPF1	4	0.998
ENSP00000268058	PML	75	0.998
ENSP00000315859	RNPS1	20	0.998
ENSP00000339151	IKBKB	1	0.998
ENSP00000240185	TARDBP	2557	0.999
ENSP00000316879	EIF4G1	260	0.999
ENSP00000341344	GGA1	4	0.999
ENSP00000349437	IGF2R	4	0.999
ENSP00000215829	SNRPD3	689	1
ENSP00000221494	SF3A2	990	1
ENSP00000227378	HSPA8	355	1
ENSP00000262477	RABEP1	4	1
ENSP00000263309	CLNS1A	573	1
ENSP00000269349	EIF4A3	73	1
ENSP00000274255	SKP2	758	1
ENSP00000276201	UPF3B	88	1
ENSP00000291552	U2AF1	18	1
ENSP00000296271	RHO	1507	1
ENSP00000307863	U2AF2	864	1
ENSP00000310596	LSM1	39	1
ENSP00000313829	KHDRBS1	2623	1

ENSP00000314491	SRRT	217	1
ENSP00000319169	PRMT5	573	1
ENSP00000348708	UPF2	220	1
ENSP00000358563	DKC1	183	1
ENSP00000361626	YBX1	10	1
ENSP00000362820	SRSF3	3	1
ENSP00000363676	RPL11	185	1
ENSP00000365439	HNRNPK	1103	1
ENSP00000366135	EXOSC10	1967	1
ENSP00000368438	PCNA	4323	1
ENSP00000374354	EXOSC8	2	1
ENSP00000377141	ARRB1	1660	1
ENSP00000414634	LSM2	177	1
ENSP00000417281	MDM2	2439	1

Supplementary Material III. Detailed information of candidate genes filtered by permutation test.

1. Candidate genes for methylation CpG site genes and microRNA target genes

Ensembl ID	Gene symbol	Betweenness ratio	Min-Max interaction score
ENSP00000264033	CBL	0.185028	200
ENSP00000344456	CTNNB1	0.091698	996
ENSP00000263253	EP300	0.08222	999
ENSP00000264657	STAT3	0.076427	814
ENSP00000306245	FOS	0.073582	940
ENSP00000352262	MLL	0.068489	988
ENSP00000252818	JUND	0.067808	324
ENSP00000337088	MEN1	0.067808	719
ENSP00000357656	FYN	0.055392	980
ENSP00000312652	LEP	0.03903	926
ENSP00000340858	B2M	0.037304	919
ENSP00000407431	HLA-C	0.0357	290
ENSP00000342215	KIR2DL3	0.0357	429
ENSP00000387662	GCG	0.031437	973
ENSP00000332353	PTCH1	0.028396	429
ENSP00000297261	SHH	0.026119	986
ENSP00000262965	TCF3	0.024729	985
ENSP00000351407	ARNT	0.024459	948

ENSP00000405890	PBX1	0.023591	822
ENSP00000361423	ABL1	0.019319	859
ENSP00000351209	EPHA2	0.018498	267
ENSP00000339328	PLAUR	0.016138	899
ENSP00000222725	LFNG	0.01611	504
ENSP00000300574	CRK	0.015121	644
ENSP00000365682	TLE1	0.014907	953
ENSP00000403005	EFNA4	0.014897	679
ENSP00000287934	FZD1	0.014879	967
ENSP00000358525	NGF	0.014664	943
ENSP00000354720	SMC3	0.01375	215
ENSP00000222005	CDC37	0.012659	0
ENSP00000318472	NCAM1	0.012407	906
ENSP00000258301	STX6	0.012397	237
ENSP00000249647	SNAP23	0.012164	299
ENSP00000331358	GAST	0.011978	899
ENSP00000244741	CDKN1A	0.011698	919
ENSP00000314491	SRRT	0.010858	199
ENSP00000170630	IL4R	0.010299	333
ENSP00000417132	BAP1	0.009916	227
ENSP00000312697	DMAP1	0.009916	0
ENSP00000351926	AP2A1	0.00986	181

ENSP00000293288	BAX	0.008974	906
ENSP00000354541	NLGN1	0.008797	629
ENSP00000257818	LMO2	0.008731	927
ENSP00000204615	THPO	0.008694	839
ENSP00000361548	MPL	0.008694	395
ENSP00000358497	RNGTT	0.008694	891
ENSP00000357283	LMNA	0.008694	848
ENSP00000386896	ITGA6	0.008694	372
ENSP00000304915	IL13	0.008675	957
ENSP00000236192	VAMP4	0.008675	0
ENSP00000200181	ITGB4	0.008591	0
ENSP00000262105	MCM4	0.008545	632
ENSP00000354901	CXCL9	0.008517	951
ENSP00000344460	CBS	0.008507	369
ENSP00000349275	NRG1	0.007631	810
ENSP00000250448	FOXA1	0.007621	470
ENSP00000314897	ANGPT2	0.007593	821
ENSP00000363827	HSPG2	0.007491	907
ENSP00000375629	LILRB2	0.007481	631
ENSP00000333188	FOXL2	0.007472	675
ENSP00000410294	FGFR2	0.007472	990
ENSP00000290295	HOXB13	0.007463	255

ENSP00000293362	PSME3	0.007463	364
ENSP00000348815	HYLS1	0.007463	260
ENSP00000376849	CASP5	0.007463	340
ENSP00000253571	RLIM	0.007463	907
ENSP00000256151	CCDC59	0.007463	473
ENSP00000355140	HOXB1	0.007463	470
ENSP00000291842	SHKBP1	0.007463	889
ENSP00000319118	GSX2	0.007463	507
ENSP00000331057	TCF12	0.007463	774
ENSP00000295600	MITF	0.007463	937
ENSP00000373700	ALK	0.007463	884
ENSP00000388996	AP1M1	0.007463	792
ENSP00000315997	LILRB1	0.007463	621
ENSP00000269280	NLRP1	0.007463	0
ENSP00000366347	NKX2-2	0.007463	788
ENSP00000247843	YEATS4	0.007463	921
ENSP00000392466	LDB1	0.007463	820
ENSP00000328181	NOG	0.007463	939
ENSP00000251337	GNAT2	0.007463	903
ENSP00000346879	NKX2-1	0.007463	429
ENSP00000333769	BSG	0.007463	412
ENSP00000075120	SLC2A3	0.007453	659

ENSP00000307479	ARNT2	0.007453	228
ENSP00000401435	VPS53	0.007453	817
ENSP00000370256	FOXC1	0.007453	429
ENSP00000328364	MAFA	0.007453	621
ENSP00000331791	TBX1	0.007453	700
ENSP00000358799	RBM15	0.007453	736
ENSP00000229307	NANOG	0.007453	824
ENSP00000371308	CENPJ	0.007453	504
ENSP00000318057	EGR3	0.007444	823
ENSP00000359215	TLX1	0.007444	654
ENSP00000310440	CHMP2A	0.007444	479
ENSP00000401018	GINS3	0.007444	531
ENSP00000405041	POU5F1	0.007444	659
ENSP00000356694	FASLG	0.007444	412
ENSP00000304414	CXCR6	0.007425	954
ENSP00000370521	AIPL1	0.007425	257
ENSP00000398644	NUB1	0.007425	0
ENSP00000355961	INTS7	0.007425	0
ENSP00000360076	SGIP1	0.007416	563
ENSP00000229030	FZD10	0.007416	871
ENSP00000341032	WNT7B	0.007416	875
ENSP00000298552	TSC1	0.007379	908

ENSP00000241125	GJA3	0.007351	657
ENSP00000006053	CX3CL1	0.007351	659
ENSP00000263431	PRKCG	0.007351	621
ENSP00000380066	MAP4K1	0.007351	0
ENSP00000382166	CX3CR1	0.007351	899
ENSP00000364092	ASIP	0.007323	267
ENSP00000354111	DNAJC5	0.007127	490
ENSP00000257829	NAT10	0.005336	305
ENSP00000316042	HNRNPA0	0.004664	0
ENSP00000335657	CCK	0.003713	916
ENSP00000260867	TIMM23	0.003666	0
ENSP00000345487	QRFP	0.00361	899
ENSP00000356162	KISS1	0.00361	933
ENSP00000276420	DOK2	0.002836	0
ENSP00000339151	IKBKB	0.002668	193
ENSP00000293831	EIF4A1	0.0025	186
ENSP00000269485	TNFRSF11A	0.0025	340
ENSP00000356623	CITED2	0.0025	905
ENSP00000347839	RAB11FIP2	0.0025	0
ENSP00000233057	EIF2AK2	0.0025	0
ENSP00000355566	TOMM20	0.002491	185
ENSP00000264499	BBS7	0.002491	0

ENSP00000360183	STX16	0.002491	341
ENSP00000354003	GYPA	0.002407	219
ENSP00000360217	RHAG	0.002407	371
ENSP00000254351	SDC1	0.002323	923
ENSP00000219473	USP10	0.002118	262
ENSP00000297338	RAD21	0.001838	430
ENSP00000263025	MAPK3	0.001576	359
ENSP00000264637	THRA	0.001493	903
ENSP00000336666	AP1S1	0.001427	191
ENSP00000357674	SNAPIN	0.001297	0
ENSP00000281821	EPHA4	0.001287	412
ENSP00000331514	ACTG1	0.001259	390
ENSP00000351141	WTAP	0.00125	625
ENSP00000321826	STXBP5	0.00125	200
ENSP00000360891	IFIT2	0.00125	0
ENSP00000222792	CHN2	0.00125	429
ENSP00000240652	IAPP	0.00125	922
ENSP00000339916	LIMK2	0.00125	161
ENSP00000347710	OPHN1	0.00125	180
ENSP00000349955	RPRD1A	0.00125	161
ENSP00000359910	PSMA7	0.00125	0
ENSP00000366603	TGOLN2	0.00125	0

ENSP00000230895	DAP	0.00125	0
ENSP00000247026	NSRP1	0.00125	0
ENSP00000339845	DROSHA	0.00125	219
ENSP00000231228	IL12B	0.00125	201
ENSP00000265529	KIF9	0.00125	0
ENSP00000274711	LRRTM2	0.00125	893
ENSP00000340688	LPHN1	0.00125	335
ENSP00000352673	ELF3	0.00125	340
ENSP00000284690	DHX32	0.00125	0
ENSP00000317872	RBBP6	0.00125	401
ENSP00000329715	DRG1	0.00125	392
ENSP00000346886	GABPA	0.00125	157
ENSP00000254691	CARD6	0.00125	0
ENSP00000265164	CASP6	0.00125	193
ENSP00000320838	GSG1	0.00125	154
ENSP00000334940	GGN	0.00125	176
ENSP00000350990	TNKS1BP1	0.00125	180
ENSP00000226091	EFNB3	0.00125	379
ENSP00000266085	TIMP3	0.00125	356
ENSP00000309622	TFDP2	0.00125	419
ENSP00000326432	CCR8	0.00125	932
ENSP00000362744	RPS4X	0.00125	171

ENSP00000406359	HSPA1A	0.00125	193
ENSP00000250111	ATP1B2	0.00125	156
ENSP00000254661	RAMP1	0.00125	188
ENSP00000283977	PGM3	0.00125	256
ENSP00000315173	ZNF41	0.00125	0
ENSP00000337194	PRPF4B	0.00125	0
ENSP00000344871	MYO1F	0.00125	295
ENSP00000308549	ADORA1	0.00125	902
ENSP00000394033	KCNK2	0.00125	180
ENSP00000263209	DGCR8	0.00125	281
ENSP00000275603	CCT6A	0.00125	316
ENSP00000307549	NPTX1	0.00125	851
ENSP00000340507	TRIM24	0.00125	229
ENSP00000365766	TIMM17B	0.00125	0
ENSP00000351363	MSMB	0.00125	203
ENSP00000357692	S100A16	0.00125	0
ENSP00000354033	PCGF2	0.00125	377
ENSP00000385021	FANCL	0.00125	238
ENSP00000225893	HNF1B	0.00125	651
ENSP00000297562	AP5Z1	0.00125	0
ENSP00000364398	HABP4	0.00125	265
ENSP00000403557	PPP1R11	0.00125	899

ENSP00000216410	GNPNAT1	0.00125	0
ENSP00000404232	EFHC2	0.00125	219
ENSP00000219244	CCL17	0.00125	524
ENSP00000250894	MAPK8IP3	0.00125	0
ENSP00000309968	ADAM17	0.00125	556
ENSP00000313581	KLK2	0.00125	0
ENSP00000334594	SLC10A7	0.00125	0
ENSP00000262053	ATF1	0.00125	500
ENSP00000294053	CLPB	0.00125	0
ENSP00000299163	HIF1AN	0.00125	0
ENSP00000359174	SLC35A3	0.00125	0
ENSP00000386171	ESRRG	0.00125	902
ENSP00000303423	FNTA	0.00125	0
ENSP00000357311	CENPW	0.00125	458
ENSP00000388241	KIF26A	0.00125	906
ENSP00000272233	RHOB	0.00125	252
ENSP00000260950	MSTN	0.00125	847
ENSP00000346148	PRKAA1	0.00125	972
ENSP00000266744	ASCL1	0.00125	461
ENSP00000311579	TNKS	0.00125	401
ENSP00000346440	TCF4	0.00125	885
ENSP00000354376	RAB25	0.00125	184

ENSP00000278198	LRRC4C	0.00125	252
ENSP00000315167	ALOX12B	0.00125	0
ENSP00000356529	RGS16	0.00125	0
ENSP00000357244	CCT3	0.00125	317
ENSP00000361473	KDM4A	0.00125	0
ENSP00000335632	CHP1	0.00125	0
ENSP00000281928	MED13L	0.00125	0
ENSP00000305913	COL8A2	0.00125	905
ENSP00000328216	ORAI1	0.00125	0
ENSP00000240874	KALRN	0.00125	659
ENSP00000371475	TP53BP1	0.00125	0
ENSP00000385057	APOBEC3G	0.00125	0
ENSP00000304102	COPS6	0.00125	0
ENSP00000419923	KLF6	0.00125	196
ENSP00000291582	AIRE	0.00125	263
ENSP00000365811	SPAG6	0.00125	260
ENSP00000168216	HSD17B10	0.00125	0
ENSP00000326830	CLK1	0.00125	0
ENSP00000219172	CENPT	0.00125	0
ENSP00000307939	GCC2	0.00125	188
ENSP00000322229	FADS1	0.00125	0
ENSP00000357748	BCCIP	0.00125	184

ENSP00000366819	UCHL3	0.00125	0
ENSP00000382791	GRIK1	0.00125	649
ENSP00000337722	ARL6	0.00125	207
ENSP00000396439	RING1	0.00125	288
ENSP00000252997	GATA5	0.00125	908
ENSP00000258774	HUS1	0.00125	231
ENSP00000338207	LMO1	0.00125	584
ENSP00000414237	INTS2	0.00125	0
ENSP00000320567	MRPS33	0.00125	0
ENSP00000352522	ATP6V1H	0.00125	0
ENSP00000326261	SRRM1	0.00125	389
ENSP00000296490	WDR82	0.00125	0
ENSP00000352708	TRAPPC2	0.00125	243
ENSP00000263205	MED15	0.00125	183
ENSP00000345728	ATP7A	0.00125	569
ENSP00000281950	GEMIN6	0.00125	462
ENSP00000261636	ARL1	0.00125	513
ENSP00000265351	XPO5	0.00125	290
ENSP00000297373	PHKG1	0.00125	206
ENSP00000378288	MYLK3	0.00125	248
ENSP00000284154	GRAP	0.001241	252
ENSP00000305595	B3GNT2	0.001241	0

ENSP00000341848	GOLGB1	0.001241	248
ENSP00000293441	SHANK1	0.001241	632
ENSP00000299339	CLDN10	0.001241	0
ENSP00000354777	TBKBP1	0.001241	0
ENSP00000228641	MYF6	0.001241	721
ENSP00000256689	SLC38A2	0.001231	168
ENSP00000367747	PLCH2	0.001231	245
ENSP00000218652	NDFIP2	0.001231	154
ENSP00000273398	ATP6V1A	0.001222	390
ENSP00000285398	ERCC3	0.001222	404
ENSP00000246194	RALY	0.001213	0
ENSP00000287322	BAG4	0.001185	157
ENSP00000310723	DDX23	0.001129	197
ENSP00000255688	RARRES3	0.001082	220
ENSP00000344822	S100A13	0.000112	0
ENSP00000248272	GAN	9.33E-05	569
ENSP00000357453	MAN1A1	6.53E-05	191
ENSP00000293897	SSTR5	5.6E-05	900
ENSP00000367959	HTR2A	4.66E-05	908
ENSP00000294309	TPCN2	3.73E-05	412
ENSP00000220478	SCG3	3.73E-05	241
ENSP00000318128	BLOC1S4	3.73E-05	374

ENSP00000384169	FBLN2	2.8E-05	529
ENSP00000362555	RNF19B	2.8E-05	227
ENSP00000384179	ZFPM2	1.87E-05	752
ENSP00000221855	TBCB	1.87E-05	0
ENSP00000345752	MTMR2	1.87E-05	195
ENSP00000381634	SLC38A1	1.87E-05	190
ENSP00000302397	ATP1A3	1.87E-05	265
ENSP00000383263	CCHCR1	1.87E-05	0
ENSP00000259216	CFC1	9.33E-06	210
ENSP00000265713	KAT6A	9.33E-06	719
ENSP00000320081	C3orf58	9.33E-06	778
ENSP00000376268	SEC14L1	9.33E-06	231
ENSP00000376350	TPCN1	9.33E-06	219
ENSP00000341550	SLC24A5	9.33E-06	379
ENSP00000355927	RPS6KC1	9.33E-06	0
ENSP00000337675	EBAG9	9.33E-06	237
ENSP00000340328	NYX	9.33E-06	302
ENSP00000382895	RPGRIP1	9.33E-06	619
ENSP00000343943	PPOX	9.33E-06	0
ENSP00000264193	CPOX	9.33E-06	324
ENSP00000290597	ALDH4A1	9.33E-06	203
ENSP00000282903	PLOD2	9.33E-06	279

ENSP00000274353	BHMT	9.33E-06	529
ENSP00000329684	GALR2	9.33E-06	899
ENSP00000184266	NDUFB4	9.33E-06	235
ENSP00000355180	COL6A1	9.33E-06	905
ENSP00000317379	GLS	9.33E-06	369
ENSP00000349708	ZMYM6	9.33E-06	899

2. Candidate genes for methylation CpG site genes and somatic mutation genes

Ensembl ID	Gene symbol	Betweenness ratio	Min-Max interaction score
ENSP00000264033	CBL	0.194381	200
ENSP00000344456	CTNNB1	0.126592	996
ENSP00000206249	ESR1	0.12163	967
ENSP00000338018	HIF1A	0.102524	241
ENSP00000350941	SRC	0.076776	930
ENSP00000264657	STAT3	0.074158	814
ENSP00000227507	CCND1	0.070837	844
ENSP00000357656	FYN	0.07029	980
ENSP00000306245	FOS	0.069938	940
ENSP00000352262	MLL	0.061772	571
ENSP00000252818	JUND	0.061225	324
ENSP00000337088	MEN1	0.061225	719
ENSP00000277541	NOTCH1	0.044073	925
ENSP00000293379	ITGA5	0.042901	262

ENSP00000312652	LEP	0.041572	866
ENSP00000302665	IGF1	0.039462	808
ENSP00000401303	SHC1	0.038134	0
ENSP00000340858	B2M	0.037235	826
ENSP00000332353	PTCH1	0.036376	429
ENSP00000387662	GCG	0.035868	929
ENSP00000351486	NTRK1	0.035672	287
ENSP00000407431	HLA-C	0.034149	317
ENSP00000358525	NGF	0.031804	953
ENSP00000296585	ITGA2	0.030945	565
ENSP00000308938	PLG	0.029929	427
ENSP00000361125	VEGFA	0.028444	818
ENSP00000297261	SHH	0.027585	985
ENSP00000312435	DAG1	0.026022	338
ENSP00000351209	EPHA2	0.025006	267
ENSP00000405890	PBX1	0.022388	271
ENSP00000262965	TCF3	0.02231	420
ENSP00000265335	RAD50	0.022271	0
ENSP00000351407	ARNT	0.02188	229
ENSP00000242577	DYNLL1	0.021255	621
ENSP00000304669	CTNNA1	0.020942	0
ENSP00000371138	FKBP1A	0.020708	271

ENSP00000359074	L1CAM	0.020591	829
ENSP00000264708	POMC	0.019966	917
ENSP00000261769	CDH1	0.019731	825
ENSP00000162330	BCAR1	0.01895	593
ENSP00000254942	TERF2	0.017817	0
ENSP00000332973	SMAD3	0.017817	695
ENSP00000300574	CRK	0.017035	644
ENSP00000300134	STAT6	0.01684	878
ENSP00000331358	GAST	0.016371	899
ENSP00000297268	COL1A2	0.015629	904
ENSP00000265709	ANK1	0.015551	390
ENSP00000222725	LFNG	0.014925	750
ENSP00000403005	EFNA4	0.014925	679
ENSP00000287934	FZD1	0.014925	813
ENSP00000365682	TLE1	0.014925	270
ENSP00000339328	PLAUR	0.014925	899
ENSP00000228307	PXN	0.013558	330
ENSP00000301838	FADD	0.012542	508
ENSP00000254950	VPS4A	0.012503	0
ENSP00000386896	ITGA6	0.012386	372
ENSP00000354541	NLGN1	0.012308	621
ENSP00000200181	ITGB4	0.012229	0

ENSP00000254480	SMARCC1	0.011409	734
ENSP00000293288	BAX	0.010627	906
ENSP00000322898	EBF1	0.010471	959
ENSP00000268171	FURIN	0.010471	723
ENSP00000276603	TERF1	0.010315	277
ENSP00000361850	PLAU	0.010198	885
ENSP00000330633	CNTN2	0.010002	243
ENSP00000340698	GIPC1	0.009885	930
ENSP00000281708	FBXW7	0.008674	195
ENSP00000254351	SDC1	0.007775	923
ENSP00000260130	SDCBP	0.007736	179
ENSP00000257818	LMO2	0.007619	379
ENSP00000267415	TINF2	0.007619	277
ENSP00000229307	NANOG	0.007502	824
ENSP00000265773	SMARCA2	0.007502	621
ENSP00000075120	SLC2A3	0.007463	340
ENSP00000204615	THPO	0.007463	368
ENSP00000253571	RLIM	0.007463	329
ENSP00000256151	CCDC59	0.007463	312
ENSP00000290295	HOXB13	0.007463	302
ENSP00000293362	PSME3	0.007463	364
ENSP00000295600	MITF	0.007463	509

ENSP00000315997	LILRB1	0.007463	191
ENSP00000328364	MAFA	0.007463	195
ENSP00000333188	FOXL2	0.007463	216
ENSP00000355140	HOXB1	0.007463	506
ENSP00000358497	RNGTT	0.007463	347
ENSP00000373700	ALK	0.007463	705
ENSP00000376849	CASP5	0.007463	340
ENSP00000401435	VPS53	0.007463	0
ENSP00000247843	YEATS4	0.007463	307
ENSP00000291842	SHKBP1	0.007463	294
ENSP00000319118	GSX2	0.007463	364
ENSP00000359215	TLX1	0.007463	311
ENSP00000361548	MPL	0.007463	373
ENSP00000375629	LILRB2	0.007463	513
ENSP00000307479	ARNT2	0.007463	0
ENSP00000331791	TBX1	0.007463	700
ENSP00000348815	HYLS1	0.007463	317
ENSP00000388996	AP1M1	0.007463	619
ENSP00000331057	TCF12	0.007463	269
ENSP00000363827	HSPG2	0.007463	907
ENSP00000229030	FZD10	0.007463	802
ENSP00000269280	NLRP1	0.007463	0

ENSP00000314897	ANGPT2	0.007463	340
ENSP00000366347	NKX2-2	0.007463	304
ENSP00000304915	IL13	0.007463	900
ENSP00000328181	NOG	0.007463	207
ENSP00000349275	NRG1	0.007463	810
ENSP00000355961	INTS7	0.007463	0
ENSP00000371308	CENPJ	0.007463	504
ENSP00000392466	LDB1	0.007463	366
ENSP00000405041	POU5F1	0.007463	537
ENSP00000341032	WNT7B	0.007463	346
ENSP00000251337	GNAT2	0.007463	899
ENSP00000236192	VAMP4	0.007463	0
ENSP00000417132	BAP1	0.007463	227
ENSP00000410294	FGFR2	0.007463	536
ENSP00000346879	NKX2-1	0.007463	373
ENSP00000357283	LMNA	0.007463	848
ENSP00000333769	BSG	0.007463	340
ENSP00000250448	FOXA1	0.007463	470
ENSP00000312697	DMAP1	0.007463	0
ENSP00000258301	STX6	0.007463	193
ENSP00000360076	SGIP1	0.007424	501
ENSP00000370521	AIPL1	0.007424	659

ENSP00000318057	EGR3	0.007424	338
ENSP00000304414	CXCR6	0.007424	899
ENSP00000398644	NUB1	0.007424	0
ENSP00000358799	RBM15	0.007424	736
ENSP00000356694	FASLG	0.007424	412
ENSP00000401018	GINS3	0.007385	426
ENSP00000310440	CHMP2A	0.007385	479
ENSP00000370256	FOXC1	0.007345	317
ENSP00000344460	CBS	0.007345	341
ENSP00000380066	MAP4K1	0.007345	0
ENSP00000298552	TSC1	0.007306	542
ENSP00000364092	ASIP	0.007267	229
ENSP00000006053	CX3CL1	0.007267	659
ENSP00000382166	CX3CR1	0.007267	466
ENSP00000241125	GJA3	0.00715	0
ENSP00000354111	DNAJC5	0.00715	227
ENSP00000263431	PRKCG	0.00715	621
ENSP00000308176	BTK	0.006681	333
ENSP00000257829	NAT10	0.005587	305
ENSP00000341170	PTN	0.005275	288
ENSP00000382193	MYBPC3	0.005275	183
ENSP00000286621	ADK	0.005236	193

ENSP00000252456	CNN1	0.005236	229
ENSP00000372313	MSLN	0.005236	340
ENSP00000383210	NEK3	0.005236	257
ENSP00000303522	TACR1	0.005236	899
ENSP00000265071	CDH6	0.005236	228
ENSP00000348634	MYH6	0.005236	914
ENSP00000352657	ME3	0.005236	0
ENSP00000385450	MAGI1	0.005236	0
ENSP00000372160	DOK6	0.005236	159
ENSP00000242839	ATP7B	0.005236	677
ENSP00000363360	INIP	0.005236	202
ENSP00000379457	FAF1	0.005236	214
ENSP00000225893	HNF1B	0.005236	651
ENSP00000309595	C10orf2	0.005236	800
ENSP00000262053	ATF1	0.005236	180
ENSP00000344468	SDC3	0.005236	899
ENSP00000367486	MEIG1	0.005236	0
ENSP00000233813	IGFBP5	0.005236	471
ENSP00000367714	HES5	0.005236	569
ENSP00000312185	ELMO1	0.005236	196
ENSP00000265362	SEMA3A	0.005236	947
ENSP00000372547	SRY	0.005236	444

ENSP00000370408	CDX2	0.005236	655
ENSP00000344742	STAMBP	0.005236	0
ENSP00000258341	LAMC1	0.005236	807
ENSP00000361777	SET	0.005236	427
ENSP00000245323	EFNB2	0.005236	644
ENSP00000380349	CAPN3	0.005236	289
ENSP00000337397	DKFZP686J19100	0.005236	0
ENSP00000343479	NBR1	0.005236	0
ENSP00000356641	RFWD2	0.005197	375
ENSP00000360200	INADL	0.005197	285
ENSP00000295709	STK36	0.005197	213
ENSP00000291525	TFF3	0.005197	372
ENSP00000339861	ENY2	0.005197	0
ENSP00000360689	TNKS2	0.005157	424
ENSP00000222399	LAMB1	0.005118	456
ENSP00000348168	GTF2E2	0.005118	290
ENSP00000267845	HDC	0.005118	513
ENSP00000261681	MPP5	0.005118	247
ENSP00000348089	ERCC6	0.00504	369
ENSP00000338785	STARD13	0.004767	0
ENSP00000345487	QRFP	0.003321	899
ENSP00000356162	KISS1	0.003321	899

ENSP00000276420	DOK2	0.00211	0
ENSP00000219473	USP10	0.001875	262
ENSP00000264637	THRA	0.00168	424
ENSP00000336666	AP1S1	0.001563	0
ENSP00000260356	THBS1	0.00086	814
ENSP00000303634	LRP8	0.000547	306
ENSP00000296785	ANKRA2	0.000547	183
ENSP00000310244	RASGRP1	0.000469	201
ENSP00000257068	MTNR1B	0.000313	899
ENSP00000257254	APLNR	0.000313	899
ENSP00000364839	ASXL1	0.000313	317
ENSP00000260570	IFT172	0.000274	340
ENSP00000361759	BEX2	0.000195	160
ENSP00000419361	ADCY5	0.000156	904
ENSP00000293897	SSTR5	0.000117	899
ENSP00000263281	GIPR	0.000117	905
ENSP00000344822	S100A13	0.000117	0
ENSP00000303325	TACR3	0.000117	899
ENSP00000221855	TBCB	7.81E-05	0
ENSP00000248272	GAN	7.81E-05	372
ENSP00000294309	TPCN2	7.81E-05	319
ENSP00000325123	ZSCAN2	7.81E-05	490

ENSP00000364946	MKX	7.81E-05	340
ENSP00000362555	RNF19B	7.81E-05	290
ENSP00000360882	COL5A1	7.81E-05	905
ENSP00000262968	TJP3	7.81E-05	0
ENSP00000346155	UCKL1	7.81E-05	0
ENSP00000304188	OR8U1	7.81E-05	0
ENSP00000326630	ZFPM1	7.81E-05	344
ENSP00000334044	UBL4B	3.91E-05	798
ENSP00000335038	VSTM2B	3.91E-05	412
ENSP00000020926	SYT13	3.91E-05	263
ENSP00000400223	FJX1	3.91E-05	0
ENSP00000341550	SLC24A5	3.91E-05	369
ENSP00000359245	ABCA4	3.91E-05	516
ENSP00000330032	UPP1	3.91E-05	0
ENSP00000263856	CHMP3	3.91E-05	157
ENSP00000274711	LRRTM2	3.91E-05	245
ENSP00000397181	RGS4	3.91E-05	746
ENSP00000400258	DLGAP2	3.91E-05	363
ENSP00000355180	COL6A1	3.91E-05	905
ENSP00000369654	HBD	3.91E-05	235
ENSP00000248933	SEZ6L	3.91E-05	299
ENSP00000299413	TRIM44	3.91E-05	0

ENSP00000222982	CYP3A5	3.91E-05	506
ENSP00000361311	TMEM53	3.91E-05	899
ENSP00000264039	GPC1	3.91E-05	902
ENSP00000411286	GABBR1	3.91E-05	899
ENSP00000313921	MSRA	3.91E-05	429
ENSP00000305464	APLN	3.91E-05	899
ENSP00000019103	SCTR	3.91E-05	906
ENSP00000171214	RDH8	3.91E-05	249

3. Candidate genes for methylation CpG site genes and mRNA genes

Ensembl ID	Gene symbol	Betweenness ratio	Min-Max interaction score
ENSP00000264033	CBL	0.208924	200
ENSP00000338018	HIF1A	0.100632	241
ENSP00000344456	CTNNB1	0.099876	996
ENSP00000264657	STAT3	0.089761	814
ENSP00000263253	EP300	0.086931	995
ENSP00000306245	FOS	0.084446	940
ENSP00000227507	CCND1	0.068991	844
ENSP00000352262	MLL	0.060034	567
ENSP00000252818	JUND	0.059972	324
ENSP00000337088	MEN1	0.059972	719
ENSP00000357656	FYN	0.059215	980
ENSP00000312652	LEP	0.046025	926
ENSP00000340858	B2M	0.045397	922
ENSP00000342215	KIR2DL3	0.044149	424
ENSP00000407431	HLA-C	0.042629	550
ENSP00000262320	AXIN1	0.035351	852
ENSP00000387662	GCG	0.033942	939
ENSP00000332353	PTCH1	0.032299	429

ENSP00000293379	ITGA5	0.031266	262
ENSP00000297261	SHH	0.02942	995
ENSP00000351407	ARNT	0.029359	976
ENSP00000262965	TCF3	0.026295	987
ENSP00000405890	PBX1	0.023963	859
ENSP00000361125	VEGFA	0.02376	818
ENSP00000264708	POMC	0.023545	977
ENSP00000348986	INS-IGF2	0.023132	942
ENSP00000303830	INSR	0.021834	526
ENSP00000340944	PTPN11	0.01893	643
ENSP00000351209	EPHA2	0.018863	267
ENSP00000222725	LFNG	0.017239	750
ENSP00000365682	TLE1	0.016568	683
ENSP00000339328	PLAUR	0.016531	899
ENSP00000287934	FZD1	0.015694	978
ENSP00000162330	BCAR1	0.013609	654
ENSP00000261769	CDH1	0.013037	825
ENSP00000320866	CALR	0.012372	430
ENSP00000228837	FGF6	0.012237	424
ENSP00000242152	NPY	0.011911	984
ENSP00000331358	GAST	0.011757	947
ENSP00000262186	KCNH2	0.011652	953
ENSP00000380227	ITGA4	0.011252	308
ENSP00000354720	SMC3	0.010982	215
ENSP00000410294	FGFR2	0.010766	999
ENSP00000410076	CASP1	0.010674	246
ENSP00000354541	NLGN1	0.010582	540
ENSP00000368683	EDN1	0.010551	987
ENSP00000338345	SNCA	0.010496	828
ENSP00000157812	PSMC4	0.010453	0
ENSP00000346879	NKX2-1	0.009911	429
ENSP00000318472	NCAM1	0.009893	906
ENSP00000250448	FOXA1	0.009868	470

ENSP00000262105	MCM4	0.009536	632
ENSP00000358866	FLNA	0.00929	908
ENSP00000328181	NOG	0.009099	924
ENSP00000334458	GATA4	0.009038	933
ENSP00000225831	CCL2	0.00897	637
ENSP00000006053	CX3CL1	0.008755	659
ENSP00000382166	CX3CR1	0.008755	885
ENSP00000405041	POU5F1	0.008718	659
ENSP00000170630	IL4R	0.008644	333
ENSP00000314897	ANGPT2	0.008515	821
ENSP00000257818	LMO2	0.008355	972
ENSP00000253571	RLIM	0.008281	904
ENSP00000355140	HOXB1	0.008275	804
ENSP00000392466	LDB1	0.008275	820
ENSP00000357283	LMNA	0.008275	435
ENSP00000333769	BSG	0.008275	952
ENSP00000328364	MAFA	0.008269	374
ENSP00000304915	IL13	0.008269	854
ENSP00000401018	GINS3	0.008256	531
ENSP00000355961	INTS7	0.008244	0
ENSP00000229030	FZD10	0.008244	887
ENSP00000358497	RNGTT	0.008244	722
ENSP00000251337	GNAT2	0.008244	829
ENSP00000341032	WNT7B	0.008238	875
ENSP00000366347	NKX2-2	0.008232	518
ENSP00000269280	NLRP1	0.008226	0
ENSP00000344460	CBS	0.008109	369
ENSP00000263431	PRKCG	0.008109	621
ENSP00000200181	ITGB4	0.008084	0
ENSP00000354901	CXCL9	0.008066	951
ENSP00000293288	BAX	0.007955	906
ENSP00000229307	NANOG	0.007776	824
ENSP00000375629	LILRB2	0.007598	800

ENSP00000349275	NRG1	0.007537	810
ENSP00000363827	HSPG2	0.007518	907
ENSP00000204615	THPO	0.007463	878
ENSP00000333188	FOXL2	0.007463	836
ENSP00000307479	ARNT2	0.007463	770
ENSP00000373700	ALK	0.007463	769
ENSP00000388996	AP1M1	0.007463	792
ENSP00000291842	SHKBP1	0.007463	385
ENSP00000361548	MPL	0.007463	395
ENSP00000315997	LILRB1	0.007457	800
ENSP00000358799	RBM15	0.007457	736
ENSP00000256151	CCDC59	0.007457	340
ENSP00000290295	HOXB13	0.007457	837
ENSP00000319118	GSX2	0.007457	908
ENSP00000331057	TCF12	0.007457	774
ENSP00000247843	YEATS4	0.007457	822
ENSP00000318057	EGR3	0.00745	791
ENSP00000401435	VPS53	0.00745	401
ENSP00000310440	CHMP2A	0.00745	306
ENSP00000331791	TBX1	0.00745	551
ENSP00000293362	PSME3	0.00745	364
ENSP00000370521	AIPL1	0.007444	527
ENSP00000348815	HYLS1	0.007444	891
ENSP00000398644	NUB1	0.007444	0
ENSP00000295600	MITF	0.007444	912
ENSP00000371308	CENPJ	0.007444	504
ENSP00000370256	FOXC1	0.007438	649
ENSP00000356694	FASLG	0.007438	412
ENSP00000236192	VAMP4	0.007438	0
ENSP00000075120	SLC2A3	0.007432	407
ENSP00000359215	TLX1	0.00742	572
ENSP00000376849	CASP5	0.007413	340
ENSP00000380066	MAP4K1	0.007407	0

ENSP00000360076	SGIP1	0.007383	563
ENSP00000304414	CXCR6	0.007377	946
ENSP00000298552	TSC1	0.00737	687
ENSP00000364092	ASIP	0.007358	659
ENSP00000241125	GJA3	0.00729	625
ENSP00000354111	DNAJC5	0.007118	933
ENSP00000257829	NAT10	0.005722	305
ENSP00000276420	DOK2	0.004196	0
ENSP00000345487	QRFP	0.003618	938
ENSP00000356162	KISS1	0.003611	933
ENSP00000335544	CCKBR	0.003599	902
ENSP00000264998	TF	0.002719	530
ENSP00000219473	USP10	0.002553	262
ENSP00000254351	SDC1	0.002504	923
ENSP00000372815	C4A	0.002473	340
ENSP00000201586	SULT2B1	0.002473	266
ENSP00000161559	CEACAM1	0.002473	163
ENSP00000281821	EPHA4	0.002473	412
ENSP00000356789	ATP1B1	0.002473	152
ENSP00000383690	MASP2	0.002473	0
ENSP00000291386	SSU72	0.002455	0
ENSP00000359285	CHRNA4	0.002442	800
ENSP00000386165	CEBPD	0.002399	435
ENSP00000264637	THRA	0.001907	903
ENSP00000019103	SCTR	0.001796	932
ENSP00000316244	HTR1A	0.00168	938
ENSP00000304767	P2RY1	0.001661	904
ENSP00000354130	SOX10	0.001655	940
ENSP00000199764	CEACAM6	0.001649	0
ENSP00000393887	AHSG	0.001649	311
ENSP00000328968	SCN5A	0.001649	227
ENSP00000231524	TRIM23	0.001649	0
ENSP00000264234	UPK1B	0.001649	0

ENSP00000283147	BMP6	0.001649	266
ENSP00000234739	BCL9	0.001649	240
ENSP00000262776	LGALS3BP	0.001649	0
ENSP00000247170	DAAM1	0.001649	167
ENSP00000264126	GPSM2	0.001649	542
ENSP00000350162	SYCP2	0.001649	0
ENSP00000005226	USH1C	0.001649	0
ENSP00000353408	MSN	0.001649	343
ENSP00000217381	SNTA1	0.001649	218
ENSP00000340088	THEG	0.001649	0
ENSP00000372991	LTA	0.001649	0
ENSP00000280193	VEGFC	0.001649	289
ENSP00000305714	BMP1	0.001649	589
ENSP00000356623	CITED2	0.001649	905
ENSP00000254301	LGALS3	0.001649	174
ENSP00000331902	COL4A5	0.001643	905
ENSP00000323568	SLC2A2	0.001643	844
ENSP00000261037	COL8A1	0.001643	905
ENSP00000295709	STK36	0.001637	226
ENSP00000353654	COL4A2	0.001637	903
ENSP00000272238	ATP6V1C2	0.001637	0
ENSP00000350256	CCR9	0.001624	829
ENSP00000225844	CCL13	0.001624	460
ENSP00000413720	CDKN1C	0.001618	940
ENSP00000248996	GNAZ	0.001618	800
ENSP00000323280	CD6	0.001612	0
ENSP00000305988	ALCAM	0.001612	0
ENSP00000357721	S100A8	0.001612	249
ENSP00000260630	CYP1B1	0.001587	800
ENSP00000396915	SCN1B	0.001507	675
ENSP00000241256	GHSR	0.001483	938
ENSP00000336666	AP1S1	0.001464	191
ENSP00000265969	KCNC1	0.001064	955

ENSP00000260570	IFT172	0.000972	508
ENSP00000239223	DUSP1	0.000954	398
ENSP00000168712	FGF4	0.00088	610
ENSP00000309913	TBX5	0.000843	641
ENSP00000292169	S100A1	0.000831	0
ENSP00000268704	SPG7	0.000824	401
ENSP00000222792	CHN2	0.000824	429
ENSP00000233616	MOGS	0.000824	226
ENSP00000362372	BRWD3	0.000824	201
ENSP00000377840	CACNB1	0.000824	0
ENSP00000252593	BST2	0.000824	0
ENSP00000264009	HSF4	0.000824	358
ENSP00000357025	CD48	0.000824	219
ENSP00000005587	SKAP2	0.000824	0
ENSP00000266659	GLIPR1	0.000824	0
ENSP00000310216	KLRC4	0.000824	625
ENSP00000318406	KIAA0319L	0.000824	200
ENSP00000392828	GPSM1	0.000824	200
ENSP00000221283	STXBP2	0.000824	0
ENSP00000252050	CUL9	0.000824	0
ENSP00000295728	CRYBA2	0.000824	347
ENSP00000301420	KLK1	0.000824	218
ENSP00000333920	TTF1	0.000824	0
ENSP00000352442	HIST1H2BM	0.000824	0
ENSP00000364246	PLA2G2D	0.000824	180
ENSP00000365877	SUV39H1	0.000824	663
ENSP00000369009	CXorf23	0.000824	0
ENSP00000373485	TSNAXIP1	0.000824	0
ENSP00000233202	SLC11A1	0.000824	203
ENSP00000248071	KLF2	0.000824	308
ENSP00000261182	NAP1L1	0.000824	265
ENSP00000263369	MIA	0.000824	200
ENSP00000283871	HGD	0.000824	260

ENSP00000300417	LRSAM1	0.000824	219
ENSP00000338728	CCDC88A	0.000824	198
ENSP00000350332	MYBPC2	0.000824	185
ENSP00000358576	DCLRE1B	0.000824	173
ENSP00000395465	NCOA4	0.000824	0
ENSP00000245539	MRPS7	0.000824	228
ENSP00000265294	GABRP	0.000824	167
ENSP00000297107	GALNT10	0.000824	0
ENSP00000355001	POU3F3	0.000824	336
ENSP00000356906	SH2D1B	0.000824	0
ENSP00000281030	THRSP	0.000824	585
ENSP00000298386	RXFP2	0.000824	899
ENSP00000352785	DSG4	0.000824	0
ENSP00000359594	CLCA4	0.000824	800
ENSP00000307218	NAT1	0.000824	341
ENSP00000354900	GJB1	0.000824	269
ENSP00000361867	SEMG1	0.000824	160
ENSP00000368450	CD83	0.000824	379
ENSP00000374981	IGHA2	0.000824	522
ENSP00000260600	ADCY3	0.000824	816
ENSP00000261170	GUCY2C	0.000824	540
ENSP00000274793	PLA2G7	0.000824	340
ENSP00000297135	COG5	0.000824	0
ENSP00000315173	ZNF41	0.000824	0
ENSP00000340396	GBP5	0.000824	207
ENSP00000343819	OTX2	0.000824	763
ENSP00000226091	EFNB3	0.000824	379
ENSP00000298032	ARMC3	0.000824	0
ENSP00000302114	PRELID1	0.000824	155
ENSP00000219281	USB1	0.000824	0
ENSP00000266085	TIMP3	0.000824	356
ENSP00000329968	PHKG2	0.000824	206
ENSP00000354033	PCGF2	0.000824	377

ENSP00000355896	TGFB2	0.000824	800
ENSP00000414598	MRVI1	0.000824	334
ENSP00000225941	ABI3	0.000824	168
ENSP00000274031	SETD7	0.000824	394
ENSP00000278823	MTA2	0.000824	219
ENSP00000343785	SPRY1	0.000824	860
ENSP00000361658	NUP188	0.000824	0
ENSP00000408395	RBFOX3	0.000824	613
ENSP00000216373	SOS2	0.000824	0
ENSP00000262545	PCSK2	0.000824	463
ENSP00000267859	BNIP2	0.000824	228
ENSP00000276072	TAF1	0.000824	619
ENSP00000294973	HAAO	0.000824	257
ENSP00000316990	TRAPPC5	0.000824	0
ENSP00000206474	HAUS4	0.000824	0
ENSP00000339521	RSU1	0.000824	0
ENSP00000340237	SH3BP4	0.000824	0
ENSP00000242839	ATP7B	0.000824	677
ENSP00000259938	CLPS	0.000824	518
ENSP00000329757	ATP6V0C	0.000824	0
ENSP00000348394	NCDN	0.000824	0
ENSP00000360371	SSBP3	0.000824	302
ENSP00000220876	STMN2	0.000824	405
ENSP00000300061	SCNN1G	0.000824	227
ENSP00000389792	DCDC1	0.000824	571
ENSP00000270223	DMWD	0.000824	0
ENSP00000273390	MAATS1	0.000824	0
ENSP00000304553	MPLKIP	0.000824	159
ENSP00000298472	SLC18A2	0.000824	609
ENSP00000353165	TPK1	0.000824	0
ENSP00000233809	IGFBP2	0.000824	262
ENSP00000240093	FZD3	0.000824	813
ENSP00000350447	SGOL2	0.000824	774

ENSP00000356737	GORAB	0.000824	204
ENSP00000366593	TMEM201	0.000824	0
ENSP00000378326	ZP3	0.000824	0
ENSP00000216338	GZMH	0.000824	224
ENSP00000260643	PREB	0.000824	796
ENSP00000273221	IQSEC1	0.000824	0
ENSP00000333496	KCND2	0.000824	913
ENSP00000357033	CD84	0.000824	0
ENSP00000223140	NOBOX	0.000824	157
ENSP00000369756	PTPRA	0.000824	0
ENSP00000375009	IGHV3-9	0.000824	183
ENSP00000269468	MBD1	0.000824	163
ENSP00000309968	ADAM17	0.000824	556
ENSP00000341815	SOX18	0.000824	228
ENSP00000391457	INO80C	0.000824	0
ENSP00000216277	PAPOLA	0.000824	0
ENSP00000229201	TIMELESS	0.000824	537
ENSP00000258682	CAMK2B	0.000824	808
ENSP00000266544	NDUFA9	0.000824	0
ENSP00000289429	CD1A	0.000824	360
ENSP00000305442	COG7	0.000824	0
ENSP00000417164	ROBO2	0.000824	290
ENSP00000290551	BTG2	0.000824	185
ENSP00000357311	CENPW	0.000824	458
ENSP00000361554	TIE1	0.000824	430
ENSP00000281938	HSPB8	0.000824	200
ENSP00000356056	DYNLT1	0.000824	340
ENSP00000374409	PKP4	0.000824	0
ENSP00000278198	LRRC4C	0.000824	252
ENSP00000315167	ALOX12B	0.000824	0
ENSP00000353344	ETS2	0.000824	713
ENSP00000362071	JPH2	0.000824	162
ENSP00000388340	CLINT1	0.000824	297

ENSP00000388724	HLA-A	0.000824	193
ENSP00000257879	ITGA7	0.000824	372
ENSP00000370962	GGT6	0.000824	207
ENSP00000289004	HPD	0.000824	0
ENSP00000321239	RCHY1	0.000824	203
ENSP00000347409	KEL	0.000824	240
ENSP00000365048	TNFSF13B	0.000824	308
ENSP00000295006	CAPN2	0.000824	177
ENSP00000344353	LPAR6	0.000824	899
ENSP00000260283	ARHGAP20	0.000824	162
ENSP00000365569	FLOT1	0.000824	0
ENSP00000369325	CDKL5	0.000824	198
ENSP00000260950	MSTN	0.000824	946
ENSP00000314080	HIC1	0.000824	305
ENSP00000274311	PELO	0.000824	750
ENSP00000329384	IL22	0.000824	243
ENSP00000350937	TES	0.000824	371
ENSP00000219172	CENPT	0.000824	0
ENSP00000305913	COL8A2	0.000824	905
ENSP00000357244	CCT3	0.000824	317
ENSP00000357348	HEY2	0.000824	748
ENSP00000378090	RAD51D	0.000824	0
ENSP00000259206	IL1RN	0.000824	319
ENSP00000231751	LTF	0.000824	240
ENSP00000321674	4-Sep	0.000824	245
ENSP00000268864	RASL10B	0.000824	374
ENSP00000324834	MUC3A	0.000824	0
ENSP00000351284	RAD52	0.000824	0
ENSP00000391490	AGR2	0.000824	180
ENSP00000227135	SPA17	0.000824	422
ENSP00000309052	CATSPER1	0.000824	462
ENSP00000311827	MSL2	0.000824	160
ENSP00000409231	TRAPPC13	0.000824	0

ENSP00000248594	PTPN12	0.000824	0
ENSP00000334145	F3	0.000824	219
ENSP00000360968	CYP4X1	0.000824	800
ENSP00000232458	ECT2	0.000824	852
ENSP00000351155	ATL1	0.000824	0
ENSP00000320340	DGKZ	0.000818	0
ENSP00000265310	TRPV5	0.000818	0
ENSP00000401632	GSTT1	0.000818	326
ENSP00000345096	IMPDH1	0.000818	508
ENSP00000369962	IGSF5	0.000818	0
ENSP00000263640	ACVR1	0.000818	273
ENSP00000301258	PSCA	0.000818	317
ENSP00000255608	BTBD2	0.000818	0
ENSP00000303077	GOT1L1	0.000818	0
ENSP00000345793	ZC3H7B	0.000818	0
ENSP00000264001	CKLF	0.000818	265
ENSP00000327417	GPR39	0.000818	730
ENSP00000238256	FKBP15	0.000812	258
ENSP00000267842	SLC27A2	0.000812	0
ENSP00000219409	ARHGDIG	0.000812	0
ENSP00000304604	MAGI3	0.000812	0
ENSP00000363431	NPY4R	0.000812	907
ENSP00000398852	SLC44A4	0.000812	0
ENSP00000362768	RBL1	0.000812	314
ENSP00000420418	ZNF398	0.000812	229
ENSP00000375859	TMEM91	0.0008	0
ENSP00000310557	KCNE3	0.000757	424
ENSP00000325136	HADHB	0.000714	0
ENSP00000331172	CD8B	0.000621	578
ENSP00000292301	CCR2	0.000221	886
ENSP00000358421	HSD3B1	0.000209	227
ENSP00000222212	CACNG7	0.000166	241
ENSP00000373091	HLA-E	0.000142	556

ENSP00000344822	S100A13	0.000135	0
ENSP00000348573	AKAP9	9.23E-05	0
ENSP00000276533	GINS4	6.15E-05	337
ENSP00000222982	CYP3A5	5.54E-05	932
ENSP00000257068	MTNR1B	5.54E-05	900
ENSP00000273158	SLC25A38	4.92E-05	565
ENSP00000303325	TACR3	4.92E-05	915
ENSP00000248272	GAN	3.69E-05	611
ENSP00000245457	PTGER2	3.69E-05	899
ENSP00000267085	CSAD	3.69E-05	374
ENSP00000260433	CYP19A1	3.08E-05	227
ENSP00000360502	PDE6C	2.46E-05	911
ENSP00000319197	OR4K17	2.46E-05	0
ENSP00000351790	MYPN	2.46E-05	196
ENSP00000261532	ESRRB	1.85E-05	900
ENSP00000384169	FBLN2	1.85E-05	529
ENSP00000354607	FZD5	1.23E-05	943
ENSP00000375557	POU5F1B	1.23E-05	675
ENSP00000259216	CFC1	1.23E-05	750
ENSP00000299550	TRIM66	1.23E-05	374
ENSP00000384179	ZFPM2	1.23E-05	968
ENSP00000294309	TPCN2	1.23E-05	466
ENSP00000340328	NYX	1.23E-05	445
ENSP00000355316	GRM3	1.23E-05	794
ENSP00000221855	TBCB	1.23E-05	0
ENSP00000345752	MTMR2	1.23E-05	195
ENSP00000395505	PATE3	1.23E-05	563
ENSP00000339801	IDS	1.23E-05	893
ENSP00000263269	GRIN2D	1.23E-05	428
ENSP00000236137	SLC19A2	1.23E-05	0
ENSP00000255262	NMUR2	6.15E-06	904
ENSP00000261454	PROX1	6.15E-06	659
ENSP00000277225	ZNF462	6.15E-06	340

ENSP00000309509	PLAC8	6.15E-06	0
ENSP00000309818	TRHR	6.15E-06	919
ENSP00000321821	CYP4F12	6.15E-06	290
ENSP00000325123	ZSCAN2	6.15E-06	330
ENSP00000326371	FOXC2	6.15E-06	675
ENSP00000352162	ELAVL3	6.15E-06	535
ENSP00000365469	KLHL35	6.15E-06	227
ENSP00000227451	DTX4	6.15E-06	305
ENSP00000296589	SLC45A2	6.15E-06	559
ENSP00000296839	FOXQ1	6.15E-06	621
ENSP00000309741	SLCO4C1	6.15E-06	340
ENSP00000320965	B4GALT3	6.15E-06	184
ENSP00000346689	LMO3	6.15E-06	550
ENSP00000351767	COL20A1	6.15E-06	911
ENSP00000315757	LCP1	6.15E-06	445
ENSP00000350833	MPPED2	6.15E-06	408
ENSP00000390784	B3GALT4	6.15E-06	318
ENSP00000406988	VPS52	6.15E-06	227
ENSP00000408405	KCTD1	6.15E-06	653
ENSP00000361668	EDN2	6.15E-06	918
ENSP00000278175	ADM	6.15E-06	919
ENSP00000341550	SLC24A5	6.15E-06	379
ENSP00000348897	GABRA2	6.15E-06	420
ENSP00000343924	PRELP	6.15E-06	676
ENSP00000364946	MKX	6.15E-06	306
ENSP00000392349	RPS18	6.15E-06	260
ENSP00000274938	SCUBE3	6.15E-06	305
ENSP00000319591	KCND3	6.15E-06	904
ENSP00000362217	DACH2	6.15E-06	715
ENSP00000389244	SLC44A4	6.15E-06	0
ENSP00000393275	NANOS1	6.15E-06	238
ENSP00000286604	UGT2A1	6.15E-06	899
ENSP00000308496	ARL10	6.15E-06	0

ENSP00000317721	PIPOX	6.15E-06	157
ENSP00000292174	CXCR5	6.15E-06	937
ENSP00000363163	NLGN3	6.15E-06	716
ENSP00000331933	WSCD2	6.15E-06	466
ENSP00000334424	AMACR	6.15E-06	0
ENSP00000241463	RASL11A	6.15E-06	162
ENSP00000362353	GLP1R	6.15E-06	925
ENSP00000381340	GGT5	6.15E-06	196

4. Candidate genes for microRNA target genes and somatic mutation genes

Ensembl ID	Gene symbol	Betweenness ratio	Min-Max interaction score
ENSP00000206249	ESR1	0.120766	997
ENSP00000350941	SRC	0.090798	999
ENSP00000344456	CTNNB1	0.090399	996
ENSP00000335153	HSP90AA1	0.056512	975
ENSP00000357656	FYN	0.053285	994
ENSP00000339007	GRB2	0.050661	999
ENSP00000297494	NOS3	0.048115	563
ENSP00000349467	CALM1	0.045052	969
ENSP00000401303	SHC1	0.041171	999
ENSP00000351486	NTRK1	0.039895	302
ENSP00000277541	NOTCH1	0.038253	948
ENSP00000293379	ITGA5	0.03786	835
ENSP00000262613	SLC9A3R1	0.036407	895
ENSP00000338934	EZR	0.035726	950
ENSP00000296585	ITGA2	0.034562	959
ENSP00000358525	NGF	0.032219	943
ENSP00000251849	RAF1	0.030759	999
ENSP00000364133	TGFBR1	0.027958	310
ENSP00000312435	DAG1	0.027238	994
ENSP00000314458	CDC42	0.027232	979
ENSP00000223023	WASL	0.024103	914
ENSP00000265335	RAD50	0.023351	899

ENSP00000242577	DYNLL1	0.023213	787
ENSP00000350311	-	0.022703	886
ENSP00000332353	PTCH1	0.022245	430
ENSP00000304669	CTNNA1	0.022147	944
ENSP00000348444	TTN(titin)	0.021257	504
ENSP00000359074	L1CAM	0.020825	960
ENSP00000371138	FKBP1A	0.02055	998
ENSP00000361125	VEGFA	0.020373	984
ENSP00000358022	MCL1	0.019156	835
ENSP00000254942	TERF2	0.019071	899
ENSP00000297268	COL1A2	0.016865	985
ENSP00000265709	ANK1	0.015726	560
ENSP00000300134	STAT6	0.015072	878
ENSP00000162330	BCAR1	0.01481	593
ENSP00000351209	EPHA2	0.014084	953
ENSP00000261769	CDH1	0.013979	955
ENSP00000256443	CDK7	0.013796	981
ENSP00000228307	PXN	0.013547	702
ENSP00000345206	RBPJ	0.012343	925
ENSP00000276603	TERF1	0.011715	956
ENSP00000273481	-	0.011708	965
ENSP00000268171	FURIN	0.011695	723
ENSP00000361850	PLAU	0.011682	965
ENSP00000352514	RUNX2	0.011656	872
ENSP00000340698	GIPC1	0.011381	805
ENSP00000322898	EBF1	0.010465	914
ENSP00000330633	CNTN2	0.010183	865
ENSP00000381227	-	0.009987	374
ENSP00000288986	NCK1	0.009843	994
ENSP00000267415	TINF2	0.009463	899
ENSP00000349960	ACTB	0.009116	877
ENSP00000281708	FBXW7	0.008639	945
ENSP00000360916	VAV2	0.00856	964

ENSP00000361405	MMP9	0.007768	924
ENSP00000357392	EFNA1	0.007703	991
ENSP00000220584	FDFT1	0.007624	340
ENSP00000348786	RAP1A	0.007585	366
ENSP00000354251	NCKAP1	0.007546	354
ENSP00000261783	ARG2	0.007493	280
ENSP00000359506	FMR1	0.007232	826
ENSP00000324549	CYFIP1	0.007219	213
ENSP00000381107	-	0.006937	204
ENSP00000386896	ITGA6	0.006616	922
ENSP00000308176	BTK	0.006597	942
ENSP00000354541	NLGN1	0.006531	629
ENSP00000245323	EFNB2	0.006479	993
ENSP00000343313	ATG5	0.006479	768
ENSP00000316854	ATOX1	0.006479	340
ENSP00000377941	ACTN1	0.006479	899
ENSP00000370408	CDX2	0.006473	802
ENSP00000328777	EFNA5	0.006473	994
ENSP00000331327	WT1	0.006473	962
ENSP00000252034	ELN	0.006473	542
ENSP00000200181	ITGB4	0.006473	925
ENSP00000358994	MYO6	0.006466	899
ENSP00000358323	TXNIP	0.006466	653
ENSP00000001008	FKBP4	0.006466	371
ENSP00000225893	HNF1B	0.006459	243
ENSP00000332592	SPAG16	0.006453	371
ENSP00000381450	-	0.006453	457
ENSP00000228841	MYL2	0.00644	674
ENSP00000267845	HDC	0.006433	625
ENSP00000257430	APC	0.006433	976
ENSP00000345656	VAPA	0.006427	350
ENSP00000380148	-	0.006427	297
ENSP00000359297	NSDHL	0.006414	166

ENSP00000363021	RPA2	0.006407	306
ENSP00000347198	SRGAP1	0.006407	246
ENSP00000261507	MSMO1	0.006401	0
ENSP00000353731	DPP4	0.006394	345
ENSP00000375809	ERCC2	0.006394	973
ENSP00000366563	PIK3CD	0.006368	946
ENSP00000302234	CCL11	0.006361	330
ENSP00000262053	ATF1	0.006342	180
ENSP00000319060	CAMK2G	0.006342	946
ENSP00000316460	FYB	0.006263	754
ENSP00000338785	STARD13	0.00623	195
ENSP00000262188	SMARCD3	0.00623	899
ENSP00000313391	DAB2	0.006198	508
ENSP00000296325	-	0.00606	899
ENSP00000261531	SNW1	0.00591	389
ENSP00000266376	CACNA1C	0.005654	317
ENSP00000384675	SOS1	0.005471	998
ENSP00000268134	-	0.005281	969
ENSP00000374372	SPTB	0.005262	899
ENSP00000382193	MYBPC3	0.005255	739
ENSP00000322542	GTF2I	0.005242	778
ENSP00000265071	CDH6	0.005236	906
ENSP00000341170	PTN	0.005236	824
ENSP00000372313	MSLN	0.005236	240
ENSP00000242839	ATP7B	0.005236	677
ENSP00000344468	SDC3	0.005236	929
ENSP00000372160	DOK6	0.005236	346
ENSP00000286621	ADK	0.005236	893
ENSP00000367714	HES5	0.005236	929
ENSP00000233813	IGFBP5	0.005236	811
ENSP00000360310	SPO11	0.005236	846
ENSP00000312185	ELMO1	0.005236	269
ENSP00000372547	SRY	0.005236	678

ENSP00000225698	C1QBP	0.005236	715
ENSP00000310448	SART1	0.005236	752
ENSP00000363360	INIP	0.005229	424
ENSP00000379457	FAF1	0.005229	808
ENSP00000360200	INADL	0.005229	900
ENSP00000265362	SEMA3A	0.005229	948
ENSP00000303522	TACR1	0.005229	899
ENSP00000360689	TNKS2	0.005229	619
ENSP00000337397	DKFZP686J19100	0.005229	411
ENSP00000361777	SET	0.005229	705
ENSP00000377958	CCT4	0.005229	265
ENSP00000417864	ANP32A	0.005229	228
ENSP00000348634	MYH6	0.005223	908
ENSP00000383210	NEK3	0.005223	703
ENSP00000295709	STK36	0.005223	213
ENSP00000367486	MEIG1	0.005223	154
ENSP00000254351	SDC1	0.005223	942
ENSP00000380349	CAPN3	0.005223	486
ENSP00000352657	ME3	0.005216	425
ENSP00000348664	-	0.005216	462
ENSP00000362058	NDUFS5	0.005216	899
ENSP00000309595	C10orf2	0.005209	839
ENSP00000385450	MAGI1	0.005209	462
ENSP00000258341	LAMC1	0.005209	909
ENSP00000261681	MPP5	0.005203	679
ENSP00000349204	CRB3	0.005203	477
ENSP00000344742	STAMBP	0.005203	845
ENSP00000356641	RFWD2	0.005196	948
ENSP00000366746	STAM	0.005196	927
ENSP00000252456	CNN1	0.00519	468
ENSP00000291525	TFF3	0.00519	372
ENSP00000339861	ENY2	0.00519	340
ENSP00000301200	CDC42EP5	0.00519	229

ENSP00000222399	LAMB1	0.005183	906
ENSP00000225655	PFN1	0.005183	581
ENSP00000390849	ABHD5	0.005183	228
ENSP00000348168	GTF2E2	0.005164	444
ENSP00000361965	ADA	0.005157	399
ENSP00000348089	ERCC6	0.005052	927
ENSP00000286548	GNAQ	0.005033	919
ENSP00000268182	IQGAP1	0.004025	917
ENSP00000360678	-	0.00375	0
ENSP00000349275	NRG1	0.002644	979
ENSP00000355566	TOMM20	0.0025	238
ENSP00000264499	BBS7	0.0025	424
ENSP00000293831	EIF4A1	0.0025	213
ENSP00000269485	TNFRSF11A	0.0025	340
ENSP00000347839	RAB11FIP2	0.0025	296
ENSP00000354003	GYPA	0.002225	514
ENSP00000297338	RAD21	0.00163	899
ENSP00000400591	SNRPE	0.001433	254
ENSP00000380378	PAFAH1B1	0.001302	980
ENSP00000357674	SNAPIN	0.001289	235
ENSP00000305913	COL8A2	0.001289	906
ENSP00000261037	COL8A1	0.001289	905
ENSP00000371682	DCAF16	0.001283	0
ENSP00000345728	ATP7A	0.001257	669
ENSP00000351141	WTAP	0.00125	254
ENSP00000204615	THPO	0.00125	368
ENSP00000222792	CHN2	0.00125	170
ENSP00000320838	GSG1	0.00125	154
ENSP00000254661	RAMP1	0.00125	0
ENSP00000326432	CCR8	0.00125	899
ENSP00000350990	TNKS1BP1	0.00125	247
ENSP00000228641	MYF6	0.00125	598
ENSP00000254691	CARD6	0.00125	229

ENSP00000366603	TGOLN2	0.00125	330
ENSP00000406359	HSPA1A	0.00125	911
ENSP00000307549	NPTX1	0.00125	625
ENSP00000309622	TFDP2	0.00125	260
ENSP00000250111	ATP1B2	0.00125	406
ENSP00000349955	RPRD1A	0.00125	0
ENSP00000247026	NSRP1	0.00125	249
ENSP00000275603	CCT6A	0.00125	226
ENSP00000231228	IL12B	0.00125	462
ENSP00000317872	RBBP6	0.00125	513
ENSP00000329715	DRG1	0.00125	0
ENSP00000334940	GGN	0.00125	0
ENSP00000354777	TBKBP1	0.00125	0
ENSP00000265164	CASP6	0.00125	939
ENSP00000337194	PRPF4B	0.00125	483
ENSP00000346886	GABPA	0.00125	846
ENSP00000341848	GOLGB1	0.00125	248
ENSP00000240652	IAPP	0.00125	899
ENSP00000313581	KLK2	0.00125	168
ENSP00000365766	TIMM17B	0.00125	0
ENSP00000360891	IFIT2	0.00125	361
ENSP00000305595	B3GNT2	0.00125	899
ENSP00000340507	TRIM24	0.00125	878
ENSP00000351363	MSMB	0.00125	329
ENSP00000226091	EFNB3	0.00125	971
ENSP00000364398	HABP4	0.00125	621
ENSP00000394033	KCNK2	0.00125	200
ENSP00000266085	TIMP3	0.00125	866
ENSP00000354033	PCGF2	0.00125	199
ENSP00000357692	S100A16	0.00125	0
ENSP00000227618	ANAPC15	0.00125	0
ENSP00000385021	FANCL	0.00125	273
ENSP00000386171	ESRRG	0.00125	463

ENSP00000240874	KALRN	0.00125	201
ENSP00000250894	MAPK8IP3	0.00125	742
ENSP00000365811	SPAG6	0.00125	540
ENSP00000266427	ETV6	0.00125	507
ENSP00000297562	AP5Z1	0.00125	263
ENSP00000403557	PPP1R11	0.00125	0
ENSP00000404232	EFHC2	0.00125	299
ENSP00000311579	TNKS	0.00125	386
ENSP00000356529	RGS16	0.00125	734
ENSP00000299163	HIF1AN	0.00125	200
ENSP00000309968	ADAM17	0.00125	984
ENSP00000334594	SLC10A7	0.00125	430
ENSP00000346440	TCF4	0.00125	462
ENSP00000272233	RHOB	0.00125	523
ENSP00000303423	FNTA	0.00125	638
ENSP00000354376	RAB25	0.00125	307
ENSP00000168216	HSD17B10	0.00125	244
ENSP00000260950	MSTN	0.00125	559
ENSP00000318775	ANAPC4	0.00125	0
ENSP00000360645	MAN1B1	0.00125	0
ENSP00000385057	APOBEC3G	0.00125	563
ENSP00000414237	INTS2	0.00125	243
ENSP00000381100	-	0.00125	247
ENSP00000291582	AIRE	0.00125	187
ENSP00000322229	FADS1	0.00125	304
ENSP00000281928	MED13L	0.00125	285
ENSP00000326830	CLK1	0.00125	693
ENSP00000361548	MPL	0.00125	305
ENSP00000371475	TP53BP1	0.00125	938
ENSP00000307939	GCC2	0.00125	0
ENSP00000346148	PRKAA1	0.00125	972
ENSP00000378288	MYLK3	0.00125	427
ENSP00000278198	LRRC4C	0.00125	569

ENSP00000357244	CCT3	0.00125	279
ENSP00000357311	CENPW	0.00125	552
ENSP00000361473	KDM4A	0.00125	0
ENSP00000362578	RNF8	0.00125	260
ENSP00000256497	EDEM1	0.00125	228
ENSP00000328216	ORAI1	0.00125	348
ENSP00000338207	LMO1	0.00125	189
ENSP00000320567	MRPS33	0.00125	0
ENSP00000419923	KLF6	0.00125	718
ENSP00000246041	AP5S1	0.00125	0
ENSP00000382791	GRIK1	0.00125	753
ENSP00000258774	HUS1	0.00125	317
ENSP00000326261	SRRM1	0.00125	528
ENSP00000340688	LPHN1	0.001243	335
ENSP00000347710	OPHN1	0.001243	581
ENSP00000216410	GNPNAT1	0.001243	180
ENSP00000218652	NDFIP2	0.001243	364
ENSP00000367747	PLCH2	0.001243	251
ENSP00000308549	ADORA1	0.001243	901
ENSP00000362744	RPS4X	0.001243	0
ENSP00000274711	LRRTM2	0.001243	893
ENSP00000315173	ZNF41	0.001243	621
ENSP00000299339	CLDN10	0.001243	235
ENSP00000283977	PGM3	0.001243	165
ENSP00000321826	STXBP5	0.001243	237
ENSP00000339845	DROSHA	0.001243	921
ENSP00000374323	EPHA6	0.001243	903
ENSP00000359910	PSMA7	0.001243	298
ENSP00000304102	COPS6	0.001243	583
ENSP00000297373	PHKG1	0.001243	0
ENSP00000256689	SLC38A2	0.001237	165
ENSP00000352673	ELF3	0.001237	633
ENSP00000293441	SHANK1	0.001237	632

ENSP00000265529	KIF9	0.001237	0
ENSP00000230895	DAP	0.001237	778
ENSP00000285398	ERCC3	0.001224	901
ENSP00000246194	RALY	0.001204	200
ENSP00000287322	BAG4	0.001152	302
ENSP00000284154	GRAP	0.001152	390
ENSP00000273398	ATP6V1A	0.001152	240
ENSP00000255688	RARRES3	0.001126	236
ENSP00000310723	DDX23	0.001113	187
ENSP00000303634	LRP8	0.000399	917
ENSP00000296785	ANKRA2	0.000321	305
ENSP00000257068	MTNR1B	0.000105	899
ENSP00000419361	ADCY5	7.85E-05	904
ENSP00000304188	OR8U1	5.89E-05	372
ENSP00000302397	ATP1A3	3.27E-05	609
ENSP00000312235	MUC13	2.62E-05	907
ENSP00000252575	NCAN	2.62E-05	918
ENSP00000372853	C2	2.62E-05	502
ENSP00000356115	MFSD4	1.96E-05	435
ENSP00000400223	FJX1	1.96E-05	420
ENSP00000020926	SYT13	1.31E-05	272
ENSP00000230538	LAMA4	1.31E-05	511
ENSP00000270233	BCAM	1.31E-05	296
ENSP00000325123	ZSCAN2	1.31E-05	380
ENSP00000332151	DSE	1.31E-05	911
ENSP00000353557	SLC35F1	1.31E-05	380
ENSP00000363642	BMS1	1.31E-05	202
ENSP00000352834	MYO1C	1.31E-05	899
ENSP00000352336	PLCG2	1.31E-05	910
ENSP00000356652	CACYBP	1.31E-05	899
ENSP00000225298	UTP18	1.31E-05	388
ENSP00000397181	RGS4	1.31E-05	746
ENSP00000184266	NDUFB4	1.31E-05	0

ENSP00000255380	CHRM3	1.31E-05	906
ENSP00000261722	AP3B2	6.54E-06	253
ENSP00000266579	SLC38A4	6.54E-06	340
ENSP00000329748	CPNE8	6.54E-06	243
ENSP00000338217	ZNF532	6.54E-06	243
ENSP00000348816	-	6.54E-06	461
ENSP00000406027	EPM2AIP1	6.54E-06	412
ENSP00000412283	IER3	6.54E-06	386
ENSP00000023939	RTFDC1	6.54E-06	350
ENSP00000282041	EPG5	6.54E-06	290
ENSP00000320324	NPEPPS	6.54E-06	641
ENSP00000337839	ZCCHC7	6.54E-06	379
ENSP00000367651	PRDM16	6.54E-06	621
ENSP00000215862	MORC2	6.54E-06	274
ENSP00000255448	DCLK1	6.54E-06	402
ENSP00000313084	EGFLAM	6.54E-06	307
ENSP00000322020	SLC25A22	6.54E-06	517
ENSP00000375238	KRTAP2-1	6.54E-06	268
ENSP00000298310	NEMF	6.54E-06	294
ENSP00000339390	CDH26	6.54E-06	266
ENSP00000363727	STARD8	6.54E-06	230
ENSP00000262219	ANXA13	6.54E-06	229
ENSP00000255882	PI4KA	6.54E-06	906
ENSP00000363799	ACTL7B	6.54E-06	412
ENSP00000332504	CCR10	6.54E-06	227
ENSP00000358099	RGS10	6.54E-06	241
ENSP00000203556	GMIP	6.54E-06	170
ENSP00000406674	MOGAT1	6.54E-06	253
ENSP00000263856	CHMP3	6.54E-06	919
ENSP00000363533	MDH1B	6.54E-06	157
ENSP00000220478	SCG3	6.54E-06	160
ENSP00000296802	NSA2	6.54E-06	235
ENSP00000381634	SLC38A1	6.54E-06	0

ENSP00000303147	MAT2A	6.54E-06	400
ENSP00000265080	RASGRF2	6.54E-06	902
ENSP00000359245	ABCA4	6.54E-06	516
ENSP00000252699	ACTN4	6.54E-06	899
ENSP00000417052	EBP	6.54E-06	281

5. Candidate genes for microRNA target genes and mRNA genes

Ensembl ID	Gene symbol	Betweenness ratio	Min-Max interaction score
ENSP00000264033	CBL	0.169869	990
ENSP00000206249	ESR1	0.105013	998
ENSP00000344456	CTNNB1	0.062609	999
ENSP00000263253	EP300	0.062112	995
ENSP00000350941	SRC	0.06149	999
ENSP00000264657	STAT3	0.061359	997
ENSP00000339007	GRB2	0.049755	939
ENSP00000357656	FYN	0.040555	981
ENSP00000266970	CDK2	0.037183	999
ENSP00000297494	NOS3	0.030974	945
ENSP00000277541	NOTCH1	0.02811	982
ENSP00000293379	ITGA5	0.026291	964
ENSP00000312652	LEP	0.023424	971
ENSP00000244007	PLCG1	0.023392	969
ENSP00000401303	SHC1	0.022899	967
ENSP00000351486	NTRK1	0.021566	800
ENSP00000332353	PTCH1	0.017828	939
ENSP00000387662	GCG	0.015605	939
ENSP00000223023	WASL	0.015471	901
ENSP00000361125	VEGFA	0.01523	984
ENSP00000278616	ATM	0.015223	972
ENSP00000269571	ERBB2	0.014603	916
ENSP00000306512	IL8	0.014293	992
ENSP00000302269	VAV1	0.013074	983
ENSP00000358525	NGF	0.013015	943

ENSP00000296585	ITGA2	0.012625	987
ENSP00000340944	PTPN11	0.011916	988
ENSP00000361423	ABL1	0.011859	989
ENSP00000344352	ATF3	0.011195	889
ENSP00000297261	SHH	0.011107	986
ENSP00000162330	BCAR1	0.010931	986
ENSP00000354720	SMC3	0.010731	986
ENSP00000302150	PRL	0.010075	931
ENSP00000367408	CASK	0.009421	982
ENSP00000300134	STAT6	0.008382	945
ENSP00000228307	PXN	0.008275	878
ENSP00000245451	BMP4	0.007896	971
ENSP00000351209	EPHA2	0.007793	932
ENSP00000318472	NCAM1	0.007432	918
ENSP00000299421	ILK	0.007316	989
ENSP00000368350	TPT1	0.006952	863
ENSP00000312999	GNAI2	0.006407	965
ENSP00000264554	SHC2	0.006184	995
ENSP00000360916	VAV2	0.006182	964
ENSP00000302961	HSPA4	0.006066	958
ENSP00000258301	STX6	0.005805	884
ENSP00000290541	PSMB4	0.005756	906
ENSP00000295598	ATP1A1	0.005755	904
ENSP00000293272	CCL5	0.005471	958
ENSP00000331746	CALCA	0.005218	992
ENSP00000363092	PRKG1	0.005009	946
ENSP00000315955	FOXA2	0.005003	939
ENSP00000354586	GLI2	0.004952	900
ENSP00000357392	EFNA1	0.004921	947
ENSP00000354541	NLGN1	0.004573	629
ENSP00000268171	FURIN	0.004541	911
ENSP00000170630	IL4R	0.004533	906
ENSP00000334122	FGF3	0.004531	904

ENSP00000314813	OAZ1	0.004528	905
ENSP00000363079	MBL2	0.004509	824
ENSP00000384675	SOS1	0.004437	963
ENSP00000322570	POLE	0.004358	904
ENSP00000356623	CITED2	0.004142	927
ENSP00000400806	APTX	0.004131	506
ENSP00000175506	ASNS	0.004075	899
ENSP00000261783	ARG2	0.004042	964
ENSP00000263923	KDR	0.003897	935
ENSP00000349960	ACTB	0.003779	974
ENSP00000242067	BBS9	0.003734	385
ENSP00000257899	BLOC1S1	0.00373	319
ENSP00000342011	XRCC4	0.003722	671
ENSP00000296140	CCR1	0.003717	965
ENSP00000204604	CHRD	0.003713	825
ENSP00000333633	MTA1	0.003692	778
ENSP00000222725	LFNG	0.003682	504
ENSP00000394624	OPRM1	0.003634	916
ENSP00000276420	DOK2	0.003517	906
ENSP00000413234	AP2A2	0.00347	906
ENSP00000269485	TNFRSF11A	0.00332	800
ENSP00000306881	SEC23A	0.00332	506
ENSP00000254227	NR0B2	0.003319	960
ENSP00000337103	CHAT	0.00331	788
ENSP00000225655	PFN1	0.003106	621
ENSP00000316460	FYB	0.002951	562
ENSP00000261037	COL8A1	0.002902	905
ENSP00000280193	VEGFC	0.002893	919
ENSP00000043402	RTN4R	0.002892	517
ENSP00000405890	PBX1	0.002891	859
ENSP00000357753	IVL	0.00289	821
ENSP00000347198	SRGAP1	0.002872	245
ENSP00000248996	GNAZ	0.002806	910

ENSP00000339151	IKBKB	0.002538	641
ENSP00000233057	EIF2AK2	0.002531	569
ENSP00000359206	BTRC	0.002513	999
ENSP00000264499	BBS7	0.002506	675
ENSP00000293831	EIF4A1	0.0025	569
ENSP00000322142	ING5	0.0025	271
ENSP00000362166	MEAF6	0.0025	306
ENSP00000347839	RAB11FIP2	0.002494	691
ENSP00000360183	STX16	0.002493	614
ENSP00000355566	TOMM20	0.002492	733
ENSP00000377446	SUCLG1	0.002489	461
ENSP00000281821	EPHA4	0.002486	985
ENSP00000161559	CEACAM1	0.002473	303
ENSP00000201586	SULT2B1	0.002471	607
ENSP00000356789	ATP1B1	0.002468	907
ENSP00000359285	CHRNA4	0.002463	852
ENSP00000291386	SSU72	0.002459	899
ENSP00000372815	C4A	0.002455	579
ENSP00000383690	MASP2	0.002455	648
ENSP00000386165	CEBPD	0.002391	900
ENSP00000354003	GYPA	0.002351	514
ENSP00000360217	RHAG	0.002345	492
ENSP00000355361	CD47	0.002341	688
ENSP00000261267	LYZ	0.002274	928
ENSP00000260867	TIMM23	0.002113	274
ENSP00000305913	COL8A2	0.002084	906
ENSP00000357311	CENPW	0.002073	595
ENSP00000260950	MSTN	0.002073	847
ENSP00000257963	ACVR1B	0.002073	677
ENSP00000226091	EFNB3	0.002072	379
ENSP00000278198	LRRC4C	0.002072	271
ENSP00000326261	SRRM1	0.002072	629
ENSP00000219172	CENPT	0.002072	914

ENSP00000370408	CDX2	0.002072	911
ENSP00000358497	RNGTT	0.002072	891
ENSP00000354033	PCGF2	0.002071	958
ENSP00000370503	CCM2	0.002071	469
ENSP00000255465	CCNA1	0.002071	931
ENSP00000222792	CHN2	0.00207	329
ENSP00000266085	TIMP3	0.00207	926
ENSP00000266427	ETV6	0.00207	677
ENSP00000355599	TSNAX	0.00207	609
ENSP00000304915	IL13	0.00207	854
ENSP00000358857	EMD	0.00207	619
ENSP00000357244	CCT3	0.002068	999
ENSP00000315173	ZNF41	0.002067	479
ENSP00000246032	STK35	0.002067	528
ENSP00000396439	RING1	0.002067	963
ENSP00000259089	BLK	0.002066	901
ENSP00000315167	ALOX12B	0.002064	899
ENSP00000309968	ADAM17	0.002063	943
ENSP00000257497	ANXA1	0.002063	946
ENSP00000267845	HDC	0.002059	679
ENSP00000324740	YES1	0.002058	925
ENSP00000382791	GRIK1	0.002058	675
ENSP00000304283	RAC3	0.002058	911
ENSP00000344460	CBS	0.002042	429
ENSP00000366563	PIK3CD	0.002042	920
ENSP00000362795	CXCR3	0.002031	986
ENSP00000302707	FPR1	0.002017	907
ENSP00000262958	GNA15	0.001986	963
ENSP00000338785	STARD13	0.001974	573
ENSP00000262188	SMARCD3	0.001974	899
ENSP00000297338	RAD21	0.001957	993
ENSP00000304767	P2RY1	0.001653	904
ENSP00000359151	DBT	0.001652	379

ENSP00000199764	CEACAM6	0.001649	283
ENSP00000393887	AHSG	0.001649	914
ENSP00000247170	DAAM1	0.001649	893
ENSP00000262776	LGALS3BP	0.001649	719
ENSP00000264234	UPK1B	0.001649	229
ENSP00000264126	GPSM2	0.001649	524
ENSP00000328968	SCN5A	0.001648	898
ENSP00000231524	TRIM23	0.001646	657
ENSP00000295709	STK36	0.001644	543
ENSP00000331902	COL4A5	0.001644	907
ENSP00000283147	BMP6	0.001642	854
ENSP00000413720	CDKN1C	0.001637	940
ENSP00000019103	SCTR	0.001635	932
ENSP00000260630	CYP1B1	0.001633	569
ENSP00000357721	S100A8	0.00163	666
ENSP00000350256	CCR9	0.001619	866
ENSP00000225844	CCL13	0.001619	750
ENSP00000323280	CD6	0.001616	385
ENSP00000305988	ALCAM	0.001616	347
ENSP00000263025	MAPK3	0.001429	935
ENSP00000241256	GHSR	0.001401	902
ENSP00000400591	SNRPE	0.001354	616
ENSP00000275603	CCT6A	0.00132	999
ENSP00000357674	SNAPIN	0.001284	440
ENSP00000371682	DCAF16	0.001252	241
ENSP00000317872	RBBP6	0.00125	496
ENSP00000231228	IL12B	0.00125	998
ENSP00000240652	IAPP	0.00125	922
ENSP00000254691	CARD6	0.00125	378
ENSP00000307549	NPTX1	0.00125	516
ENSP00000329715	DRG1	0.00125	619
ENSP00000337194	PRPF4B	0.00125	396
ENSP00000341848	GOLGB1	0.00125	847

ENSP00000204615	THPO	0.00125	878
ENSP00000228641	MYF6	0.00125	647
ENSP00000351141	WTAP	0.00125	604
ENSP00000250111	ATP1B2	0.00125	907
ENSP00000320838	GSG1	0.00125	154
ENSP00000326432	CCR8	0.00125	947
ENSP00000313581	KLK2	0.00125	692
ENSP00000340507	TRIM24	0.00125	401
ENSP00000265164	CASP6	0.00125	379
ENSP00000297562	AP5Z1	0.00125	360
ENSP00000346440	TCF4	0.00125	972
ENSP00000303423	FNTA	0.00125	644
ENSP00000403557	PPP1R11	0.00125	899
ENSP00000240874	KALRN	0.00125	790
ENSP00000361548	MPL	0.00125	875
ENSP00000386171	ESRRG	0.00125	902
ENSP00000356529	RGS16	0.00125	243
ENSP00000250894	MAPK8IP3	0.00125	0
ENSP00000346148	PRKAA1	0.00125	865
ENSP00000335632	CHP1	0.00125	800
ENSP00000284995	TSEN2	0.00125	175
ENSP00000247026	NSRP1	0.001249	159
ENSP00000351363	MSMB	0.001249	657
ENSP00000406359	HSPA1A	0.001249	918
ENSP00000366603	TGOLN2	0.001249	401
ENSP00000283977	PGM3	0.001249	425
ENSP00000359910	PSMA7	0.001249	912
ENSP00000346886	GABPA	0.001249	462
ENSP00000309622	TFDP2	0.001249	958
ENSP00000299163	HIF1AN	0.001249	638
ENSP00000281928	MED13L	0.001249	899
ENSP00000365811	SPAG6	0.001249	556
ENSP00000352673	ELF3	0.001248	722

ENSP00000334940	GGN	0.001248	306
ENSP00000362744	RPS4X	0.001248	286
ENSP00000293441	SHANK1	0.001248	460
ENSP00000230895	DAP	0.001248	778
ENSP00000339845	DROSHA	0.001248	477
ENSP00000350990	TNKS1BP1	0.001248	0
ENSP00000354777	TBKBP1	0.001248	335
ENSP00000364398	HABP4	0.001248	439
ENSP00000337722	ARL6	0.001248	258
ENSP00000360891	IFIT2	0.001247	267
ENSP00000264079	MCOLN1	0.001247	307
ENSP00000385021	FANCL	0.001247	993
ENSP00000334594	SLC10A7	0.001247	465
ENSP00000357692	S100A16	0.001246	792
ENSP00000320604	FAXDC2	0.001246	461
ENSP00000339916	LIMK2	0.001246	800
ENSP00000308549	ADORA1	0.001245	902
ENSP00000254661	RAMP1	0.001245	619
ENSP00000274711	LRRTM2	0.001245	893
ENSP00000321826	STXBP5	0.001245	798
ENSP00000227618	ANAPC15	0.001245	434
ENSP00000349955	RPRD1A	0.001244	623
ENSP00000340688	LPHN1	0.001243	335
ENSP00000365766	TIMM17B	0.001243	250
ENSP00000218652	NDFIP2	0.001243	348
ENSP00000305595	B3GNT2	0.001242	910
ENSP00000347710	OPHN1	0.001241	807
ENSP00000284690	DHX32	0.00124	229
ENSP00000367747	PLCH2	0.00124	508
ENSP00000216410	GNPNAT1	0.001239	902
ENSP00000265529	KIF9	0.001239	268
ENSP00000256689	SLC38A2	0.001235	463
ENSP00000285398	ERCC3	0.001202	899

ENSP00000273398	ATP6V1A	0.00119	350
ENSP00000246194	RALY	0.001188	429
ENSP00000284154	GRAP	0.001161	514
ENSP00000255688	RARRES3	0.001153	675
ENSP00000287322	BAG4	0.00114	294
ENSP00000310723	DDX23	0.001086	204
ENSP00000368686	E2F4	0.001047	907
ENSP00000340396	GBP5	0.000829	899
ENSP00000168712	FGF4	0.000827	923
ENSP00000377840	CACNB1	0.000825	621
ENSP00000300417	LRSAM1	0.000824	430
ENSP00000352442	HIST1H2BM	0.000824	322
ENSP00000221283	STXBP2	0.000824	601
ENSP00000357025	CD48	0.000824	511
ENSP00000264009	HSF4	0.000824	619
ENSP00000343819	OTX2	0.000824	534
ENSP00000350332	MYBPC2	0.000824	770
ENSP00000233202	SLC11A1	0.000824	524
ENSP00000264001	CKLF	0.000824	263
ENSP00000356906	SH2D1B	0.000824	506
ENSP00000369009	CXorf23	0.000824	518
ENSP00000268704	SPG7	0.000824	430
ENSP00000216373	SOS2	0.000824	899
ENSP00000301420	KLK1	0.000824	286
ENSP00000283871	HGD	0.000824	765
ENSP00000265294	GABRP	0.000824	219
ENSP00000245539	MRPS7	0.000823	563
ENSP00000252593	BST2	0.000823	326
ENSP00000310658	SCUBE2	0.000823	260
ENSP00000354900	GJB1	0.000823	745
ENSP00000369962	IGSF5	0.000823	906
ENSP00000338728	CCDC88A	0.000823	257
ENSP00000301258	PSCA	0.000823	254

ENSP00000248071	KLF2	0.000823	886
ENSP00000316990	TRAPPC5	0.000823	698
ENSP00000233616	MOGS	0.000823	580
ENSP00000281030	THRSP	0.000823	430
ENSP00000365877	SUV39H1	0.000822	663
ENSP00000352785	DSG4	0.000822	420
ENSP00000373485	TSNAXIP1	0.000822	164
ENSP00000265310	TRPV5	0.000821	563
ENSP00000420418	ZNF398	0.00082	243
ENSP00000363431	NPY4R	0.000818	899
ENSP00000364246	PLA2G2D	0.000817	899
ENSP00000219409	ARHGDIG	0.000811	899
ENSP00000238256	FKBP15	0.000809	378
ENSP00000304604	MAGI3	0.000809	646
ENSP00000267842	SLC27A2	0.000796	478
ENSP00000257829	NAT10	0.000461	819
ENSP00000222212	CACNG7	0.000154	768
ENSP00000276533	GINS4	0.000105	394
ENSP00000292301	CCR2	0.000105	970
ENSP00000339428	SOCS2	6.29E-05	817
ENSP00000367959	HTR2A	4.12E-05	908
ENSP00000357453	MAN1A1	2.99E-05	517
ENSP00000220478	SCG3	2.47E-05	750
ENSP00000324944	MBOAT1	1.85E-05	899
ENSP00000383295	NBEA	1.75E-05	210
ENSP00000302397	ATP1A3	1.34E-05	904
ENSP00000384169	FBLN2	1.24E-05	619
ENSP00000356652	CACYBP	9.27E-06	899
ENSP00000283415	LPCAT1	9.27E-06	412
ENSP00000381634	SLC38A1	8.24E-06	463
ENSP00000352834	MYO1C	7.21E-06	745
ENSP00000280155	ADRA2A	6.18E-06	907
ENSP00000394382	PDHA1	6.18E-06	964

ENSP00000285039	MYO5B	6.18E-06	563
ENSP00000323880	FOXJ1	6.18E-06	669
ENSP00000355731	CDC42BPA	6.18E-06	379
ENSP00000356951	C1orf192	5.15E-06	317
ENSP00000203556	GMIP	5.15E-06	677
ENSP00000332504	CCR10	5.15E-06	820
ENSP00000291536	RSPH1	5.15E-06	458
ENSP00000327349	TMCC1	5.15E-06	515
ENSP00000327916	ACSM5	5.15E-06	230
ENSP00000241463	RASL11A	5.15E-06	356
ENSP00000319851	CHDH	5.15E-06	338
ENSP00000350012	ACSL3	5.15E-06	270
ENSP00000363727	STARD8	4.12E-06	827
ENSP00000408405	KCTD1	4.12E-06	430
ENSP00000355963	LPGAT1	4.12E-06	371
ENSP00000364685	MFAP2	4.12E-06	859
ENSP00000341151	ZNF81	4.12E-06	522
ENSP00000316476	DEGS1	4.12E-06	899
ENSP00000261407	LPCAT3	4.12E-06	430
ENSP00000261483	MAN2A1	4.12E-06	462
ENSP00000330005	RGMA	4.12E-06	902
ENSP00000273258	ARL6IP5	3.09E-06	459
ENSP00000195654	DOPEY1	3.09E-06	347
ENSP00000245934	SYMPK	3.09E-06	538
ENSP00000406674	MOGAT1	3.09E-06	253
ENSP00000361236	RSPH9	3.09E-06	449
ENSP00000407497	SPINK8	3.09E-06	382
ENSP00000219091	ZNF205	3.09E-06	341
ENSP00000288139	CACNA1D	3.09E-06	374
ENSP00000306407	DBNDD1	3.09E-06	430
ENSP00000346550	ANXA6	3.09E-06	800
ENSP00000222250	ARRDC2	3.09E-06	379
ENSP00000253669	HAUS8	3.09E-06	657

ENSP00000362298	SGPL1	3.09E-06	902
ENSP00000261047	GUCA1C	3.09E-06	0
ENSP00000196169	TDRD3	2.06E-06	384
ENSP00000297784	TMC1	2.06E-06	734
ENSP00000338217	ZNF532	2.06E-06	465
ENSP00000344140	PPP1R32	2.06E-06	376
ENSP00000360065	WDR78	2.06E-06	305
ENSP00000363162	ATP6V1G1	2.06E-06	0
ENSP00000258111	KCNMB4	2.06E-06	899
ENSP00000329748	CPNE8	2.06E-06	330
ENSP00000335612	SPATA21	2.06E-06	271
ENSP00000355927	RPS6KC1	2.06E-06	227
ENSP00000369003	TRPC4	2.06E-06	688
ENSP00000335388	KIR3DX1	2.06E-06	424
ENSP00000332812	PTGDR2	2.06E-06	912
ENSP00000355045	FAM179B	2.06E-06	428
ENSP00000385025	SMCR8	2.06E-06	534
ENSP00000359819	MOSPD1	2.06E-06	460
ENSP00000220244	KIAA1199	2.06E-06	235
ENSP00000406219	PPT2	2.06E-06	899
ENSP00000273347	NXPE3	2.06E-06	430
ENSP00000344431	DNAJB13	2.06E-06	437
ENSP00000348349	MYO9A	2.06E-06	319
ENSP00000249363	LRRC4	2.06E-06	274
ENSP00000384179	ZFPM2	2.06E-06	968
ENSP00000363229	SLC18A3	2.06E-06	517
ENSP00000407674	HLA-DPB1	2.06E-06	363
ENSP00000331474	SYNDIG1L	2.06E-06	426
ENSP00000316794	ZCCHC5	2.06E-06	427
ENSP00000258711	CHST12	2.06E-06	306
ENSP00000261622	SLC7A5	2.06E-06	444
ENSP00000260382	LRRC49	2.06E-06	260
ENSP00000322427	ZNF611	2.06E-06	272

ENSP00000205636	CMTM6	2.06E-06	251
ENSP00000311313	CST6	2.06E-06	340
ENSP00000361507	HYI	2.06E-06	899
ENSP00000299140	SPATA19	2.06E-06	174
ENSP00000248958	SDF2L1	1.03E-06	257
ENSP00000322791	KIF1A	1.03E-06	373
ENSP00000330509	DEXI	1.03E-06	198
ENSP00000349336	FAM163B	1.03E-06	411
ENSP00000361681	SLC25A53	1.03E-06	307
ENSP00000389427	CEP44	1.03E-06	319
ENSP00000269703	CYP4F22	1.03E-06	621
ENSP00000278772	ZNF343	1.03E-06	279
ENSP00000358853	SH3BGRL2	1.03E-06	245
ENSP00000384700	PAPOLB	1.03E-06	567
ENSP00000175091	LAPTM4A	1.03E-06	388
ENSP00000250113	FXR2	1.03E-06	401
ENSP00000266579	SLC38A4	1.03E-06	408
ENSP00000314810	EDDM3B	1.03E-06	273
ENSP00000318753	WFDC11	1.03E-06	425
ENSP00000415026	PRRT4	1.03E-06	340
ENSP00000216327	ABHD4	1.03E-06	302
ENSP00000257776	MRAP2	1.03E-06	319
ENSP00000406027	EPM2AIP1	1.03E-06	412
ENSP00000256649	TRIM45	1.03E-06	752
ENSP00000357540	FUOM	1.03E-06	429
ENSP00000221086	MTMR9	1.03E-06	792
ENSP00000320303	DNAJC28	1.03E-06	196
ENSP00000328426	FAM208B	1.03E-06	288
ENSP00000340672	ARMCX3	1.03E-06	241
ENSP00000351608	PRRT2	1.03E-06	462
ENSP00000228567	SYT10	1.03E-06	305
ENSP00000398930	SGCE	1.03E-06	609
ENSP00000407818	USP46	1.03E-06	659

ENSP00000254712	INPP5K	1.03E-06	908
ENSP00000325123	ZSCAN2	1.03E-06	330
ENSP00000327453	ACSM2B	1.03E-06	205
ENSP00000336888	SLC44A2	1.03E-06	347
ENSP00000261722	AP3B2	1.03E-06	253
ENSP00000300527	COL6A2	1.03E-06	977
ENSP00000326200	ZBTB11	1.03E-06	302
ENSP00000352413	TMC3	1.03E-06	380
ENSP00000298596	STOX1	1.03E-06	412
ENSP00000285238	ABCC3	1.03E-06	340
ENSP00000288462	C9orf43	1.03E-06	379
ENSP00000316329	SCD5	1.03E-06	285
ENSP00000248706	RASL11B	1.03E-06	219
ENSP00000322020	SLC25A22	1.03E-06	517
ENSP00000323087	WSCD1	1.03E-06	200
ENSP00000297290	BRI3	1.03E-06	480
ENSP00000389813	ACAD10	1.03E-06	344
ENSP00000255305	XPO4	1.03E-06	235
ENSP00000295727	FEV	1.03E-06	625
ENSP00000365160	NUDT11	1.03E-06	367
ENSP00000382713	UBE2QL1	1.03E-06	347
ENSP00000270001	ZFP14	1.03E-06	424
ENSP00000352936	SPINK5	1.03E-06	524
ENSP00000359505	C6orf57	1.03E-06	659
ENSP00000364700	OMD	1.03E-06	912
ENSP00000369344	GPR150	1.03E-06	461
ENSP00000328397	VMO1	1.03E-06	374
ENSP00000365426	GGACT	1.03E-06	200
ENSP00000299333	SCN3B	1.03E-06	598
ENSP00000299578	C16orf46	1.03E-06	177
ENSP00000332171	DMTF1	1.03E-06	373
ENSP00000369292	SAMD9	1.03E-06	319
ENSP00000332674	LRRC8B	1.03E-06	317

ENSP00000229729	SLC44A4	1.03E-06	318
ENSP00000328983	CHST6	1.03E-06	907
ENSP00000286760	WHAMM	1.03E-06	319
ENSP00000375907	PID1	1.03E-06	378
ENSP00000298292	DNAAF2	1.03E-06	334
ENSP00000255194	AP3B1	1.03E-06	619
ENSP00000267116	ANKRD52	1.03E-06	302
ENSP00000226413	GNRHR	1.03E-06	927
ENSP00000333019	ADSSL1	1.03E-06	899
ENSP00000337396	CCNB1IP1	1.03E-06	350
ENSP00000276185	FRMPD3	1.03E-06	534
ENSP00000341963	RSC1A1	1.03E-06	318
ENSP00000404524	HSPA1A	1.03E-06	911
ENSP00000261326	MOCOS	1.03E-06	430
ENSP00000367125	GPR153	1.03E-06	530
ENSP00000400513	FASTKD1	1.03E-06	301
ENSP00000405738	ESRP1	1.03E-06	274
ENSP00000315137	SGPP2	1.03E-06	917
ENSP00000340083	KRCC1	1.03E-06	270
ENSP00000339390	CDH26	1.03E-06	364
ENSP00000379203	ATP6V1C1	1.03E-06	0
ENSP00000278499	SESN3	1.03E-06	215
ENSP00000330289	TRAPPC6B	1.03E-06	291
ENSP00000359719	PRKACB	1.03E-06	907
ENSP00000017003	XYLT2	1.03E-06	369
ENSP00000298310	NEMF	1.03E-06	429
ENSP00000355560	TBCE	1.03E-06	383
ENSP00000356433	UST	1.03E-06	659
ENSP00000244625	TBCC	1.03E-06	329
ENSP00000328938	AFMID	1.03E-06	430
ENSP00000365756	KIAA2013	1.03E-06	241

6. Candidate genes for somatic mutation genes and mRNA genes

Ensembl ID	Gene symbol	Betweenness ratio	Min-Max interaction score
ENSP00000264033	CBL	0.190351	990
ENSP00000338018	HIF1A	0.096934	994
ENSP00000344456	CTNNB1	0.095998	996
ENSP00000350941	SRC	0.075733	999
ENSP00000256474	VHL	0.071464	771
ENSP00000335153	HSP90AA1	0.058321	979
ENSP00000357656	FYN	0.057238	981
ENSP00000297494	NOS3	0.050043	563
ENSP00000349467	CALM1	0.047988	987
ENSP00000277541	NOTCH1	0.047776	948
ENSP00000293379	ITGA5	0.046361	835
ENSP00000266970	CDK2	0.041833	999
ENSP00000401303	SHC1	0.040253	967
ENSP00000351486	NTRK1	0.039364	550
ENSP00000296585	ITGA2	0.039317	959
ENSP00000262613	SLC9A3R1	0.035281	940
ENSP00000338934	EZR	0.033434	950
ENSP00000312435	DAG1	0.03314	985
ENSP00000251849	RAF1	0.03131	974
ENSP00000358525	NGF	0.030524	947
ENSP00000242577	DYNLL1	0.029117	803
ENSP00000314458	CDC42	0.028155	975
ENSP00000309503	YWHAZ	0.028099	930
ENSP00000329380	GP1BA	0.027438	948
ENSP00000265335	RAD50	0.026415	899
ENSP00000332353	PTCH1	0.026049	430
ENSP00000361125	VEGFA	0.025578	994
ENSP00000358022	MCL1	0.02282	752
ENSP00000254942	TERF2	0.022457	899
ENSP00000359074	L1CAM	0.022259	900
ENSP00000371138	FKBP1A	0.021935	401

ENSP00000304669	CTNNA1	0.021754	944
ENSP00000387662	GCG	0.019246	929
ENSP00000308541	F2	0.01919	997
ENSP00000265709	ANK1	0.017222	951
ENSP00000261769	CDH1	0.01714	899
ENSP00000228307	PXN	0.016889	702
ENSP00000297268	COL1A2	0.016449	993
ENSP00000300574	CRK	0.015146	975
ENSP00000351209	EPHA2	0.014434	953
ENSP00000265171	EGF	0.014071	998
ENSP00000162330	BCAR1	0.014023	593
ENSP00000288986	NCK1	0.013933	961
ENSP00000268171	FURIN	0.013704	723
ENSP00000223095	SERPINE1	0.013506	953
ENSP00000297261	SHH	0.012698	985
ENSP00000345206	RBPJ	0.01266	925
ENSP00000300134	STAT6	0.012224	878
ENSP00000276603	TERF1	0.012068	928
ENSP00000352514	RUNX2	0.011891	872
ENSP00000256443	CDK7	0.01111	966
ENSP00000322898	EBF1	0.010471	909
ENSP00000361850	PLAU	0.010415	941
ENSP00000340698	GIPC1	0.010398	983
ENSP00000267415	TINF2	0.010316	899
ENSP00000354586	GLI2	0.010178	512
ENSP00000330633	CNTN2	0.00994	462
ENSP00000380227	ITGA4	0.009828	873
ENSP00000312999	GNAI2	0.009064	907
ENSP00000354541	NLGN1	0.008339	540
ENSP00000308176	BTK	0.008335	942
ENSP00000266376	CACNA1C	0.008054	901
ENSP00000360916	VAV2	0.007959	965
ENSP00000311113	JUP	0.007752	994

ENSP00000380942	ARHGEF12	0.0077	439
ENSP00000265371	NRP1	0.007661	887
ENSP00000357392	EFNA1	0.007631	947
ENSP00000377958	CCT4	0.007562	265
ENSP00000281708	FBXW7	0.0071	945
ENSP00000348786	RAP1A	0.006988	366
ENSP00000244289	LIPE	0.00691	505
ENSP00000390849	ABHD5	0.00688	899
ENSP00000223642	C5	0.006871	899
ENSP00000300055	PLIN1	0.006871	369
ENSP00000346294	S100A4	0.006871	918
ENSP00000295709	STK36	0.006833	213
ENSP00000001008	FKBP4	0.006833	371
ENSP00000369129	DSP	0.006828	869
ENSP00000347198	SRGAP1	0.006794	245
ENSP00000261783	ARG2	0.006746	280
ENSP00000316460	FYB	0.006591	562
ENSP00000386896	ITGA6	0.006207	922
ENSP00000245323	EFNB2	0.00609	900
ENSP00000374372	SPTB	0.006073	902
ENSP00000370408	CDX2	0.006056	911
ENSP00000242839	ATP7B	0.006051	778
ENSP00000200181	ITGB4	0.006051	925
ENSP00000301200	CDC42EP5	0.006047	229
ENSP00000316854	ATOX1	0.006047	368
ENSP00000377941	ACTN1	0.006038	899
ENSP00000380349	CAPN3	0.006	659
ENSP00000339861	ENY2	0.005995	210
ENSP00000291688	MCM3AP	0.005995	0
ENSP00000267845	HDC	0.005982	679
ENSP00000400717	GNA13	0.005978	800
ENSP00000366563	PIK3CD	0.005935	946
ENSP00000319060	CAMK2G	0.005857	946

ENSP00000338785	STARD13	0.005723	573
ENSP00000262188	SMARCD3	0.005723	899
ENSP00000254351	SDC1	0.005426	942
ENSP00000260130	SDCBP	0.005408	179
ENSP00000344468	SDC3	0.005404	929
ENSP00000322542	GTF2I	0.005305	738
ENSP00000358994	MYO6	0.005287	899
ENSP00000265071	CDH6	0.005253	902
ENSP00000382193	MYBPC3	0.005253	937
ENSP00000417864	ANP32A	0.005249	228
ENSP00000360200	INADL	0.005236	899
ENSP00000372313	MSLN	0.005236	530
ENSP00000312185	ELMO1	0.005236	905
ENSP00000367714	HES5	0.005236	609
ENSP00000233813	IGFBP5	0.005236	811
ENSP00000360310	SPO11	0.005236	925
ENSP00000303522	TACR1	0.005236	899
ENSP00000358323	TXNIP	0.005236	809
ENSP00000328777	EFNA5	0.005236	908
ENSP00000225698	C1QBP	0.005236	778
ENSP00000363360	INIP	0.005231	424
ENSP00000286621	ADK	0.005231	893
ENSP00000379457	FAF1	0.005231	352
ENSP00000258341	LAMC1	0.005231	811
ENSP00000361777	SET	0.005231	679
ENSP00000372547	SRY	0.005231	573
ENSP00000372160	DOK6	0.005227	609
ENSP00000383210	NEK3	0.005223	429
ENSP00000309595	C10orf2	0.005223	778
ENSP00000341170	PTN	0.005223	552
ENSP00000360689	TNKS2	0.005223	609
ENSP00000337397	DKFZP686J19100	0.005223	196
ENSP00000345656	VAPA	0.005223	350

ENSP00000367486	MEIG1	0.005218	407
ENSP00000332592	SPAG16	0.005218	357
ENSP00000352657	ME3	0.005214	412
ENSP00000348634	MYH6	0.005214	726
ENSP00000385450	MAGI1	0.005214	734
ENSP00000225893	HNF1B	0.005214	804
ENSP00000344742	STAMBP	0.005214	660
ENSP00000366746	STAM	0.005214	903
ENSP00000252456	CNN1	0.00521	659
ENSP00000265362	SEMA3A	0.005205	507
ENSP00000283875	GTF2E1	0.005201	210
ENSP00000356641	RFWD2	0.005197	845
ENSP00000228841	MYL2	0.005192	956
ENSP00000261681	MPP5	0.005184	726
ENSP00000349204	CRB3	0.005184	429
ENSP00000362058	NDUFS5	0.005179	196
ENSP00000348168	GTF2E2	0.005158	326
ENSP00000262053	ATF1	0.005132	180
ENSP00000222399	LAMB1	0.005119	815
ENSP00000361965	ADA	0.005106	399
ENSP00000291525	TFF3	0.005085	838
ENSP00000286548	GNAQ	0.004994	948
ENSP00000348089	ERCC6	0.004964	351
ENSP00000268182	IQGAP1	0.004588	932
ENSP00000309103	BAD	0.002741	619
ENSP00000281821	EPHA4	0.002495	903
ENSP00000359285	CHRNA4	0.002465	379
ENSP00000201586	SULT2B1	0.00246	427
ENSP00000161559	CEACAM1	0.00246	674
ENSP00000291386	SSU72	0.002443	0
ENSP00000372815	C4A	0.002434	0
ENSP00000386165	CEBPD	0.002413	350
ENSP00000276420	DOK2	0.002387	629

ENSP00000005226	USH1C	0.001696	884
ENSP00000304767	P2RY1	0.001653	899
ENSP00000262776	LGALS3BP	0.001649	160
ENSP00000234739	BCL9	0.001649	0
ENSP00000264234	UPK1B	0.001649	173
ENSP00000264126	GPSM2	0.001649	545
ENSP00000353408	MSN	0.001649	822
ENSP00000261037	COL8A1	0.001649	962
ENSP00000340088	THEG	0.001649	267
ENSP00000254301	LGALS3	0.001649	844
ENSP00000350162	SYCP2	0.001649	899
ENSP00000283147	BMP6	0.001644	318
ENSP00000231524	TRIM23	0.001644	193
ENSP00000393887	AHSG	0.001644	802
ENSP00000328968	SCN5A	0.001644	752
ENSP00000323568	SLC2A2	0.001644	545
ENSP00000260630	CYP1B1	0.00164	505
ENSP00000199764	CEACAM6	0.001636	388
ENSP00000331902	COL4A5	0.001632	918
ENSP00000350256	CCR9	0.001627	159
ENSP00000413720	CDKN1C	0.001627	514
ENSP00000247170	DAAM1	0.001627	206
ENSP00000225844	CCL13	0.001627	465
ENSP00000019103	SCTR	0.001623	906
ENSP00000323280	CD6	0.001619	271
ENSP00000305988	ALCAM	0.001619	442
ENSP00000357721	S100A8	0.001619	913
ENSP00000248996	GNAZ	0.001606	899
ENSP00000241256	GHSR	0.001472	899
ENSP00000303634	LRP8	0.000868	901
ENSP00000362095	SRPX2	0.000846	798
ENSP00000369962	IGSF5	0.000846	167
ENSP00000292169	S100A1	0.000846	785

ENSP00000298032	ARMC3	0.000842	224
ENSP00000356975	ADAMTS4	0.000842	583
ENSP00000168712	FGF4	0.000837	859
ENSP00000378326	ZP3	0.000833	841
ENSP00000350937	TES	0.000829	637
ENSP00000260600	ADCY3	0.000824	164
ENSP00000222792	CHN2	0.000824	170
ENSP00000303077	GOT1L1	0.000824	0
ENSP00000368450	CD83	0.000824	609
ENSP00000300417	LRSAM1	0.000824	430
ENSP00000316990	TRAPPC5	0.000824	0
ENSP00000328364	MAFA	0.000824	195
ENSP00000264009	HSF4	0.000824	204
ENSP00000310216	KLRC4	0.000824	154
ENSP00000333920	TTF1	0.000824	180
ENSP00000354900	GJB1	0.000824	354
ENSP00000377840	CACNB1	0.000824	803
ENSP00000221283	STXBP2	0.000824	0
ENSP00000265310	TRPV5	0.000824	506
ENSP00000310658	SCUBE2	0.000824	0
ENSP00000352785	DSG4	0.000824	238
ENSP00000355001	POU3F3	0.000824	369
ENSP00000357025	CD48	0.000824	361
ENSP00000358576	DCLRE1B	0.000824	167
ENSP00000362372	BRWD3	0.000824	399
ENSP00000369009	CXorf23	0.000824	335
ENSP00000395465	NCOA4	0.000824	185
ENSP00000216373	SOS2	0.000824	899
ENSP00000225941	ABI3	0.000824	286
ENSP00000233202	SLC11A1	0.000824	430
ENSP00000233616	MOGS	0.000824	272
ENSP00000263640	ACVR1	0.000824	196
ENSP00000268704	SPG7	0.000824	813

ENSP00000283871	HGD	0.000824	765
ENSP00000352442	HIST1H2BM	0.000824	0
ENSP00000359594	CLCA4	0.000824	430
ENSP00000365877	SUV39H1	0.000824	281
ENSP00000005587	SKAP2	0.000824	197
ENSP00000248071	KLF2	0.000824	250
ENSP00000252593	BST2	0.000824	221
ENSP00000295728	CRYBA2	0.000824	157
ENSP00000266659	GLIPR1	0.000824	428
ENSP00000252050	CUL9	0.000824	722
ENSP00000274793	PLA2G7	0.000824	542
ENSP00000281030	THRSP	0.000824	407
ENSP00000298386	RXFP2	0.000824	904
ENSP00000392828	GPSM1	0.000824	820
ENSP00000262545	PCSK2	0.000824	233
ENSP00000265294	GABRP	0.000824	374
ENSP00000297135	COG5	0.000824	0
ENSP00000302114	PRELID1	0.000824	0
ENSP00000350332	MYBPC2	0.000824	937
ENSP00000301420	KLK1	0.000824	229
ENSP00000361867	SEMG1	0.000824	215
ENSP00000329757	ATP6V0C	0.000824	274
ENSP00000340237	SH3BP4	0.000824	202
ENSP00000374981	IGHA2	0.000824	385
ENSP00000259938	CLPS	0.000824	184
ENSP00000341815	SOX18	0.000824	282
ENSP00000311219	TRIM59	0.000824	432
ENSP00000240093	FZD3	0.000824	800
ENSP00000260643	PREB	0.000824	318
ENSP00000261170	GUCY2C	0.000824	563
ENSP00000332247	ATP6V0A2	0.000824	428
ENSP00000278823	MTA2	0.000824	868
ENSP00000343785	SPRY1	0.000824	912

ENSP00000355896	TGFB2	0.000824	899
ENSP00000273390	MAATS1	0.000824	0
ENSP00000294973	HAAO	0.000824	229
ENSP00000289429	CD1A	0.000824	0
ENSP00000389792	DCDC1	0.000824	467
ENSP00000401632	GSTT1	0.000824	540
ENSP00000206474	HAUS4	0.000824	0
ENSP00000226091	EFNB3	0.000824	915
ENSP00000247655	COX7C	0.000824	0
ENSP00000255945	GIMAP4	0.000824	326
ENSP00000311697	FGF5	0.000824	826
ENSP00000340396	GBP5	0.000824	351
ENSP00000365048	TNFSF13B	0.000824	534
ENSP00000258682	CAMK2B	0.000824	918
ENSP00000360054	PHACTR3	0.000824	0
ENSP00000408395	RBFOX3	0.000824	486
ENSP00000350869	ZNF346	0.000824	503
ENSP00000333496	KCND2	0.000824	238
ENSP00000353165	TPK1	0.000824	165
ENSP00000264634	WNT5A	0.000824	369
ENSP00000216064	SUN2	0.000824	901
ENSP00000305442	COG7	0.000824	0
ENSP00000220876	STMN2	0.000824	180
ENSP00000388724	HLA-A	0.000824	290
ENSP00000339521	RSU1	0.000824	339
ENSP00000289004	HPD	0.000824	571
ENSP00000370962	GGT6	0.000824	190
ENSP00000216277	PAPOLA	0.000824	431
ENSP00000216338	GZMH	0.000824	0
ENSP00000356056	DYNLT1	0.000824	803
ENSP00000257879	ITGA7	0.000824	921
ENSP00000274311	PELO	0.000824	186
ENSP00000314080	HIC1	0.000824	659

ENSP00000347409	KEL	0.000824	274
ENSP00000391457	INO80C	0.000824	352
ENSP00000264010	CTCF	0.000824	422
ENSP00000266427	ETV6	0.000824	507
ENSP00000290551	BTG2	0.000824	933
ENSP00000365569	FLOT1	0.000824	235
ENSP00000295006	CAPN2	0.000824	379
ENSP00000305913	COL8A2	0.000824	924
ENSP00000231751	LTF	0.000824	647
ENSP00000253571	RLIM	0.000824	329
ENSP00000260283	ARHGAP20	0.000824	241
ENSP00000259206	IL1RN	0.000824	627
ENSP00000364802	HSPA1A	0.000824	766
ENSP00000269468	MBD1	0.000824	205
ENSP00000329384	IL22	0.000824	824
ENSP00000278198	LRRC4C	0.000824	569
ENSP00000417164	ROBO2	0.000824	778
ENSP00000260950	MSTN	0.000824	559
ENSP00000358272	NDUFAF4	0.000824	0
ENSP00000281938	HSPB8	0.000824	171
ENSP00000357311	CENPW	0.000824	552
ENSP00000364246	PLA2G2D	0.00082	0
ENSP00000329968	PHKG2	0.00082	0
ENSP00000345096	IMPDH1	0.00082	522
ENSP00000327417	GPR39	0.00082	157
ENSP00000276072	TAF1	0.00082	818
ENSP00000301258	PSCA	0.00082	235
ENSP00000345793	ZC3H7B	0.00082	238
ENSP00000260570	IFT172	0.00082	340
ENSP00000315173	ZNF41	0.00082	621
ENSP00000265447	ANXA11	0.00082	299
ENSP00000420418	ZNF398	0.000816	0
ENSP00000219409	ARHGDIG	0.000811	250

ENSP00000363431	NPY4R	0.000811	899
ENSP00000375859	TMEM91	0.000807	196
ENSP00000238256	FKBP15	0.000807	0
ENSP00000365643	DOCK9	0.000807	264
ENSP00000304604	MAGI3	0.000807	619
ENSP00000267842	SLC27A2	0.000803	330
ENSP00000398852	SLC44A4	0.000803	196
ENSP00000331172	CD8B	0.000647	249
ENSP00000363390	TRIM63	0.000522	859
ENSP00000257068	MTNR1B	0.000306	899
ENSP00000222212	CACNG7	0.000164	899
ENSP00000419361	ADCY5	0.000112	904
ENSP00000304188	OR8U1	0.000104	460
ENSP00000271620	PRUNE	7.34E-05	714
ENSP00000312235	MUC13	4.75E-05	961
ENSP00000351790	MYPN	4.75E-05	944
ENSP00000276218	GPR119	4.32E-05	305
ENSP00000264039	GPC1	3.88E-05	938
ENSP00000347046	PDE5A	3.88E-05	912
ENSP00000356652	CACYBP	3.45E-05	899
ENSP00000296181	ITGB5	3.45E-05	835
ENSP00000397181	RGS4	3.02E-05	746
ENSP00000381822	CDH23	3.02E-05	515
ENSP00000323880	FOXJ1	2.59E-05	856
ENSP00000263666	PDZRN3	2.59E-05	366
ENSP00000401514	DNAH1	2.16E-05	738
ENSP00000319197	OR4K17	2.16E-05	465
ENSP00000020926	SYT13	1.73E-05	243
ENSP00000357986	PLEKHA1	1.73E-05	218
ENSP00000265080	RASGRF2	1.73E-05	903
ENSP00000352834	MYO1C	1.73E-05	899
ENSP00000352336	PLCG2	1.73E-05	913
ENSP00000317300	LPCAT4	1.73E-05	899

ENSP00000338512	AGTPBP1	1.29E-05	307
ENSP00000355316	GRM3	1.29E-05	619
ENSP00000360107	EFHC1	1.29E-05	659
ENSP00000400223	FJX1	1.29E-05	183
ENSP00000355731	CDC42BPA	1.29E-05	286
ENSP00000227495	ST3GAL4	1.29E-05	914
ENSP00000299367	C2	8.63E-06	388
ENSP00000329318	CECR6	8.63E-06	273
ENSP00000262219	ANXA13	8.63E-06	778
ENSP00000369346	ARSK	8.63E-06	219
ENSP00000368994	ARHGAP44	8.63E-06	0
ENSP00000363533	MDH1B	8.63E-06	157
ENSP00000353557	SLC35F1	8.63E-06	380
ENSP00000396774	MUC20	8.63E-06	915
ENSP00000359245	ABCA4	8.63E-06	516
ENSP00000362353	GLP1R	8.63E-06	904
ENSP00000356430	RGS18	8.63E-06	417
ENSP00000349320	CACNA2D1	8.63E-06	765
ENSP00000225298	UTP18	8.63E-06	388
ENSP00000253354	BPIFB1	4.32E-06	228
ENSP00000335038	VSTM2B	4.32E-06	305
ENSP00000337209	CCDC68	4.32E-06	313
ENSP00000339823	ZNF546	4.32E-06	348
ENSP00000349568	WDR96	4.32E-06	160
ENSP00000367164	PRR20A	4.32E-06	424
ENSP00000371932	TAS2R1	4.32E-06	552
ENSP00000177648	ALPK1	4.32E-06	229
ENSP00000216139	ACR	4.32E-06	366
ENSP00000300527	COL6A2	4.32E-06	967
ENSP00000325123	ZSCAN2	4.32E-06	270
ENSP00000313084	EGFLAM	4.32E-06	453
ENSP00000338217	ZNF532	4.32E-06	243
ENSP00000366477	C10orf112	4.32E-06	467

ENSP00000398930	SGCE	4.32E-06	813
ENSP00000298923	SLC6A5	4.32E-06	424
ENSP00000331678	PKP3	4.32E-06	241
ENSP00000331827	TNFAIP8L1	4.32E-06	235
ENSP00000375907	PID1	4.32E-06	319
ENSP00000391594	ERV3-1	4.32E-06	290
ENSP00000310878	KLF14	4.32E-06	305
ENSP00000352011	CACNA1G	4.32E-06	824
ENSP00000324651	TMEM108	4.32E-06	366
ENSP00000328983	CHST6	4.32E-06	200
ENSP00000339390	CDH26	4.32E-06	266
ENSP00000352177	ADAM29	4.32E-06	290
ENSP00000244625	TBCC	4.32E-06	262
ENSP00000355560	TBCE	4.32E-06	274
ENSP00000317224	SAMD4B	4.32E-06	249
ENSP00000328422	PP13439	4.32E-06	609
ENSP00000346537	SMOC2	4.32E-06	534
ENSP00000348349	MYO9A	4.32E-06	899
ENSP00000383219	KRTAP10-6	4.32E-06	530
ENSP00000333097	FIGLA	4.32E-06	540
ENSP00000364946	MKX	4.32E-06	306
ENSP00000345824	BZRAP1	4.32E-06	305
ENSP00000340328	NYX	4.32E-06	200
ENSP00000203556	GMIP	4.32E-06	170
ENSP00000335203	ATPIF1	4.32E-06	899
ENSP00000266682	SLC6A15	4.32E-06	424
ENSP00000363799	ACTL7B	4.32E-06	276
ENSP00000389244	SLC44A4	4.32E-06	196
ENSP00000286657	ADAMTS3	4.32E-06	655
ENSP00000374323	EPHA6	4.32E-06	903
ENSP00000326340	ATG16L2	4.32E-06	329
ENSP00000357066	ARG1	4.32E-06	393
ENSP00000364685	MFAP2	4.32E-06	859

ENSP00000262764	PGS1	4.32E-06	0
ENSP00000286604	UGT2A1	4.32E-06	200
ENSP00000363163	NLGN3	4.32E-06	716
ENSP00000292174	CXCR5	4.32E-06	899

Supplementary Material IV. Detailed information of candidate genes filtered by further selection.

1. Candidate genes for methylation CpG site genes and microRNA target genes

Ensembl ID	Gene symbol	Betweenness	Permutation FDR	Betweenness ratio	Min-Max interaction score
ENSP00000344456	CTNNB1	9830	0.006	0.091698	996
ENSP00000263253	EP300	8814	0.003	0.08222	999
ENSP00000264657	STAT3	8193	0.015	0.076427	814
ENSP00000306245	FOS	7888	0.001	0.073582	940
ENSP00000352262	MLL	7342	<0.001	0.068489	988
ENSP00000337088	MEN1	7269	<0.001	0.067808	719
ENSP00000357656	FYN	5938	0.023	0.055392	980
ENSP00000312652	LEP	4184	0.016	0.03903	926
ENSP00000340858	B2M	3999	0.016	0.037304	919
ENSP00000342215	KIR2DL3	3827	0.023	0.0357	429

ENSP00000387662	GCG	3370	0.007	0.031437	973
ENSP00000332353	PTCH1	3044	0.001	0.028396	429
ENSP00000297261	SHH	2800	0.001	0.026119	986
ENSP00000262965	TCF3	2651	<0.001	0.024729	985
ENSP00000351407	ARNT	2622	0.015	0.024459	948
ENSP00000405890	PBX1	2529	<0.001	0.023591	822
ENSP00000361423	ABL1	2071	0.008	0.019319	859
ENSP00000339328	PLAUR	1730	0.002	0.016138	899
ENSP00000222725	LFNG	1727	0.001	0.01611	504
ENSP00000300574	CRK	1621	0.035	0.015121	644
ENSP00000365682	TLE1	1598	0.001	0.014907	953
ENSP00000403005	EFNA4	1597	<0.001	0.014897	679
ENSP00000287934	FZD1	1595	0.004	0.014879	967

ENSP00000358525	NGF	1572	0.045	0.014664	943
ENSP00000318472	NCAM1	1330	0.019	0.012407	906
ENSP00000331358	GAST	1284	0.008	0.011978	899
ENSP00000244741	CDKN1A	1254	0.027	0.011698	919

2. Candidate genes for methylation CpG site genes and somatic mutation genes

Ensembl ID	Gene symbol	Betweenness	Permutation FDR	Betweenness ratio	Min-Max interaction score
ENSP00000344456	CTNNB1	3240	<0.001	0.126592	996
ENSP00000206249	ESR1	3113	0.046	0.12163	967
ENSP00000350941	SRC	1965	0.036	0.076776	930
ENSP00000264657	STAT3	1898	0.031	0.074158	814
ENSP00000227507	CCND1	1813	0.039	0.070837	844
ENSP00000357656	FYN	1799	0.002	0.07029	980
ENSP00000306245	FOS	1790	0.002	0.069938	940
ENSP00000352262	MLL	1581	<0.001	0.061772	571
ENSP00000337088	MEN1	1567	<0.001	0.061225	719
ENSP00000277541	NOTCH1	1128	0.022	0.044073	925
ENSP00000312652	LEP	1064	0.02	0.041572	866

ENSP00000302665	IGF1	1010	0.017	0.039462	808
ENSP00000340858	B2M	953	0.028	0.037235	826
ENSP00000332353	PTCH1	931	<0.001	0.036376	429
ENSP00000387662	GCG	918	0.004	0.035868	929
ENSP00000358525	NGF	814	0.002	0.031804	953
ENSP00000296585	ITGA2	792	0.001	0.030945	565
ENSP00000308938	PLG	766	0.044	0.029929	427
ENSP00000361125	VEGFA	728	0.013	0.028444	818
ENSP00000297261	SHH	706	<0.001	0.027585	985
ENSP00000262965	TCF3	571	0.002	0.02231	420
ENSP00000242577	DYNLL1	544	0.04	0.021255	621
ENSP00000359074	L1CAM	527	0.004	0.020591	829
ENSP00000264708	POMC	511	0.038	0.019966	917
ENSP00000261769	CDH1	505	0.022	0.019731	825
ENSP00000162330	BCAR1	485	0.003	0.01895	593
ENSP00000332973	SMAD3	456	0.031	0.017817	695
ENSP00000300574	CRK	436	0.041	0.017035	644
ENSP00000300134	STAT6	431	0.041	0.01684	878
ENSP00000331358	GAST	419	0.006	0.016371	899

ENSP00000297268	COL1A2	400	0.003	0.015629	904
ENSP00000222725	LFNG	382	<0.001	0.014925	750
ENSP00000287934	FZD1	382	0.002	0.014925	813
ENSP00000339328	PLAUR	382	0.006	0.014925	899
ENSP00000403005	EFNA4	382	<0.001	0.014925	679
ENSP00000301838	FADD	321	0.028	0.012542	508
ENSP00000354541	NLGN1	315	0.004	0.012308	621
ENSP00000254480	SMARCC1	292	0.032	0.011409	734
ENSP00000293288	BAX	272	0.021	0.010627	906
ENSP00000268171	FURIN	268	0.011	0.010471	723
ENSP00000322898	EBF1	268	0.003	0.010471	959
ENSP00000361850	PLAU	261	0.025	0.010198	885

3. Candidate genes for methylation CpG site genes and mRNA genes

Ensembl ID	Gene symbol	Betweenness	Permutation FDR	Betweenness ratio	Min-Max interaction score
ENSP00000344456	CTNNB1	16234	0.001	0.099876	996
ENSP00000264657	STAT3	14590	0.001	0.089761	814
ENSP00000263253	EP300	14130	0.001	0.086931	995
ENSP00000306245	FOS	13726	<0.001	0.084446	940
ENSP00000227507	CCND1	11214	0.027	0.068991	844

ENSP00000352262	MLL	9758	<0.001	0.060034	567
ENSP00000337088	MEN1	9748	<0.001	0.059972	719
ENSP00000357656	FYN	9625	0.008	0.059215	980
ENSP00000312652	LEP	7481	<0.001	0.046025	926
ENSP00000340858	B2M	7379	0.001	0.045397	922
ENSP00000342215	KIR2DL3	7176	0.002	0.044149	424
ENSP00000407431	HLA-C	6929	0.003	0.042629	550
ENSP00000262320	AXIN1	5746	0.038	0.035351	852
ENSP00000387662	GCG	5517	0.004	0.033942	939
ENSP00000332353	PTCH1	5250	<0.001	0.032299	429
ENSP00000297261	SHH	4782	<0.001	0.02942	995
ENSP00000351407	ARNT	4772	0.002	0.029359	976
ENSP00000262965	TCF3	4274	<0.001	0.026295	987
ENSP00000405890	PBX1	3895	<0.001	0.023963	859
ENSP00000361125	VEGFA	3862	0.024	0.02376	818
ENSP00000264708	POMC	3827	0.009	0.023545	977
ENSP00000348986	INS-IGF2	3760	0.021	0.023132	942
ENSP00000303830	INSR	3549	0.027	0.021834	526
ENSP00000340944	PTPN11	3077	0.023	0.01893	643

ENSP00000222725	LFNG	2802	<0.001	0.017239	750
ENSP00000365682	TLE1	2693	<0.001	0.016568	683
ENSP00000339328	PLAUR	2687	0.003	0.016531	899
ENSP00000287934	FZD1	2551	0.002	0.015694	978
ENSP00000162330	BCAR1	2212	0.019	0.013609	654
ENSP00000261769	CDH1	2119	0.044	0.013037	825
ENSP00000320866	CALR	2011	0.045	0.012372	430
ENSP00000228837	FGF6	1989	0.015	0.012237	424
ENSP00000242152	NPY	1936	0.005	0.011911	984
ENSP00000331358	GAST	1911	0.008	0.011757	947
ENSP00000262186	KCNH2	1894	0.024	0.011652	953
ENSP00000410294	FGFR2	1750	0.007	0.010766	999
ENSP00000354541	NLGN1	1720	0.002	0.010582	540
ENSP00000368683	EDN1	1715	0.012	0.010551	987
ENSP00000338345	SNCA	1706	0.036	0.010496	828

4. Candidate genes for microRNA target genes and somatic mutation genes

**Ensembl ID**	**Gene symbol**	**Betweenness**	**Permutation FDR**	**Betweenness ratio**	**Min-Max interaction score**
ENSP00000206249	ESR1	18453	0.017	0.120766	997
ENSP00000350941	SRC	13874	<0.001	0.090798	999

ENSP00000344456	CTNNB1	13813	0.001	0.090399	996
ENSP00000335153	HSP90AA1	8635	0.021	0.056512	975
ENSP00000357656	FYN	8142	0.007	0.053285	994
ENSP00000339007	GRB2	7741	0.032	0.050661	999
ENSP00000297494	NOS3	7352	0.002	0.048115	563
ENSP00000349467	CALM1	6884	0.007	0.045052	969
ENSP00000401303	SHC1	6291	<0.001	0.041171	999
ENSP00000277541	NOTCH1	5845	0.018	0.038253	948
ENSP00000293379	ITGA5	5785	0.002	0.03786	835
ENSP00000262613	SLC9A3R1	5563	0.014	0.036407	895
ENSP00000338934	EZR	5459	0.008	0.035726	950
ENSP00000296585	ITGA2	5281	<0.001	0.034562	959
ENSP00000358525	NGF	4923	<0.001	0.032219	943
ENSP00000251849	RAF1	4700	0.037	0.030759	999
ENSP00000312435	DAG1	4162	0.001	0.027238	994
ENSP00000314458	CDC42	4161	0.028	0.027232	979
ENSP00000223023	WASL	3683	0.014	0.024103	914
ENSP00000265335	RAD50	3568	<0.001	0.023351	899
ENSP00000242577	DYNLL1	3547	0.008	0.023213	787

ENSP00000332353	PTCH1	3399	0.006	0.022245	430
ENSP00000304669	CTNNA1	3384	0.001	0.022147	944
ENSP00000348444	TTN(titin)	3248	<0.001	0.021257	504
ENSP00000359074	L1CAM	3182	0.001	0.020825	960
ENSP00000371138	FKBP1A	3140	<0.001	0.02055	998
ENSP00000361125	VEGFA	3113	0.026	0.020373	984
ENSP00000358022	MCL1	2927	0.03	0.019156	835
ENSP00000254942	TERF2	2914	0.001	0.019071	899
ENSP00000297268	COL1A2	2577	<0.001	0.016865	985
ENSP00000265709	ANK1	2403	0.001	0.015726	560
ENSP00000300134	STAT6	2303	0.011	0.015072	878
ENSP00000162330	BCAR1	2263	0.004	0.01481	593
ENSP00000351209	EPHA2	2152	0.003	0.014084	953
ENSP00000261769	CDH1	2136	0.043	0.013979	955
ENSP00000256443	CDK7	2108	0.005	0.013796	981
ENSP00000228307	PXN	2070	0.002	0.013547	702
ENSP00000345206	RBPJ	1886	0.024	0.012343	925
ENSP00000276603	TERF1	1790	0.001	0.011715	956
ENSP00000268171	FURIN	1787	0.001	0.011695	723

ENSP00000361850	PLAU	1785	0.002	0.011682	965
ENSP00000352514	RUNX2	1781	0.028	0.011656	872
ENSP00000340698	GIPC1	1739	0.001	0.011381	805
ENSP00000322898	EBF1	1599	0.001	0.010465	914
ENSP00000330633	CNTN2	1556	<0.001	0.010183	865

5. Candidate genes for microRNA target genes and mRNA genes

Ensembl ID	Gene symbol	Betweenness	Permutation FDR	Betweenness ratio	Min-Max interaction score
ENSP00000264033	CBL	164841	0.023	0.169869	990
ENSP00000206249	ESR1	101905	0.038	0.105013	998
ENSP00000344456	CTNNB1	60756	0.041	0.062609	999
ENSP00000263253	EP300	60273	<0.001	0.062112	995
ENSP00000350941	SRC	59670	0.026	0.06149	999
ENSP00000264657	STAT3	59543	0.01	0.061359	997
ENSP00000339007	GRB2	48282	<0.001	0.049755	939
ENSP00000357656	FYN	39355	0.028	0.040555	981
ENSP00000266970	CDK2	36082	0.009	0.037183	999
ENSP00000297494	NOS3	30057	0.047	0.030974	945
ENSP00000277541	NOTCH1	27278	0.039	0.02811	982
ENSP00000293379	ITGA5	25513	0.004	0.026291	964

ENSP00000312652	LEP	22731	0.046	0.023424	971
ENSP00000244007	PLCG1	22700	0.019	0.023392	969
ENSP00000401303	SHC1	22221	<0.001	0.022899	967
ENSP00000351486	NTRK1	20928	<0.001	0.021566	800
ENSP00000332353	PTCH1	17300	<0.001	0.017828	939
ENSP00000387662	GCG	15143	0.035	0.015605	939
ENSP00000223023	WASL	15013	0.026	0.015471	901
ENSP00000361125	VEGFA	14779	0.03	0.01523	984
ENSP00000278616	ATM	14772	0.036	0.015223	972
ENSP00000269571	ERBB2	14171	0.024	0.014603	916
ENSP00000306512	IL8	13870	0.027	0.014293	992
ENSP00000302269	VAV1	12687	0.006	0.013074	983
ENSP00000358525	NGF	12630	0.005	0.013015	943
ENSP00000296585	ITGA2	12251	0.002	0.012625	987
ENSP00000340944	PTPN11	11563	0.029	0.011916	988
ENSP00000361423	ABL1	11508	0.021	0.011859	989
ENSP00000344352	ATF3	10864	0.034	0.011195	889
ENSP00000297261	SHH	10778	<0.001	0.011107	986
ENSP00000162330	BCAR1	10607	<0.001	0.010931	986

ENSP00000354720	SMC3	10413	0.001	0.010731	986
ENSP00000302150	PRL	9777	0.015	0.010075	931

6. Candidate genes for somatic mutation genes and mRNA genes

Ensembl ID	Gene symbol	Betweenness	Permutation FDR	Betweenness ratio	Min-Max interaction score
ENSP00000264033	CBL	44101	0.015	0.190351	990
ENSP00000338018	HIF1A	22458	0.017	0.096934	994
ENSP00000344456	CTNNB1	22241	<0.001	0.095998	996
ENSP00000350941	SRC	17546	0.006	0.075733	999
ENSP00000256474	VHL	16557	0.009	0.071464	771
ENSP00000335153	HSP90AA1	13512	0.019	0.058321	979
ENSP00000357656	FYN	13261	0.003	0.057238	981
ENSP00000297494	NOS3	11594	0.001	0.050043	563
ENSP00000349467	CALM1	11118	0.001	0.047988	987
ENSP00000277541	NOTCH1	11069	<0.001	0.047776	948
ENSP00000293379	ITGA5	10741	<0.001	0.046361	835
ENSP00000266970	CDK2	9692	0.03	0.041833	999
ENSP00000401303	SHC1	9326	<0.001	0.040253	967
ENSP00000351486	NTRK1	9120	<0.001	0.039364	550
ENSP00000296585	ITGA2	9109	<0.001	0.039317	959

ENSP00000262613	SLC9A3R1	8174	0.026	0.035281	940
ENSP00000338934	EZR	7746	0.025	0.033434	950
ENSP00000312435	DAG1	7678	<0.001	0.03314	985
ENSP00000251849	RAF1	7254	0.027	0.03131	974
ENSP00000358525	NGF	7072	<0.001	0.030524	947
ENSP00000242577	DYNLL1	6746	<0.001	0.029117	803
ENSP00000314458	CDC42	6523	0.02	0.028155	975
ENSP00000309503	YWHAZ	6510	0.02	0.028099	930
ENSP00000329380	GP1BA	6357	0.014	0.027438	948
ENSP00000265335	RAD50	6120	<0.001	0.026415	899
ENSP00000332353	PTCH1	6035	0.003	0.026049	430
ENSP00000361125	VEGFA	5926	0.006	0.025578	994
ENSP00000358022	MCL1	5287	0.005	0.02282	752
ENSP00000254942	TERF2	5203	<0.001	0.022457	899
ENSP00000359074	L1CAM	5157	0.001	0.022259	900
ENSP00000371138	FKBP1A	5082	<0.001	0.021935	401
ENSP00000304669	CTNNA1	5040	0.001	0.021754	944
ENSP00000387662	GCG	4459	0.049	0.019246	929
ENSP00000308541	F2	4446	0.035	0.01919	997

ENSP00000265709	ANK1	3990	<0.001	0.017222	951
ENSP00000261769	CDH1	3971	0.016	0.01714	899
ENSP00000228307	PXN	3913	<0.001	0.016889	702
ENSP00000297268	COL1A2	3811	<0.001	0.016449	993
ENSP00000300574	CRK	3509	0.03	0.015146	975
ENSP00000351209	EPHA2	3344	0.004	0.014434	953
ENSP00000265171	EGF	3260	0.011	0.014071	998
ENSP00000162330	BCAR1	3249	0.007	0.014023	593
ENSP00000288986	NCK1	3228	0.004	0.013933	961
ENSP00000268171	FURIN	3175	<0.001	0.013704	723
ENSP00000223095	SERPINE1	3129	0.021	0.013506	953
ENSP00000297261	SHH	2942	0.025	0.012698	985
ENSP00000345206	RBPJ	2933	0.013	0.01266	925
ENSP00000300134	STAT6	2832	0.041	0.012224	878
ENSP00000276603	TERF1	2796	0.002	0.012068	928
ENSP00000352514	RUNX2	2755	0.03	0.011891	872
ENSP00000256443	CDK7	2574	0.027	0.01111	966
ENSP00000322898	EBF1	2426	0.001	0.010471	909
ENSP00000361850	PLAU	2413	0.008	0.010415	941

ENSP00000340698	GIPC1	2409	0.003	0.010398	983
ENSP00000267415	TINF2	2390	0.01	0.010316	899
ENSP00000354586	GLI2	2358	0.005	0.010178	512

Supplementary Material V. Detailed Analysis of candidate genes of two levels

For the levels of methylation diversity and microRNA expression abundance, tens of genes have been predicted to be related to both of the two levels. TCF3 (transcription factor 3) is a member of the E protein family of helix-loop-helix transcription factors. As a crucial transcriptional factor for lymphopoiesis, TCF3 has been proven to be up-regulated in several types of malignant neoplasms [1,2]. The alteration of the TCF3 methylation level has been clearly reported to be associated with Helicobacter pylori-related gastric cancer and lung adenocarcinoma, which directly validates our computational results [3,4]. Furthermore, TCF3 is not only regulated at the DNA level (methylation and demethylation) but on the transcriptional and translational levels as well. A group of microRNAs, such as microRNA-590, microRNA-17, and microRNA-148a, have all proven to have interactions with our predicted TCF3 gene and contribute to the tumorigenesis processes of their respective tumor subtypes including lung adenocarcinoma, indicating that an abundance of microRNA expression may participate in tumorigenesis via the regulation of such genes [5-7]. Such a result is also in accordance with our prediction, which implies that TCF3 is a specific gene which may be a cancer (lung adenocarcinoma) driver gene of at least two levels: methylation diversity and microRNA expression abundance. In addition to TCF3, MEN1 (multiple endocrine neoplasia I) is also a candidate gene on our prediction list. The epigenetic alterations of MEN1 are widely reported in various types of tumor cases, especially in endocrine neoplasms [8,9]. As a functional transcriptional activator, MEN1 contributes to methylation regulation via its specific histone modification functions, especially the process of histone H3 lysine 9 methylation, which is quite significant for the tumor genesis of lung adenocarcinoma[10,11]. MEN1 is also a functional target of microRNAs, which has been confirmed in diverse tumors, such as let-7a in pancreatic islet hyperplasia, MicroRNA-142-3p in osteosarcoma, and microRNA-17 in pancreatic cancer, indicating the significance of microRNA expression abundance in these tumor subtypes [12-14].As for lung adenocarcinoma, though no direct evidence, considering the similar function of microRNA-17 in lung tissues, our predicted gene may also contribute to lung adenocarcinoma [15]. Moreover, genes in our list, such as MLL, EFNA4, PBX1, and SHH, are all tumor-associated genes that have been reported to have variations in methylation level and can be regulated by specific microRNAs in specific tumor subtypes, including lung adenocarcinoma, verifying the accuracy of our algorithm [16-21]. Therefore, our predicted genes in this set have been proven to contribute to the tumorigenesis process at least two levels: methylation diversity and microRNA expression abundance.

As for the methylation diversity and the mutation differentiation in the tumor and the adjacent normal tissues, several genes have also been predicted to be related to both levels. TCF3, as mentioned above, contributes to the levels of methylation diversity and microRNA expression abundance. However, it also plays a crucial role in a third level, which turns out to be mutation diversity. Several mutations of TCF3 have been detected only in tumor tissues [22,23]. In addition to TCF3, MLL, MEN1 and SHH have all been predicted to be shared in these three levels. As mentioned above, MLL has been

reported to contribute to tumorigenesis on at least two levels: methylation diversity and microRNA expression abundance. Although MLL always contributes to specific diseases (including tumors) as a fusion gene, MLL has also been confirmed to contain functional mutational status in accordance with our prediction, which may be crucial for various processes during tumorigenesis, especially in lung adenocarcinoma[24-26]. Moreover, CTNNB1 (cadherin-associated protein beta 1) is a functional gene encoding the specific motif of cadherin-associated proteins, which further constitute adherens junctions (AJs) [27]. CTNNB1 has been reported to be regulated by various methylation statuses during tumorigenesis and may directly contribute to tumor metastasis[28,29]. In addition, the mutational status of CTNNB1 has been considered to directly connect to the basic functions of cadherin-associated proteins, a quite significant group of metastasis associated proteins in lung adeno carcinoma, which has been confirmed by experiments in vitro [30,31]. Genes such as LFNG, MLL, and ITGA2, are also candidate cancer driver genes at two levels in our prediction, which is in agreement with the existing literatures[24,32-34]. Overall, we predicted a set of crucial cancer driver genes which have all been proven to drive tumorigenesis on both the DNA methylation diversity level and the mutation differentiation level in lung adenocarcinoma, indicating the efficacy of our method.

The fourth level of cancer driver genes can be measured by specific mRNA sequencing data, which reflects the direct gene expression data of a candidate gene. Therefore, this level, corresponding to the mRNA level diversity between the tumor tissue and the adjacent normal tissue, is associated with the first level (methylation diversity). In addition to genes, such as MEN1, TCF3, and SHH, which have been analyzed above as candidate genes for the other three levels, there remains a group of crucial genes for cell metabolism which participate in tumorigenesis on the mRNA level. PTCH1 is a functional receptor for sonic hedgehog, which has been regarded as a crucial tumor suppressor in various tumor subtypes, including lung adenocarcinoma[35]. This gene has been proven to be regulated by methylation during tumorigenesis, especially in gastric cancers [36-38]. Furthermore, PTCH1 mutations have been regarded as frequent events in tumorigenesis, including the lung adenocarcinoma[39]. Specific tumor subtypes, such as sporadic keratocystic odontogenic tumor, childhood medulloblastoma, and oesophageal basaloid squamous cell carcinoma, have all been confirmed to be related to unique PTCH1 mutations, suggesting that such gene may also be crucial in lung adenocarcinoma [40-42]. Apart from PTCH1, TLE1 is another crucial cancer driver gene which has always been regarded as a transcriptional co-repressor that binds to a group of transcriptional factors [43,44]. Regulated by epigenetic modification, TLE1 has been confirmed to be a tumor-associated gene due to methylation alterations during lung adenocarcinoma and hepatic carcinoma initiation and proliferation[45,46]. The epigenetic inactivation of TLE1 may directly induce the expression differentiation of specific mRNA of TLE1, which is consistent with our computational results. Genes such as PBX1 and FOS are also on our list, which are also crucial lung adenocarcinoma driver genes on the methylation and expression levels, thus validating our algorithm[20,47-51]. The genes mentioned above are all multi-functional genes that contribute to tumorigenesis via various biological processes.

As mentioned above, some cancer driver genes may contribute to tumorigenesis via multiple biological processes and levels. Some of the candidate genes have also been predicted to be related to both microRNA expression differentiation and mutation diversity of malignant and somatic cells. Apart from those functional genes already mentioned above, there still remain a few genes which specifically contribute to these two levels. COL1A2 is a functional protein coding gene which participates in the formation of connective tissues especially in tumor tissues [52]. COL1A2 has been reported to be regulated by functional microRNAs (such as let-7) and may be associated with specific microRNAs, e.g., miR-29b, during the entire progression of tumorigenesis, especially during the metastasis process [53,54]. Furthermore, mutations in COL1A2 have been widely confirmed in heredofamilial tumor subtypes, such as hepatocellular carcinoma and lipoblastoma, indicating the mutation diversity of this cancer driver gene [55]. Although no in vivo experiments verify the functional role of COL1A2 during the tumor genesis of lung adenocarcinoma, it has been confirmed that such gene may definitely contribute to lung adenocarcinoma via similar ways in other tumor subtypes in vitro [56]. SHC1 is another candidate gene that can drive tumorigenesis on both levels. Encoding an adapter protein for the Ras signaling pathway, SHC1 is quite significant for the abnormal cell proliferation in the tumor microenvironment [57]. Furthermore, the abnormal epigenetic modification of SHC1 in the tumor microenvironment has been confirmed to be associated with cell proliferation and may further contribute to tumor metastasis in various tumor subtypes including lung adenocarcinoma[58-60]. MicroRNAs also affect the function of this gene. MiR-365 and miR-27b are both functional regulatory molecules of SHC1 in tumorigenesis, indicating that SHC1 is definitely a cancer driver gene at the microRNA expression differentiation level [61,62]. Apart from those two functional genes mentioned above, FKBP1A, TTN and NGF are also specific functional genes for the two levels [63-68]. As previously noted, the genes that we have predicted to contribute to these two levels have all been confirmed by existing results in recent literature, indicating that our method is valid for at least these two levels.

The fifth set of genes contains candidates that are functional cancer driver genes that induce tumorigenesis via both the microRNA expression differentiation level and the mRNA expression level. As we all know, microRNA can block the translation process of mRNA. Therefore, these two levels have specific internal relationships, which show that the genes we screened are actually functional and crucial genes for tumorigenesis. PTCH1 and SHH have all been proven to be associated with tumor initiation at multiple levels, as previously mentioned and can definitely be attributed to this cluster [35,38,40,69,70]. Furthermore, there are some new candidate genes in this list that have not been mentioned before. ITGA2 and ITGA5 are functional homologues that both encode subunits of a specific transmembrane receptor for collagens and related proteins [71,72]. MicroRNAs such as miR-29b and miR32 have been reported to participate in the abnormal metabolism of collagens in a tumor microenvironment and have specific interactions with ITGA-associated proteins including ITGA2 and ITGA5 [73-75]. Not limited to only ITGA2 and ITGA5, the alteration of the expression levels of ITGA families contribute to nearly every stage of tumorigenesis, especially in the metastasis process [76-78]. Apart from ITGA2 and ITGA5, GRB2 is also significant on our list. GRB2

is a growth factor receptor bound protein that may act as a critical link between cell surface growth factor receptors and the Ras signaling pathway, a functional pathway in lung adenocarcinoma[79,80]. Like ITGA2 and ITGA5, GRB2 is also regulated by specific microRNAs, such as miR-19b and miR-433, which may further contribute to the functional alteration of its downstream pathways including Ras [81-83]. Other predicted genes such as EP300 and SMC3 have also been confirmed by recent experimental results [84-87]. All of the genes mentioned above have been predicted by our method and confirmed by existing publications, indicating the efficacy of our method.

The last set of genes contributes to tumors on both the mutation and mRNA levels, which means the candidate genes not only obtain specific mutations but are up or down regulated during tumorigenesis. Apart from the genes we have analyzed above, e.g., ITGA2 and ITGA5, which certainly contain functional mutations, many new candidate genes are on our list, such as NOTCH1, PXN, and DYNLL1 [71,88]. NOTCH1 is a functional member of the NOTCH family that contributes to the NOTCH pathway, one of the key metastasis associated genes in lung adenocarcinoma [89]. Mutations in NOTCH1 have been widely identified in various tumor subtypes, especially in some non-solid tumor subtypes including lung adenocarcinoma and chronic lymphocytic leukemia[89-91]. In addition to mutations, the up and down regulation in NOTCH1 expression has been confirmed to be related to the progression of specific tumor subtypes, validating the accuracy of our algorithm. Moreover, another two genes, PXN and DYNLL1, are also functional genes that have been shown to be differentially expressed in tumor and normal tissues [92]. In addition to having diverse expression, PXN has also been shown to contain significant cancer driver mutations in various tumor subtypes, especially in lung cancer [93-95]. At the same time, DYNLL1, a crucial gene for presenting and processing MHC antigens, has been confirmed to have specific functions on tumorigenesis [96,97]. Both of these genes are not only differentially expressed but also polymorphic in tumor tissues and normal tissues, which directly support our prediction and thus validate our method.

References

1 Li, C., Cai, S., Wang, X. & Jiang, Z. Hypomethylation-associated up-regulation of TCF3 expression and recurrence in stage II and III colorectal cancer. *PLoS One* **9**, e112005, doi:10.1371/journal.pone.0112005 (2014).

2 Ge, M. H., Chen, C., Xu, J. J. & Ling, Z. Q. Critical regions and spreading of runt-related transcription factor-3 C-phosphate-G (CpG) island methylation in human salivary gland adenoid cystic carcinoma. *Human Pathology* **42**, 1862-1872, doi:10.1016/j.humpath.2011.02.003 (2011).

3 Nadarajan, N., Balasubramanian, L. K., Kuppannan, S., Ramachandran, C. & Leelakrishnan, V. Runt-related transcription factor 3: single nucleotide polymorphism rs760805, gene expression, and methylation status in Helicobacter pylori -infected patients for determination of gastric cancer risk. *Journal of gastrointestinal cancer* **44**, 444-449, doi:10.1007/s12029-013-9540-4 (2013).

4 Wei, S. S. *et al.* The Activating Transcription Factor 3 Protein Suppresses the Oncogenic

Function of Mutant p53 Proteins. *Journal of Biological Chemistry* **289**, 8947-8959, doi:10.1074/jbc.M113.503755 (2014).

5 Miranda, P. J., Vimalraj, S. & Selvamurugan, N. A feedback expression of microRNA-590 and activating transcription factor-3 in human breast cancer cells. *International journal of biological macromolecules* **72**, 145-150, doi:10.1016/j.ijbiomac.2014.07.051 (2015).

6 Zuo, J. B. *et al.* MicroRNA-148a can regulate runt-related transcription factor 3 gene expression via modulation of DNA methyltransferase 1 in gastric cancer. *Molecules and cells* **35**, 313-319, doi:10.1007/s10059-013-2314-9 (2013).

7 Akbari Moqadam, F., Boer, J. M., Lange-Turenhout, E. A., Pieters, R. & den Boer, M. L. Altered expression of miR-24, miR-126 and miR-365 does not affect viability of childhood TCF3-rearranged leukemia cells. *Leukemia* **28**, 1008-1014, doi:10.1038/leu.2013.308 (2014).

8 Chung, Y. J. *et al.* Genetic and epigenetic analysis in korean patients with multiple endocrine neoplasia type 1. *Endocrinology and metabolism* **29**, 270-279, doi:10.3803/EnM.2014.29.3.270 (2014).

9 Sulaiman, L. *et al.* Global and gene-specific promoter methylation analysis in primary hyperparathyroidism. *Epigenetics* **8**, 646-655, doi:10.4161/epi.24823 (2013).

10 Yang, Y. J. *et al.* Menin mediates epigenetic regulation via histone H3 lysine 9 methylation. *Cell Death Dis* **4**, e583, doi:10.1038/cddis.2013.98 (2013).

11 Lu, Y. J. *et al.* Lung cancer-associated JmjC domain protein mdig suppresses formation of tri-methyl lysine 9 of histone H3. *Cell cycle* **8**, 2101-2109, doi:DOI 10.4161/cc.8.13.8927 (2009).

12 Gurung, B., Muhammad, A. B. & Hua, X. X. Menin Is Required for Optimal Processing of the MicroRNA let-7a*. *Journal of Biological Chemistry* **289**, 9902-9908, doi:10.1074/jbc.M113.520692 (2014).

13 Yang, Y. Q., Qi, J., Xu, J. Q. & Hao, P. MicroRNA-142-3p, a novel target of tumor suppressor menin, inhibits osteosarcoma cell proliferation by down-regulation of FASN. *Tumor Biology* **35**, 10287-10293, doi:10.1007/s13277-014-2316-z (2014).

14 Lu, Y., Fei, X. Q., Yang, S. F., Xu, B. K. & Li, Y. Y. Glucose-induced microRNA-17 promotes pancreatic beta cell proliferation through down-regulation of Menin. *Eur Rev Med Pharmaco* **19**, 624-629 (2015).

15 Pullamsetti, S. S. *et al.* Inhibition of MicroRNA-17 Improves Lung and Heart Function in Experimental Pulmonary Hypertension. *Am J Resp Crit Care* **185**, 409-419, doi:10.1164/rccm.201106-1093OC (2012).

16 Chen, C. W. *et al.* DOT1L inhibits SIRT1-mediated epigenetic silencing to maintain leukemic gene expression in MLL-rearranged leukemia. *Nature medicine* **21**, 335-343, doi:10.1038/nm.3832 (2015).

17 Colamaio, M. *et al.* miR-142-3p Down-Regulation Contributes to Thyroid Follicular Tumorigenesis by Targeting ASH1L and MLL1. *Journal of Clinical Endocrinology & Metabolism* **100**, E59-E69, doi:10.1210/jc.2014-2280 (2015).

18 McKinney, N. *et al.* EphrinB1 expression is dysregulated and promotes oncogenic signaling in medulloblastoma. *Journal of neuro-oncology* **121**, 109-118, doi:10.1007/s11060-014-1618-8 (2015).

19 Yan, Y. *et al.* MicroRNA-10a Is Involved in the Metastatic Process by Regulating Eph Tyrosine Kinase Receptor A4-Mediated Epithelial-Mesenchymal Transition and Adhesion in Hepatoma Cells. *Hepatology* **57**, 667-677, doi:10.1002/hep.26071 (2013).

20 Cimmino, L. & Aifantis, I. Fingerprinting acute leukemia: DNA methylation profiling of B-acute lymphoblastic leukemia. *Cancer Discov* **2**, 976-978, doi:10.1158/2159-8290.CD-12-0435 (2012).
21 Marin-Muller, C. *et al.* A Tumorigenic Factor Interactome Connected through Tumor Suppressor MicroRNA-198 in Human Pancreatic Cancer. *Clinical Cancer Research* **19**, 5901-5913, doi:10.1158/1078-0432.CCR-12-3776 (2013).
22 Fischer, U. *et al.* Genomics and drug profiling of fatal TCF3-HLF-positive acute lymphoblastic leukemia identifies recurrent mutation patterns and therapeutic options. *Nat Genet* **47**, 1020-1029, doi:10.1038/ng.3362 (2015).
23 Diakos, C. *et al.* Direct and indirect targets of the E2A-PBX1 leukemia-specific fusion protein. *PLoS One* **9**, e87602, doi:10.1371/journal.pone.0087602 (2014).
24 Matveeva, E. *et al.* A new variant of KMT2A(MLL)-FLNA fusion transcript in acute myeloid leukemia with ins(X;11)(q28;q23q23). *Cancer genetics* **208**, 148-151, doi:10.1016/j.cancergen.2015.03.001 (2015).
25 Huang, D. *et al.* BRCC3 mutations in myeloid neoplasms. *Haematologica* **100**, 1051-1057, doi:10.3324/haematol.2014.111989 (2015).
26 Struski, S. *et al.* Identification of chromosomal loci associated with non-P-glycoprotein-mediated multidrug resistance to topoisomerase II inhibitor in lung adenocarcinoma cell line by comparative genomic hybridization. *Gene Chromosome Canc* **30**, 136-142, doi:Doi 10.1002/1098-2264(2000)9999:9999<::Aid-Gcc1071>3.0.Co;2-T (2001).
27 Hoggard, L. R. *et al.* Rational Design of Selective Small-Molecule Inhibitors for beta-Catenin/B-Cell Lymphoma 9 Protein-Protein Interactions. *J Am Chem Soc* **137**, 12249-12260, doi:10.1021/jacs.5b04988 (2015).
28 Zhang, S. *et al.* Pygopus-2 promotes invasion and metastasis of hepatic carcinoma cell by decreasing E-cadherin expression. *Oncotarget* **6**, 11074-11086 (2015).
29 Bujko, M. *et al.* Epigenetic-Mediated Downregulation of mu-Protocadherin in Colorectal Tumours. *Gastroenterology research and practice* **2015**, 317093, doi:10.1155/2015/317093 (2015).
30 Rubtsova, S. N., Zhitnyak, I. Y. & Gloushankova, N. A. A Novel Role of E-Cadherin-Based Adherens Junctions in Neoplastic Cell Dissemination. *PLoS One* **10**, e0133578, doi:10.1371/journal.pone.0133578 (2015).
31 Farmakovskaya, M. *et al.* E-Cadherin repression increases amount of cancer stem cells in human A549 lung adenocarcinoma and stimulates tumor growth. *Cell cycle* **15**, 1084-1092, doi:10.1080/15384101.2016.1156268 (2016).
32 Zhang, S. B., Chung, W. C., Miele, L. & Xu, K. L. Targeting Met and Notch in the Lfng-deficient, Met-amplified triple-negative breast cancer. *Cancer Biology & Therapy* **15**, 633-642, doi:10.4161/cbt.28180 (2014).
33 Andersson, A. K. *et al.* The landscape of somatic mutations in infant MLL-rearranged acute lymphoblastic leukemias. *Nat Genet* **47**, 330-U192, doi:10.1038/ng.3230 (2015).
34 Dmitriev, A. A. *et al.* Genetic and epigenetic analysis of non-small cell lung cancer with NotI-microarrays. *Epigenetics* **7**, 502-513, doi:10.4161/epi.19801 (2012).
35 Wu, X. *et al.* Melittin induces PTCH1 expression by down-regulating MeCP2 in human hepatocellular carcinoma SMMC-7721 cells. *Toxicol Appl Pharmacol* **288**, 74-83, doi:10.1016/j.taap.2015.07.010 (2015).
36 Zuo, Y. & Song, Y. Detection and analysis of the methylation status of PTCH1 gene involved in

the hedgehog signaling pathway in a human gastric cancer cell line. *Experimental and therapeutic medicine* **6**, 1365-1368, doi:10.3892/etm.2013.1334 (2013).
37 Qu, Y., Dang, S. & Hou, P. Gene methylation in gastric cancer. *Clin Chim Acta* **424**, 53-65, doi:10.1016/j.cca.2013.05.002 (2013).
38 Peng, L. *et al.* Aberrant methylation of the PTCH1 gene promoter region in aberrant crypt foci. *International journal of cancer. Journal international du cancer* **132**, E18-25, doi:10.1002/ijc.27812 (2013).
39 Gialmanidis, I. P. *et al.* Expression of Bmi1, FoxF1, Nanog, and gamma-Catenin in Relation to Hedgehog Signaling Pathway in Human Non-small-Cell Lung Cancer. *Lung* **191**, 511-521, doi:10.1007/s00408-013-9490-4 (2013).
40 Qu, J. *et al.* Underestimated PTCH1 mutation rate in sporadic keratocystic odontogenic tumors. *Oral Oncol* **51**, 40-45, doi:10.1016/j.oraloncology.2014.09.016 (2015).
41 Smith, M. J. *et al.* Germline mutations in SUFU cause Gorlin syndrome-associated childhood medulloblastoma and redefine the risk associated with PTCH1 mutations. *J Clin Oncol* **32**, 4155-4161, doi:10.1200/JCO.2014.58.2569 (2014).
42 Saito, T. *et al.* PTCH1 mutation is a frequent event in oesophageal basaloid squamous cell carcinoma. *Mutagenesis* **30**, 297-301, doi:10.1093/mutage/geu072 (2015).
43 Endo, M., Su, L. & Nielsen, T. O. Activating transcription factor 2 in mesenchymal tumors. *Human Pathology* **45**, 276-284, doi:10.1016/j.humpath.2013.09.003 (2014).
44 Larabee, J. L., Shakir, S. M., Barua, S. & Ballard, J. D. Increased cAMP in Monocytes Augments Notch Signaling Mechanisms by Elevating RBP-J and Transducin-like Enhancer of Split (TLE). *Journal of Biological Chemistry* **288**, 21526-21536, doi:10.1074/jbc.M113.465120 (2013).
45 Fraga, M. F. *et al.* Epigenetic inactivation of the Groucho homologue gene TLE1 in hematologic malignancies. *Cancer Res* **68**, 4116-4122, doi:10.1158/0008-5472.CAN-08-0085 (2008).
46 Yao, X. *et al.* TLE1 promotes EMT in A549 lung cancer cells through suppression of E-cadherin. *Biochemical and Biophysical Research Communications* **455**, 277-284, doi:10.1016/j.bbrc.2014.11.007 (2014).
47 Duque-Afonso, J. *et al.* Comparative genomics reveals multistep pathogenesis of E2A-PBX1 acute lymphoblastic leukemia. *J Clin Invest* **125**, 3667-3680, doi:10.1172/JCI81158 (2015).
48 Wright, K. N. *et al.* Methyl supplementation attenuates cocaine-seeking behaviors and cocaine-induced c-Fos activation in a DNA methylation-dependent manner. *J Neurosci* **35**, 8948-8958, doi:10.1523/JNEUROSCI.5227-14.2015 (2015).
49 Burmeister, D. W. *et al.* The expression of RUNDC3B is associated with promoter methylation in lymphoid malignancies. *Hematological oncology*, doi:10.1002/hon.2238 (2015).
50 Risolino, M. *et al.* Transcription factor PREP1 induces EMT and metastasis by controlling the TGF-beta-SMAD3 pathway in non-small cell lung adenocarcinoma. *Proceedings of the National Academy of Sciences of the United States of America* **111**, E3775-E3784, doi:10.1073/pnas.1407074111 (2014).
51 Ikari, A., Sato, T., Watanabe, R., Yamazaki, Y. & Sugatani, J. Increase in claudin-2 expression by an EGFR/MEK/ERK/c-Fos pathway in lung adenocarcinoma A549 cells. *Bba-Mol Cell Res* **1823**, 1110-1118, doi:10.1016/j.bbamcr.2012.04.005 (2012).
52 Nakerakanti, S. S., Kapanadze, B., Yamasaki, M., Markiewicz, M. & Trojanowska, M. Fli1 and Ets1 have distinct roles in connective tissue growth factor/CCN2 gene regulation and induction of the profibrotic gene program. *Journal of Biological Chemistry* **281**, 25259-25269,

doi:10.1074/jbc.M600466200 (2006).
53 Park, J. T. *et al.* Repression of let-7 by transforming growth factor-beta1-induced Lin28 upregulates collagen expression in glomerular mesangial cells under diabetic conditions. *American journal of physiology. Renal physiology* **307**, F1390-1403, doi:10.1152/ajprenal.00458.2014 (2014).
54 Kaneto, C. M. *et al.* COL1A1 and miR-29b show lower expression levels during osteoblast differentiation of bone marrow stromal cells from Osteogenesis Imperfecta patients. *Bmc Med Genet* **15**, 45, doi:10.1186/1471-2350-15-45 (2014).
55 Zhu, Z. S. *et al.* An insertion/deletion polymorphism in the 3 ' untranslated region of type I collagen a2 (COL1A2) is associated with susceptibility for hepatocellular carcinoma in a Chinese population. *Cancer genetics* **204**, 265-269, doi:10.1016/j.cancergen.2011.03.007 (2011).
56 Rupard, J. H., Dimari, S. J., Damjanov, I. & Haralson, M. A. Synthesis of Type-I Homotrimer Collagen Molecules by Cultured Human-Lung Adenocarcinoma Cells. *American Journal of Pathology* **133**, 316-326 (1988).
57 Debnath, J. p66(Shc) and Ras: controlling anoikis from the inside-out. *Oncogene* **29**, 5556-5558, doi:10.1038/onc.2010.347 (2010).
58 Zhang, W. *et al.* Epigenetic enhancement of p66Shc during cellular replicative or premature senescence. *Toxicology* **278**, 189-194, doi:10.1016/j.tox.2010.07.011 (2010).
59 Northey, J. J. *et al.* Distinct phosphotyrosine-dependent functions of the ShcA adaptor protein are required for transforming growth factor beta (TGFbeta)-induced breast cancer cell migration, invasion, and metastasis. *Journal of Biological Chemistry* **288**, 5210-5222, doi:10.1074/jbc.M112.424804 (2013).
60 Zheng, Z. C. *et al.* Downregulated adaptor protein p66(Shc) mitigates autophagy process by low nutrient and enhances apoptotic resistance in human lung adenocarcinoma A549 cells. *Febs Journal* **280**, 4522-4530, doi:10.1111/febs.12416 (2013).
61 Hamada, S., Masamune, A., Miura, S., Satoh, K. & Shimosegawa, T. MiR-365 induces gemcitabine resistance in pancreatic cancer cells by targeting the adaptor protein SHC1 and pro-apoptotic regulator BAX. *Cell Signal* **26**, 179-185, doi:10.1016/j.cellsig.2013.11.003 (2014).
62 Wang, J. M. *et al.* MicroRNA miR-27b rescues bone marrow-derived angiogenic cell function and accelerates wound healing in type 2 diabetes mellitus. *Arterioscler Thromb Vasc Biol* **34**, 99-109, doi:10.1161/ATVBAHA.113.302104 (2014).
63 Fong, S. *et al.* Functional identification of distinct sets of antitumor activities mediated by the FKBP gene family. *Proc Natl Acad Sci U S A* **100**, 14253-14258, doi:10.1073/pnas.2332307100 (2003).
64 Bhushan, L. & Kandpal, R. P. EphB6 receptor modulates micro RNA profile of breast carcinoma cells. *PLoS One* **6**, e22484, doi:10.1371/journal.pone.0022484 (2011).
65 Ceyhan-Birsoy, O. *et al.* Recessive truncating titin gene, TTN, mutations presenting as centronuclear myopathy. *Neurology* **81**, 1205-1214 (2013).
66 Hasselbalch, H. C. *et al.* Whole Blood Transcriptional Profiling Reveals Deregulation of Oxidative and Antioxidative Defence Genes in Myelofibrosis and Related Neoplasms. Potential Implications of Downregulation of Nrf2 for Genomic Instability and Disease Progression. *PLoS One* **9**, e112786, doi:10.1371/journal.pone.0112786 (2014).
67 Liu, S. *et al.* Novel NTRK1 Frameshift Mutation in Congenital Insensitivity to Pain With Anhidrosis. *J Child Neurol* **30**, 1357-1361, doi:10.1177/0883073814552438 (2015).

68 Montalban, E. *et al.* MiR-21 is an Ngf-Modulated MicroRNA That Supports Ngf Signaling and Regulates Neuronal Degeneration in PC12 Cells. *Neuromol Med* **16**, 415-430, doi:10.1007/s12017-014-8292-z (2014).

69 Miele, E. *et al.* Characterization of medulloblastoma in Fanconi Anemia: a novel mutation in the BRCA2 gene and SHH molecular subgroup. *Biomarker research* **3**, 13, doi:10.1186/s40364-015-0038-z (2015).

70 Fan, H. *et al.* Genome-wide profiling of DNA methylation reveals preferred sequences of DNMTs in hepatocellular carcinoma cells. *Tumour Biol*, doi:10.1007/s13277-015-3202-z (2015).

71 Gurkan, A., Emingil, G., Afacan, B., Berdeli, A. & Atilla, G. Alpha 2 integrin gene (ITGA2) polymorphism in renal transplant recipients with and without drug induced gingival overgrowth. *Archives of Oral Biology* **59**, 283-288, doi:10.1016/j.archoralbio.2013.12.003 (2014).

72 Sen, N., Weingarten, M. & Peter, Y. Very Late Antigen-5 Facilitates Stromal Progenitor Cell Differentiation Into Myofibroblast. *Stem Cell Transl Med* **3**, 1342-1353, doi:10.5966/sctm.2014-0014 (2014).

73 Price, K. J. *et al.* Matrigel Basement Membrane Matrix influences expression of microRNAs in cancer cell lines. *Biochem Biophys Res Commun* **427**, 343-348, doi:10.1016/j.bbrc.2012.09.059 (2012).

74 Cong, D. *et al.* Expression profiles of pivotal microRNAs and targets in thyroid papillary carcinoma: an analysis of The Cancer Genome Atlas. *Oncotargets Ther* **8**, 2271-2277, doi:10.2147/Ott.S85753 (2015).

75 Zhang, X. *et al.* MicroRNA-26a promotes anoikis in human hepatocellular carcinoma cells by targeting alpha5 integrin. *Oncotarget* **6**, 2277-2289 (2015).

76 Ma, J. Y., Zhao, D., Wu, Y. Q., Xu, C. & Zhang, F. Q. Cyclic stretch induced gene expression of extracellular matrix and adhesion molecules in human periodontal ligament cells. *Archives of Oral Biology* **60**, 447-455, doi:10.1016/j.archoralbio.2014.11.019 (2015).

77 Viana, L. D. *et al.* Relationship between the Expression of the Extracellular Matrix Genes SPARC, SPP1, FN1, ITGA5 and ITGAV and Clinicopathological Parameters of Tumor Progression and Colorectal Cancer Dissemination. *Oncology* **84**, 81-91, doi:10.1159/000343436 (2013).

78 Ding, W. *et al.* Epigenetic Silencing of ITGA2 by MiR-373 Promotes Cell Migration in Breast Cancer. *PLoS One* **10**, e0135128, doi:10.1371/journal.pone.0135128 (2015).

79 Ahmed, Z. *et al.* Grb2 monomer-dimer equilibrium determines normal versus oncogenic function. *Nature Communications* **6**, 7354, doi:10.1038/Ncomms8354 (2015).

80 Rajala, R. V. S., Rajala, A. & Gupta, V. K. Conservation and divergence of Grb7 family of Ras-binding domains. *Protein & cell* **3**, 60-70, doi:10.1007/s13238-012-2001-1 (2012).

81 Ge, S., Xie, J., Liu, F., He, J. & He, J. MicroRNA-19b Reduces Hepatic Stellate Cell Proliferation by Targeting GRB2 in Hepatic Fibrosis Models In Vivo and In Vitro as Part of the Inhibitory Effect of Estradiol. *J Cell Biochem* **116**, 2455-2464, doi:10.1002/jcb.25116 (2015).

82 Wang, K. *et al.* The different expression profiles of microRNAs in elderly and young human dental pulp and the role of miR-433 in human dental pulp cells. *Mechanisms of ageing and development* **146-148**, 1-11, doi:10.1016/j.mad.2015.03.001 (2015).

83 Nagalingam, R. S. *et al.* A cardiac-enriched microRNA, miR-378, blocks cardiac hypertrophy by targeting Ras signaling. *Journal of Biological Chemistry* **288**, 11216-11232, doi:10.1074/jbc.M112.442384 (2013).

84 Zhou, Y. *et al.* The miR-106b~25 cluster promotes bypass of doxorubicin-induced senescence and increase in motility and invasion by targeting the E-cadherin transcriptional activator EP300. *Cell death and differentiation* **21**, 462-474, doi:10.1038/cdd.2013.167 (2014).

85 Romao, J. M., Jin, W., He, M., McAllister, T. & Guan le, L. MicroRNAs in bovine adipogenesis: genomic context, expression and function. *BMC Genomics* **15**, 137, doi:10.1186/1471-2164-15-137 (2014).

86 Lourdusamy, A., Rahman, R. & Grundy, R. G. Expression alterations define unique molecular characteristics of spinal ependymomas. *Oncotarget* **6**, 19780-19791 (2015).

87 Solomon, D. A., Kim, J. S. & Waldman, T. Cohesin gene mutations in tumorigenesis: from discovery to clinical significance. *BMB reports* **47**, 299-310 (2014).

88 Pon, J. R. *et al.* MEF2B mutations in non-Hodgkin lymphoma dysregulate cell migration by decreasing MEF2B target gene activation. *Nature Communications* **6**, 7953, doi:10.1038/ncomms8953 (2015).

89 Xu, F. Q. *et al.* Metastasis-associated lung adenocarcinoma transcript 1 promotes the proliferation of chondrosarcoma cell via activating Notch-1 signaling pathway. *Oncotargets Ther* **9**, 2143-2151, doi:10.2147/Ott.S100003 (2016).

90 Puente, X. S. *et al.* Non-coding recurrent mutations in chronic lymphocytic leukaemia. *Nature* **526**, 519-524, doi:10.1038/nature14666 (2015).

91 Pozzo, F. *et al.* NOTCH1 mutations associate with low CD20 level in chronic lymphocytic leukemia: evidence for a NOTCH1 mutation-driven epigenetic dysregulation. *Leukemia*, doi:10.1038/leu.2015.182 (2015).

92 Ketscher, A. *et al.* LSD1 controls metastasis of androgen-independent prostate cancer cells through PXN and LPAR6. *Oncogenesis* **3**, e120, doi:10.1038/oncsis.2014.34 (2014).

93 Kawada, I. *et al.* Paxillin mutations affect focal adhesions and lead to altered mitochondrial dynamics: relevance to lung cancer. *Cancer Biology & Therapy* **14**, 679-691, doi:10.4161/cbt.25091 (2013).

94 Kim, M. S., Yoo, N. J. & Lee, S. H. Absence of paxillin gene mutation in lung cancer and other common solid cancers. *Tumori* **97**, 211-213, doi:10.1700/667.7785 (2011).

95 Cai, H., Zhang, T., Tang, W. X. & Li, S. L. [Expression of paxillin in breast cancer cell with high and low metastatic potentiality]. *Sichuan da xue xue bao. Yi xue ban = Journal of Sichuan University. Medical science edition* **41**, 91-94 (2010).

96 Aouacheria, A. *et al.* In silico whole-genome scanning of cancer-associated nonsynonymous SNPs and molecular characterization of a dynein light chain tumour variant. *Oncogene* **24**, 6133-6142, doi:10.1038/sj.onc.1208745 (2005).

97 Vera, B., Rodriguez, A. D. & La Clair, J. J. Aplysqualenol A binds to the light chain of dynein type 1 (DYNLL1). *Angewandte Chemie* **50**, 8134-8138, doi:10.1002/anie.201102546 (2011).

Supplementary Material VI. Frequency of all candidate genes.

Ensembl ID	Gene symbol	Frequency
ENSP00000332353	PTCH1	6
ENSP00000344456	CTNNB1	6
ENSP00000357656	FYN	6
ENSP00000162330	BCAR1	5
ENSP00000297261	SHH	5
ENSP00000358525	NGF	5
ENSP00000361125	VEGFA	5
ENSP00000387662	GCG	5
ENSP00000261769	CDH1	4
ENSP00000264657	STAT3	4
ENSP00000277541	NOTCH1	4
ENSP00000296585	ITGA2	4
ENSP00000312652	LEP	4
ENSP00000350941	SRC	4
ENSP00000206249	ESR1	3
ENSP00000222725	LFNG	3
ENSP00000242577	DYNLL1	3
ENSP00000262965	TCF3	3
ENSP00000263253	EP300	3
ENSP00000268171	FURIN	3

ENSP00000287934	FZD1	3
ENSP00000293379	ITGA5	3
ENSP00000297268	COL1A2	3
ENSP00000297494	NOS3	3
ENSP00000300134	STAT6	3
ENSP00000300574	CRK	3
ENSP00000306245	FOS	3
ENSP00000322898	EBF1	3
ENSP00000331358	GAST	3
ENSP00000337088	MEN1	3
ENSP00000339328	PLAUR	3
ENSP00000340858	B2M	3
ENSP00000352262	MLL	3
ENSP00000359074	L1CAM	3
ENSP00000361850	PLAU	3
ENSP00000401303	SHC1	3
ENSP00000223023	WASL	2
ENSP00000227507	CCND1	2
ENSP00000228307	PXN	2
ENSP00000251849	RAF1	2
ENSP00000254942	TERF2	2
ENSP00000256443	CDK7	2

ENSP00000262613	SLC9A3R1	2
ENSP00000264033	CBL	2
ENSP00000264708	POMC	2
ENSP00000265335	RAD50	2
ENSP00000265709	ANK1	2
ENSP00000266970	CDK2	2
ENSP00000276603	TERF1	2
ENSP00000304669	CTNNA1	2
ENSP00000312435	DAG1	2
ENSP00000314458	CDC42	2
ENSP00000335153	HSP90AA1	2
ENSP00000338934	EZR	2
ENSP00000339007	GRB2	2
ENSP00000340698	GIPC1	2
ENSP00000340944	PTPN11	2
ENSP00000342215	KIR2DL3	2
ENSP00000345206	RBPJ	2
ENSP00000349467	CALM1	2
ENSP00000351209	EPHA2	2
ENSP00000351407	ARNT	2
ENSP00000351486	NTRK1	2
ENSP00000352514	RUNX2	2

ENSP00000354541	NLGN1	2
ENSP00000358022	MCL1	2
ENSP00000361423	ABL1	2
ENSP00000365682	TLE1	2
ENSP00000371138	FKBP1A	2
ENSP00000403005	EFNA4	2
ENSP00000405890	PBX1	2
ENSP00000223095	SERPINE1	1
ENSP00000228837	FGF6	1
ENSP00000242152	NPY	1
ENSP00000244007	PLCG1	1
ENSP00000244741	CDKN1A	1
ENSP00000254480	SMARCC1	1
ENSP00000256474	VHL	1
ENSP00000262186	KCNH2	1
ENSP00000262320	AXIN1	1
ENSP00000265171	EGF	1
ENSP00000267415	TINF2	1
ENSP00000269571	ERBB2	1
ENSP00000278616	ATM	1
ENSP00000288986	NCK1	1
ENSP00000293288	BAX	1

ENSP00000301838	FADD	1
ENSP00000302150	PRL	1
ENSP00000302269	VAV1	1
ENSP00000302665	IGF1	1
ENSP00000303830	INSR	1
ENSP00000306512	IL8	1
ENSP00000308541	F2	1
ENSP00000308938	PLG	1
ENSP00000309503	YWHAZ	1
ENSP00000318472	NCAM1	1
ENSP00000320866	CALR	1
ENSP00000329380	GP1BA	1
ENSP00000330633	CNTN2	1
ENSP00000332973	SMAD3	1
ENSP00000338018	HIF1A	1
ENSP00000338345	SNCA	1
ENSP00000344352	ATF3	1
ENSP00000348444	TTN(titin)	1
ENSP00000348986	INS-IGF2	1
ENSP00000354586	GLI2	1
ENSP00000354720	SMC3	1
ENSP00000368683	EDN1	1

ENSP00000407431	HLA-C	1
ENSP00000410294	FGFR2	1

Supplementary Material VII. Detailed Analysis of candidate genes of high frequencies

Three genes, PTCH1, CTNNB1, and FYN, have the highest frequency (six) in the candidate frequency list. PTCH1, as analyzed above, contributes to the process of tumorigenesis at multiple levels (mutations, mRNA, *etc.*) [1,2]. The mutations of PTCH1 have been proven to contribute to various subtypes of tumors, validating the fact that PTCH1 is a functional cancer driver gene [3,4]. Furthermore, at the same frequency, the candidate gene CTNNB1 has also been widely reported to be associated with tumor genesis of lung adenocarcinoma[5-8]. As an optimal cancer driver gene analyzed above, CTNNB1 has been confirmed to undeniably contribute to tumorigenesis, especially in the metastasis of specific tumor subtypes (*e.g.* lung adenocarcinoma) [9,10]. FYN is a high frequency gene not mentioned previously. As a functional member of the Src family tyrosine kinase, FYN has been widely reported to be a proto-oncogene which may directly contribute to the initiation and pathogenesis of various cancer subtypes, including lung adenocarcinoma[11-14]. As a PI-3K cascade associated gene, FYN has been reported to regulate the process of cell growth, survival, cell adhesion and integrin-mediated signaling [15-17]. Abnormity in such pathways have been reported to be directly related to the so-called precancerous lesions, implying that the abnormity of this gene may trigger the initiation of cancer [11,18]. Therefore, FYN is definitely a cancer driver gene of lung adenocarcinoma. Furthermore, this gene has also been reported to be related to drug resistance, validating the irreplaceable role of this gene during the process of tumorigenesis [19].

Five genes, i.e., BCAR1, SHH, NGF, VEGFA, and GCG, have a relatively high frequency (= 5), indicating that they may also contribute to tumorigenesis as specific cancer driver genes. BCAR1, although not in our optimal gene list mentioned above, has been widely reported to be a cancer driver gene, especially in breast cancer and lung adenocarcinoma[20-22]. BCAR1 is also a Src family kinase that is involved in various cellular events, especially those related to survival, proliferation and invasion processes [23]. As a docking protein for tyrosine kinase signaling, BCAR1 has been reported to contribute to the initiation of various cancer subyptes, especially of lung adenocarcinoma and breast cancer [24]. As we all know, the anti-estrogen characteristics of breast tumor cells promote the proliferation and invasion of breast cancer cells [25,26]. Accordingly, BCAR1 has been regarded as the core regulator and effector of the resistance feature of the cancer cells against estrogen, indicating that BCAR1 is definitely a functional cancer driver gene [27]. Apart from BCAR1, SHH (also known as Sonic Hedgehog), already mentioned as an optimal gene in a previous section, also shows quite a high frequency on our candidate list. This gene has been reported to contribute to the initiation and proliferation of tumors at least two levels (methylation and microRNA), which has been confirmed to be an actual cancer driver gene validating the accuracy of our prediction and screening [28-30]. NGF (known as Nerve Growth Factor), already mentioned in Section 3.4, is also on the list with a high frequency. NGF has been widely reported to contribute to the initiation and proliferation of specific tumor subtypes, including the lung adenocarcinoma and is regulated by

specific microRNAs and mutations, showing that this gene may actually be a core tumor driver gene [31-33]. During the initiation and invasion of a tumor, the reconstitution of the blood supply system may be one of the most fundamental and crucial processes for tumor survival, especially for lung adenocarcinoma [34-37]. Another gene with a high frequency on our prediction list is a vascular-associated gene, VEGFA. As a member of the PDGF/VEGF growth factor family, this gene contributes to various vascular-associated processes including mediating vascular permeability, inducing angiogenesis, promoting vasculogenesis and endothelial cell growth, which have all been reported to contribute to tumor angiogenesis processes during the initiation of tumorigenesis [38-40]. Therefore, regulating the blood supply of tumor tissues, VEGFA is definitely a cancer driver gene, verifying the accuracy of our prediction algorithm. Another candidate gene with a frequency of five is GCG (Glucagon), which has not been mentioned yet. This gene is actually a preproprotein which is usually cleaved into four distinct mature peptides with distinct functions [41,42]. Widely reported in pancreatic cancer, one splicing isoform of GCG (glucagon-like peptide-1) can actually drive the initiation of specific cancer subtypes, confirming our prediction of GCG as a cancer driver gene [43]. Although such gene haven't been reported to function in lung adenocarcinoma, the strong tumor genesis function of such gene indicates its underlying functional role for lung adenocarcinoma.

Furthermore, candidate genes with a frequency of four have also been confirmed to be functional cancer driver genes, reflecting the accuracy of our prediction. In addition to genes like NOTCH1 and ITGA2, which were analyzed above, four other candidate genes are also strongly suggested to be associated with the initiation and proliferation of tumors. CDH1, encoding cadherin 1, regulate the cell-cell adhesion which may further regulate the proliferation of cells especially in solid tumor tissues, especially in lung adenocarcinoma[44-48]. As a calcium dependent cell-cell adhesion glycoprotein comprised of five extracellular cadherin repeats, the abnormal expression and mutation of this gene has been shown to contribute to the abnormal proliferation process of tumor cells and further initiate tumorigenesis, validating our prediction of CDH1 as a core cancer drive gene [49-51]. Another gene, i.e., STAT3, encodes a functional transcription factor which has been confirmed to participate in the proliferation and survival process of living cells [52]. The abnormal expression and specific mutation in STAT3 have both been reported in various tumor subtypes including lung adenocarcinoma, suggesting that the dysfunctions of this gene may contribute to the tumorigenesis process [53,54]. Another two genes, i.e., SRC and LEP, contribute to tumorigenesis in their respective ways. SRC with a frequency of 4 has been screened out as a cancer driver candidate gene by our algorithm. Just like FYN and BCAR1, SRC encodes a core member of the Src family as a functional proto-oncogene, which has been widely reported in breast cancer and lung adenocarcinoma[55-57]. In accordance with our prediction, SRC has been reported to initiate tumorigenesis by abnormally activating the PI-3K cascade, which is regarded as one of the core pathways regulating cell proliferation in lung adenocarcinoma[58,59]. Therefore, SRC can be clustered into cancer driver genes, validating the prediction accuracy of our algorithm. LEP, as a functional gene with a high frequency (four), has been generally regarded as a metabolism related gene which

has proven to be associated with morbid obesity [60-62]. As a functional regulatory of nutrition intake, leptin encoded by LEP has proven to be associated with chronic inflammation, which is also regarded as one of the main origins of precancerous lesions, indicating the potential cancer driver role of this gene [63]. Furthermore, the serum level of leptin has proven to be associated with the initiation, proliferation and drug-resistance processes of cancer cells, especially for lung adenocarcinoma [64]. Interacting with specific regulatory factors (i.e., transcription factors, cytokines, *etc.*) in tumor cells and in the micro-environment, LEP has been confirmed to actually play a promotive role in tumorigenesis, validating the cancer driver role of LEP in various tumor subtypes [65,66].

As we have analyzed above, candidate genes with high frequencies in our prediction list have been extensively proven to be actual cancer driver genes for lung adenocarcinoma. The higher the frequency, the more relevant the gene is to tumorigenesis. Even genes with fewer frequencies still show strong tendencies to be functional tumor driver genes (MLL, EGF, *etc.*) [67-70]. Screening for genes with high frequencies in our prediction list may be an effective way to identify functional cancer driver genes. Such a result verifies the accuracy and effectiveness of our newly presented method in identifying new cancer driver genes.

References

1 Qu, J. F. *et al.* Underestimated PTCH1 mutation rate in sporadic keratocystic odontogenic tumors. *Oral Oncology* **51**, 40-45, doi:10.1016/j.oraloncology.2014.09.016 (2015).

2 Zuo, Y. & Song, Y. Detection and analysis of the methylation status of PTCH1 gene involved in the hedgehog signaling pathway in a human gastric cancer cell line. *Experimental and therapeutic medicine* **6**, 1365-1368, doi:10.3892/etm.2013.1334 (2013).

3 Smith, M. J. *et al.* Germline Mutations in SUFU Cause Gorlin Syndrome-Associated Childhood Medulloblastoma and Redefine the Risk Associated With PTCH1 Mutations. *Journal of Clinical Oncology* **32**, 4155-U4391, doi:10.1200/Jco.2014.58.2569 (2014).

4 Saito, T. *et al.* PTCH1 mutation is a frequent event in oesophageal basaloid squamous cell carcinoma. *Mutagenesis* **30**, 297-301, doi:10.1093/mutage/geu072 (2015).

5 Zhang, S. *et al.* Pygopus-2 promotes invasion and metastasis of hepatic carcinoma cell by decreasing E-cadherin expression. *Oncotarget* **6**, 11074-11086 (2015).

6 Teo, A. E. D. *et al.* Pregnancy, Primary Aldosteronism, and Adrenal CTNNB1 Mutations. *New England Journal of Medicine* **373**, 1429-1436, doi:10.1056/NEJMoa1504869 (2015).

7 Crago, A. M. *et al.* Near universal detection of alterations in CTNNB1 and Wnt pathway regulators in desmoid-type fibromatosis by whole-exome sequencing and genomic analysis. *Gene Chromosome Canc* **54**, 606-615, doi:10.1002/gcc.22272 (2015).

8 Dahlin, A. M. *et al.* CCND2, CTNNB1, DDX3X, GLI2, SMARCA4, MYC, MYCN, PTCH1, TP53, and MLL2 gene variants and risk of childhood medulloblastoma. *Journal of neuro-oncology* **125**, 75-78, doi:10.1007/s11060-015-1891-1 (2015).

9 Lee, C. I. *et al.* CTNNB1 (beta-catenin) mutation is rare in brain tumours but involved as a sporadic event in a brain metastasis. *Acta Neurochir* **151**, 1107-1111, doi:10.1007/s00701-009-0242-4 (2009).

10 Xie, C. Y. *et al.* C-Myc participates in beta-catenin-mediated drug resistance in A549/DDP lung

adenocarcinoma cells. *APMIS : acta pathologica, microbiologica, et immunologica Scandinavica* **122**, 1251-1258, doi:10.1111/apm.12296 (2014).

11 Chen, H. Y., Yang, Y. M., Stevens, B. M. & Noble, M. Inhibition of redox/Fyn/c-Cbl pathway function by Cdc42 controls tumour initiation capacity and tamoxifen sensitivity in basal-like breast cancer cells. *Embo Molecular Medicine* **5**, 723-736, doi:10.1002/emmm.201202140 (2013).

12 Hwang, M. K., Kang, N. J., Heo, Y. S., Lee, K. W. & Lee, H. J. Fyn kinase is a direct molecular target of delphinidin for the inhibition of cyclooxygenase-2 expression induced by tumor necrosis factor-alpha. *Biochemical Pharmacology* **77**, 1213-1222, doi:10.1016/j.bcp.2008.12.021 (2009).

13 Grabulovski, D., Kaspar, M. & Neri, D. A novel, non-immunogenic Fyn SH3-derived binding protein with tumor vascular targeting properties. *Journal of Biological Chemistry* **282**, 3196-3204, doi:10.1074/jbc.M609211200 (2007).

14 Masaki, T. *et al.* pp60c-src activation in lung adenocarcinoma. *European journal of cancer* **39**, 1447-1455 (2003).

15 Matsumura, Y. *et al.* Transient Helical Structure during PI3K and Fyn SH3 Domain Folding. *J Phys Chem B* **117**, 4836-4843, doi:10.1021/jp400167s (2013).

16 Yang, X. *et al.* A topical PI3K/mTOR inhibitor induces regression of squamous cell carcinomas in K14-Fyn Y528F mice. *Journal of Investigative Dermatology* **132**, S25-S25 (2012).

17 Yadav, V. & Denning, M. F. Fyn Is Induced by Ras/PI3K/Akt Signaling and Is Required for Enhanced Invasion/Migration. *Molecular Carcinogenesis* **50**, 346-352, doi:10.1002/mc.20716 (2011).

18 Filipp, D., Leung, B. L., Zhang, J., Veillette, A. & Julius, M. Enrichment of Lck in lipid rafts regulates colocalized Fyn activation and the initiation of proximal signals through TCR alpha beta. *Journal of immunology* **172**, 4266-4274 (2004).

19 Elias, D. & Ditzel, H. J. Fyn is an important molecule in cancer pathogenesis and drug resistance. *Pharmacological research : the official journal of the Italian Pharmacological Society* **100**, 250-254, doi:10.1016/j.phrs.2015.08.010 (2015).

20 Konstantinovsky, S., Davidson, B. & Reich, R. Ezrin and BCAR1/p130Cas mediate breast cancer growth as 3-D spheroids. *Clin Exp Metastasis* **29**, 527-540, doi:10.1007/s10585-012-9468-2 (2012).

21 Kumbrink, J. & Kirsch, K. H. Regulation of p130(Cas)/BCAR1 expression in tamoxifen-sensitive and tamoxifen-resistant breast cancer cells by EGR1 and NAB2. *Neoplasia* **14**, 108-120 (2012).

22 Huang, W. *et al.* BCAR1 protein plays important roles in carcinogenesis and predicts poor prognosis in non-small-cell lung cancer. *PLoS One* **7**, e36124, doi:10.1371/journal.pone.0036124 (2012).

23 Grebenchtchikov, N. *et al.* Development of an ELISA for measurement of BCAR1 protein in human breast cancer tissue. *Clin Chem* **50**, 1356-1363, doi:10.1373/clinchem.2003.029868 (2004).

24 Schlaepfer, D. D., Broome, M. A. & Hunter, T. Fibronectin-stimulated signaling from a focal adhesion kinase-c-Src complex: involvement of the Grb2, p130cas, and Nck adaptor proteins. *Mol Cell Biol* **17**, 1702-1713 (1997).

25 Wallez, Y., Riedl, S. J. & Pasquale, E. B. Association of the breast cancer antiestrogen resistance protein 1 (BCAR1) and BCAR3 scaffolding proteins in cell signaling and antiestrogen resistance. *J Biol Chem* **289**, 10431-10444, doi:10.1074/jbc.M113.541839 (2014).

26 Brinkman, A. *et al.* The substrate domain of BCAR1 is essential for anti-estrogen-resistant proliferation of human breast cancer cells. *Breast Cancer Research and Treatment* **120**, 401-408, doi:10.1007/s10549-009-0403-4 (2010).

27 Brinkman, A., van der Flier, S., Kok, E. M. & Dorssers, L. C. J. BCAR1, a human homologue of the adapter protein p130Cas, and antiestrogen resistance in breast cancer cells. *Journal of the National Cancer Institute* **92**, 112-120, doi:DOI 10.1093/jnci/92.2.112 (2000).

28 Miele, E. *et al.* Characterization of medulloblastoma in Fanconi Anemia: a novel mutation in the BRCA2 gene and SHH molecular subgroup. *Biomarker research* **3**, 13, doi:10.1186/s40364-015-0038-z (2015).

29 ten Haaf, A. *et al.* Paradox of sonic hedgehog (SHH) transcriptional regulation: Alternative transcription initiation overrides the effect of downstream promoter DNA methylation. *Epigenetics* **6**, 465-477 (2011).

30 Yakushiji, N. *et al.* Correlation between Shh expression and DNA methylation status of the limb-specific Shh enhancer region during limb regeneration in amphibians. *Dev Biol* **312**, 171-182, doi:10.1016/j.ydbio.2007.09.022 (2007).

31 Montalban, E. *et al.* MiR-21 is an Ngf-Modulated MicroRNA That Supports Ngf Signaling and Regulates Neuronal Degeneration in PC12 Cells. *Neuromol Med* **16**, 415-430, doi:10.1007/s12017-014-8292-z (2014).

32 Aubert, L. *et al.* NGF-induced TrkA/CD44 association is involved in tumor aggressiveness and resistance to lestaurtinib. *Oncotarget* **6**, 9807-9819 (2015).

33 Hayakawa, Y. *et al.* NGF Promotes Gastrointestinal Cancer Development Through Tumor-Associated Neurogenesis. *Gastroenterology* **148**, S75-S75 (2015).

34 Wang, W. F., Zhang, E. D. & Lin, C. J. MicroRNAs in tumor angiogenesis. *Life sciences* **136**, 28-35, doi:10.1016/j.lfs.2015.06.025 (2015).

35 Yan, J. H., Zhao, C. L., Ding, L. B. & Zhou, X. FOXD3 suppresses tumor growth and angiogenesis in non-small cell lung cancer. *Biochemical and Biophysical Research Communications* **466**, 111-116, doi:10.1016/j.bbrc.2015.08.116 (2015).

36 Paiva, T. F. *et al.* Angiogenesis-related protein expression in bevacizumab-treated metastatic colorectal cancer: NOTCH1 detrimental to overall survival. *BMC Cancer* **15**, 643, doi:10.1186/s12885-015-1648-4 (2015).

37 Li, Z. *et al.* miR-506 Inhibits Epithelial-to-Mesenchymal Transition and Angiogenesis in Gastric Cancer. *American Journal of Pathology* **185**, 2412-2420, doi:10.1016/j.ajpath.2015.05.017 (2015).

38 Xu, M. *et al.* Sorafenib Blocks the HIF-1 alpha/VEGFA Pathway, Inhibits Tumor Invasion, and Induces Apoptosis in Hepatoma Cells. *DNA and cell biology* **33**, 275-281, doi:10.1089/dna.2013.2184 (2014).

39 Zhou, B. S. *et al.* MicroRNA-503 targets FGF2 and VEGFA and inhibits tumor angiogenesis and growth. *Cancer letters* **333**, 159-169, doi:10.1016/j.canlet.2013.01.028 (2013).

40 Claesson-Welsh, L. & Welsh, M. VEGFA and tumour angiogenesis. *J Intern Med* **273**, 114-127, doi:10.1111/joim.12019 (2013).

41 Robbins, R. A. & Hamel, F. G. Chemotactic Factor Inactivator Interaction with Gc-Globulin (Vitamin-D-Binding Protein) - a Mechanism of Modulating the Chemotactic Activity of C5a. *Journal of immunology* **144**, 2371-2376 (1990).

42 Gault, V. A., Bhat, V. K., Irwin, N. & Flatt, P. R. A Novel Glucagon-like Peptide-1 (GLP-1)/Glucagon

Hybrid Peptide with Triple-acting Agonist Activity at Glucose-dependent Insulinotropic Polypeptide, GLP-1 and Glucagon Receptors and Therapeutic Potential in High Fat-fed Mice. *Journal of Biological Chemistry* **288**, 35581-35591, doi:10.1074/jbc.M113.512046 (2013).

43 Zhao, H. *et al.* Activation of glucagon-like peptide-1 receptor inhibits growth and promotes apoptosis of human pancreatic cancer cells in a cAMP-dependent manner. *American journal of physiology. Endocrinology and metabolism* **306**, E1431-1441, doi:10.1152/ajpendo.00017.2014 (2014).

44 Pluciennik, E. *et al.* The role of WWOX tumor suppressor gene in the regulation of EMT process via regulation of CDH1-ZEB1-VIM expression in endometrial cancer. *International journal of oncology* **46**, 2639-2648, doi:10.3892/ijo.2015.2964 (2015).

45 Schreiber, S. C. *et al.* Polysialylated NCAM represses E-cadherin-mediated cell-cell adhesion in pancreatic tumor cells. *Gastroenterology* **134**, 1555-1566, doi:10.1053/j.gastro.2008.02.023 (2008).

46 Kang, H. G. *et al.* E-cadherin cell-cell adhesion in Ewing tumor cells mediates suppression of anoikis through activation of the ErbB4 tyrosine kinase. *Cancer Research* **67**, 3094-3105, doi:10.1158/0008-5472.Can-06-3259 (2007).

47 Margulis, A. *et al.* Abrogation of E-cadherin-mediated adhesion induces tumor cell invasion in human skin-like organotypic culture. *Journal of Investigative Dermatology* **121**, 1182-1190, doi:DOI 10.1046/j.1523-1747.2003.12523.x (2003).

48 Kato, Y. *et al.* A proteomic profiling of laser-microdissected lung adenocarcinoma cells of early lepidic-types. *Clin Transl Med* **4**, 64, doi:10.1186/s40169-015-0064-3 (2015).

49 Lajus, T. B. P. & Sales, R. M. D. CDH1 germ-line missense mutation identified by multigene sequencing in a family with no history of diffuse gastric cancer. *Gene* **568**, 215-219, doi:10.1016/j.gene.2015.05.035 (2015).

50 Choi, H. J., Ki, C. S., Suh, S. P. & Kim, J. W. Presymptomatic Identification of CDH1 Germline Mutation in a Healthy Korean Individual with Family History of Gastric Cancer. *Ann Lab Med* **34**, 386-389, doi:10.3343/alm.2014.34.5.386 (2014).

51 Oliveira, C. *et al.* Quantification of Epigenetic and Genetic 2nd Hits in CDH1 During Hereditary Diffuse Gastric Cancer Syndrome Progression. *Gastroenterology* **136**, 2137-2148, doi:10.1053/j.gastro.2009.02.065 (2009).

52 Bosch-Barrera, J. & Menendez, J. A. Silibinin and STAT3: A natural way of targeting transcription factors for cancer therapy. *Cancer Treatment Reviews* **41**, 540-546, doi:10.1016/j.ctrv.2015.04.008 (2015).

53 Rajala, H. L. M., Porkka, K., Maciejewski, J. P., Loughran, T. P. & Mustjoki, S. Uncovering the pathogenesis of large granular lymphocytic leukemia-novel STAT3 and STAT5b mutations. *Ann Med* **46**, 114-122, doi:10.3109/07853890.2014.882105 (2014).

54 Wang, X., Crowe, P. J., Goldstein, D. & Yang, J. L. STAT3 inhibition, a novel approach to enhancing targeted therapy in human cancers (review). *International journal of oncology* **41**, 1181-1191, doi:10.3892/ijo.2012.1568 (2012).

55 Elsberger, B. Translational evidence on the role of Src kinase and activated Src kinase in invasive breast cancer. *Crit Rev Oncol Hematol* **89**, 343-351, doi:10.1016/j.critrevonc.2013.12.009 (2014).

56 Goel, R. K. & Lukong, K. E. Tracing the footprints of the breast cancer oncogene BRK - Past till present. *Biochim Biophys Acta* **1856**, 39-54, doi:10.1016/j.bbcan.2015.05.001 (2015).

57 Diao, Y. *et al.* Dasatinib promotes paclitaxel-induced necroptosis in lung adenocarcinoma with phosphorylated caspase-8 by c-Src. *Cancer letters* **379**, 12-23, doi:10.1016/j.canlet.2016.05.003 (2016).

58 Villegas-Comonfort, S., Castillo-Sanchez, R., Serna-Marquez, N., Cortes-Reynosa, P. & Salazar, E. P. Arachidonic acid promotes migration and invasion through a PI3K/Akt-dependent pathway in MDA-MB-231 breast cancer cells. *Prostag Leukotr Ess* **90**, 169-177, doi:10.1016/j.plefa.2014.01.007 (2014).

59 Zhao, Y. *et al.* Distinctive regulation and function of PI 3K/Akt and MAPKs in doxorubicin-induced apoptosis of human lung adenocarcinoma cells. *J Cell Biochem* **91**, 621-632, doi:10.1002/jcb.10751 (2004).

60 Saeed, S. *et al.* Genetic variants in LEP, LEPR, and MC4R explain 30% of severe obesity in children from a consanguineous population. *Obesity* **23**, 1687-1695, doi:10.1002/oby.21142 (2015).

61 Collares, R. V. A., Salgado, W., Tirapelli, D. P. D. & dos Santos, J. S. The Expression of LEP, LEPR, IGF1 and IL10 in Obesity and the Relationship with microRNAs. *PLoS One* **9**, e93512, doi:10.1371/journal.pone.0093512 (2014).

62 Gregoor, J. G. *et al.* Polymorphisms of the LEP, LEPR and HTR2C gene: obesity and BMI change in patients using antipsychotic medication in a naturalistic setting. *Pharmacogenomics* **12**, 919-923, doi:10.2217/Pgs.11.40 (2011).

63 Ahbap, E. *et al.* Relationship between relative interdialytic weight gain and serum leptin levels, nutrition, and inflammation in chronic hemodialysis patients. *Clinical Nephrology* **83**, 154-160, doi:10.5414/Cn108450 (2015).

64 Artac, M. *et al.* Serum leptin level and waist-to-hip ratio (WHR) predict the overall survival of metastatic breast cancer (MBC) patients treated with aromatase inhibitors (AIs). *Breast Cancer-Tokyo* **20**, 174-180, doi:10.1007/s12282-011-0322-1 (2013).

65 Rehem, R. A., Elwafa, W. A., Elwafa, R. A. & Abdel-Aziz, T. E. Study of Serum Leptin in Well-differentiated Thyroid Carcinoma: Correlation with Patient and Tumor Characteristics. *World J Surg* **38**, 2621-2627, doi:10.1007/s00268-014-2634-8 (2014).

66 Shahramian, I., Noori, N. M., Hashemi, M., Sharafi, E. & Baghbanian, A. A study of serum levels of leptin, ghrelin and tumour necrosis factor-alpha in child patients with cyanotic and acyanotic, congenital heart disease. *J Pak Med Assoc* **63**, 1332-1337 (2013).

67 Jiang, X. *et al.* MiR-495 is a tumor-suppressor microRNA down-regulated in MLL-rearranged leukemia. *Proc Natl Acad Sci U S A* **109**, 19397-19402, doi:10.1073/pnas.1217519109 (2012).

68 Tamai, H. *et al.* Resistance of MLL-AFF1-positive acute lymphoblastic leukemia to tumor necrosis factor-alpha is mediated by S100A6 upregulation. *Blood Cancer J* **1**, e38, doi:10.1038/bcj.2011.37 (2011).

69 Bahr, H. I. *et al.* Chemopreventive effect of leflunomide against Ehrlich's solid tumor grown in mice: Effect on EGF and EGFR expression and tumor proliferation. *Life sciences* **141**, 193-201, doi:10.1016/j.lfs.2015.10.003 (2015).

70 Elbaz, M. *et al.* Modulation of the tumor microenvironment and inhibition of EGF/EGFR pathway: novel anti-tumor mechanisms of Cannabidiol in breast cancer. *Mol Oncol* **9**, 906-919, doi:10.1016/j.molonc.2014.12.010 (2015).
